# A novel integrative multi-omics approach to unravel the genetic determinants of rare diseases with application in sinusoidal obstruction syndrome

**DOI:** 10.1371/journal.pone.0281892

**Published:** 2023-04-05

**Authors:** Nicolas Waespe, Simona Jurkovic Mlakar, Isabelle Dupanloup, Mohamed Aziz Rezgui, Henrique Bittencourt, Maja Krajinovic, Claudia E. Kuehni, Tiago Nava, Marc Ansari

**Affiliations:** 1 Institute of Social and Preventive Medicine, Childhood Cancer Research Group, University of Bern, Bern, Switzerland; 2 Faculty of Medicine, Department of Pediatrics, Gynecology and Obstetrics, CANSEARCH Research Platform in Pediatric Oncology and Hematology, University of Geneva, Geneva, Switzerland; 3 Graduate School for Cellular and Biomedical Sciences (GCB), University of Bern, Bern, Switzerland; 4 Swiss Institute of Bioinformatics, Lausanne, Switzerland; 5 Department of Pediatrics, Charles-Bruneau Cancer Center, CHU Sainte-Justine Research Center, Montreal, QC, Canada; 6 Department of Pediatrics, Clinical Pharmacology Unit, CHU Sainte-Justine, Montreal, QC, Canada; 7 Faculty of Medicine, Department of Pharmacology, University of Montreal, Montreal, QC, Canada; 8 Department of Pediatrics, Division of Pediatric Oncology and Hematology, University Hospital of Bern, Bern, Switzerland; 9 Department of Women, Child and Adolescent, Division of Pediatric Oncology and Hematology, Geneva University Hospitals and University of Geneva, Geneva, Switzerland; University of Vermont, UNITED STATES

## Abstract

**Background:**

Genotype-phenotype analyses of rare diseases often suffer from a lack of power, due to small sample size, which makes identifying significant associations difficult. Sinusoidal obstruction syndrome (SOS) of the liver is a rare but life-threatening complication of hematopoietic stem cell transplantation (HSCT). The alkylating agent busulfan is commonly used in HSCT and known to trigger SOS. We developed a novel pipeline to identify genetic determinants in rare diseases by combining *in vitro* information with clinical whole-exome sequencing (WES) data and applied it in SOS patients and controls.

**Methods:**

First, we analysed differential gene expression in six lymphoblastoid cell lines (LCLs) before and after incubation with busulfan. Second, we used WES data from 87 HSCT patients and estimated the association with SOS at the SNP and the gene levels. We then combined the results of the expression and the association analyses into an association statistic at the gene level. We used an over-representation analysis to functionally characterize the genes that were associated with a significant combined test statistic.

**Results:**

After treatment of LCLs with busulfan, 1708 genes were significantly up-, and 1385 down-regulated. The combination of the expression experiment and the association analysis of WES data into a single test statistic revealed 35 genes associated with the outcome. These genes are involved in various biological functions and processes, such as “*Cell growth and death*”, “*Signalling molecules and interaction*”, “*Cancer*”, and “*Infectious disease*”.

**Conclusions:**

This novel data analysis pipeline integrates two independent omics datasets and increases statistical power for identifying genotype-phenotype associations. The analysis of the transcriptomics profile of cell lines treated with busulfan and WES data from HSCT patients allowed us to identify potential genetic contributors to SOS. Our pipeline could be useful for identifying genetic contributors to other rare diseases where limited power renders genome-wide analyses unpromising.

**Trial registration:**

For the clinical dataset: Clinicaltrials.gov: NCT01257854. https://clinicaltrials.gov/ct2/history/NCT01257854.

## 1. Introduction

Sinusoidal obstruction syndrome (SOS) of the liver is a serious, sometimes life-threatening complication of chemotherapies and hematopoietic stem cell transplantation (HSCT) [1]. SOS commonly arises from endothelial damage and hepatic injury which are triggered by the conditioning regimens given prior to HSCT [2]. Known risk factors for SOS are treatment with multiple alkylating antineoplastic agents, younger age at HSCT, underlying disease, previous liver damage, iron overload, HSCT-specific factors such as myeloablative versus reduced-intensity conditioning regimens, donor type, number of previous HSCTs, and concomitant hepatotoxic treatments [3–5]. Particularly the alkylating agent busulfan which is used in a large proportion of patients undergoing HSCT shows a strong and dose-dependent association with SOS impacting survival [6–8]. Mouse models identified the exposure to busulfan as initiating factor leading to endothelial cell injury and later SOS [9]. The further mechanisms involved in SOS are complex. Initial endothelial cell damage caused by the conditioning regimen leads to inflammation resulting in activation of the coagulation cascade leading to microthrombi in the liver microvasculature [10,11] and obstruction of the centrilobular vein of the liver [12]. The outflow obstruction in the liver causes additional damage to the hepatic cells and leads to painful hepatomegaly, jaundice, and liver dysfunction. In severe cases, patients may develop multiple organ failure with a mortality rate of over 80% [5]. Defibrotide, an anticoagulant and anti-inflammatory drug, is an effective treatment if administered early in the course of the disease [13,14]. Also, prophylactic treatment with defibrotide starting simultaneously with the conditioning regimen in high-risk populations is feasible and effective but not without side effects [15]. To target the right patients for prophylaxis, we need to identify the risk factors associated with SOS, including genetic contributors.

Various studies have reported germline genetic variants in association with SOS. Most of these studies have used a candidate gene approach. They identified several genetic determinants of SOS in genes that encode for detoxification enzymes, particularly glutathione S transferases such as *GSTA1* [16–18], enzymes which affect glutathione levels (e.g. cystathionine gamma-lyase *[CTH]*) [19]), oxidative liver injury (e.g. methylenetetrahydrofolate reductase *[MTHFR]*) [19,20], the iron metabolism (e.g. homeostatic iron regulator *[HFE]*) [21], and urea cycle (e.g. carbamoyl phosphate synthetase I *[CPS1]*) [21]. We previously performed an exome-wide association analysis focusing on exonic variants with predicted functional impact and those in adjacent untranslated regions (UTRs) in patients undergoing busulfan-based HSCT. We identified genetic variants in the *UGT2B10* and *LNPK* genes, which were replicated in an independent population [22]. The polymorphisms that were found to be associated with SOS in previous studies were not validated in the exome-wide discovery analysis, which likely reflects the coverage of the exome-specific analysis and selection of functional variants. Currently, our knowledge of the genetic predictors for SOS is still very limited, identified associations were only inconsistently replicated [23], and novel approaches should be developed to estimate the genetic contribution to SOS.

Since a majority of studies addressing complications of HSCT and similar other rare conditions lack the power to identify genetic predictors due to the small sample size, we used a strategy that is built on the strong link between the exposure to busulfan and the onset of SOS [24]. We generated transcriptomic data from lymphoblastoid cell lines (LCLs) exposed to busulfan *in vitro* and evaluated gene expression changes following treatment. We used the raw whole-exome sequencing (WES) data published previously [22]. Additional filtering steps on the WES data were applied to reduce genetic heterogeneity in the sample, prior to conducting the association analysis. We performed the association analysis with SOS at the SNP and gene level. Importantly, we combined the results of the association analysis and the transcriptomic analysis into a test statistic which measures the strength of association with the phenotype of interest, at the gene level.

## 2. Materials and methods

### 2.1. Design of the study

**Fig 1:**
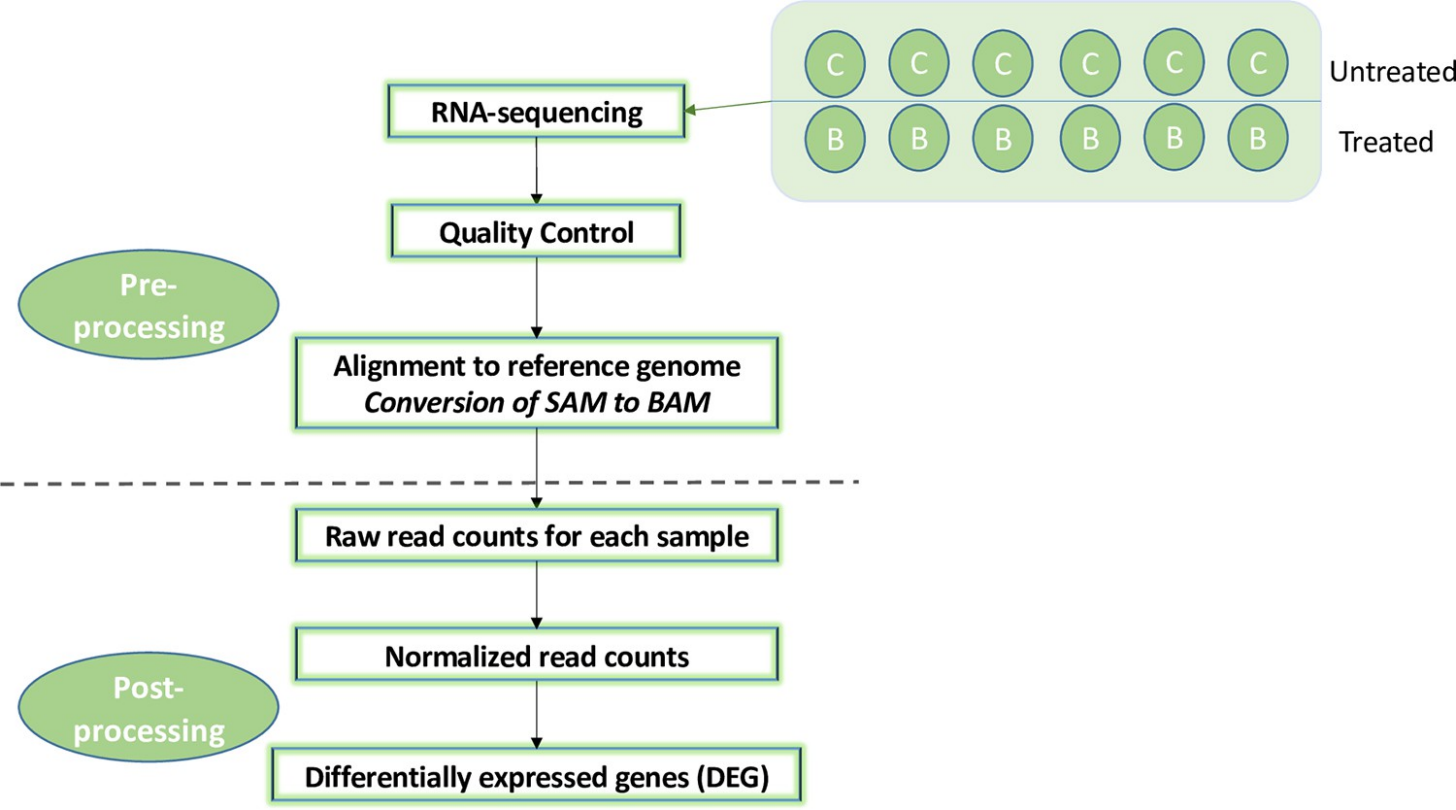
Flowchart of the transcriptomic data analysis pipeline in 6 lymphoblastic cell lines with and without *in vitro* busulfan treatment. Legend: BAM, Binary Alignment Map; DEG, differentially expressed genes; RNA, ribonucleic acid; SAM, Sequence Alignment Map; WES, whole-exome sequencing.

**Fig 2:**
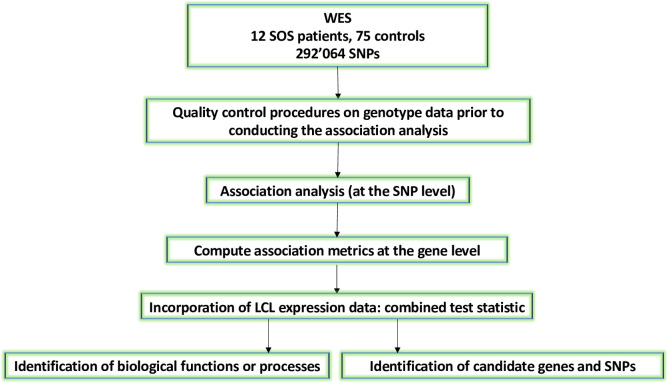
Flowchart of the data analysis pipeline combining whole-exome sequencing of predominantly pediatric patients with *in vitro* transcriptomic data. **Legend:** LCL, lymphoblastoid cell line; SNP, single nucleotide polymorphism; SOS, sinusoidal obstruction syndrome; WES, whole-exome sequencing.

We developed a data analysis pipeline which includes the following steps:

First, we performed a differential gene expression analysis of LCLs before and after *in vitro* exposure to 100 μM busulfan **(Fig 1).** Second, we performed an association analysis with SOS at the SNP level, using the raw whole-exome sequencing (WES) data published previously [22], after the application of a series of filtering steps to reduce genetic heterogeneity in the data [25]. Third, we used a gene-based test for association, which allows us to combine the effects of all SNPs in a gene into one test statistic while correcting for linkage disequilibrium (VEGAS2) [26]. A gene-based approach allows achieving greater power for identifying relevant genes by (i) detecting significant associations through the combination of several SNPs showing marginal levels of significance that are often indistinguishable from random noise in the initial WES results [26], and (ii) reducing the multiple-testing problem of WES analysis by considering statistical tests for approximately 20,000 genes per genome as opposed to testing hundreds of thousands of SNPs in a typical exome-wide analysis [26]. Then, we computed a gene-based score, and its associated p-value, by combining the results of the differential gene expression analysis and the gene-based test for association. By integrating the expression and association evidence at the gene level, we increased the power to detect genetic determinants of the clinical outcome of interest, since (i) the resolution of our approach should be higher than a classical exome-wide analysis, as we combine evidence from independent experiments, and (ii) the resolution of our approach should be higher than a candidate gene study, as we don’t focus exclusively on a few candidates, selected a priori. This increase in power relies on the following assumption: genetic variants which predispose to SOS are found preferentially in genes which expression level is affected by the treatment. Fourth, we performed a functional analysis, which consists of identifying pathways and gene sets that are over-represented in the list of significantly associated genes. Fifth, we estimated polygenic risk scores (PRS) for the cases and the controls for which the exome data was available [22]. These scores were computed as the sum of the number of risk alleles carried at each variant, weighted by their effect size **(Fig 2).**

### 2.2 Transcriptomic analysis of lymphoblastoid cell lines

We collected RNA sequencing data on LCLs (Coriell Cell Repository, Camden, NJ, USA) before and after exposure to busulfan. Briefly, we performed true-seq RNA-sequencing of mRNA extracted from six LCLs (GM7056, GM12239, GM12762, GM12057, GM12489, and GM12546) pre and post 48h of 100 μM busulfan treatment. The chosen concentration of busulfan represents 80% of cell viability in our set of LCLs with an average 50% inhibitory concentration of 415,1+/-203,3 μM. To avoid having non-genetic cell-to-cell variability in gene expression which could be introduced by the culturing condition, we fixed a limit on the maximum number of cell culture medium passages of 15 times.

Library construction and sequencing were performed at the iGE3 Genomics Platform–CMU, Geneva, Switzerland, using the Illumina HiSeq 2000 library preparation kit. The differential expression analysis was performed with DESeq2 [27,28] following a standard data analysis pipeline (**Supporting Information: Text and Figures;** Text S01 in S1 File). The p-values of the test for differential expression were adjusted for multiple testing using the procedure of Benjamini and Hochberg [32].

### 2.3. Data filtering on WES genotype data

We used the unfiltered whole-exome sequencing data, of which analyses were published previously [22]. Briefly, 87 patients were included, of which 12 developed the main outcome SOS as defined by the modified Seattle criteria [3] **(S1 Tabe).** Prior to conducting the association analysis, we applied a series of filtering steps on genotype data to reduce genetic heterogeneity as previously described [25], using PLINK version 1.9 [29]. (**Supporting Information: Text and Figures;** Text S02 in S1 File**)**. Written informed consent was obtained from every patient or parent/legal guardian by the local research team, who participated in the study and provided genetic material and clinical data. The study was conducted in accordance with the Declaration of Helsinki. The Institutional Review Board of the CHU Sainte-Justine, Montreal, Canada, approved the study and all patients/parents provided an informed consent form (IRB number: 2450, trial registration: Clinicaltrials.gov: NCT01257854).

### 2.4. Tests of association of WES data with clinical outcome

Our clinical outcome SOS was defined using the modified Seattle criteria, as outlined in the description of the clinical dataset [22]. The test for association between the SNPs and the binary outcome, i.e. SOS, was performed with PLINK version 1.9, using both the standard chi-squared test and the logistic regression model implemented in the software. We included the multidimensional scaling components as covariates in the logistic regression analysis, which have been estimated previously to control for population stratification.

We used the Versatile Gene-based Association Study-2 (VEGAS2) software [26] to combine the effects of all SNPs in a single gene into one test statistic (i.e. a chi-squared statistic) and its associated p-value. The tool accounts for linkage disequilibrium (LD) and gene size (number of SNPs per gene) [26].

### 2.5. Combined test of transcriptomic and WES data

We computed a gene-based score, and its associated p-value, by combining the results of the differential gene expression analysis (*Z_exp_*) and the gene-based test for association (*Z_assoc_*), using the weighted Z‐method [30]. We converted the p-values of the 2 tests into z-scores and computed the *Z*_*s*_- combined test statistic, using the following formula:

$$Z_{s}=\frac{Z_{exp}+Z_{assoc}}{\sqrt{2}}∼N(0,1)$$

To assign equal weights to the 2 experiments into the combined statistic, we rescaled the p-values derived from the LCL expression analysis to have the same range as the p-values calculated from the WES data, before converting them to Z-scores. We used Bonferroni correction for multiple testing.

### 2.6. Over-representation analysis

The functional characterization of the genes that show a significant difference in expression in LCLs after treatment with busulfan was performed using an over-representation analysis (ORA). We used the Kyoto Encyclopedia of Genes and Genomes (KEGG) [31] release 97 and the Reactome [32] version 75 databases as a collection of annotated gene sets for ORA. We used the same strategy to characterize the genes that were associated with a significant combined test statistic and outcome, after Bonferroni correction for multiple testing.

### 2.7. Polygenic risk scores

We computed polygenic risk scores (PRSs), for the cases and controls, as the sum of the number of risk alleles carried at each locus (∑*nb_effect allele_*), weighted by their effect size [33]. The latter was estimated, using PLINK version 1.9, as the logarithm of the odds ratio (ln (*OR*)).


$$PRS=\frac{\sum {nb}_{effect\;allele}*ln\;(OR)}{{nb}_{loci}}$$

We used for the estimations of the PRSs the SNPs with the p-value of the association test using the logistic regression model < 0.05, and located within the boundaries of the genes (including the UTRs) for which the combined test was significant after a Bonferroni correction for multiple testing.

## 3. Results

### 3.1. Transcriptomic analysis of lymphoblastoid cell lines

We identified 3093 genes that showed significant differential expression in the LCLs after *in vitro* exposure to busulfan. Among these genes, 1708 genes (55%) were found to be upregulated and 1385 (45%) downregulated in the treated LCLs (**S2 and S3 Tables**).

Among the gene sets that were enriched in up-regulated genes, we found pathways involved in “Cell growth and death” (p53 signaling pathway, Apoptosis, Necroptosis; Regulation by c-FLIP, Caspase activation via Death Receptors in the presence of ligand, RIPK1-mediated regulated necrosis), “immune system” and “immune disease” (Graft-versus-host disease, NOD-like receptor signaling pathway, Allograft rejection; Interferon alpha/ beta/ gamma, Interleukin-2 / -4/ -10/ -13/ -35 signaling), “Signal transduction” (TNF, NF-kappa B, Phosphatidylinositol, FoxO, JAK-STAT) and “Signaling molecules and interaction” (Cell adhesion molecules, Cytokine-cytokine receptor interaction, ECM-receptor interaction; Signaling by KIT in disease, *PECAM1* interactions, RAF-independent *MAPK1/3* activation, Activated *NOTCH1* Transmits Signal to the Nucleus, Signaling by *VEGF/ PDGF*).

Among the down-regulated gene sets, we identified pathways involved in *“Cell growth and death”* (cell cycle, cell cycle checkpoints), “*Replication and Repair*” (DNA replication, DNA repair, base excision repair, mismatch repair, nucleotide excision repair, chromosome maintenance, cellular responses to stress, activation of *ATR* in response to replication stress).

### 3.2. Data filtering on WES genotype data

We used the unfiltered whole-exome sequencing data from our previous publication [19] which contains genotype information for 87 individuals (12 SOS cases and 75 controls). We identified 292,064 bi-allelic SNPs with MAF≥0.05. We filtered out SNPs and individuals in the following series of steps: (1) we excluded 21,138 SNPs with a proportion of missing genotypes exceeding 20%; no individuals had a proportion of missing genotypes larger than 20%; (2) 11 individuals (10 women, 1 man) showed inconsistencies in their assigned and genetic sex and were discarded from the dataset; (3) we excluded 4,898 SNPs with MAF < 0.05 in the remaining sample; (4) we excluded 8,744 markers which deviated from Hardy–Weinberg equilibrium; (5) we discarded 1 individual from Sub-Saharan African ancestry which showed a heterozygosity rate below the chosen threshold; (6) we excluded 4 individuals that showed cryptic relatedness; (7) we removed 14 ethnic outliers, from Asia and Africa. After these filtering steps, our dataset contained genotype information for 57 individuals (11 cases and 46 controls): 1 individual of American origin, 6 individuals from North Africa and 50 individuals of European ancestry) and 252,817 autosomal SNPs.

### 3.3. Association tests in the clinical WES dataset

The analysis at the SNP level using a standard chi-squared test identified 60 variants associated with SOS after Benjamini and Hochberg (1995) step-up false discovery rate (FDR) control [34], and 7 variants after Bonferroni adjustment (**S4 Table**) [35]. Using the logistic regression model, no SNPs reached significance after any of the above adjustments for multiple testing.

At the gene-level, we identified 12 genes that reached significance after Benjamini and Hochberg FDR control *(NACAP1*, *E2F4*, *SHCBP1*, *MIR34C*, *PF4V1*, *CYP7B1*, *TMEM208*, *CXCL6*, *RAB3B*, *LOC101928744*, *LOC101928773*, *CFHR3)*, and 4 genes after Bonferroni correction *(NACAP1*, *E2F4*, *SHCBP1*, *MIR34C)* **(S5 Table).**

### 3.4. Combined test, Over-representation analysis and polygenic risk score

**Fig 3:**
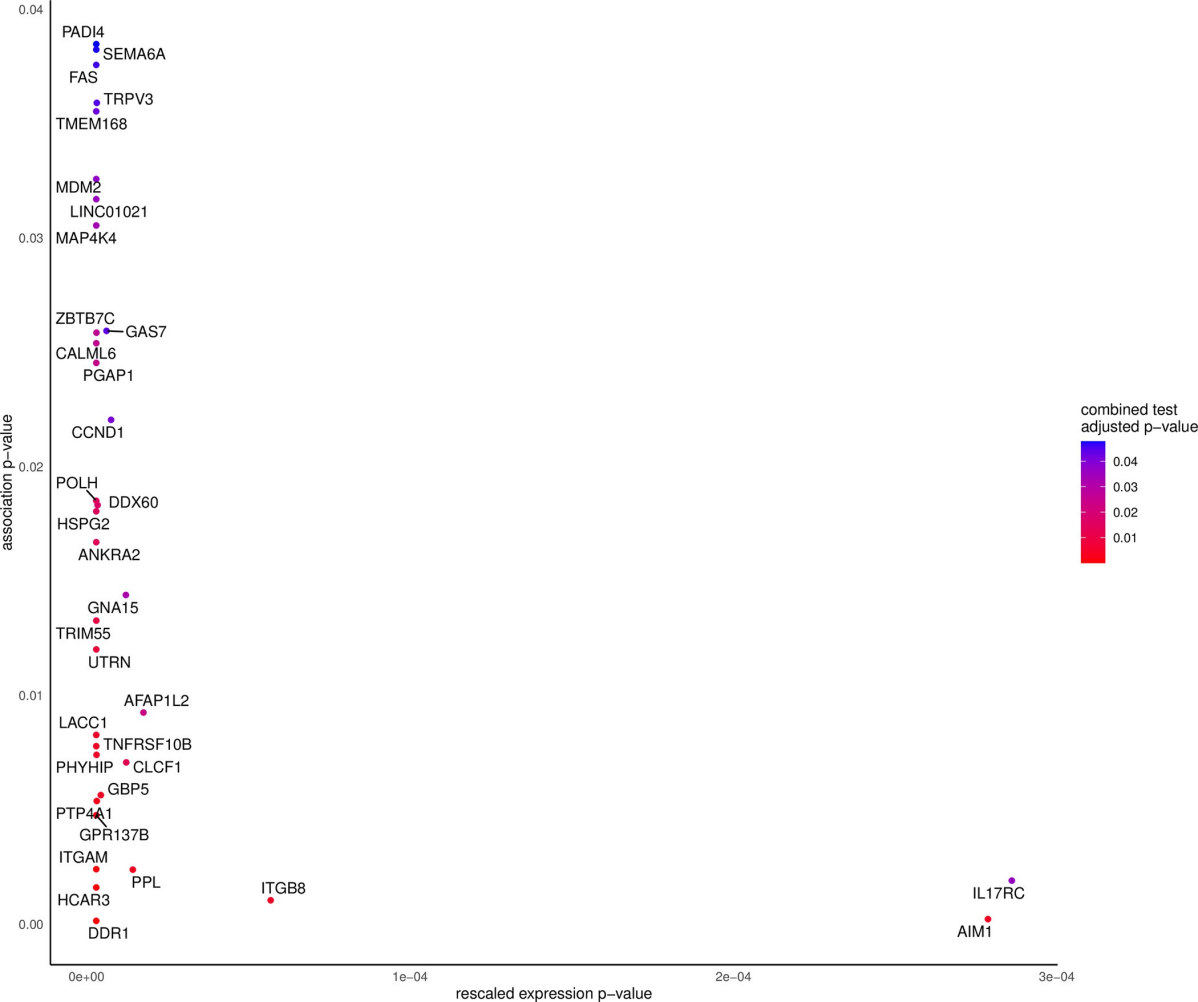
Graphical representation of the relationship between the p-values of the combined test (color coded), the lymphoblastoid cell line expression test (x-axis), and the whole-exome sequencing association test (y axis): We see that the majority of the genes that are associated with a significant combined test statistic show very low p values for the expression test and that the p-value of the combined test increases with the p-value of the association test.

We identified 35 genes that were found to be significant for the test which combined the results of the differential gene expression analysis in LCLs and the gene-based test for an association in the WES dataset after Bonferroni correction for multiple testing **(Fig 3; S6 Table).** There was no overlap between the genes being significant for the combined test and the genes identified from the WES analysis only. But all genes that were significant for the combined test were found to be differentially expressed before and after exposure to busulfan (**Supporting Information: Text and Figures:** Figure S01 and S02 in S1 File).

The functional characterization of these genes using the Reactome database identified 14 significantly enriched pathways **(S7 Table).** These pathways are mostly involved in “apoptosis and regulated necrosis” (dimerization of procaspase-8, regulation by c-FLIP, inhibition of *CASP8* activity, regulation of necroptotic cell death, *TP53-* regulated transcription of death genes, receptors and ligands, ligand-dependent caspase activation, *RIPK1*-mediated regulated necrosis, caspase activation via extrinsic apoptotic signalling pathway). Among the enriched gene sets, we found a pathway implicated in “cell proliferation and differentiation” (i.e. transcriptional regulation by *RUNX3*). “Cell surface proteins” important in cell interaction (integrin cell surface interactions, non-integrin membrane-ECM interactions, extracellular matrix organization).

**Fig 4:**
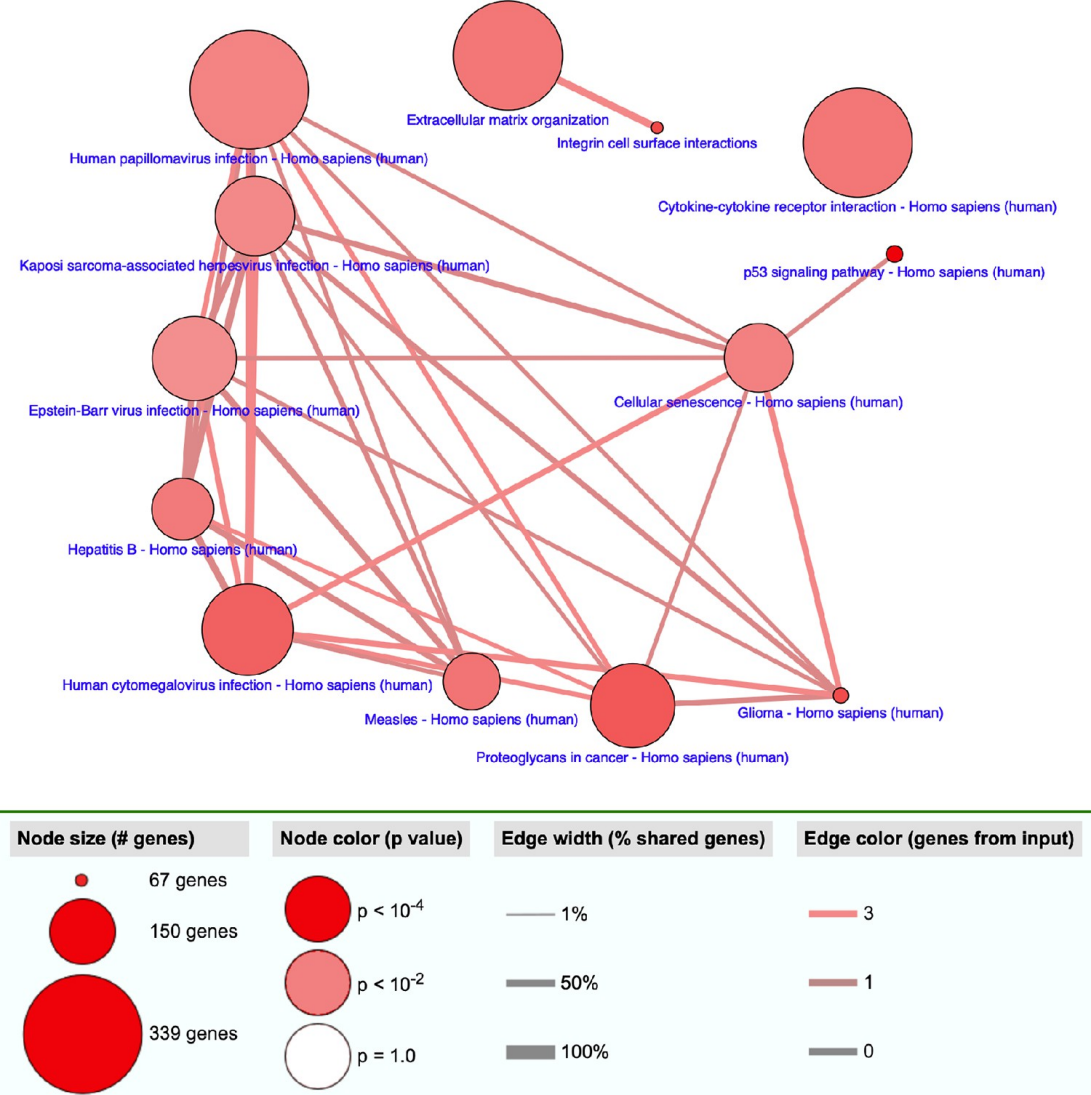
Over representation analysis using CPDB.org incorporating 35 genes associated with sinusoidal obstruction syndrome identified through a combined test statistic.

The KEGG analysis identified 16 enriched pathways. These pathways were involved in “cell growth and death” (p53 signaling pathway, cellular senescence), “signaling molecules and interaction” (cytokine-cytokine receptor interaction), cancer, and infection (**Fig 4**).

The polygenic risk score computed using 209 SNPs from the 35 genes identified through combined expression and association analyses at the gene level showed a significantly different distribution between the cases and the controls (p-value = 8.506e-07; **Supplementary Figure S02 in S1 File**).

## 4. Discussion

In this study, we identified new potential genetic determinants of SOS at the SNP, gene, and pathway levels using a new multi-omics data analysis pipeline integrating data from two different experiments. We analyzed *in vitro* transcriptomics data of LCLs after treatment with busulfan and WES data of predominantly pediatric HSCT patients with and without SOS. We combined the results of these analyses into a test statistic which measures the strength of association to SOS at the gene level. Our pipeline addresses the lack of power of small datasets by prioritizing genes from a differential gene expression analysis in LCLs, and the existence of genetic confounders by using filtering steps to increase genetic homogeneity in WES data. Our pipeline allowed the identification of new potential genetic determinants of SOS that can be validated in a follow-up study using a replication cohort.

In the LCLs treated with busulfan, we found several upregulated pathways, which are mainly involved in the inflammatory response, signaling by interleukins and interferon-gamma, *TP53* signaling, and apoptosis. Interleukins-1 and -2, tumor necrosis factor-alpha, and transforming growth factor-beta were identified previously to be elevated in patients with SOS [36,37] or after busulfan exposure *in vitro* in endothelial cells [38]. The expression change we measured is in agreement with these findings, supporting the assumption that LCLs might be a valid cell model for our study. Among the downregulated pathways, we identified cell cycle and mitochondrial translation. Previous *in vitro* analyses have demonstrated a cell cycle arrest after busulfan exposure which supports our findings [39].

We identified new genetic markers in significant association with SOS, using the WES data, after the application of filtering steps to increase the homogeneity of the genetic dataset. Our data workflow reduced the number of individuals from the unfiltered WES dataset by 34.5% and the number of SNPs by 12.4%. Our pipeline thus generated a more homogenous sample at the cost of a smaller sample size. This workflow allowed us to identify through the analyses at the gene level 12 genes in significant association with SOS. None of these genes was identified in the previously published study, where a different strategy was used with a focus on single SNPs and functional variants [22].

Existing data suggest that the association of the currently identified genes with SOS could be meaningful. *NACAP1* is a pseudogene which was recently associated with pre-eclampsia, a disorder of pregnant women with arterial hypertension, neurological symptoms, and fetal growth restriction [40]. This disease is understood as being mediated by endothelial cell dysfunction [41], which is also a major driver in SOS. *PF4V1* is involved in platelet disorders and clot formation [42,43]; interaction with the coagulation system was long suggested to play a role in SOS [44]. *SHCBP1* is involved in various cell signalling processes, cell proliferation, and upregulated in various neoplasms [45]. *CXCL6* is a chemokine with angiogenic properties and is involved in TNF signaling pathway. The *CYP7B1* gene from the cytochrome P450 gene family is involved in cholesterol and lipid metabolism and is associated with neonatal liver disease [46]. Additional genes identified here are associated with apoptosis (*MIR34C*, *E2F4*) [47,48] and autophagy (*TMEM208*) [49]. Among those genes, only *SHCBP1* was significantly downregulated in LCLs after treatment with busulfan, while *CXCL6*, *NACAP1* and *PF4V1* were not expressed in LCLs.

The combined test statistic allowed to integrate the expression results and the association metrics into a single statistic measure. We identified 35 genes that were found to be associated with SOS after correction for multiple testing. None of these genes was previously investigated for an association with SOS. The functional analysis of these genes, using the KEGG database revealed that many are involved in the *TP53* signaling pathway and cellular senescence (*CCND1*, *FAS*, *MDM2*, *TNFRSF10B*, *CALML6*). This result can be explained by the response of the cells to the toxic effect of busulfan and its metabolites which might also play a role in SOS [50]. In addition, *TP53* regulated genes were significantly upregulated in our gene expression analysis such as *MDM2*, and the pro-apoptotic genes *BBC3* and *GADD45A*. *CALML6* plays an important role in the regulation of the innate immune response through the NF-kB signaling pathway. Previous reports have shown a similar signal of cell cycle gene dysregulation after busulfan exposure [38]. *TNFRSF10B* is a member of the TNF-receptor superfamily, that is activated by TNF-related apoptosis-inducing ligand (*TNFSF10*), and transduces an apoptosis signal [51]. Additionally, the cytokine-cytokine receptor interaction gene set could be explained by inflammatory processes.

The functional analysis using the Reactome database identified pathways mostly involved in programmed cell death. Additionally, gene sets linked to the integrin cell surface interactions were found (*ITGB8*, *HSPG2*, *ITGAM*). Cellular responses to external stimuli are mainly a stress response pathway and regulate cell adaptations when exposed to reactive oxygen species, heat, or other stimuli. Integrins are the receptors that interact with the extracellular matrix and are important for cell adhesion. Two upregulated genes after the treatment with busulfan in this gene set, *ITGAM* and *ITGB8*, are also involved in the complement and coagulation pathway [52], a relevant mechanism involved in SOS [44,53–55], and endothelial cell function [38]. Other genes of interest but not identified in the ORA analysis were *GBP5* which is expressed in endothelial cells and promotes the inflammasome [56], *DDR1* which is associated with endothelial cell senescence [57], *HSPG2* that regulates vascular response to injury and may inhibit thrombosis [58], *LACC1* and *HCAR3* which are proteins from fatty-acid oxidation, lipogenesis, and lipid metabolism, while *POLH* is implicated in DNA damage repair [59].

We estimated polygenic risk scores for the cases and the controls that were included in this study, using the SNPs that were found within the boundaries of the 35 genes that were associated with a significant combined test statistic. The difference in the distributions of the PRSs between the cases and the controls is highly significant. Classically, PRSs are estimated in another cohort than the one that was used to estimate the association metrics with the phenotype of interest. Here, our goal was to estimate the magnitude of differences in scores between our cases and our controls. The difference in scores between cases and controls was highly significant and underscores the notion that the SNPs we used to estimate those scores are good candidates for further validation. In a future study, selecting those SNPs that contributed most to the PRS should be tested or some of the SNPs could be further prioritized, choosing some specific functional categories, i.e. non-synonymous variants.

The pipeline we developed and used in this study allowed us to identify genes and pathways in association with a rare clinical outcome with a limited number of patient samples, where machine learning-based approaches are inapplicable [60]. Classical approaches are based on candidate gene studies that rely on specific genes which are selected based on previous knowledge. Some of these candidate-gene studies have led to relevant findings with an impact on clinical practice (e.g. *TPMT* polymorphism-associated mercaptopurine intolerance in leukemia) [61]. But often, candidate-gene studies suffered from inconsistent replication efforts [62,63] such as was witnessed for SOS [23]. In contrast to a classical candidate gene approach, our method does not rely on the selection of genes a priori. By combining two independent tests, we enable the identification of novel associations that were not previously investigated or hypothesized.

There are some limitations to our approach that are associated with the assumptions that are made in the setup of this study. First, the hypothesis that the genes that are dysregulated in LCLs after busulfan exposure are more likely to carry variants which are modifiers of busulfan toxicity might not be true. The importance of transcriptomic changes after drug exposure was shown previously to correlate with adverse events after drug exposure [64]. Based on the data on busulfan exposure as initiating factor of SOS, we hypothesized that transcriptomic changes after treatment with busulfan play a pivotal role in the development of this complication. The disadvantage is that we focus with this approach on an upstream event and not on the outcome directly. Second, the magnitude of dysregulation might not be linked to the outcome of interest. Third, LCLs have been extensively used in pharmacogenomic research [65], but some genes which might be relevant for SOS are not expressed in LCLs, which limits their applicability to model endothelial or liver cells. This inherent weakness of our model could be addressed in a future study by choosing cell types that reflect more closely the effector cells of the target disease. Conversely, peripheral blood cells such as the used lymphoid cells might harbor the advantage of being more easily be accessible in patients and transcriptomic data could be measured as a potential biomarker of SOS in future studies as previously illustrated [66]. Fourth, to further assess the impact of the identified genes, additional analyses will be needed such as inhibition or overexpression analyses. As this manuscript covers the methodological aspects of combining in vitro data with clinical information and due to the complexity of mechanisms leading to SOS, we will perform these analyses in a future study. Fifth, we did not include clinical confounders (such as patient age, conditioning regimen, underlying disease among others) in our pipeline due to the low number of patients with SOS in our sample. We will include these confounders in a future replication analysis.

In this paper, we proposed a novel approach to unravel the genetic determinants of SOS by combining *in vitro* gene expression information after treatment exposure with clinical genomic data into a multi-omics model. Our approach could be used in other research contexts to increase the power to identify the genetic contribution to other rare diseases.

## Supporting information

S1 Table Clinical data of 87 patients included in this study (adapted from Ansari et al. BBMT, 2020, with permission). (PDF)

S2 Table Differential gene expression analysis in lymphoblastoid cell lines; sorted by expression change. (PDF)

S3 Table Over-representation analysis of differentially expressed genes in lymphoblastoid cell lines, sorted by strength of association. (PDF)

S4 Table List of SNPs that are found to be in significant association with VOD, after correction for multiple testing. The SNPs are sorted by chromosome and position. Functional annotations of the variants were retrieved from ENSEMBL (Genome assembly: GRCh37.p13). The r2 column shows the r2 measure of linkage disequilibrium between each SNP and the following SNP in the table, in the CEU population from the 1,000 Genomes phase 3 data, as reported in ENSEMBL.
(PDF)

S5 Table Whole-exome sequencing association analysis of 57 individuals (11 cases with sinusoidal obstruction syndrome and 46 controls), genes with the strongest association, sorted by adjusted association metric. (PDF)

S6 Table Combined association analysis of differential gene expression in lymphoblastoid cell lines and whole-exome sequencing in 57 individuals; genes sorted by adjusted association metric. (PDF)

S7 Table Over-representation analysis of differentially expressed genes in a combined test statistic integrating differential expression data of lymphoblastoid cell lines after exposure to busulfan and a whole-exome association analysis. (PDF)

S1 File Supporting Information: Text and Figures. **• Text S01.** Description of lymphoblastic cell models, RNA sequencing, and post-processing. **• Text S02.** Description of data filtering steps used in the whole-exome sequencing dataset. **• Figure S01.** Venn diagram showing the number of genes identified in (a) the whole-exome sequencing analysis using the VEGAS2 tool (VEGAS2), (b) the differential gene expression (DGE) from the in vitro analysis before and after treatment of lymphoblastoid cell lines with busulfan, and (c) the combined test aggregating data from both previously mentioned tests into one test statistic. **• Figure S02.** Polygenic risk score including 209 SNPs as output of the data analysis pipeline combining LCL expression and WES clinical association data (MAF > 0.05, p < 0.05), stratified by presence (cases) or absence (controls) of sinusoidal obstruction syndrome.
(DOCX)

## Abbreviations

ALL: Acute lymphoblastic leukemia
CI: Confidence interval
CNS: Central nervous system
DNA: Deoxyribonucleic acid
FDR: False discovery rate
HSCT: Hematopoietic stem cell transplantation
HWE: Hardy–Weinberg equilibrium
LCL: Lymphoblastoid cell line
LD: Linkage disequilibrium
KEGG: Kyoto Encyclopedia of Genes and Genomes
MAF: Minor allele frequency
ORA: Over-representation analysis
PRS: Polygenic risk scores
RNA: Ribonucleic acid
SNP: Single nucleotide polymorphism
SOS: Sinusoidal obstruction syndrome
UTR: Untranslated region
VEGAS2: Versatile Gene-based Association Study-2
WES: Whole-exome sequencing
