# Supplementary material for: A novel integrative multi-omics approach to unravel the genetic determinants of rare diseases with application in sinusoidal obstruction syndrome
Source: PLoS One. 2023 Apr 5;18(4):e0281892. doi: 10.1371/journal.pone.0281892 (PMC10075428; doi:10.1371/journal.pone.0281892)
Supplement: S1 Table — (PDF) [file pone.0281892.s001.pdf]

**Supplementary Table S01. Clinical data of 87 patients included in this study (adapted from Ansari et al. BBMT, 2020, with permission)**

| Clinical Characteristics | Patients n | % |
|---|---|---|
| ***Sex*** | | |
| ***-male*** | 40 | 46 |
| ***-female*** | 47 | 54 |
| ***Ethnicity*** | | |
| ***-caucasian*** | 62 | 71.3 |
| ***-other*** | 25 | 28.7 |
| ***Diagnosis*** | | |
| ***-malignant*** | 45 | 51.7 |
| ***-non-malignant*** | 42 | 48.3 |
| ***HLA compatibilits*** | | |
| ***-unrelated donor*** | 50 | 57.5 |
| ***-related donor*** | 1 | 1.1 |
| ***-HLA-identical sibling*** | 36 | 41.4 |
| ***Stem cell source*** | | |
| ***-bone marros*** | 43 | 49.4 |
| ***-peripheral blood stem cells*** | 2 | 2.3 |
| ***-umbilical cord blood*** | 42 | 48.3 |
| ***Conditioning regimen*** | | |
| ***-Bu/Cy*** | 62 | 71.3 |
| ***-Bu/Cy/VP-16*** | 5 | 5.7 |
| ***-Bu/Flu*** | 19 | 21.8 |
| ***-Bu/Flu/Thiotepa*** | 1 | 1.1 |
| ***Busulfan protocol*** | | |
| ***- one dose per day*** | 67 | 77 |
| ***-four doses per day*** | 20 | 23 |
| ***Chemotherapy regimen*** | | |
| ***-myeloablative*** | 68 | 78.2 |
| ***-myeloablative with reduced toxicity*** | 16 | 18.4 |
| ***-non-myeloablative*** | 3 | 3.4 |
| ***Sinusoidal obstruction syndrome*** | | |
| ***-yes*** | 12 | 13.8 |
| ***-no*** | 75 | 86.2 |
| ***Prophylaxis of SOS*** | | |
| ***-ursodeoxy cholic acid*** | 87 | 100 |
| ***Age in years, median (range)*** | 7.4 | (0.1-23.5) |
| ***cumAUC(mg x h/L), median (range)*** | 59.6 | (25.5-79) |
| ***GSTA1 status**** | 13 (14.9%) | 74 (85.1%) |

***Legend: BM, bone marrow; PBSC, peripheral blood stem cell; Bu, Busulfan; Cy, Cyclophosphamide; VP16, etoposide; Flu, Fludarabine; SOS, Sinusoidal Obstruction Syndrome; cumAUC, cumulative area under the curve; GSTA1, glu- tathione S-transferase A1.***

****, Number and frequency of diplotypes, as derived from genotype***
