# Supplementary material for: A novel integrative multi-omics approach to unravel the genetic determinants of rare diseases with application in sinusoidal obstruction syndrome
Source: PLoS One. 2023 Apr 5;18(4):e0281892. doi: 10.1371/journal.pone.0281892 (PMC10075428; doi:10.1371/journal.pone.0281892)
Supplement: S2 Table — (PDF) [file pone.0281892.s002.pdf]

Supplementary Table S01. Differential gene expression analysis in lymphoblastoid cell lines; sorted by expression change.

Gene	Differential expression (log2 fold change)	p-value	p-value (adjusted)	Base expression (mean)
NT5C1A	-2.077	2.012E-09	7.988E-08	39.018
HIST1H2BO	-1.956	5.982E-08	1.652E-06	22.529
DOC2A	-1.949	8.613E-03	4.400E-02	5.540
HIST1H2BM	-1.920	3.865E-04	3.570E-03	11.478
HIST1H2AL	-1.811	7.523E-04	6.230E-03	11.620
DDN	-1.759	1.340E-14	1.389E-12	78.250
HIST1H3F	-1.731	3.212E-04	3.061E-03	17.070
HIST1H3G	-1.704	1.222E-05	1.867E-04	22.781
HIST1H2BL	-1.663	6.355E-03	3.442E-02	7.398
HIST1H3A	-1.627	4.926E-03	2.802E-02	7.762
HIST1H1D	-1.625	5.816E-05	7.183E-04	23.205
PSRC1	-1.603	9.433E-06	1.501E-04	171.705
EPHB2	-1.590	3.426E-05	4.550E-04	42.906
HIST1H4C	-1.571	4.891E-03	2.786E-02	8.599
HIST1H3C	-1.570	8.307E-05	9.804E-04	19.563
HIST1H1C	-1.562	8.985E-34	5.438E-31	1477.665
HIST1H3J	-1.533	9.276E-03	4.650E-02	7.856
HIST1H2BJ	-1.444	3.833E-15	4.264E-13	130.283
OR2B6	-1.372	3.855E-03	2.312E-02	13.549
PHGDH	-1.370	3.179E-06	5.748E-05	834.898
HIST1H3H	-1.341	3.128E-07	7.208E-06	43.675
HIST1H4I	-1.338	1.439E-03	1.051E-02	17.036
HPDL	-1.326	3.979E-09	1.486E-07	76.014
HIST1H2BF	-1.306	2.496E-03	1.646E-02	17.541
HIST1H2AI	-1.278	3.190E-03	2.003E-02	14.827
SLCO3A1	-1.262	5.242E-03	2.936E-02	19.137
IMPA2	-1.210	2.214E-09	8.699E-08	102.649
HIST1H2BH	-1.208	6.622E-07	1.429E-05	56.376
H1F0	-1.180	1.099E-12	7.919E-11	149.346
TFAP4	-1.177	2.633E-19	4.377E-17	260.503
KIF20A	-1.161	4.563E-06	7.862E-05	1136.859
MCM6	-1.151	4.864E-32	2.453E-29	2327.391
HIST1H2BK	-1.150	1.008E-23	2.825E-21	1814.319
HIST1H1E	-1.145	2.142E-03	1.454E-02	29.135
HIST1H2AE	-1.138	2.382E-05	3.315E-04	38.621
SPTBN2	-1.137	4.655E-03	2.675E-02	16.336
LOC100130872	-1.111	1.201E-07	3.111E-06	133.345
H1FX	-1.096	8.369E-07	1.769E-05	1186.736
IRS1	-1.081	1.342E-08	4.365E-07	94.211
SCD	-1.071	8.667E-07	1.825E-05	13069.391
E2F8	-1.069	7.858E-22	1.723E-19	439.660
PAQR4	-1.062	1.004E-27	3.616E-25	752.499
GNAZ	-1.057	8.310E-04	6.738E-03	55.847
TXNDC5	-1.056	4.575E-03	2.637E-02	19.971

OLMALINC	-1.052	2.585E-12	1.770E-10	273.834
HIST1H2AM	-1.036	3.572E-03	2.183E-02	22.483
SLC6A9	-1.030	3.725E-05	4.897E-04	92.183
ALDH1L2	-1.028	3.574E-14	3.489E-12	322.475
E2F2	-1.019	8.995E-15	9.721E-13	594.038
SREBF1	-1.016	2.492E-07	5.874E-06	2744.851
HIST1H3B	-1.006	7.825E-03	4.080E-02	25.653
SLC19A1	-0.994	4.550E-19	7.171E-17	410.224
CDCA7	-0.981	1.211E-19	2.131E-17	608.560
PSAT1	-0.972	6.543E-04	5.546E-03	859.147
LRRC26	-0.967	2.285E-03	1.532E-02	31.047
LCN10	-0.958	1.182E-04	1.318E-03	42.249
MZB1	-0.952	3.965E-39	2.999E-36	2605.995
CHAC1	-0.950	4.291E-06	7.471E-05	213.290
HIST1H4H	-0.948	3.019E-04	2.908E-03	69.356
UHRF1	-0.944	8.556E-29	3.499E-26	2602.450
HIST1H2BC	-0.943	6.907E-06	1.135E-04	104.843
SNHG19	-0.942	4.249E-12	2.795E-10	281.087
FZD1	-0.927	2.436E-08	7.445E-07	113.521
HMX2	-0.908	4.380E-06	7.611E-05	193.113
FOXD2-AS1	-0.903	5.512E-06	9.245E-05	72.232
FAM72D	-0.900	1.666E-03	1.185E-02	35.450
FJX1	-0.889	1.118E-04	1.260E-03	56.088
FASN	-0.886	1.847E-06	3.574E-05	19400.239
MACROD1	-0.884	2.420E-05	3.356E-04	71.135
FADS1	-0.881	2.440E-18	3.619E-16	3526.090
PIM1	-0.880	9.167E-09	3.117E-07	491.252
HIST4H4	-0.876	5.475E-03	3.042E-02	29.750
CTH	-0.871	6.482E-08	1.773E-06	259.438
ETS2	-0.870	7.965E-08	2.152E-06	149.259
DEPDC1B	-0.869	5.255E-14	4.909E-12	509.958
CDCA3	-0.868	6.425E-10	2.794E-08	974.717
SLC7A5	-0.867	4.536E-15	5.010E-13	2775.849
FADS2	-0.857	1.249E-13	1.099E-11	15226.634
TEAD4	-0.834	1.617E-07	4.050E-06	149.405
BMP4	-0.822	9.902E-04	7.742E-03	102.025
PLK1	-0.822	9.625E-04	7.569E-03	2744.691
UNG	-0.820	1.060E-20	2.109E-18	926.569
HIST1H2BG	-0.817	3.615E-03	2.200E-02	45.020
ASRGL1	-0.815	2.144E-08	6.687E-07	171.839
HMGN5	-0.814	1.217E-09	5.116E-08	372.926
PYCR1	-0.810	4.812E-20	8.772E-18	846.161
CENPV	-0.810	2.014E-10	9.523E-09	226.035
ANKRD37	-0.809	1.253E-08	4.140E-07	194.247
SLC38A5	-0.809	2.823E-14	2.848E-12	610.485
SLCO4C1	-0.806	4.602E-07	1.021E-05	209.185
CDC20	-0.804	4.340E-05	5.568E-04	2992.395
MCM7	-0.802	3.876E-21	8.146E-19	6550.971

CIDEB	-0.801	1.035E-06	2.140E-05	147.568
ANP32E	-0.799	9.827E-19	1.502E-16	1683.623
EIF5AL1	-0.797	2.337E-03	1.558E-02	49.326
ADM5	-0.794	5.039E-06	8.547E-05	122.647
GLIS2	-0.789	6.548E-05	7.977E-04	146.017
EFNA3	-0.787	6.575E-03	3.546E-02	33.703
MIR210HG	-0.786	2.368E-06	4.462E-05	206.376
AURKB	-0.784	6.718E-11	3.579E-09	1825.172
SAPCD2	-0.773	8.240E-14	7.556E-12	852.413
RAB33A	-0.772	6.066E-08	1.671E-06	213.602
HIST2H2BF	-0.769	9.651E-03	4.785E-02	30.161
MKI67	-0.762	4.159E-12	2.761E-10	18938.992
CISD3	-0.761	5.285E-09	1.908E-07	197.737
HIST1H3D	-0.759	1.925E-03	1.335E-02	53.892
NME4	-0.754	5.692E-12	3.619E-10	1013.303
HIST1H2AC	-0.746	2.549E-10	1.186E-08	331.121
LRRC20	-0.743	1.788E-10	8.643E-09	591.841
GINS2	-0.743	7.592E-17	9.903E-15	792.972
METRN	-0.742	3.213E-03	2.011E-02	63.689
SLC43A1	-0.740	7.079E-10	3.060E-08	281.015
HMGB2	-0.740	5.693E-14	5.284E-12	6836.591
PM20D2	-0.738	4.720E-13	3.681E-11	399.583
CDCA7L	-0.738	4.314E-13	3.399E-11	920.354
PTMA	-0.737	4.238E-19	6.821E-17	16741.762
KCTD12	-0.734	4.882E-07	1.077E-05	268.501
MCM4	-0.733	1.092E-20	2.145E-18	4354.833
MCM3	-0.733	3.525E-22	8.081E-20	6103.312
LMNB1	-0.733	1.421E-12	1.005E-10	4157.460
TTC28	-0.730	5.887E-03	3.234E-02	37.710
CTNNAL1	-0.730	6.519E-11	3.485E-09	356.440
ASNS	-0.728	4.276E-13	3.387E-11	891.311
FAM81A	-0.727	7.169E-11	3.766E-09	376.582
E2F1	-0.727	5.077E-17	6.797E-15	1242.977
TMTC4	-0.722	4.310E-08	1.237E-06	177.308
WWOX	-0.719	1.712E-06	3.343E-05	157.261
WDR76	-0.719	1.359E-12	9.651E-11	692.673
LDHA	-0.717	3.422E-12	2.312E-10	13264.812
NFIX	-0.715	6.109E-03	3.334E-02	53.715
DLGAP5	-0.714	3.146E-14	3.150E-12	1217.707
H2AFJ	-0.712	5.515E-11	2.991E-09	399.809
CAMKK1	-0.712	1.278E-03	9.543E-03	62.926
MAZ	-0.712	8.531E-13	6.358E-11	4155.284
CCDC85C	-0.708	2.131E-03	1.448E-02	94.015
LEPR	-0.708	7.237E-03	3.830E-02	48.930
PRR18	-0.708	3.313E-03	2.062E-02	64.470
EIF4EBP1	-0.708	2.827E-10	1.304E-08	516.010
PFKFB4	-0.708	1.048E-06	2.162E-05	597.221
GJC1	-0.705	1.472E-07	3.725E-06	220.816

DUT	-0.705	4.358E-19	6.941E-17	1541.530
IMMP2L	-0.702	1.689E-04	1.785E-03	85.740
BSPRY	-0.700	8.655E-04	6.954E-03	86.618
P2RX5	-0.699	1.278E-06	2.572E-05	263.191
ANKRD9	-0.698	1.266E-03	9.484E-03	60.617
SNORD104	-0.692	4.046E-04	3.703E-03	78.800
NEIL3	-0.690	3.973E-05	5.165E-04	123.732
GPR160	-0.688	1.052E-05	1.651E-04	258.425
PHF19	-0.685	3.539E-09	1.342E-07	2180.626
RRM2	-0.685	9.301E-17	1.193E-14	5561.462
PBK	-0.684	5.127E-10	2.261E-08	344.624
MCM5	-0.683	1.587E-14	1.634E-12	4723.191
HAGHL	-0.683	8.540E-10	3.640E-08	292.967
ZNF367	-0.683	1.095E-08	3.656E-07	329.962
AARS	-0.681	1.997E-12	1.393E-10	5827.654
CPNE7	-0.679	1.338E-05	2.033E-04	221.975
KIF18B	-0.677	2.359E-07	5.611E-06	1552.808
CCNA2	-0.675	6.691E-09	2.376E-07	1222.235
CENPA	-0.672	1.970E-07	4.768E-06	523.058
CENPF	-0.671	2.516E-09	9.737E-08	4091.280
STMN1	-0.664	2.461E-12	1.693E-10	3243.511
KHK	-0.664	8.250E-05	9.744E-04	129.570
VPS9D1-AS1	-0.663	1.349E-08	4.379E-07	415.234
OPN3	-0.660	2.447E-09	9.492E-08	361.697
CENPE	-0.659	4.065E-11	2.287E-09	1169.287
CCNE1	-0.659	1.101E-09	4.641E-08	490.112
RCC1	-0.656	3.415E-16	4.200E-14	2146.912
CDKN3	-0.654	1.350E-07	3.445E-06	481.903
MYBL2	-0.651	1.177E-11	7.183E-10	3857.133
UBE2S	-0.650	2.358E-14	2.411E-12	2877.659
FGFRL1	-0.649	9.203E-04	7.313E-03	94.484
EBP	-0.649	1.291E-10	6.510E-09	1066.040
SNHG3	-0.649	1.964E-03	1.358E-02	1687.228
HMGB1	-0.648	1.122E-13	9.929E-12	7902.729
XBP1	-0.646	4.160E-13	3.313E-11	2991.070
SIGMAR1	-0.645	7.365E-13	5.571E-11	635.686
RANBP1	-0.645	9.440E-13	6.934E-11	2574.961
STC2	-0.643	3.406E-05	4.528E-04	354.287
KLF2	-0.642	4.203E-03	2.476E-02	111.437
SDF2L1	-0.635	1.705E-09	6.863E-08	587.550
EEF2KMT	-0.635	8.849E-08	2.365E-06	286.809
CCNE2	-0.633	7.975E-04	6.533E-03	111.654
MSH6	-0.629	4.312E-12	2.824E-10	2295.159
NT5DC2	-0.628	3.598E-09	1.361E-07	1230.469
WWC1	-0.628	2.167E-03	1.466E-02	82.531
GLYATL1B	-0.628	8.927E-03	4.519E-02	62.194
SLC27A5	-0.626	2.443E-04	2.427E-03	102.174
ETV4	-0.626	1.035E-05	1.629E-04	238.885

GPT2	-0.625	4.451E-05	5.683E-04	462.838
NDUFAF2	-0.624	2.314E-06	4.366E-05	311.216
ENDOG	-0.624	1.349E-06	2.703E-05	180.424
SHMT2	-0.622	1.943E-10	9.217E-09	4599.204
SNRPA1	-0.620	4.150E-14	3.974E-12	1353.503
ANP32B	-0.619	1.316E-13	1.144E-11	4327.041
MIR17HG	-0.619	1.511E-03	1.093E-02	78.181
KIF15	-0.618	5.507E-12	3.516E-10	999.333
FAM64A	-0.617	1.410E-05	2.131E-04	200.954
SRM	-0.615	9.210E-13	6.798E-11	2814.238
MTFP1	-0.615	5.695E-13	4.396E-11	890.510
EPM2A	-0.615	5.028E-06	8.547E-05	177.116
MCM2	-0.613	5.131E-11	2.813E-09	5545.617
ESPL1	-0.611	2.175E-09	8.591E-08	2139.563
H2AFZ	-0.611	7.547E-12	4.738E-10	5304.800
NRM	-0.611	1.873E-06	3.619E-05	714.015
ARHGEF39	-0.610	7.278E-06	1.189E-04	489.156
PKP4	-0.609	4.139E-10	1.858E-08	1012.275
SLC7A11	-0.607	1.641E-04	1.742E-03	311.207
CDC45	-0.604	7.545E-10	3.252E-08	733.307
INSIG1	-0.603	1.086E-12	7.901E-11	5026.538
SLC25A37	-0.603	6.512E-06	1.074E-04	298.289
ELOVL6	-0.602	3.245E-07	7.461E-06	324.780
IFRD2	-0.594	7.847E-12	4.906E-10	1047.433
IL10	-0.593	1.631E-04	1.736E-03	160.907
SLC7A1	-0.592	1.257E-11	7.636E-10	3551.872
C15orf61	-0.591	2.482E-04	2.459E-03	108.470
CCNF	-0.585	1.024E-06	2.120E-05	2021.809
BIRC5	-0.585	1.663E-06	3.256E-05	1618.361
MEX3D	-0.583	8.089E-04	6.608E-03	97.911
KCNQ5	-0.583	7.275E-08	1.980E-06	732.500
RPP25	-0.583	6.056E-04	5.206E-03	100.119
OXCT2	-0.583	3.574E-03	2.183E-02	78.108
POLA1	-0.583	3.703E-11	2.106E-09	1146.907
HNRNPAB	-0.581	3.379E-13	2.748E-11	5106.735
RFX2	-0.581	4.413E-05	5.648E-04	191.709
PPP1R14B	-0.580	9.483E-12	5.856E-10	1947.955
MRTO4	-0.576	3.532E-09	1.342E-07	1117.060
PYGL	-0.575	3.803E-05	4.978E-04	303.720
SSRP1	-0.572	1.016E-11	6.225E-10	6143.294
WDR4	-0.570	1.230E-07	3.170E-06	620.594
NDC1	-0.569	1.514E-07	3.823E-06	713.710
CCDC85B	-0.568	9.158E-12	5.702E-10	1287.364
LRRC45	-0.568	4.572E-08	1.298E-06	631.235
SLC17A9	-0.568	2.175E-12	1.503E-10	1783.845
PHLDB2	-0.566	2.790E-03	1.803E-02	111.092
MCM10	-0.565	7.824E-09	2.721E-07	770.445
SNORA73A	-0.565	1.499E-03	1.087E-02	188.473

UQCC3	-0.565	3.339E-06	5.992E-05	260.141
SPC25	-0.565	4.875E-05	6.167E-04	185.077
THEM6	-0.564	8.496E-09	2.902E-07	519.209
NCL	-0.563	3.963E-09	1.484E-07	23954.199
PLEKHH3	-0.562	2.294E-07	5.470E-06	1195.701
NTHL1	-0.561	5.863E-06	9.769E-05	315.648
XPOT	-0.560	1.517E-09	6.168E-08	1210.308
HMGA1	-0.560	4.403E-11	2.458E-09	15696.229
MAD2L1	-0.559	4.163E-08	1.204E-06	551.375
HIST2H2BE	-0.557	2.267E-04	2.279E-03	136.218
TMEM180	-0.553	3.577E-03	2.183E-02	86.135
KIF14	-0.552	5.018E-06	8.541E-05	628.416
PEG10	-0.551	3.027E-09	1.159E-07	3219.091
TMEM109	-0.551	2.946E-08	8.813E-07	2127.005
SLC1A5	-0.550	2.796E-09	1.077E-07	3504.814
EIF5A	-0.549	1.099E-12	7.919E-11	12232.582
PLCXD1	-0.547	2.762E-05	3.795E-04	1141.597
PAGR1	-0.546	4.541E-09	1.676E-07	1034.467
CKS1B	-0.544	1.132E-06	2.305E-05	965.279
MNX1	-0.544	8.247E-04	6.694E-03	200.656
H2AFX	-0.544	1.234E-08	4.086E-07	2905.463
NUF2	-0.544	2.535E-06	4.741E-05	381.572
MIF	-0.543	1.428E-09	5.854E-08	4897.142
GAS2L3	-0.543	1.313E-03	9.753E-03	115.664
EML2-AS1	-0.543	9.681E-04	7.601E-03	108.378
TMEM147	-0.542	1.324E-08	4.337E-07	545.783
MYC	-0.542	3.662E-10	1.664E-08	3180.719
NEUROG2	-0.542	1.474E-03	1.072E-02	112.663
LONRF1	-0.541	2.268E-08	6.976E-07	393.391
POLE2	-0.538	8.190E-04	6.659E-03	113.349
TRIP13	-0.538	2.101E-08	6.566E-07	421.951
MTHFD1L	-0.537	2.240E-08	6.916E-07	1089.971
PECAM1	-0.537	1.865E-03	1.304E-02	172.320
GTSE1	-0.535	1.224E-05	1.869E-04	1647.823
RIMKLA	-0.535	1.891E-03	1.317E-02	147.845
NANP	-0.534	7.579E-04	6.267E-03	116.606
MANF	-0.534	2.297E-06	4.344E-05	1199.216
PAICS	-0.533	1.626E-07	4.065E-06	3783.833
TTLL12	-0.533	1.693E-10	8.291E-09	2237.905
ECE2	-0.533	3.287E-04	3.124E-03	170.558
FAM104B	-0.531	3.448E-03	2.128E-02	112.877
CD3EAP	-0.531	3.088E-06	5.623E-05	471.686
UBE2T	-0.530	2.670E-07	6.244E-06	528.895
ARHGAP19	-0.530	7.659E-06	1.246E-04	817.262
BUB1B	-0.529	2.975E-08	8.877E-07	1677.011
DDX39A	-0.529	9.617E-11	4.966E-09	2680.722
PRMT1	-0.528	5.166E-12	3.326E-10	5688.551
TMPO	-0.526	9.032E-11	4.696E-09	4178.190

PAFAH1B3	-0.526	2.028E-05	2.892E-04	257.043
HMGB3	-0.526	5.128E-09	1.865E-07	912.002
LYPD6B	-0.526	1.582E-03	1.135E-02	184.692
SNRPD1	-0.525	1.166E-07	3.041E-06	1667.343
ILF3-AS1	-0.525	1.500E-05	2.241E-04	202.318
LSR	-0.525	3.878E-08	1.130E-06	793.290
DMKN	-0.522	2.857E-04	2.771E-03	184.242
TBC1D30	-0.522	1.648E-03	1.175E-02	220.195
FAM111B	-0.522	1.137E-05	1.774E-04	713.622
GARS	-0.522	9.513E-10	4.020E-08	3470.241
TECR	-0.521	4.412E-09	1.632E-07	1323.236
SLBP	-0.519	1.962E-09	7.810E-08	1538.680
N4BP3	-0.519	1.451E-05	2.173E-04	714.989
NAA38	-0.517	1.108E-08	3.693E-07	742.524
FBXO5	-0.515	2.133E-06	4.073E-05	444.839
LOC100506844	-0.514	9.128E-03	4.593E-02	75.545
CCR10	-0.514	3.514E-04	3.286E-03	191.082
CENPM	-0.513	4.829E-07	1.068E-05	902.096
TUBA1B	-0.513	6.250E-07	1.354E-05	22437.863
ZBTB32	-0.513	1.497E-09	6.104E-08	1790.956
DTYMK	-0.512	9.862E-09	3.346E-07	990.397
SAMD1	-0.512	6.893E-09	2.443E-07	835.405
KIF11	-0.512	1.341E-08	4.365E-07	1776.226
EXOSC8	-0.512	3.793E-07	8.566E-06	660.473
LOC63930	-0.512	9.187E-03	4.617E-02	116.641
PKN3	-0.510	1.426E-05	2.150E-04	564.105
HJURP	-0.509	1.691E-06	3.305E-05	1182.022
KIFC1	-0.508	3.721E-06	6.615E-05	2846.517
HK2	-0.508	3.385E-08	1.004E-06	2128.943
KIF23	-0.508	1.096E-07	2.879E-06	1211.630
CD320	-0.508	3.279E-09	1.250E-07	574.508
FKBP11	-0.506	2.821E-06	5.199E-05	529.437
PPRC1	-0.506	4.409E-09	1.632E-07	2471.130
B4GALT2	-0.505	4.179E-07	9.396E-06	529.509
HIST2H2BC	-0.504	1.011E-02	4.961E-02	71.500
CAMSAP3	-0.503	5.980E-05	7.355E-04	353.392
DEPDC1	-0.502	3.732E-05	4.901E-04	296.338
EXO1	-0.502	4.581E-07	1.018E-05	609.278
ANLN	-0.502	4.148E-08	1.202E-06	751.812
LONP1	-0.501	5.496E-08	1.537E-06	2789.566
NTMT1	-0.501	1.301E-07	3.331E-06	514.070
PFAS	-0.500	4.269E-09	1.587E-07	2649.561
INCENP	-0.500	1.591E-05	2.355E-04	2715.073
FABP5	-0.500	6.371E-06	1.055E-04	1254.213
NCAPG	-0.500	7.514E-09	2.625E-07	1419.920
TCL1A	-0.500	9.862E-07	2.050E-05	2282.020
CHEK1	-0.500	1.287E-09	5.331E-08	815.784
KCND2	-0.498	1.241E-03	9.327E-03	205.694

TRIB3	-0.497	1.750E-05	2.546E-04	657.821
MGST1	-0.497	1.767E-04	1.854E-03	388.527
MRPS28	-0.497	4.634E-05	5.887E-04	458.809
FGFR4	-0.497	8.639E-03	4.410E-02	127.395
SLC29A2	-0.495	1.557E-05	2.311E-04	383.210
ITPRIPL1	-0.494	1.135E-04	1.277E-03	373.911
YWHAH	-0.494	2.007E-08	6.312E-07	1741.031
CLSPN	-0.493	5.169E-09	1.875E-07	1298.083
TK1	-0.492	1.719E-08	5.496E-07	1740.978
HNRNPD	-0.490	2.186E-08	6.762E-07	6561.205
C20orf27	-0.489	4.436E-08	1.266E-06	742.800
ZFAT	-0.489	1.937E-08	6.143E-07	1758.279
FHIT	-0.489	2.196E-03	1.483E-02	133.388
GMNN	-0.488	3.812E-06	6.754E-05	524.438
NCAPH	-0.488	1.002E-06	2.080E-05	1920.770
KIF22	-0.487	5.482E-06	9.206E-05	2261.038
VARS	-0.487	7.771E-07	1.656E-05	3356.618
STAG1	-0.486	1.193E-05	1.834E-04	885.362
SLC25A10	-0.486	2.606E-06	4.855E-05	601.447
CDC6	-0.485	2.133E-06	4.073E-05	916.845
NASP	-0.484	4.160E-12	2.761E-10	4817.842
ARHGAP11A	-0.484	2.073E-07	4.995E-06	994.673
WDYHV1	-0.482	2.278E-04	2.288E-03	211.531
THOC6	-0.481	3.171E-06	5.745E-05	474.941
DHFR	-0.480	9.665E-12	5.944E-10	2195.950
MVK	-0.479	8.192E-08	2.205E-06	1392.955
CCDC78	-0.479	4.227E-03	2.488E-02	139.379
SUSD3	-0.479	4.195E-05	5.410E-04	475.419
ORC1	-0.478	1.835E-06	3.556E-05	1106.741
ALDOC	-0.478	4.577E-05	5.834E-04	2206.474
MRPS34	-0.478	3.800E-09	1.427E-07	1343.080
SNRPG	-0.477	5.680E-05	7.039E-04	969.603
WHSC1	-0.476	2.115E-06	4.051E-05	5982.877
IRF4	-0.475	2.404E-10	1.126E-08	25003.476
FEN1	-0.473	4.217E-08	1.215E-06	3293.217
PIGW	-0.472	8.433E-04	6.812E-03	184.530
SKA1	-0.472	3.916E-06	6.921E-05	466.524
ARL2	-0.472	1.055E-05	1.654E-04	718.654
F13A1	-0.471	9.866E-05	1.133E-03	756.474
TRIM65	-0.471	2.514E-07	5.916E-06	603.437
BRIX1	-0.471	1.592E-04	1.700E-03	474.370
PRPF40B	-0.471	2.388E-05	3.320E-04	418.008
ARL6IP6	-0.469	1.641E-05	2.416E-04	356.579
SLC43A3	-0.469	3.987E-13	3.208E-11	3678.822
PYCRL	-0.469	3.002E-06	5.491E-05	386.940
RRS1	-0.469	1.258E-06	2.537E-05	718.300
IMPDH1	-0.468	5.486E-08	1.537E-06	1014.809
DNPH1	-0.468	2.368E-08	7.266E-07	757.956

NCKIPSD	-0.468	2.991E-07	6.920E-06	801.838
SRSF7	-0.467	2.435E-08	7.445E-07	3023.699
POLD2	-0.466	2.743E-10	1.269E-08	2412.509
CNTNAP1	-0.466	8.260E-07	1.750E-05	1830.516
CDK2AP2	-0.465	6.114E-06	1.017E-04	1783.885
CKAP4	-0.463	7.823E-06	1.270E-04	794.503
SNRPF	-0.462	4.427E-07	9.893E-06	1012.614
GFI1	-0.462	7.918E-06	1.281E-04	515.604
ABHD11	-0.461	1.217E-04	1.355E-03	213.310
HDAC7	-0.461	5.336E-06	8.971E-05	712.618
RFC4	-0.461	4.694E-07	1.040E-05	704.886
PCK2	-0.459	2.734E-06	5.062E-05	2373.578
TOMM5	-0.458	5.060E-05	6.379E-04	889.927
ALYREF	-0.458	1.110E-07	2.912E-06	1762.787
TOMM40	-0.457	1.158E-06	2.351E-05	2295.002
ZNF511	-0.457	2.553E-06	4.763E-05	427.188
ATF4	-0.456	1.336E-08	4.365E-07	7630.804
FOXM1	-0.456	1.161E-04	1.300E-03	2575.504
ODC1	-0.455	2.314E-09	9.001E-08	4795.620
LBR	-0.454	1.372E-07	3.495E-06	2203.679
LRR1	-0.454	8.843E-05	1.029E-03	333.984
TEX30	-0.453	1.271E-03	9.509E-03	166.283
PUS1	-0.453	1.031E-08	3.481E-07	983.859
STRA13	-0.452	4.408E-06	7.640E-05	466.919
HAUS7	-0.451	1.004E-05	1.588E-04	416.668
IGLL5	-0.450	1.296E-06	2.601E-05	9430.800
PIF1	-0.450	4.685E-05	5.942E-04	1340.047
IL17RB	-0.450	1.237E-04	1.374E-03	882.553
PKMYT1	-0.449	2.507E-06	4.701E-05	1278.888
DHODH	-0.449	5.416E-05	6.761E-04	330.723
SDHAF3	-0.448	1.322E-03	9.808E-03	169.170
SEPHS1	-0.448	2.590E-07	6.075E-06	1088.466
DAZAP1	-0.448	1.349E-09	5.561E-08	3675.431
GNB1L	-0.448	1.604E-04	1.709E-03	239.722
GADD45GIP1	-0.448	7.800E-07	1.660E-05	776.594
CHRNA5	-0.448	9.931E-03	4.885E-02	108.155
HADH	-0.448	5.596E-08	1.559E-06	1292.703
ISG20	-0.448	1.674E-07	4.151E-06	1834.723
HGH1	-0.447	1.458E-07	3.694E-06	1046.644
GTPBP6	-0.446	5.192E-09	1.879E-07	1584.933
DENND6A	-0.446	8.008E-05	9.488E-04	252.031
CHCHD4	-0.445	1.228E-03	9.244E-03	241.095
CEP85	-0.445	2.862E-06	5.262E-05	1105.431
RNF126	-0.445	4.833E-09	1.770E-07	1343.034
YEATS4	-0.445	4.279E-04	3.888E-03	229.063
CDCA2	-0.445	2.943E-06	5.391E-05	991.495
CDCA8	-0.444	3.230E-05	4.351E-04	2086.137
DTD1	-0.443	5.077E-06	8.602E-05	729.811

TRIM28	-0.443	8.409E-09	2.879E-07	11057.584
HMGCS1	-0.443	1.149E-05	1.789E-04	2595.473
HNRNPA2B1	-0.442	1.678E-10	8.258E-09	23657.695
SNRPE	-0.442	6.900E-06	1.135E-04	784.524
CDKN2C	-0.442	1.392E-03	1.025E-02	756.650
PAXIP1	-0.441	2.441E-05	3.382E-04	841.988
LDLR	-0.441	6.057E-05	7.444E-04	3152.812
SKA3	-0.441	1.176E-05	1.816E-04	423.893
NT5M	-0.439	9.446E-03	4.708E-02	103.151
CKS2	-0.439	2.300E-06	4.345E-05	1450.731
CHAF1B	-0.439	1.157E-05	1.797E-04	707.926
CDC25A	-0.438	8.885E-06	1.421E-04	817.628
NR2F6	-0.438	5.950E-04	5.135E-03	230.344
MARCH9	-0.438	3.858E-05	5.032E-04	700.137
PTMS	-0.437	2.417E-04	2.411E-03	1170.458
CEP41	-0.436	1.825E-04	1.901E-03	261.998
TCF3	-0.436	1.864E-07	4.550E-06	7044.812
CHDH	-0.436	1.455E-06	2.882E-05	1141.989
BUB1	-0.436	1.296E-05	1.973E-04	2735.702
FKBP4	-0.436	3.447E-08	1.021E-06	2500.703
CCNB1	-0.436	8.487E-05	9.954E-04	2096.241
MCMBP	-0.435	2.682E-07	6.253E-06	1340.773
ENO1	-0.434	1.257E-08	4.143E-07	37699.353
SLC27A4	-0.434	1.258E-05	1.917E-04	742.294
GNL3	-0.433	1.919E-04	1.981E-03	2974.841
BMP8B	-0.433	6.954E-04	5.842E-03	343.533
COQ2	-0.433	7.099E-04	5.947E-03	200.809
KNOP1	-0.432	3.024E-05	4.114E-04	447.284
SLC39A4	-0.431	5.733E-03	3.159E-02	140.010
GPRIN1	-0.431	8.417E-05	9.887E-04	370.843
C10orf2	-0.430	3.243E-06	5.835E-05	797.747
LYAR	-0.430	1.058E-03	8.194E-03	645.191
GALK1	-0.430	8.223E-05	9.719E-04	359.162
DKC1	-0.430	1.887E-05	2.717E-04	2462.420
LPCAT1	-0.430	2.610E-08	7.912E-07	4881.073
HMGN2	-0.429	7.297E-08	1.982E-06	10247.760
SMC1A	-0.429	4.902E-06	8.389E-05	8515.861
PRC1	-0.429	7.074E-05	8.502E-04	2699.066
TNFRSF19	-0.428	4.398E-03	2.564E-02	274.255
DIAPH3	-0.428	3.312E-04	3.145E-03	556.799
FUNDC2	-0.428	3.831E-06	6.780E-05	876.898
TPX2	-0.428	8.325E-05	9.810E-04	3776.873
RAN	-0.427	2.227E-07	5.324E-06	11389.260
CLN6	-0.427	5.022E-07	1.103E-05	1293.942
RNF145	-0.427	3.932E-06	6.934E-05	1147.390
ATAD3A	-0.427	4.200E-08	1.213E-06	1390.470
FBXO43	-0.427	9.067E-03	4.570E-02	124.341
TOX2	-0.425	9.633E-04	7.571E-03	532.822

MRPL12	-0.425	4.983E-06	8.500E-05	1910.489
NXT1	-0.425	5.434E-05	6.778E-04	385.204
RASSF1	-0.424	2.010E-05	2.868E-04	594.569
PPP1R3E	-0.424	1.796E-05	2.605E-04	426.038
GCSH	-0.423	6.281E-03	3.410E-02	194.193
C9orf142	-0.423	2.814E-04	2.737E-03	334.797
TRA2B	-0.423	3.302E-08	9.814E-07	3622.591
GEMIN4	-0.423	1.657E-05	2.431E-04	1565.485
NDUFAF4	-0.423	2.322E-03	1.552E-02	274.516
REEP4	-0.422	2.159E-04	2.193E-03	812.669
CDKN2D	-0.422	3.922E-03	2.344E-02	216.541
ABHD17C	-0.421	3.999E-03	2.385E-02	150.548
LMNB2	-0.421	4.797E-08	1.359E-06	7880.663
CCBL1	-0.421	7.071E-05	8.502E-04	312.683
GMEB1	-0.420	3.142E-04	3.011E-03	314.247
ALKBH2	-0.420	5.974E-04	5.150E-03	211.303
TMEM97	-0.420	3.605E-06	6.425E-05	933.204
FDPS	-0.420	7.366E-09	2.591E-07	8348.475
UCHL1	-0.420	2.858E-05	3.910E-04	900.024
CCT5	-0.420	7.147E-06	1.172E-04	5648.756
USP1	-0.419	4.900E-07	1.079E-05	915.553
MYADM	-0.419	2.543E-03	1.671E-02	266.904
SLC48A1	-0.419	2.893E-03	1.849E-02	205.265
MPP6	-0.418	1.044E-05	1.640E-04	533.321
DNASE2	-0.418	6.070E-05	7.448E-04	749.759
HSPBP1	-0.418	6.289E-06	1.042E-04	686.748
CDK4	-0.418	7.381E-09	2.591E-07	2759.585
TPI1	-0.417	4.790E-09	1.759E-07	15102.010
NDC80	-0.417	4.206E-05	5.421E-04	720.281
TSEN15	-0.417	4.817E-04	4.284E-03	401.351
TAF6L	-0.417	1.705E-05	2.497E-04	495.828
HSPD1	-0.416	4.123E-05	5.332E-04	12911.801
HMBS	-0.416	2.050E-06	3.932E-05	735.443
CEP55	-0.416	3.930E-06	6.934E-05	897.603
ANAPC15	-0.416	5.233E-05	6.571E-04	609.530
BNIP3	-0.416	8.438E-06	1.358E-04	745.525
PCBP1	-0.415	6.022E-09	2.159E-07	9656.919
CCDC183-AS1	-0.415	2.906E-03	1.856E-02	183.135
RPL17	-0.415	2.858E-03	1.833E-02	168.200
CDC7	-0.415	2.096E-04	2.140E-03	394.451
SIVA1	-0.414	1.495E-06	2.956E-05	970.933
FXN	-0.414	2.769E-03	1.793E-02	237.869
SIT1	-0.413	4.647E-03	2.673E-02	559.338
WDR62	-0.413	3.255E-06	5.850E-05	1409.101
LIG3	-0.413	2.675E-05	3.689E-04	905.779
MAPKAPK3	-0.412	1.111E-04	1.253E-03	480.939
DHCR7	-0.412	1.121E-06	2.285E-05	3473.047
YARS	-0.412	1.750E-07	4.306E-06	4108.254

NUDCD2	-0.412	3.019E-04	2.908E-03	379.463
BANF1	-0.411	2.462E-07	5.821E-06	2348.494
CCDC86	-0.411	5.088E-05	6.405E-04	1386.049
ICT1	-0.411	1.248E-04	1.385E-03	415.401
FBL	-0.411	4.561E-08	1.297E-06	3957.096
FARS2	-0.411	4.486E-04	4.038E-03	334.026
DNAAF5	-0.409	1.917E-05	2.752E-04	966.878
HIRIP3	-0.409	1.163E-04	1.301E-03	811.579
LIPE	-0.409	2.466E-03	1.631E-02	284.313
AVEN	-0.408	2.788E-03	1.803E-02	225.392
MYB	-0.408	1.228E-03	9.244E-03	293.954
GAR1	-0.406	7.335E-04	6.094E-03	250.905
SNRPA	-0.406	1.230E-07	3.170E-06	2629.242
ECI1	-0.406	2.033E-05	2.896E-04	669.417
MARS	-0.405	2.455E-07	5.813E-06	4650.806
PGM1	-0.405	1.404E-06	2.799E-05	1630.041
MTHFD1	-0.405	1.942E-06	3.743E-05	4833.397
SAP30	-0.405	9.072E-05	1.055E-03	349.768
SKP2	-0.403	1.146E-06	2.330E-05	796.965
SRSF2	-0.402	1.838E-08	5.842E-07	7002.829
PINX1	-0.402	4.008E-04	3.673E-03	385.572
GPI	-0.402	4.497E-07	1.001E-05	11338.889
RBBP8	-0.401	4.164E-04	3.793E-03	1016.478
HCFC1	-0.401	9.633E-06	1.528E-04	7813.486
RACGAP1	-0.401	4.591E-05	5.842E-04	1801.265
NOP56	-0.400	6.387E-05	7.799E-04	4584.493
WNT10A	-0.400	6.656E-04	5.626E-03	932.778
RFC2	-0.400	4.367E-08	1.249E-06	1203.180
ASPH	-0.400	1.824E-03	1.280E-02	269.970
PTGES3	-0.399	8.842E-07	1.851E-05	3512.463
GALNT18	-0.399	3.840E-03	2.307E-02	204.052
C1QBP	-0.398	6.069E-05	7.448E-04	3409.650
CDCA4	-0.398	8.187E-08	2.205E-06	1453.345
POP7	-0.398	1.845E-05	2.664E-04	601.812
FAM173A	-0.398	1.821E-04	1.899E-03	396.968
SNHG17	-0.397	4.320E-04	3.921E-03	371.159
MXD3	-0.396	5.666E-04	4.935E-03	586.666
COA7	-0.396	1.160E-04	1.299E-03	363.947
FARSB	-0.396	1.093E-03	8.425E-03	1184.468
CECR5	-0.395	2.150E-06	4.097E-05	762.012
HIST1H2BD	-0.395	1.819E-03	1.277E-02	246.729
GTF3A	-0.394	1.954E-05	2.799E-04	1660.670
GOLM1	-0.394	1.594E-04	1.701E-03	483.607
RAD54L	-0.394	1.547E-05	2.300E-04	1194.725
KNSTRN	-0.393	6.504E-06	1.074E-04	685.623
SH3PXD2A	-0.393	2.915E-04	2.815E-03	1543.218
DIS3L	-0.393	4.500E-05	5.740E-04	725.480
CAD	-0.393	1.989E-05	2.844E-04	5865.875

ALDH1B1	-0.393	3.233E-06	5.823E-05	983.703
CACYBP	-0.393	8.846E-05	1.029E-03	1247.938
NOLC1	-0.393	4.891E-05	6.182E-04	4971.646
NT5C	-0.392	1.066E-05	1.669E-04	626.472
NOP16	-0.392	7.115E-04	5.954E-03	943.297
PDCD2L	-0.392	2.861E-03	1.833E-02	202.671
NPM3	-0.392	3.226E-06	5.818E-05	797.417
MMAB	-0.391	4.507E-06	7.785E-05	770.511
TMPO-AS1	-0.391	2.820E-03	1.815E-02	372.424
HNRNPA0	-0.390	2.681E-07	6.253E-06	5458.138
TOE1	-0.390	7.644E-05	9.113E-04	522.430
SCFD2	-0.390	9.145E-05	1.061E-03	840.180
H2AFV	-0.390	9.260E-07	1.932E-05	3056.040
SRSF3	-0.389	1.585E-07	3.976E-06	4446.434
SUV39H1	-0.389	7.167E-05	8.606E-04	767.464
SNRPB	-0.389	2.947E-08	8.813E-07	4976.919
EXOSC9	-0.388	2.283E-05	3.199E-04	906.071
IKZF2	-0.388	1.161E-05	1.798E-04	3064.879
SLIRP	-0.387	1.298E-03	9.669E-03	533.264
FDXACB1	-0.387	5.164E-03	2.905E-02	178.612
NARS2	-0.387	1.557E-04	1.674E-03	502.441
SIPA1	-0.387	5.136E-04	4.518E-03	2945.173
KLHL29	-0.387	3.280E-03	2.044E-02	528.442
RPIA	-0.386	1.748E-05	2.546E-04	886.074
SLC3A2	-0.386	6.171E-06	1.025E-04	2361.521
PPP1R26	-0.386	4.414E-04	3.980E-03	666.681
SLC26A6	-0.386	3.850E-04	3.560E-03	631.619
TIPIN	-0.386	1.064E-03	8.234E-03	274.744
MRPS26	-0.386	5.600E-06	9.366E-05	807.068
TAF5	-0.385	3.317E-03	2.064E-02	234.240
PSMB8-AS1	-0.385	2.946E-05	4.022E-04	809.943
CTCF	-0.385	4.450E-05	5.683E-04	1970.107
NOP58	-0.385	4.617E-04	4.131E-03	2105.324
C6orf223	-0.384	9.160E-07	1.914E-05	1962.151
SMC4	-0.384	2.464E-08	7.516E-07	3280.953
LSM4	-0.384	5.330E-06	8.971E-05	2545.989
ACTL6A	-0.384	2.215E-05	3.118E-04	867.122
ATP8B2	-0.383	1.539E-04	1.658E-03	1498.300
MRPS2	-0.383	2.891E-07	6.720E-06	1419.026
MCAM	-0.383	1.783E-03	1.254E-02	652.500
ANP32A	-0.383	1.057E-07	2.791E-06	4373.238
LRP8	-0.383	3.495E-06	6.243E-05	1370.844
ECT2	-0.383	9.638E-05	1.110E-03	666.146
PTBP1	-0.383	9.513E-08	2.534E-06	10059.245
MRM1	-0.382	4.187E-05	5.405E-04	434.376
CEBPG	-0.382	1.176E-05	1.816E-04	668.851
RPA3	-0.382	1.347E-03	9.972E-03	658.210
TUBE1	-0.381	8.552E-03	4.377E-02	137.533

UBE2J1	-0.381	3.591E-05	4.727E-04	3901.152
NRF1	-0.381	1.590E-04	1.699E-03	475.997
PPA2	-0.381	1.399E-04	1.528E-03	763.964
MAP1B	-0.380	7.938E-04	6.506E-03	1396.427
ACSL3	-0.380	4.072E-06	7.131E-05	1336.143
EXOSC5	-0.380	3.257E-05	4.381E-04	524.503
TFDP1	-0.380	1.162E-07	3.037E-06	4263.135
SET	-0.379	9.348E-06	1.489E-04	9101.451
LTB	-0.379	1.686E-03	1.196E-02	888.829
RBM14	-0.379	1.654E-05	2.429E-04	2184.778
RABAC1	-0.379	4.013E-04	3.676E-03	637.152
PCCA	-0.378	1.174E-03	8.923E-03	299.295
PRPS2	-0.378	6.948E-06	1.140E-04	1083.849
SRSF1	-0.378	1.254E-06	2.533E-05	6850.660
CMSS1	-0.376	3.336E-04	3.157E-03	513.806
PUS7	-0.376	1.327E-03	9.841E-03	456.280
MBD3	-0.376	5.031E-08	1.423E-06	3111.898
DDX11	-0.375	3.401E-05	4.526E-04	1537.184
NUP35	-0.374	4.175E-03	2.466E-02	230.522
EBPL	-0.374	6.110E-03	3.334E-02	181.528
MAGOHB	-0.374	1.125E-03	8.617E-03	321.966
UBE2C	-0.374	1.221E-03	9.222E-03	1647.735
MRPL22	-0.374	1.294E-03	9.649E-03	522.870
SMYD5	-0.374	5.213E-05	6.550E-04	662.738
MANEAL	-0.374	1.396E-03	1.028E-02	261.414
SUCLG2	-0.374	1.009E-05	1.593E-04	1030.759
TRAP1	-0.373	1.557E-06	3.060E-05	3879.761
DBF4	-0.372	3.164E-04	3.025E-03	597.221
NLN	-0.372	5.558E-04	4.855E-03	650.506
YBX1	-0.372	1.697E-07	4.181E-06	19433.571
C12orf65	-0.372	2.040E-04	2.085E-03	371.054
DDX10	-0.372	5.597E-05	6.941E-04	849.665
ATAD3B	-0.372	9.567E-05	1.105E-03	1037.880
EIF4A3	-0.371	2.539E-06	4.742E-05	2018.619
NCAPG2	-0.371	1.994E-05	2.849E-04	1587.112
NPM1	-0.371	1.151E-04	1.293E-03	17433.096
EXOSC2	-0.369	6.693E-05	8.107E-04	849.160
MRPL23	-0.369	2.945E-03	1.874E-02	211.489
PPP3CB	-0.369	2.565E-04	2.535E-03	817.206
WDR18	-0.369	2.100E-05	2.975E-04	941.457
C4orf46	-0.369	6.725E-04	5.678E-03	406.583
CABLES1	-0.369	5.789E-04	5.019E-03	1198.269
USP7	-0.368	9.574E-06	1.520E-04	4383.703
PRDX2	-0.368	1.199E-05	1.839E-04	1437.633
TCOF1	-0.368	2.997E-05	4.081E-04	3607.286
RANGAP1	-0.368	1.747E-06	3.402E-05	4634.999
PPAT	-0.368	1.382E-04	1.512E-03	754.122
MRPL14	-0.368	1.545E-05	2.298E-04	994.455

HNRNPR	-0.367	4.171E-07	9.390E-06	4964.757
IFT122	-0.367	2.379E-03	1.581E-02	411.569
RPF2	-0.367	6.536E-03	3.531E-02	562.975
ESAM	-0.366	1.244E-05	1.897E-04	901.900
PGK1	-0.366	1.544E-06	3.046E-05	11641.371
MRPS17	-0.366	5.467E-03	3.039E-02	277.579
PLK4	-0.366	5.277E-05	6.620E-04	651.757
ZFAND2B	-0.366	3.412E-04	3.215E-03	360.213
SSNA1	-0.366	2.165E-06	4.121E-05	1322.214
SSSCA1	-0.366	1.501E-04	1.624E-03	425.938
NHP2	-0.365	1.426E-06	2.832E-05	1634.610
DCTPP1	-0.364	8.697E-05	1.016E-03	1038.461
NCAPD3	-0.364	2.322E-04	2.327E-03	2949.614
PPIA	-0.364	5.091E-06	8.616E-05	29044.101
HSP90AB1	-0.364	1.115E-04	1.257E-03	47090.232
MFSD3	-0.364	6.080E-04	5.219E-03	359.302
FUT11	-0.364	7.867E-03	4.096E-02	332.744
SUB1	-0.363	1.009E-03	7.876E-03	3096.206
USP13	-0.363	1.022E-04	1.165E-03	740.376
FAM207A	-0.363	3.896E-05	5.073E-04	566.901
CCNB2	-0.363	2.922E-03	1.864E-02	1434.887
CHAF1A	-0.363	1.739E-05	2.540E-04	1963.829
POLR3H	-0.362	7.748E-05	9.216E-04	1044.255
SMC3	-0.362	2.890E-06	5.301E-05	2161.747
ZWINT	-0.362	2.777E-07	6.464E-06	2317.446
GEMIN2	-0.361	4.166E-03	2.461E-02	287.550
C6orf136	-0.361	5.979E-04	5.152E-03	394.247
PLA2G4A	-0.360	4.655E-03	2.675E-02	311.874
ANKLE1	-0.360	9.063E-04	7.226E-03	577.019
NAGLU	-0.359	1.152E-04	1.293E-03	969.402
IGF2BP1	-0.359	2.784E-04	2.714E-03	619.892
SAAL1	-0.359	4.620E-04	4.131E-03	381.026
WDR54	-0.359	2.546E-03	1.673E-02	610.609
PA2G4	-0.358	2.792E-05	3.833E-04	5628.716
RABL6	-0.358	2.154E-05	3.043E-04	4106.399
MZT2B	-0.358	7.414E-05	8.875E-04	1362.575
GTPBP3	-0.357	1.323E-04	1.458E-03	622.872
GGH	-0.357	6.733E-04	5.679E-03	371.606
SNHG1	-0.357	2.710E-03	1.761E-02	1585.488
ASPM	-0.357	2.804E-03	1.809E-02	1250.155
ZNF22	-0.357	3.557E-04	3.318E-03	557.156
C9orf40	-0.356	7.675E-04	6.338E-03	387.059
CBX3	-0.356	3.983E-04	3.662E-03	2447.479
CTU2	-0.356	1.158E-04	1.299E-03	612.340
KLF16	-0.355	1.769E-04	1.855E-03	733.604
PSMA3	-0.355	2.375E-03	1.579E-02	1520.145
CHI3L2	-0.355	3.384E-04	3.190E-03	6592.666
HS6ST1	-0.355	8.736E-05	1.019E-03	628.684

OXNAD1	-0.354	3.498E-03	2.151E-02	259.486
DNAJC9	-0.354	2.263E-05	3.174E-04	1473.214
STAG3	-0.353	2.247E-03	1.510E-02	464.729
ERCC6L	-0.352	2.478E-03	1.637E-02	336.926
PHF5A	-0.352	2.251E-04	2.264E-03	584.638
MRPL11	-0.352	4.324E-05	5.559E-04	1068.044
HELLS	-0.352	5.020E-04	4.431E-03	635.635
GINS3	-0.351	7.775E-04	6.396E-03	410.007
AGAP3	-0.351	7.367E-05	8.825E-04	1407.477
PRKRA	-0.351	3.251E-04	3.093E-03	588.346
IMP4	-0.351	1.338E-05	2.033E-04	1456.241
RAD23A	-0.351	1.356E-06	2.714E-05	4063.234
C4orf27	-0.350	2.196E-03	1.483E-02	287.577
KDELR2	-0.350	1.720E-04	1.813E-03	1298.940
YDJC	-0.348	1.144E-04	1.286E-03	679.146
DHX15	-0.348	1.001E-04	1.145E-03	3413.905
CTDSP1	-0.347	1.407E-04	1.534E-03	1997.897
MID1IP1	-0.347	5.768E-04	5.010E-03	1672.800
EMG1	-0.347	4.110E-04	3.750E-03	610.399
SPAG5	-0.347	3.094E-03	1.948E-02	3643.994
RNASEH2A	-0.347	1.011E-03	7.884E-03	708.563
UCK2	-0.347	7.871E-06	1.275E-04	1634.679
TBL2	-0.347	2.725E-04	2.672E-03	759.233
CHRAC1	-0.346	4.416E-05	5.648E-04	615.917
FAM57A	-0.346	4.398E-04	3.968E-03	419.591
ATAD2	-0.346	3.377E-05	4.502E-04	2083.368
SRPRB	-0.345	2.362E-03	1.573E-02	792.680
EBNA1BP2	-0.345	7.930E-04	6.503E-03	1715.613
IL6R	-0.345	5.789E-04	5.019E-03	451.649
KPNA3	-0.345	1.081E-03	8.347E-03	1003.974
ECSIT	-0.345	2.448E-04	2.428E-03	685.950
ACACA	-0.345	7.102E-04	5.947E-03	3074.883
FHOD1	-0.344	2.530E-05	3.499E-04	2344.769
POLD1	-0.344	2.208E-04	2.229E-03	2843.180
UAP1	-0.344	3.158E-03	1.986E-02	504.745
SSB	-0.344	1.618E-03	1.157E-02	1733.703
WRAP53	-0.343	1.103E-03	8.481E-03	615.715
PRR12	-0.343	1.982E-03	1.367E-02	1997.285
MELK	-0.342	3.906E-05	5.081E-04	1065.428
MTFR1	-0.342	4.516E-03	2.612E-02	246.995
SFXN4	-0.341	3.205E-03	2.009E-02	341.506
TIMM13	-0.341	9.780E-06	1.549E-04	1194.463
TIMM44	-0.341	1.617E-05	2.385E-04	1159.434
CDK5R1	-0.341	2.861E-03	1.833E-02	921.864
HNRNPF	-0.341	7.221E-07	1.548E-05	8156.944
ZNF341	-0.340	8.315E-03	4.286E-02	199.103
CDK16	-0.340	9.538E-06	1.516E-04	2268.685
RABGGTB	-0.340	6.316E-03	3.426E-02	1536.833

TST	-0.340	5.239E-03	2.936E-02	391.837
CLUH	-0.340	1.449E-05	2.173E-04	5932.562
SMARCA4	-0.340	2.399E-05	3.330E-04	5524.730
C15orf39	-0.339	9.764E-05	1.123E-03	5551.085
ZMYND19	-0.339	2.372E-04	2.369E-03	557.317
TMEM106C	-0.339	1.659E-04	1.757E-03	1077.836
CYP51A1	-0.339	7.072E-05	8.502E-04	2477.094
SSBP4	-0.339	3.329E-05	4.445E-04	1143.970
CCDC59	-0.339	9.246E-03	4.640E-02	340.004
GPSM2	-0.338	9.222E-03	4.629E-02	280.675
FAM35A	-0.338	9.113E-03	4.588E-02	353.021
MTDH	-0.338	2.648E-04	2.608E-03	4952.676
CARM1	-0.338	1.448E-05	2.173E-04	3480.462
TMCO6	-0.338	5.642E-03	3.118E-02	317.539
TUBGCP3	-0.338	2.654E-04	2.612E-03	818.235
GMPPB	-0.338	1.479E-03	1.075E-02	851.804
FAM136A	-0.336	7.836E-05	9.314E-04	1033.953
DUS3L	-0.335	5.890E-04	5.090E-03	560.832
SEH1L	-0.335	1.415E-04	1.540E-03	949.953
SEC13	-0.335	1.579E-04	1.690E-03	2106.424
SLAIN1	-0.335	2.180E-04	2.207E-03	838.810
NHEJ1	-0.335	6.670E-03	3.586E-02	230.124
MRPL3	-0.334	8.247E-04	6.694E-03	1499.893
ADCY3	-0.334	1.180E-03	8.965E-03	2680.541
MRPL17	-0.334	6.223E-04	5.317E-03	1230.571
CTPS1	-0.334	9.526E-04	7.521E-03	1991.919
NOC4L	-0.333	1.561E-04	1.677E-03	721.896
BDH1	-0.333	2.391E-05	3.322E-04	932.115
PRPF19	-0.333	2.960E-05	4.039E-04	4227.921
CLPP	-0.333	1.140E-05	1.776E-04	1430.841
EXOSC4	-0.333	8.781E-04	7.037E-03	450.621
FAM189B	-0.332	9.508E-04	7.512E-03	854.158
DDX21	-0.332	1.493E-03	1.084E-02	4003.754
GRWD1	-0.332	3.282E-05	4.403E-04	1629.016
ARHGDIA	-0.332	4.017E-06	7.068E-05	12793.335
PGAM1	-0.330	4.623E-06	7.939E-05	13402.890
ZNF296	-0.330	1.100E-03	8.463E-03	408.794
MRPL54	-0.330	2.041E-03	1.399E-02	418.914
RUVBL2	-0.330	2.274E-06	4.306E-05	2197.808
RBBP7	-0.329	3.510E-06	6.263E-05	2907.702
SLCO4A1	-0.329	3.428E-03	2.118E-02	452.673
RRP9	-0.329	8.368E-04	6.774E-03	925.113
CHCHD10	-0.329	2.000E-04	2.050E-03	1004.921
MRPL36	-0.329	1.612E-03	1.154E-02	510.198
TXNL4A	-0.328	7.631E-04	6.306E-03	1260.847
TMEM223	-0.328	9.038E-03	4.560E-02	187.046
GCNT2	-0.328	5.327E-03	2.977E-02	313.099
CETN3	-0.327	1.002E-02	4.923E-02	232.711

TALDO1	-0.327	1.212E-05	1.855E-04	3297.809
RAVER1	-0.327	1.291E-04	1.426E-03	5356.301
CUTC	-0.327	3.676E-03	2.229E-02	402.387
TNFRSF13B	-0.327	1.066E-03	8.243E-03	2601.931
TRMT61A	-0.327	5.811E-04	5.036E-03	472.585
HNRNPH3	-0.327	1.018E-05	1.606E-04	3088.425
ZDHHC4	-0.326	6.828E-03	3.654E-02	356.403
EIF1AX	-0.326	1.226E-03	9.236E-03	1744.740
HMMR	-0.325	5.021E-03	2.844E-02	1113.455
PDE4A	-0.325	2.921E-03	1.864E-02	1076.448
DANCR	-0.325	2.646E-04	2.608E-03	846.464
BOP1	-0.325	9.633E-05	1.110E-03	2511.917
GNPNAT1	-0.325	9.721E-03	4.813E-02	382.804
MLST8	-0.325	3.803E-05	4.978E-04	958.503
C8orf33	-0.325	3.330E-04	3.153E-03	771.349
DNMT1	-0.325	2.994E-04	2.887E-03	8761.395
C19orf70	-0.325	1.938E-04	1.999E-03	666.502
SFPQ	-0.324	5.845E-05	7.208E-04	8267.087
FH	-0.324	3.700E-04	3.434E-03	1781.286
WDR12	-0.324	4.726E-03	2.704E-02	652.579
TDP1	-0.323	9.520E-05	1.100E-03	973.925
MRPL48	-0.323	8.097E-03	4.191E-02	429.226
MRPL2	-0.323	1.909E-04	1.973E-03	672.623
SLC25A1	-0.323	1.584E-03	1.136E-02	2213.185
SMPD4	-0.323	2.402E-04	2.397E-03	2762.646
GSPT1	-0.322	3.373E-04	3.182E-03	3038.825
PPP5C	-0.322	3.743E-05	4.912E-04	1929.713
CYCS	-0.322	1.983E-03	1.367E-02	2502.067
BYSL	-0.321	4.925E-04	4.360E-03	684.592
PSMA7	-0.321	1.777E-04	1.861E-03	3517.012
TUBB	-0.321	9.738E-04	7.634E-03	55836.937
DLAT	-0.321	3.986E-04	3.662E-03	1183.994
SNU13	-0.321	3.460E-06	6.187E-05	2430.230
ATP5G1	-0.321	2.370E-04	2.368E-03	1740.527
PNPT1	-0.321	1.098E-03	8.453E-03	741.768
TUBB4B	-0.321	1.173E-04	1.311E-03	8506.744
UBL4A	-0.320	3.454E-04	3.242E-03	690.331
SNRPD2	-0.320	1.324E-04	1.458E-03	3458.740
SF3B5	-0.320	2.123E-05	3.002E-04	2006.524
TACC3	-0.320	1.083E-03	8.358E-03	5255.151
MAPK12	-0.320	4.824E-04	4.287E-03	605.954
HMGCR	-0.320	1.973E-04	2.031E-03	2959.030
HNRNPM	-0.320	7.933E-06	1.282E-04	7798.304
VPRBP	-0.319	9.534E-04	7.521E-03	1799.051
PSMG3	-0.319	3.047E-04	2.929E-03	585.786
RNF157	-0.318	2.399E-03	1.591E-02	1579.119
SLC4A7	-0.318	6.233E-04	5.319E-03	487.251
MIPEP	-0.318	3.791E-03	2.282E-02	342.698

TUBGCP4	-0.318	2.656E-04	2.613E-03	739.023
SFXN1	-0.318	5.695E-04	4.957E-03	1441.766
DCPS	-0.317	9.610E-05	1.108E-03	1153.185
PARP2	-0.317	1.993E-04	2.047E-03	821.501
ERMP1	-0.317	3.394E-03	2.103E-02	640.169
CLPTM1L	-0.317	5.604E-04	4.890E-03	2081.455
POLR2I	-0.317	1.113E-03	8.534E-03	396.698
ADAP1	-0.317	8.673E-04	6.961E-03	1232.168
KIAA0101	-0.317	3.040E-04	2.924E-03	845.175
THAP7	-0.316	3.415E-03	2.112E-02	386.373
LPCAT4	-0.316	5.042E-04	4.440E-03	1003.043
ILF2	-0.316	2.795E-04	2.723E-03	4309.652
NDUFS7	-0.315	1.727E-04	1.818E-03	1336.672
APBB2	-0.315	9.068E-03	4.570E-02	391.855
XRCC6	-0.315	9.957E-05	1.142E-03	10418.582
ADCK2	-0.315	2.803E-03	1.809E-02	519.533
MTAP	-0.314	2.336E-03	1.558E-02	558.810
ILVBL	-0.314	8.372E-04	6.774E-03	758.896
FTSJ1	-0.314	1.774E-04	1.858E-03	913.829
DHPS	-0.314	6.544E-05	7.977E-04	1355.814
LAS1L	-0.313	2.150E-04	2.186E-03	1673.630
TCERG1	-0.313	9.978E-05	1.143E-03	2389.152
PPP6R1	-0.313	1.198E-04	1.335E-03	9485.115
SLC2A4RG	-0.313	6.178E-03	3.365E-02	840.566
SPDL1	-0.313	1.572E-04	1.685E-03	761.605
SQLE	-0.313	1.064E-04	1.205E-03	2537.328
SRSF4	-0.312	4.188E-06	7.309E-05	3788.351
RFC5	-0.312	3.429E-04	3.226E-03	749.584
COASY	-0.312	2.806E-05	3.848E-04	2782.368
ZNF395	-0.311	9.794E-03	4.838E-02	1291.653
NUDCD1	-0.311	1.580E-03	1.134E-02	488.112
CTSC	-0.311	1.454E-03	1.059E-02	4152.871
SYNCRIP	-0.311	3.201E-04	3.052E-03	4876.927
PGAM5	-0.311	1.821E-05	2.633E-04	1721.173
LRRC58	-0.310	8.397E-04	6.790E-03	921.228
LDHB	-0.310	3.572E-04	3.328E-03	13781.603
SAE1	-0.310	2.333E-05	3.253E-04	3828.936
MRPL55	-0.310	1.743E-03	1.230E-02	465.942
ANXA2	-0.310	5.522E-05	6.853E-04	2754.651
TCP1	-0.310	7.826E-04	6.425E-03	4567.445
IARS	-0.310	1.223E-04	1.361E-03	4664.035
NDUFS6	-0.310	1.666E-04	1.763E-03	1209.922
RANGRF	-0.310	2.180E-04	2.207E-03	681.436
ITPR3	-0.310	6.070E-04	5.216E-03	3863.017
PDIA6	-0.310	1.395E-04	1.523E-03	5167.133
WIBG	-0.309	4.261E-04	3.876E-03	748.621
UTP20	-0.309	3.017E-03	1.907E-02	1097.537
MRPL4	-0.309	3.207E-06	5.790E-05	2524.203

SPCS2	-0.308	7.007E-04	5.880E-03	1410.723
RNH1	-0.308	1.158E-05	1.797E-04	2952.946
CLCN5	-0.308	9.569E-03	4.762E-02	256.632
HSPA5	-0.308	4.547E-03	2.625E-02	13940.368
DTL	-0.308	4.868E-04	4.315E-03	1473.024
NANS	-0.308	9.581E-04	7.543E-03	1020.130
DEXI	-0.308	7.528E-04	6.230E-03	528.575
CCT2	-0.307	6.999E-03	3.732E-02	3958.465
NCAPD2	-0.307	7.544E-03	3.961E-02	9993.120
PRR3	-0.307	1.236E-03	9.298E-03	460.306
SLC25A19	-0.306	6.480E-04	5.499E-03	698.085
LAT2	-0.306	1.550E-06	3.054E-05	6296.735
PCIF1	-0.306	2.611E-03	1.712E-02	986.498
DCXR	-0.305	4.511E-04	4.055E-03	1204.278
CMC2	-0.305	4.379E-03	2.558E-02	560.873
CDCA5	-0.305	8.983E-04	7.169E-03	2259.911
C14orf80	-0.305	3.222E-03	2.014E-02	643.015
COCH	-0.305	1.658E-03	1.180E-02	489.721
PIH1D1	-0.305	1.585E-04	1.694E-03	1203.208
HNRNPDL	-0.304	6.184E-04	5.292E-03	5394.499
TKT	-0.304	2.200E-04	2.224E-03	7955.971
RFX1	-0.304	8.857E-03	4.492E-02	597.498
C21orf2	-0.303	4.443E-03	2.579E-02	394.136
PFDN6	-0.302	1.436E-03	1.050E-02	1114.540
EIF4G1	-0.302	8.050E-04	6.587E-03	19501.047
CCDC61	-0.302	4.622E-03	2.661E-02	430.178
GART	-0.302	3.074E-04	2.949E-03	2757.365
ALDOA	-0.302	2.200E-04	2.224E-03	21755.528
ADAM15	-0.301	2.122E-03	1.442E-02	634.644
MRGBP	-0.301	1.933E-03	1.340E-02	486.622
GMDS	-0.301	4.484E-03	2.599E-02	666.882
COX7B	-0.301	9.742E-03	4.820E-02	1447.271
NREP	-0.301	3.060E-03	1.930E-02	697.322
ESCO2	-0.301	2.309E-03	1.544E-02	424.409
ANAPC11	-0.301	9.554E-04	7.533E-03	902.543
POR	-0.301	6.608E-04	5.592E-03	1692.372
MRPS15	-0.300	5.932E-05	7.303E-04	1406.717
DPY19L1	-0.300	9.625E-03	4.776E-02	270.782
ADK	-0.299	8.123E-04	6.621E-03	862.150
KDM1A	-0.299	3.183E-04	3.040E-03	2415.673
GCDH	-0.299	3.560E-04	3.319E-03	1146.392
NDUFAB1	-0.299	1.430E-03	1.047E-02	1279.186
AURKAIP1	-0.298	1.428E-05	2.150E-04	2020.845
MYBBP1A	-0.298	1.142E-03	8.721E-03	3198.774
POLR3K	-0.297	2.615E-03	1.713E-02	672.058
NUCKS1	-0.297	1.880E-04	1.951E-03	4800.005
MSC	-0.297	4.324E-04	3.921E-03	3236.397
ZNF593	-0.297	8.393E-03	4.316E-02	342.589

RPUSD1	-0.297	2.880E-04	2.786E-03	1041.922
KPNA2	-0.296	4.268E-04	3.881E-03	6371.744
KHSRP	-0.296	4.672E-04	4.170E-03	6987.882
LEF1	-0.296	9.257E-03	4.644E-02	295.096
PRELID1	-0.296	1.797E-04	1.878E-03	3986.425
SSBP1	-0.295	2.901E-03	1.853E-02	1352.632
GRPEL2	-0.295	6.122E-03	3.339E-02	304.357
PRMT3	-0.295	8.240E-03	4.253E-02	456.204
SNAPC2	-0.295	1.336E-03	9.902E-03	642.787
DUSP12	-0.295	2.817E-03	1.815E-02	411.616
CCDC106	-0.294	4.969E-03	2.820E-02	378.967
AAAS	-0.294	2.771E-04	2.708E-03	1384.778
PFDN2	-0.294	1.379E-03	1.017E-02	1016.556
NDUFAF3	-0.294	5.040E-04	4.440E-03	1081.211
COL24A1	-0.293	1.019E-02	4.985E-02	344.662
SCMH1	-0.293	1.057E-03	8.187E-03	831.849
DOLPP1	-0.293	4.141E-03	2.448E-02	471.563
DARS2	-0.293	6.912E-04	5.814E-03	1443.529
EIF2S2	-0.293	8.889E-04	7.108E-03	3168.488
TAGLN2	-0.293	8.513E-06	1.367E-04	6658.770
RNASEH1	-0.293	3.324E-04	3.149E-03	846.532
UBE2E3	-0.293	2.639E-03	1.725E-02	585.836
NUP88	-0.292	2.129E-04	2.169E-03	1307.561
PDAP1	-0.292	3.099E-05	4.201E-04	3192.290
KIF4A	-0.292	7.596E-03	3.979E-02	1371.147
KAT8	-0.291	1.796E-03	1.262E-02	784.306
NELFB	-0.291	7.447E-04	6.177E-03	2383.091
ASF1B	-0.291	2.899E-04	2.801E-03	2711.937
CALM1	-0.291	1.744E-04	1.835E-03	4047.624
CARHSP1	-0.291	4.831E-03	2.756E-02	1055.634
RBX1	-0.291	2.328E-03	1.554E-02	1221.387
PRKDC	-0.291	1.104E-03	8.483E-03	12433.175
CCDC167	-0.290	1.275E-03	9.527E-03	934.546
DDX51	-0.290	6.359E-04	5.411E-03	778.214
NUDC	-0.290	7.561E-05	9.030E-04	3197.026
YARS2	-0.290	1.739E-03	1.228E-02	487.591
SHCBP1	-0.289	2.019E-03	1.387E-02	1118.386
FAM101B	-0.289	8.410E-03	4.322E-02	813.322
NUFIP1	-0.289	9.826E-03	4.846E-02	273.058
UQCRQ	-0.289	1.098E-03	8.453E-03	1392.956
DNAJC1	-0.288	2.355E-03	1.569E-02	491.100
MAGEF1	-0.288	8.345E-04	6.763E-03	783.869
PARP1	-0.288	3.547E-04	3.310E-03	14899.991
DHX9	-0.287	1.554E-03	1.118E-02	6536.124
PHB	-0.287	1.140E-03	8.721E-03	3307.357
PIEZO2	-0.287	9.501E-03	4.730E-02	2156.244
SLC39A3	-0.287	3.187E-03	2.002E-02	849.996
IDH1	-0.287	4.847E-04	4.302E-03	1630.771

ZBTB14	-0.287	4.763E-03	2.722E-02	359.798
TRAPPC5	-0.286	3.549E-03	2.172E-02	899.388
GPATCH4	-0.286	2.822E-03	1.815E-02	1353.387
PTP4A2	-0.286	3.216E-03	2.011E-02	3461.450
TICRR	-0.286	4.491E-03	2.602E-02	1166.184
CNN3	-0.285	3.574E-03	2.183E-02	468.941
GAPDH	-0.285	4.698E-04	4.189E-03	114984.236
PRDX3	-0.285	1.247E-03	9.368E-03	2708.752
ALG3	-0.285	1.505E-03	1.091E-02	691.752
AMD1	-0.285	4.296E-03	2.519E-02	1575.721
FAF1	-0.285	2.261E-03	1.518E-02	1290.667
RTN4	-0.285	9.532E-04	7.521E-03	2036.408
NUP85	-0.284	3.157E-04	3.021E-03	1211.471
COX18	-0.284	4.298E-03	2.519E-02	350.589
AURKA	-0.284	8.548E-03	4.377E-02	1371.366
RPS6KA4	-0.284	2.103E-03	1.432E-02	1150.066
ACAT2	-0.284	3.530E-04	3.299E-03	2610.340
ALKBH5	-0.284	1.185E-03	8.984E-03	2935.409
DPAGT1	-0.284	4.435E-03	2.578E-02	784.566
PCYT2	-0.283	7.790E-04	6.402E-03	1525.725
SNHG5	-0.283	9.823E-03	4.846E-02	1231.007
VEGFB	-0.283	5.253E-03	2.940E-02	957.772
GMPS	-0.282	3.439E-03	2.124E-02	1605.302
SNHG7	-0.282	7.026E-03	3.743E-02	697.564
ABCB8	-0.282	1.395E-03	1.027E-02	1315.559
GMEB2	-0.282	1.494E-03	1.084E-02	962.240
CLPB	-0.282	9.289E-04	7.374E-03	1041.833
FUS	-0.282	1.030E-04	1.171E-03	9980.981
ZW10	-0.281	8.676E-03	4.423E-02	463.697
POU2F1	-0.281	3.538E-03	2.168E-02	1233.328
HM13	-0.281	2.002E-03	1.376E-02	1828.918
RAD21	-0.281	3.584E-04	3.335E-03	4148.764
KIAA1524	-0.281	1.693E-03	1.198E-02	547.060
UCHL5	-0.281	1.689E-03	1.197E-02	787.604
CHCHD2	-0.280	1.569E-04	1.682E-03	5540.413
ALMS1	-0.280	4.412E-03	2.570E-02	839.728
APRT	-0.279	2.740E-05	3.768E-04	2530.315
BCS1L	-0.278	3.938E-03	2.352E-02	596.049
BTLA	-0.278	7.357E-03	3.878E-02	479.767
MRPL37	-0.278	8.322E-05	9.810E-04	2615.296
CFDP1	-0.278	1.458E-03	1.061E-02	1089.741
TMSB4X	-0.278	2.315E-04	2.321E-03	77451.716
EIF4E	-0.278	3.669E-03	2.228E-02	1270.267
TPRN	-0.278	3.858E-03	2.313E-02	636.339
ELAVL1	-0.278	1.267E-03	9.486E-03	3342.408
RPUSD2	-0.278	1.698E-03	1.201E-02	522.131
NUP153	-0.278	2.209E-03	1.489E-02	3081.714
RFT1	-0.277	9.314E-04	7.390E-03	711.970

MRPS12	-0.277	6.414E-04	5.448E-03	1042.303
CNTROB	-0.277	8.144E-04	6.631E-03	1459.327
VDAC1	-0.277	1.224E-03	9.234E-03	7059.758
SEC62	-0.277	1.890E-03	1.317E-02	2116.907
DNA2	-0.277	7.823E-03	4.080E-02	404.074
MSMO1	-0.276	8.087E-03	4.187E-02	1074.292
RPS26	-0.276	1.141E-03	8.721E-03	5685.766
FANCF	-0.276	8.942E-03	4.522E-02	426.593
PGP	-0.276	9.677E-04	7.601E-03	900.425
PUF60	-0.276	5.112E-05	6.429E-04	3131.238
ALG1	-0.276	3.805E-03	2.288E-02	577.995
UTP18	-0.276	5.745E-03	3.165E-02	933.810
RRBP1	-0.276	2.009E-03	1.380E-02	3262.960
POLR3E	-0.276	3.053E-04	2.933E-03	1426.306
CCT4	-0.276	2.625E-03	1.718E-02	4345.894
PES1	-0.276	7.007E-04	5.880E-03	2589.185
CCT8	-0.276	4.084E-03	2.424E-02	4734.883
ERH	-0.276	3.522E-03	2.161E-02	2031.977
MRPL20	-0.275	2.323E-03	1.552E-02	1363.732
NUSAP1	-0.275	1.877E-04	1.949E-03	3127.097
ST6GALNAC4	-0.275	7.069E-03	3.762E-02	1460.970
THAP11	-0.275	2.727E-03	1.770E-02	866.150
HMGN1	-0.275	4.363E-04	3.948E-03	4815.112
YWHAE	-0.274	4.607E-04	4.125E-03	6389.416
ARF1	-0.274	1.268E-04	1.404E-03	8067.543
TONSL	-0.274	1.961E-03	1.358E-02	1561.627
PPIB	-0.274	1.185E-03	8.984E-03	5907.118
PDXP	-0.273	7.718E-04	6.357E-03	1031.335
UQCRC1	-0.273	2.537E-04	2.509E-03	3141.038
KAT5	-0.273	8.410E-04	6.797E-03	1044.236
ATP5D	-0.273	5.302E-04	4.651E-03	1979.031
TSR3	-0.273	3.715E-04	3.446E-03	1001.527
ARPC5L	-0.272	7.319E-04	6.084E-03	1736.153
PPIH	-0.272	5.060E-03	2.860E-02	700.662
ATP5I	-0.272	2.189E-03	1.480E-02	1699.783
NRROS	-0.271	1.649E-03	1.175E-02	1657.584
SLC50A1	-0.271	2.832E-03	1.820E-02	755.194
SF3A2	-0.271	1.406E-03	1.033E-02	2749.750
SARNP	-0.271	4.065E-03	2.416E-02	1393.561
NUP155	-0.271	8.381E-03	4.312E-02	806.514
DGCR8	-0.271	1.133E-03	8.674E-03	1315.025
PIEZO1	-0.271	2.000E-03	1.376E-02	5226.236
CCDC28B	-0.271	6.032E-04	5.194E-03	1061.356
ATXN2L	-0.271	1.495E-03	1.084E-02	8738.391
CHCHD1	-0.270	5.203E-03	2.922E-02	648.846
EMC8	-0.270	1.184E-03	8.984E-03	719.205
Mar.02	-0.269	1.081E-03	8.347E-03	830.757
DVL1	-0.269	1.856E-03	1.299E-02	789.587

SNUPN	-0.269	6.089E-03	3.325E-02	441.289
HLTF	-0.269	1.256E-03	9.419E-03	971.204
FANCA	-0.269	1.985E-03	1.368E-02	1604.915
SLC25A5	-0.269	1.021E-04	1.165E-03	7360.440
SF1	-0.268	2.123E-04	2.164E-03	9037.194
NDUFB10	-0.268	1.185E-03	8.984E-03	1285.943
PFN1	-0.268	3.876E-05	5.051E-04	36605.015
XRCC5	-0.267	4.965E-03	2.820E-02	9187.988
RUVBL1	-0.267	2.381E-03	1.581E-02	1761.392
RNPEPL1	-0.267	9.705E-03	4.806E-02	2561.155
IPO5	-0.267	2.312E-03	1.546E-02	4640.108
COLGALT1	-0.266	5.232E-04	4.597E-03	1497.445
PIGO	-0.266	5.691E-03	3.139E-02	832.220
RDH11	-0.266	7.046E-04	5.906E-03	2046.907
FAM195A	-0.265	2.113E-03	1.437E-02	704.661
FAM78A	-0.265	3.741E-03	2.258E-02	1353.352
CFAP20	-0.265	5.774E-03	3.178E-02	472.552
ME2	-0.265	7.908E-03	4.112E-02	1577.503
JMJD8	-0.265	2.089E-03	1.425E-02	860.239
PCBP2	-0.265	8.547E-04	6.890E-03	11397.451
SRRT	-0.264	3.354E-04	3.167E-03	4718.737
EMP3	-0.264	1.459E-03	1.061E-02	975.290
SLC16A1	-0.263	8.335E-03	4.292E-02	1294.734
SEC63	-0.263	2.937E-03	1.871E-02	1200.435
PRPS1	-0.263	2.953E-03	1.876E-02	1040.641
MDH2	-0.263	1.137E-04	1.279E-03	6373.264
NIF3L1	-0.263	9.934E-03	4.885E-02	440.496
NDUFB6	-0.263	6.683E-03	3.592E-02	651.564
MYO19	-0.262	1.275E-03	9.527E-03	1827.300
ENOSF1	-0.262	3.419E-03	2.113E-02	570.127
MFNG	-0.262	3.265E-04	3.105E-03	1800.506
SAFB	-0.261	7.180E-04	5.992E-03	4275.396
AHSA1	-0.261	1.279E-03	9.545E-03	3545.483
SLC31A1	-0.261	2.680E-03	1.746E-02	839.091
SUPT16H	-0.261	1.364E-03	1.007E-02	7252.424
LYRM4	-0.261	8.034E-03	4.164E-02	444.435
CTDNEP1	-0.260	1.680E-03	1.192E-02	3159.662
SARS2	-0.260	3.686E-03	2.232E-02	690.491
GADD45B	-0.260	2.481E-04	2.459E-03	2569.269
CNPY3	-0.259	4.967E-03	2.820E-02	1834.310
VKORC1L1	-0.259	5.105E-03	2.878E-02	759.261
ZNF276	-0.259	5.404E-03	3.013E-02	1444.485
GYPC	-0.258	6.468E-03	3.497E-02	1053.830
SLC25A23	-0.258	5.480E-03	3.044E-02	7339.088
POLE	-0.258	3.844E-03	2.308E-02	3929.176
PRMT5	-0.258	3.632E-03	2.209E-02	2214.320
LSM7	-0.258	9.974E-04	7.793E-03	1307.041
ETS1	-0.258	7.896E-03	4.107E-02	5046.520

MAFK	-0.258	3.496E-03	2.151E-02	784.626
CHERP	-0.257	2.883E-04	2.787E-03	3324.922
TSFM	-0.257	6.653E-03	3.580E-02	764.022
BARD1	-0.257	7.833E-03	4.081E-02	609.076
CDV3	-0.256	5.142E-03	2.893E-02	6773.775
BLMH	-0.256	3.410E-03	2.110E-02	1690.587
RAC1	-0.256	7.409E-04	6.149E-03	2973.308
NOL12	-0.256	4.652E-03	2.675E-02	579.492
SOD2	-0.256	5.673E-03	3.132E-02	637.466
TLK1	-0.256	3.497E-04	3.276E-03	2249.359
RNASEH2C	-0.256	1.899E-03	1.321E-02	1035.131
DHX33	-0.255	1.630E-03	1.165E-02	1393.999
UBE2I	-0.255	8.363E-04	6.774E-03	2879.487
HNRNPA3	-0.255	8.613E-04	6.928E-03	8873.250
NOC2L	-0.255	4.516E-04	4.057E-03	3504.600
CSK	-0.255	1.168E-03	8.892E-03	8329.244
RQCD1	-0.255	8.963E-04	7.159E-03	2308.558
CHTOP	-0.255	8.271E-04	6.710E-03	1508.926
PSMA5	-0.255	7.505E-03	3.941E-02	3007.795
POLD3	-0.255	1.744E-03	1.230E-02	850.792
KPNB1	-0.254	6.335E-03	3.434E-02	10902.321
UBA2	-0.254	4.911E-03	2.795E-02	1886.774
COX17	-0.254	6.175E-03	3.364E-02	1546.611
ISG20L2	-0.253	1.874E-03	1.309E-02	1525.315
UBALD2	-0.253	9.820E-03	4.846E-02	2588.123
POLR2H	-0.253	8.119E-03	4.198E-02	972.218
SLC39A7	-0.253	8.016E-04	6.563E-03	2773.472
IGSF8	-0.252	4.984E-03	2.825E-02	705.650
RNF26	-0.252	7.283E-03	3.847E-02	1300.676
HNRNPU	-0.252	2.988E-03	1.895E-02	15599.696
YIF1A	-0.251	1.909E-03	1.327E-02	802.931
MRPL24	-0.251	2.996E-03	1.898E-02	1005.052
FAM213B	-0.251	7.747E-03	4.046E-02	483.266
ZFP64	-0.251	5.591E-03	3.097E-02	628.355
TBRG4	-0.251	1.097E-03	8.452E-03	2156.641
TBCB	-0.250	1.055E-03	8.177E-03	1137.464
MED16	-0.250	6.187E-03	3.367E-02	1661.339
DARS	-0.250	3.331E-03	2.070E-02	1864.782
STIP1	-0.250	3.972E-03	2.370E-02	5231.482
RRM1	-0.250	3.033E-03	1.917E-02	3057.224
ATIC	-0.250	5.121E-03	2.885E-02	3560.223
UQCC2	-0.250	1.013E-02	4.965E-02	504.382
HNRNPL	-0.250	1.255E-03	9.419E-03	8344.982
TARS	-0.249	1.976E-03	1.365E-02	3917.436
HDGF	-0.249	4.331E-04	3.923E-03	5805.223
STOML2	-0.249	5.478E-04	4.788E-03	2344.361
SERBP1	-0.249	8.607E-04	6.927E-03	10832.007
TOMM6	-0.249	9.276E-03	4.650E-02	1893.358

POLR2F	-0.249	7.070E-03	3.762E-02	663.772
RNPS1	-0.249	1.954E-04	2.012E-03	4713.946
C21orf59	-0.249	2.866E-03	1.836E-02	669.360
CCT7	-0.248	1.633E-03	1.166E-02	7898.261
U2AF2	-0.248	4.089E-04	3.736E-03	7020.705
ATP5G3	-0.248	2.113E-03	1.437E-02	4221.759
TNFRSF8	-0.248	2.739E-03	1.777E-02	4322.719
SURF4	-0.248	1.517E-03	1.096E-02	3378.235
NDUFA7	-0.247	3.615E-03	2.200E-02	962.249
SYMPK	-0.247	1.028E-03	7.996E-03	5164.183
MAPK9	-0.247	4.492E-03	2.602E-02	799.574
TMEM209	-0.247	5.397E-03	3.011E-02	565.754
PPP1CC	-0.247	6.471E-03	3.498E-02	2554.471
USF2	-0.247	4.958E-03	2.819E-02	2754.980
P4HA1	-0.246	9.736E-03	4.819E-02	1070.059
ADRBK1	-0.246	2.690E-03	1.751E-02	7086.102
NDUFV2	-0.246	2.203E-03	1.486E-02	2202.791
SLFN13	-0.246	3.349E-03	2.078E-02	1479.046
SNRNP40	-0.246	5.049E-03	2.855E-02	1285.824
LSS	-0.246	1.307E-03	9.718E-03	5880.388
CXXC1	-0.246	1.558E-03	1.121E-02	2522.658
MASTL	-0.246	2.624E-03	1.718E-02	917.018
SLC25A11	-0.245	1.884E-03	1.313E-02	1492.901
AKT1	-0.245	1.511E-03	1.093E-02	3847.746
RAD23B	-0.245	3.767E-03	2.272E-02	3054.527
MSH2	-0.244	3.326E-03	2.068E-02	984.726
MRPL34	-0.244	5.167E-03	2.905E-02	838.780
TXNDC11	-0.243	9.804E-03	4.841E-02	2131.142
RITA1	-0.243	3.205E-03	2.009E-02	883.846
SNRNP35	-0.243	7.130E-03	3.784E-02	563.153
POLG	-0.243	1.219E-03	9.206E-03	3472.673
APEX1	-0.243	7.819E-04	6.423E-03	3559.132
PSMB2	-0.243	1.338E-03	9.914E-03	3547.420
DUSP3	-0.242	8.137E-03	4.205E-02	471.805
KNTC1	-0.242	3.264E-03	2.035E-02	1269.645
COX10	-0.242	8.194E-03	4.233E-02	558.165
GRPEL1	-0.242	8.704E-03	4.432E-02	750.464
SRPK1	-0.242	3.043E-03	1.922E-02	2208.265
CBFB	-0.242	5.929E-03	3.251E-02	889.027
GTF2E2	-0.241	9.300E-03	4.659E-02	932.789
HPRT1	-0.241	6.226E-03	3.384E-02	741.039
C1orf35	-0.241	8.908E-03	4.512E-02	492.878
EDRF1	-0.241	4.275E-03	2.512E-02	1257.064
HIRA	-0.241	1.983E-03	1.367E-02	1844.946
C19orf24	-0.241	6.185E-03	3.367E-02	581.876
SNRPC	-0.241	2.518E-03	1.659E-02	1765.998
THAP4	-0.240	9.725E-04	7.628E-03	1195.969
POLR2E	-0.240	1.898E-04	1.963E-03	4621.706

MORF4L2	-0.240	8.245E-03	4.253E-02	2185.556
ERO1A	-0.240	7.011E-03	3.737E-02	1364.215
MRPL16	-0.239	3.880E-03	2.321E-02	1202.489
TRABD	-0.238	8.761E-04	7.025E-03	2702.748
B3GNT2	-0.238	7.038E-04	5.902E-03	1875.110
HNRNPUL1	-0.238	5.582E-03	3.093E-02	10079.672
TMEM243	-0.237	7.477E-03	3.929E-02	898.178
RPN1	-0.237	3.255E-03	2.031E-02	3781.653
COX6A1	-0.237	2.043E-03	1.400E-02	3858.074
NUP107	-0.237	1.180E-03	8.964E-03	1199.534
TMX1	-0.236	5.689E-03	3.139E-02	1069.994
COX5A	-0.236	5.408E-03	3.014E-02	3132.736
DPH2	-0.236	4.667E-03	2.680E-02	1343.498
CDR2	-0.235	4.803E-03	2.743E-02	903.561
MORF4L1	-0.235	2.644E-03	1.727E-02	4726.777
COTL1	-0.235	6.073E-03	3.317E-02	7731.201
MTG1	-0.235	2.370E-03	1.577E-02	1122.879
NDUFS8	-0.235	4.056E-03	2.412E-02	1417.935
RRP7A	-0.235	6.280E-04	5.350E-03	2053.113
ACAD9	-0.235	2.840E-03	1.824E-02	1381.580
MVD	-0.234	6.216E-04	5.313E-03	3807.173
HMGXB4	-0.234	5.570E-03	3.087E-02	860.137
TTLL4	-0.233	4.503E-03	2.607E-02	1244.144
CYC1	-0.233	8.508E-04	6.869E-03	2726.189
C7orf49	-0.233	4.178E-03	2.466E-02	1743.371
TIMM50	-0.232	5.448E-03	3.030E-02	1041.155
FTL	-0.231	7.885E-03	4.102E-02	46350.220
VPS26B	-0.231	8.474E-03	4.349E-02	1128.610
PDHB	-0.230	9.387E-03	4.692E-02	1524.119
FOXRED1	-0.229	5.894E-03	3.236E-02	1259.409
TYSND1	-0.229	7.214E-03	3.821E-02	969.369
RPL35	-0.229	4.122E-03	2.442E-02	11569.657
PROSER1	-0.229	2.149E-03	1.457E-02	2086.420
PELP1	-0.228	2.092E-03	1.425E-02	3109.612
AKAP1	-0.228	6.521E-03	3.523E-02	1219.247
EPRS	-0.228	5.563E-03	3.084E-02	4477.584
MCRS1	-0.227	2.469E-03	1.633E-02	1417.782
AFG3L1P	-0.227	7.454E-03	3.921E-02	800.575
ICMT	-0.227	9.157E-03	4.603E-02	1607.346
KDELR1	-0.226	8.509E-03	4.363E-02	2211.339
TMUB1	-0.226	1.002E-02	4.923E-02	1308.414
IRAK1	-0.226	2.084E-03	1.423E-02	3769.209
LARP1	-0.226	5.063E-03	2.860E-02	10065.414
AIFM1	-0.226	2.950E-03	1.875E-02	2345.370
FNBP1	-0.226	1.846E-03	1.292E-02	24337.936
SRP9	-0.225	6.740E-03	3.614E-02	2142.766
MRPL38	-0.225	2.943E-03	1.873E-02	1552.412
SRSF9	-0.224	2.496E-03	1.646E-02	3835.644

HNRNPC	-0.224	1.454E-03	1.059E-02	12196.517
APEH	-0.224	1.430E-03	1.047E-02	3789.526
WSB2	-0.223	2.005E-03	1.378E-02	1306.085
ZFAS1	-0.223	9.425E-03	4.702E-02	1266.637
ACAA2	-0.222	7.091E-03	3.773E-02	1202.871
PSMC3	-0.222	4.710E-03	2.698E-02	2956.671
PABPC4	-0.222	2.768E-03	1.793E-02	7596.425
ILKAP	-0.222	6.780E-03	3.631E-02	776.490
TUBG1	-0.221	5.501E-03	3.053E-02	2508.913
CTBP1	-0.221	7.480E-03	3.929E-02	3959.519
TUBA1C	-0.221	4.515E-03	2.612E-02	5579.044
RPS12	-0.221	4.962E-03	2.820E-02	18170.844
UBE2M	-0.221	3.095E-03	1.948E-02	1638.567
NUP50	-0.221	2.023E-03	1.388E-02	2836.064
TBCD	-0.220	9.330E-03	4.671E-02	2359.922
CS	-0.220	2.060E-03	1.409E-02	6508.360
AKR1B1	-0.220	9.598E-03	4.772E-02	2015.445
ACTB	-0.220	1.037E-03	8.062E-03	321700.389
SNRNP70	-0.219	1.020E-03	7.947E-03	5768.204
CCDC12	-0.219	9.846E-03	4.854E-02	1040.913
UBE2D2	-0.219	6.637E-03	3.572E-02	1558.363
PHRF1	-0.219	7.599E-03	3.980E-02	3428.613
RBM12	-0.219	4.394E-03	2.564E-02	2189.391
SEC23A	-0.217	5.980E-03	3.276E-02	1098.327
CLCN6	-0.217	6.222E-03	3.383E-02	1396.778
PSMD3	-0.217	4.579E-03	2.638E-02	4635.494
SDCCAG3	-0.217	6.689E-03	3.594E-02	837.466
COMT	-0.217	4.604E-03	2.652E-02	987.881
RAB1B	-0.217	4.758E-03	2.721E-02	5159.048
FOCAD	-0.216	5.597E-03	3.097E-02	2870.845
KHDRBS1	-0.216	2.092E-03	1.425E-02	5445.937
MRPL9	-0.215	7.252E-03	3.834E-02	1073.594
PEBP1	-0.215	1.754E-03	1.235E-02	3941.990
CDK1	-0.215	9.584E-03	4.768E-02	996.394
TUFM	-0.214	1.764E-03	1.242E-02	5241.615
ACLY	-0.214	8.818E-03	4.480E-02	9855.565
SMC2	-0.214	2.299E-03	1.539E-02	1646.319
REXO1	-0.214	4.023E-03	2.396E-02	2137.422
OAZ1	-0.214	3.512E-03	2.157E-02	14836.548
TIMM17B	-0.213	8.356E-03	4.302E-02	913.650
RAB35	-0.213	4.407E-03	2.569E-02	3557.498
ACTG1	-0.213	3.471E-03	2.137E-02	107544.129
USP5	-0.212	2.533E-03	1.667E-02	4155.329
TIAL1	-0.211	6.703E-03	3.599E-02	1894.456
MAGED1	-0.211	3.305E-03	2.059E-02	5786.454
PAF1	-0.211	6.734E-03	3.613E-02	1597.690
TAF15	-0.210	4.796E-03	2.740E-02	5591.587
DHX30	-0.210	2.137E-03	1.451E-02	3305.315

NPRL3	-0.210	6.713E-03	3.603E-02	1674.132
SRRM1	-0.210	3.723E-03	2.248E-02	4276.961
ARMC6	-0.209	6.885E-03	3.682E-02	1002.799
ZNF706	-0.209	7.396E-03	3.898E-02	1031.026
BUB3	-0.208	4.052E-03	2.411E-02	2787.926
FUBP1	-0.208	2.888E-03	1.847E-02	4194.404
SLC29A1	-0.208	9.627E-03	4.776E-02	1490.201
DESI1	-0.208	2.927E-03	1.866E-02	2525.331
PDHA1	-0.206	5.386E-03	3.006E-02	2471.899
CBX1	-0.206	7.115E-03	3.781E-02	1917.833
PPIF	-0.206	7.698E-03	4.026E-02	3168.746
PTGES2	-0.206	3.685E-03	2.232E-02	1515.589
SF3B2	-0.205	9.624E-03	4.776E-02	9374.828
CHD3	-0.205	7.566E-03	3.967E-02	5601.383
THRAP3	-0.205	5.680E-03	3.135E-02	7391.802
FARSA	-0.204	8.994E-03	4.543E-02	3005.055
PGD	-0.204	8.887E-03	4.505E-02	4790.777
ELAC2	-0.204	5.719E-03	3.152E-02	3037.854
EZH2	-0.204	6.840E-03	3.659E-02	1916.671
CD48	-0.203	5.317E-03	2.973E-02	7791.640
NACA	-0.201	3.692E-03	2.235E-02	11697.920
FKBP5	-0.201	7.118E-03	3.781E-02	2820.171
ARHGAP17	-0.201	5.616E-03	3.107E-02	5611.522
MED22	-0.201	6.576E-03	3.546E-02	1385.703
CPSF6	-0.200	2.626E-03	1.718E-02	3037.948
LGALS1	-0.199	7.657E-03	4.007E-02	5553.898
CFL1	-0.198	2.657E-03	1.735E-02	25112.033
PHB2	-0.195	1.927E-03	1.336E-02	7007.584
PSMB6	-0.195	7.400E-03	3.898E-02	1958.681
FDFT1	-0.195	3.214E-03	2.011E-02	7049.099
GOT2	-0.195	7.553E-03	3.964E-02	3662.235
AK2	-0.194	1.010E-02	4.956E-02	4225.048
EWSR1	-0.194	3.000E-03	1.900E-02	9845.620
LPGAT1	-0.191	4.535E-03	2.621E-02	2999.100
OXCT1	-0.189	8.404E-03	4.320E-02	2268.360
PSIP1	-0.188	4.538E-03	2.621E-02	6215.432
PI4K2B	-0.187	9.915E-03	4.880E-02	1697.998
LSM14A	-0.186	6.454E-03	3.491E-02	2563.676
TCEB2	-0.186	9.338E-03	4.671E-02	2076.678
GPS1	-0.186	8.536E-03	4.374E-02	2154.924
GLUD1	-0.184	8.486E-03	4.354E-02	2696.465
RBMX	-0.179	8.980E-03	4.539E-02	5020.978
ANXA5	-0.174	8.016E-03	4.158E-02	2981.140
NCLN	-0.173	8.914E-03	4.513E-02	2839.345
ARF6	-0.169	6.935E-03	3.704E-02	3721.021
LRPAP1	0.177	9.774E-03	4.833E-02	1857.249
OS9	0.178	8.597E-03	4.394E-02	5738.354
RABGAP1	0.179	1.002E-02	4.923E-02	1668.615

SNX1	0.179	4.229E-03	2.488E-02	3829.369
PRR13	0.180	8.447E-03	4.337E-02	4136.462
CTSA	0.183	8.774E-03	4.461E-02	2134.861
ATP6AP1	0.184	6.141E-03	3.348E-02	2141.083
DCTN5	0.185	8.695E-03	4.430E-02	2716.727
BSG	0.185	5.442E-03	3.029E-02	8195.005
HDAC1	0.186	4.225E-03	2.488E-02	5844.186
NCSTN	0.189	4.669E-03	2.680E-02	2508.139
N4BP1	0.189	6.904E-03	3.690E-02	1666.634
EFCAB14	0.189	5.948E-03	3.261E-02	3467.491
GRIPAP1	0.193	9.484E-03	4.724E-02	2879.013
USP4	0.193	6.323E-03	3.429E-02	2386.990
RASSF2	0.193	9.139E-03	4.595E-02	3015.190
PARN	0.194	8.552E-03	4.377E-02	1959.526
CHPF2	0.195	8.727E-03	4.442E-02	2181.104
BTAF1	0.196	6.958E-03	3.713E-02	3324.376
EIF3F	0.198	4.252E-03	2.501E-02	7428.633
GCSAM	0.198	7.561E-03	3.966E-02	3337.326
ALAS1	0.199	7.623E-03	3.991E-02	2543.743
PTEN	0.199	3.793E-03	2.283E-02	3120.553
CLEC16A	0.200	9.779E-03	4.834E-02	2700.392
ST13	0.200	2.993E-03	1.897E-02	5913.113
TOX4	0.201	4.501E-03	2.606E-02	2252.365
KIAA0319L	0.202	9.067E-03	4.570E-02	2808.727
IFIH1	0.202	8.774E-03	4.461E-02	3178.299
EXOC3	0.203	8.592E-03	4.393E-02	2072.948
STRADA	0.203	9.400E-03	4.696E-02	1991.610
CES2	0.204	7.450E-03	3.921E-02	1510.026
CDC42BPB	0.204	7.926E-03	4.119E-02	1806.565
SNAP23	0.204	3.783E-03	2.279E-02	1727.477
TTC17	0.204	4.293E-03	2.519E-02	1775.965
TP53BP1	0.204	7.355E-03	3.878E-02	2766.855
PTTG1IP	0.205	3.196E-03	2.005E-02	1917.427
CPNE3	0.205	5.026E-03	2.845E-02	2534.003
RFTN1	0.205	2.969E-03	1.885E-02	9395.177
LANCL1	0.206	1.020E-02	4.989E-02	927.758
PIGG	0.206	9.619E-03	4.776E-02	957.369
JAK1	0.206	4.709E-03	2.698E-02	5176.353
GALNT10	0.207	8.114E-03	4.197E-02	3395.830
SEMA7A	0.208	8.718E-03	4.438E-02	15153.878
STAT1	0.208	2.713E-03	1.762E-02	27405.280
NR3C1	0.208	3.679E-03	2.230E-02	2854.258
PHIP	0.211	9.686E-03	4.799E-02	2388.697
NAGK	0.212	7.252E-03	3.834E-02	1844.589
GABARAP	0.213	2.091E-03	1.425E-02	3952.265
TBC1D5	0.214	8.680E-03	4.423E-02	3602.209
WIPF2	0.215	8.578E-03	4.389E-02	1788.538
BTBD7	0.215	7.938E-03	4.124E-02	898.019

PACSIN2	0.215	2.531E-03	1.666E-02	2684.702
VPS39	0.216	1.360E-03	1.005E-02	4093.494
FTSJ2	0.217	4.436E-03	2.578E-02	1455.868
REST	0.217	8.082E-03	4.186E-02	1087.092
HIF1AN	0.217	3.368E-03	2.088E-02	2917.129
ZNF266	0.218	6.056E-03	3.310E-02	1412.439
DDX26B	0.218	9.334E-03	4.671E-02	860.949
MED23	0.219	5.240E-03	2.936E-02	962.139
RELT	0.220	7.201E-03	3.815E-02	1626.874
ADPRH	0.220	7.832E-03	4.081E-02	1413.443
NIN	0.220	2.308E-03	1.544E-02	8916.511
CXXC5	0.221	4.100E-03	2.431E-02	1496.855
SIN3B	0.221	4.367E-03	2.553E-02	2316.674
NCKAP1L	0.221	2.426E-03	1.607E-02	7538.280
PARM1	0.222	9.963E-03	4.897E-02	1758.807
C22orf46	0.222	3.590E-03	2.190E-02	1045.552
PIP4K2C	0.222	2.884E-03	1.845E-02	1124.148
FURIN	0.222	2.953E-03	1.876E-02	2237.667
PBX3	0.222	5.860E-03	3.222E-02	1287.790
CNNM4	0.223	4.840E-03	2.760E-02	1370.167
GPR107	0.223	2.583E-03	1.694E-02	1561.985
MRPL49	0.223	2.950E-03	1.875E-02	1969.986
KLHL12	0.224	2.085E-03	1.423E-02	1578.683
USP8	0.224	3.686E-03	2.232E-02	1314.036
DENND2D	0.224	1.308E-03	9.719E-03	2635.336
DAPP1	0.224	5.354E-03	2.989E-02	1588.041
ACAP2	0.225	2.449E-03	1.622E-02	1573.991
WBP1L	0.225	1.997E-03	1.374E-02	1437.532
UBE4A	0.225	3.979E-03	2.374E-02	1924.219
FAM53C	0.225	3.543E-03	2.169E-02	2488.390
GRAMD1A	0.226	5.253E-03	2.940E-02	2898.471
IL16	0.226	2.542E-03	1.671E-02	4391.028
RALB	0.226	2.150E-03	1.457E-02	1451.684
ATP6V1A	0.226	2.978E-03	1.889E-02	2333.155
LETMD1	0.227	4.824E-03	2.753E-02	1256.546
UBA7	0.227	9.896E-03	4.872E-02	3854.164
SPN	0.228	4.460E-03	2.586E-02	12438.476
PACS2	0.228	9.132E-03	4.593E-02	843.290
PIK3AP1	0.228	4.094E-03	2.428E-02	6007.996
IRF2BPL	0.229	9.611E-03	4.775E-02	1675.554
TMEM87B	0.229	8.417E-03	4.323E-02	772.947
WDR37	0.229	5.170E-03	2.905E-02	747.067
ALDH5A1	0.229	7.708E-03	4.030E-02	985.346
ALS2	0.231	7.570E-03	3.967E-02	710.761
CAPN7	0.231	8.324E-03	4.289E-02	637.447
HEXA	0.231	2.785E-03	1.801E-02	1907.588
IRF5	0.231	4.975E-03	2.821E-02	6057.552
CELF2	0.231	1.254E-03	9.419E-03	4195.499

INPP5D	0.231	3.780E-03	2.279E-02	9841.685
TNFRSF1B	0.232	1.234E-03	9.285E-03	4454.246
ELMOD3	0.232	7.961E-03	4.135E-02	645.973
ZNF84	0.232	9.819E-03	4.846E-02	729.493
C6orf89	0.232	9.578E-04	7.543E-03	2265.958
RAB5B	0.232	4.467E-04	4.023E-03	4013.698
LASP1	0.232	5.124E-03	2.885E-02	8844.740
IGBP1	0.233	3.372E-03	2.090E-02	1536.432
RNF111	0.233	5.207E-03	2.923E-02	1079.941
STX12	0.233	5.980E-03	3.276E-02	722.605
ABI3	0.233	2.404E-03	1.593E-02	2601.106
PPP1R9B	0.234	2.878E-03	1.843E-02	7620.123
TSPYL1	0.234	1.271E-03	9.509E-03	2104.323
STX16	0.234	2.884E-03	1.845E-02	2798.802
CYBRD1	0.234	3.226E-03	2.016E-02	1194.954
SLC12A6	0.235	9.485E-04	7.498E-03	1676.396
NCF1	0.235	2.667E-03	1.740E-02	4937.463
RSU1	0.235	6.054E-03	3.310E-02	1305.697
SLC12A9	0.236	3.403E-03	2.107E-02	1473.416
VAMP7	0.236	8.801E-03	4.473E-02	762.886
VAV2	0.236	1.692E-03	1.198E-02	4407.552
UCP2	0.237	6.342E-03	3.437E-02	5897.464
IDS	0.237	6.147E-04	5.263E-03	4248.115
MS4A1	0.237	3.748E-03	2.261E-02	16992.313
VIPAS39	0.238	1.752E-03	1.235E-02	995.374
HEATR5B	0.238	3.570E-03	2.183E-02	1651.940
DNAJB2	0.238	9.583E-04	7.543E-03	1660.914
GBE1	0.238	1.217E-03	9.198E-03	1608.391
ZBTB40	0.239	1.529E-03	1.103E-02	2559.327
STAT3	0.239	1.300E-03	9.676E-03	8175.401
TAOK1	0.239	7.674E-03	4.015E-02	1605.650
HCP5	0.240	3.195E-04	3.048E-03	5137.416
LGALS3	0.240	5.069E-03	2.861E-02	1244.640
SLC12A7	0.241	9.849E-03	4.854E-02	1991.837
CHST2	0.241	4.194E-03	2.473E-02	1209.318
HIF1A	0.241	8.085E-04	6.608E-03	2383.481
SCRIB	0.242	4.074E-03	2.419E-02	3958.763
AKAP9	0.242	5.889E-03	3.234E-02	1744.170
SH3BP5	0.242	2.456E-03	1.626E-02	979.082
CLP1	0.243	8.658E-03	4.417E-02	553.832
ZNF292	0.243	6.274E-03	3.407E-02	1679.576
CDK19	0.243	5.647E-03	3.119E-02	1205.162
C19orf66	0.243	6.587E-03	3.550E-02	2655.535
POMGNT1	0.243	3.673E-03	2.229E-02	1232.946
AFF4	0.243	1.765E-03	1.242E-02	1430.930
ZER1	0.243	4.288E-03	2.517E-02	1917.312
ATRX	0.244	9.124E-03	4.592E-02	1928.892
SLAMF7	0.244	6.694E-03	3.595E-02	7081.489

NEMP1	0.245	4.111E-03	2.436E-02	1294.571
CYHR1	0.245	4.351E-03	2.547E-02	834.126
GSTK1	0.246	6.738E-04	5.679E-03	2271.332
PML	0.246	3.702E-03	2.240E-02	4781.580
PRKCD	0.246	4.597E-04	4.121E-03	5159.978
DYNC1H1	0.246	6.021E-03	3.294E-02	16757.661
DMXL1	0.246	1.532E-03	1.105E-02	1612.014
ARHGEF12	0.246	2.555E-03	1.678E-02	2916.104
ETV5	0.246	8.824E-03	4.480E-02	710.013
PDE7A	0.247	1.684E-03	1.195E-02	1068.488
LRMP	0.247	6.769E-03	3.627E-02	5310.165
MICAL1	0.247	9.330E-03	4.671E-02	4798.117
DENND4C	0.247	1.443E-03	1.052E-02	1573.321
FAM21C	0.247	2.166E-03	1.466E-02	1329.284
SNX20	0.248	1.600E-03	1.146E-02	1406.610
KIAA0922	0.248	5.775E-04	5.013E-03	8508.314
MON2	0.248	1.658E-03	1.180E-02	1198.568
GABARAPL2	0.248	3.061E-03	1.930E-02	1348.453
ITFG1	0.248	5.065E-03	2.861E-02	556.421
BCL7C	0.249	8.014E-03	4.158E-02	515.249
TFDP2	0.249	1.204E-03	9.110E-03	1190.426
KIAA1109	0.250	5.870E-03	3.226E-02	1820.656
NHLRC3	0.250	4.180E-03	2.466E-02	708.889
RPS19	0.251	3.991E-04	3.664E-03	30618.857
UBA3	0.251	3.875E-03	2.321E-02	1125.287
UBR1	0.251	2.565E-03	1.683E-02	1286.807
AP1G2	0.251	6.945E-03	3.708E-02	4377.979
BCL2	0.251	1.593E-03	1.142E-02	4050.755
SCPEP1	0.252	3.343E-04	3.159E-03	1851.127
ZKSCAN1	0.252	3.658E-03	2.222E-02	2324.023
TRAF1	0.252	8.823E-03	4.480E-02	18834.922
MAP2K2	0.253	3.259E-03	2.032E-02	5128.293
ZNF264	0.253	5.069E-03	2.861E-02	552.023
EPG5	0.253	2.752E-03	1.784E-02	1601.708
ZBTB7B	0.253	6.315E-03	3.426E-02	2641.044
CASP4	0.253	5.503E-03	3.053E-02	877.997
ARNTL	0.254	9.619E-03	4.776E-02	463.308
ATPIF1	0.254	7.242E-03	3.831E-02	3334.081
TTPAL	0.254	3.122E-03	1.964E-02	1287.571
APTX	0.254	1.679E-03	1.192E-02	940.410
PCYT1A	0.254	2.749E-03	1.783E-02	1198.505
GNPTAB	0.255	3.796E-03	2.284E-02	1443.101
ARHGAP26	0.255	4.910E-03	2.795E-02	697.420
HSDL2	0.255	6.870E-04	5.781E-03	1119.573
BRWD1	0.255	4.014E-03	2.392E-02	747.436
EPM2AIP1	0.256	1.021E-03	7.949E-03	1488.743
CDS2	0.256	9.238E-04	7.337E-03	2199.376
SMG6	0.257	7.840E-04	6.433E-03	1922.174

ARFGEF1	0.257	1.837E-03	1.286E-02	1461.114
AIM1	0.257	2.754E-04	2.697E-03	5343.011
ZNF623	0.258	5.597E-03	3.097E-02	694.941
GLTSCR2	0.258	3.831E-04	3.546E-03	7514.723
CFLAR	0.259	9.178E-04	7.299E-03	8871.954
SIRT2	0.259	4.142E-03	2.448E-02	957.272
PLD3	0.259	1.442E-03	1.052E-02	2268.932
RYBP	0.260	8.622E-03	4.403E-02	745.865
CD99	0.260	5.230E-03	2.934E-02	2043.663
ATRN	0.260	2.038E-03	1.397E-02	772.517
PKN2	0.260	5.073E-03	2.862E-02	646.975
OPTN	0.260	1.925E-04	1.987E-03	2333.589
THTPA	0.261	4.533E-03	2.621E-02	525.226
ZDHHC14	0.263	8.245E-03	4.253E-02	1060.311
ZNF655	0.264	8.113E-04	6.618E-03	1741.864
HECTD4	0.264	7.128E-03	3.784E-02	5733.898
ZMYM6	0.265	5.215E-03	2.927E-02	499.254
C18orf25	0.266	2.690E-03	1.751E-02	645.672
KIAA0355	0.266	1.074E-03	8.302E-03	847.426
SGSM2	0.266	3.450E-03	2.128E-02	2101.310
STK17A	0.267	1.098E-03	8.453E-03	1197.918
PSMD5-AS1	0.267	6.628E-04	5.605E-03	856.433
ZNF189	0.267	4.069E-03	2.417E-02	519.099
NEDD4L	0.267	2.082E-03	1.423E-02	1056.971
ATP1B1	0.267	3.810E-03	2.290E-02	710.829
TMF1	0.267	3.051E-03	1.926E-02	1442.628
TULP4	0.268	1.974E-03	1.364E-02	961.758
PRKAB2	0.268	1.017E-02	4.980E-02	416.077
PCNA	0.268	6.616E-05	8.046E-04	4632.651
CHD6	0.269	3.165E-03	1.989E-02	2542.771
QSOX1	0.269	1.833E-03	1.284E-02	1050.826
WSB1	0.269	1.025E-03	7.975E-03	2410.752
CTSH	0.269	4.348E-04	3.937E-03	10398.624
PTPN22	0.269	2.670E-03	1.741E-02	998.180
MGME1	0.269	1.882E-03	1.313E-02	653.073
C17orf89	0.270	6.049E-03	3.309E-02	629.490
MACC1	0.270	1.659E-03	1.180E-02	1040.537
ACOX1	0.270	4.949E-04	4.376E-03	1154.337
DNAJB14	0.270	4.454E-03	2.584E-02	973.131
NBEAL2	0.271	3.717E-03	2.245E-02	11031.400
SSH1	0.271	1.397E-03	1.028E-02	3902.593
NCOA3	0.271	1.749E-04	1.838E-03	7768.597
RALGAPA2	0.271	4.907E-03	2.794E-02	1728.547
PIK3R3	0.272	4.245E-04	3.864E-03	1682.011
SLC15A3	0.272	1.376E-03	1.015E-02	1056.638
CD40	0.272	4.703E-03	2.697E-02	4162.727
SP110	0.272	2.776E-04	2.708E-03	3601.274
TMBIM1	0.272	3.083E-03	1.942E-02	1499.310

HLA-DRB1	0.272	2.162E-03	1.464E-02	5783.761
FYN	0.272	6.381E-04	5.427E-03	1678.487
ICAM1	0.273	2.636E-03	1.723E-02	7586.213
DEDD2	0.273	3.499E-03	2.151E-02	650.700
A4GALT	0.273	2.810E-03	1.812E-02	816.144
IRAK3	0.273	9.215E-03	4.627E-02	473.763
PPM1F	0.273	4.130E-03	2.443E-02	1033.334
RICTOR	0.274	4.064E-03	2.416E-02	592.545
TOR1A	0.274	2.445E-04	2.427E-03	1147.844
LOC728175	0.274	4.624E-03	2.661E-02	968.913
ESYT2	0.274	4.647E-04	4.153E-03	1895.585
CCDC115	0.275	1.923E-03	1.335E-02	798.629
BCL10	0.275	3.254E-03	2.031E-02	558.666
IQCE	0.276	3.007E-03	1.903E-02	512.264
ITSN2	0.276	1.835E-04	1.911E-03	2607.646
TRIM66	0.277	6.596E-04	5.585E-03	1816.172
ETV6	0.277	1.625E-04	1.730E-03	2206.090
C2CD2	0.277	3.213E-03	2.011E-02	616.173
TM7SF2	0.278	2.708E-03	1.760E-02	1171.108
MANBA	0.278	9.458E-04	7.484E-03	925.025
PEA15	0.278	8.073E-04	6.602E-03	4867.694
LRRC16A	0.278	1.143E-03	8.728E-03	1049.906
CTSD	0.278	1.331E-04	1.465E-03	2240.257
UBN2	0.278	4.128E-03	2.443E-02	635.540
LNPEP	0.279	2.244E-03	1.509E-02	1764.379
ARRB1	0.279	8.935E-03	4.521E-02	463.701
MARCKSL1	0.279	1.201E-03	9.095E-03	12338.681
HLA-DPA1	0.279	7.431E-05	8.888E-04	16462.329
HLA-DRA	0.279	4.092E-04	3.737E-03	32696.869
WDR59	0.280	1.160E-04	1.299E-03	2046.319
ARAP2	0.280	1.195E-03	9.051E-03	817.594
IP6K2	0.280	2.015E-04	2.064E-03	1730.231
ADAM22	0.281	1.425E-03	1.044E-02	699.008
EBI3	0.281	5.796E-03	3.189E-02	4322.068
TLR6	0.282	5.330E-03	2.978E-02	762.746
EXOC1	0.282	9.422E-04	7.460E-03	709.368
UGT2B17	0.283	6.741E-03	3.614E-02	532.643
ZNF37A	0.283	8.079E-03	4.186E-02	973.795
USP49	0.283	3.527E-03	2.163E-02	653.344
PSMG3-AS1	0.283	5.081E-03	2.865E-02	451.324
N4BP2	0.283	9.430E-03	4.703E-02	587.627
APOBEC3F	0.283	4.835E-04	4.293E-03	1166.684
ITPR1	0.284	6.595E-04	5.585E-03	3205.860
ATP10D	0.284	1.562E-03	1.123E-02	1394.950
TMUB2	0.285	4.285E-03	2.516E-02	992.856
NCOA4	0.286	3.488E-05	4.625E-04	4129.344
IFI44	0.286	2.846E-04	2.762E-03	3758.649
HNF1B	0.288	8.413E-03	4.322E-02	1055.629

KSR1	0.288	2.193E-03	1.482E-02	2441.347
HLA-DRB5	0.288	4.856E-03	2.767E-02	431.156
PLCG2	0.289	2.688E-04	2.643E-03	13701.980
C5orf42	0.289	2.933E-03	1.869E-02	536.353
GNG2	0.290	5.860E-04	5.075E-03	1353.769
TET2	0.290	1.028E-03	7.996E-03	1920.495
SERTAD2	0.290	3.341E-04	3.159E-03	1662.691
ZNF561	0.291	4.439E-03	2.578E-02	450.562
TMEM159	0.291	8.761E-03	4.457E-02	326.538
PLBD2	0.291	3.335E-03	2.071E-02	934.194
EMID1	0.291	3.604E-03	2.196E-02	1853.240
KRBA1	0.292	2.799E-03	1.808E-02	688.216
KIAA1468	0.292	5.102E-04	4.491E-03	1052.533
SCIMP	0.292	1.319E-03	9.794E-03	2857.216
NEK8	0.292	4.313E-03	2.526E-02	750.606
ZDBF2	0.292	2.026E-03	1.390E-02	608.418
ARID4B	0.292	3.198E-03	2.006E-02	1583.477
MTA2	0.292	2.047E-05	2.913E-04	6726.702
CHD9	0.292	1.222E-03	9.222E-03	1716.038
CEP120	0.293	1.264E-03	9.477E-03	568.635
JAK3	0.293	1.415E-04	1.540E-03	3740.358
TWSG1	0.294	2.227E-03	1.500E-02	884.999
RAB29	0.294	1.110E-03	8.520E-03	1207.893
CERS5	0.295	1.412E-03	1.036E-02	666.163
FAM129A	0.295	1.534E-03	1.106E-02	2803.945
NCEH1	0.295	9.448E-03	4.708E-02	259.712
NQO1	0.296	4.661E-03	2.677E-02	947.268
SIL1	0.296	1.032E-04	1.172E-03	1018.614
GEN1	0.296	4.233E-03	2.490E-02	912.474
CPEB2	0.296	4.370E-03	2.554E-02	422.527
SQSTM1	0.297	9.100E-05	1.057E-03	6892.197
LSP1	0.297	2.079E-05	2.950E-04	12591.446
CTSS	0.298	3.650E-04	3.393E-03	2122.328
RALGAPB	0.299	1.638E-04	1.740E-03	2051.092
ZDHHC21	0.299	5.128E-03	2.886E-02	648.014
MVP	0.299	1.096E-04	1.238E-03	7039.891
RABGGTA	0.299	6.732E-04	5.679E-03	1115.900
SPIB	0.300	4.389E-03	2.562E-02	4037.582
KIAA0753	0.300	1.432E-03	1.047E-02	640.424
PARP10	0.300	2.394E-03	1.589E-02	4379.967
GNS	0.301	3.441E-04	3.232E-03	1735.245
ADNP2	0.301	1.361E-04	1.494E-03	1105.321
TP53TG1	0.301	1.892E-03	1.317E-02	380.932
GOLGB1	0.302	6.859E-05	8.276E-04	4719.923
PCNX	0.302	1.601E-04	1.707E-03	2718.992
ASH1L	0.303	5.287E-05	6.622E-04	2643.903
UXS1	0.303	4.432E-03	2.578E-02	557.353
PRMT2	0.303	2.206E-04	2.228E-03	1668.945

IFIT3	0.303	1.255E-04	1.391E-03	4100.800
OFD1	0.303	7.095E-03	3.773E-02	1452.800
DNMT3A	0.303	1.849E-04	1.924E-03	1538.690
SERPINB9	0.304	1.368E-03	1.009E-02	4681.281
VAMP1	0.304	2.811E-03	1.812E-02	2543.291
PINK1	0.304	8.839E-03	4.485E-02	339.123
CD226	0.304	4.126E-03	2.443E-02	2162.832
CEP290	0.305	8.024E-03	4.160E-02	627.598
ASCC1	0.305	5.345E-04	4.680E-03	673.183
CYLD	0.306	3.165E-04	3.025E-03	4345.740
KLK1	0.308	7.291E-03	3.851E-02	413.244
RSRP1	0.308	7.280E-04	6.059E-03	1622.285
TYK2	0.308	2.422E-04	2.412E-03	10124.556
POLD4	0.308	2.436E-04	2.422E-03	3570.140
PRKX	0.309	8.168E-04	6.644E-03	1472.136
SRGN	0.309	6.598E-03	3.555E-02	10778.808
BOD1L1	0.309	4.391E-04	3.964E-03	3196.281
ALG10B	0.309	6.575E-03	3.546E-02	314.389
HLA-DQA2	0.309	6.798E-04	5.724E-03	1582.387
TCP11L1	0.309	1.438E-03	1.050E-02	579.972
ERAP2	0.309	4.700E-05	5.956E-04	2155.217
MCTP2	0.310	2.246E-03	1.510E-02	540.453
TAP1	0.310	7.323E-06	1.195E-04	13871.947
XRN1	0.310	6.914E-05	8.329E-04	1476.665
ZNF490	0.310	5.928E-03	3.251E-02	338.748
ASAP1	0.311	1.144E-03	8.728E-03	1079.887
STK10	0.311	2.774E-04	2.708E-03	7151.639
RCSD1	0.312	3.123E-05	4.226E-04	3782.573
ZNF513	0.312	1.801E-03	1.265E-02	741.129
SLC43A2	0.312	8.524E-04	6.879E-03	1561.858
PIK3CG	0.312	5.765E-03	3.174E-02	646.860
RIC1	0.312	1.785E-05	2.594E-04	2396.945
ZNF277	0.313	4.281E-03	2.515E-02	503.443
ANKRD33B	0.314	6.280E-04	5.350E-03	6713.164
KIAA0556	0.314	4.066E-04	3.719E-03	1248.687
HP1BP3	0.314	4.093E-05	5.297E-04	6022.498
MYO6	0.315	1.439E-05	2.163E-04	2381.113
VPS13C	0.315	2.316E-05	3.241E-04	3136.444
TRIM35	0.315	1.983E-04	2.038E-03	1003.209
MICALL1	0.315	7.950E-05	9.426E-04	1367.229
SOX4	0.315	5.307E-03	2.969E-02	505.482
VPS41	0.315	6.186E-05	7.579E-04	1397.608
DNAH1	0.316	3.717E-03	2.245E-02	910.987
CLIP4	0.316	2.815E-04	2.737E-03	581.598
B4GAT1	0.316	9.026E-03	4.555E-02	244.110
ZNF251	0.316	4.538E-03	2.621E-02	343.301
ZNF789	0.316	9.924E-03	4.883E-02	274.530
TMEM140	0.316	3.610E-03	2.199E-02	997.582

DCBLD2	0.317	8.643E-03	4.411E-02	270.883
ZFYVE16	0.318	2.856E-03	1.833E-02	368.358
LILRB1	0.319	1.549E-03	1.115E-02	3784.491
INPP4A	0.319	3.300E-05	4.418E-04	2339.981
CUL9	0.319	4.973E-04	4.395E-03	2301.873
LINC00174	0.320	9.891E-03	4.872E-02	417.987
MTRNR2L9	0.321	4.570E-04	4.101E-03	2916.354
CCNG2	0.321	1.006E-02	4.941E-02	357.151
CD86	0.321	2.123E-04	2.164E-03	1559.811
ZSCAN2	0.321	5.025E-03	2.845E-02	301.512
RB1	0.321	6.684E-05	8.107E-04	2313.816
ID3	0.321	1.546E-03	1.113E-02	1899.474
DYNC1LI2	0.322	3.390E-05	4.515E-04	1613.492
NOTCH2NL	0.322	9.485E-03	4.724E-02	217.501
APOL1	0.322	7.146E-04	5.970E-03	1441.212
CCL5	0.323	2.713E-03	1.762E-02	784.953
RPL23AP53	0.324	4.303E-03	2.522E-02	302.494
PTBP3	0.324	3.060E-05	4.153E-04	3116.412
KCNN4	0.324	2.237E-03	1.506E-02	1852.316
PCMTD1	0.324	4.181E-03	2.466E-02	568.285
LOC389906	0.324	2.022E-04	2.070E-03	781.307
DUSP14	0.324	4.398E-03	2.564E-02	282.240
CLSTN3	0.325	4.068E-04	3.719E-03	2430.650
S1PR2	0.325	1.517E-03	1.096E-02	896.696
PILRA	0.325	8.937E-03	4.521E-02	226.438
NEU1	0.325	3.498E-05	4.634E-04	1147.927
NICN1	0.325	9.080E-04	7.234E-03	548.542
MYO1G	0.325	3.299E-05	4.418E-04	10598.783
BBS2	0.325	6.623E-03	3.567E-02	407.310
TRAFD1	0.325	4.149E-06	7.249E-05	3715.303
PLCL2	0.325	1.019E-05	1.606E-04	1184.302
C1RL	0.326	1.150E-03	8.773E-03	575.387
TESPA1	0.326	5.838E-05	7.205E-04	1298.927
GPATCH2L	0.327	4.021E-05	5.213E-04	1681.339
HLA-DMA	0.327	1.897E-05	2.728E-04	4217.388
SUV420H1	0.327	4.717E-04	4.203E-03	512.021
CREBRF	0.328	5.233E-03	2.935E-02	362.983
DGKZ	0.328	4.342E-05	5.568E-04	3558.761
ZNF550	0.328	2.478E-03	1.637E-02	361.047
HCG26	0.328	7.883E-03	4.102E-02	313.181
DTX2	0.328	1.000E-03	7.807E-03	972.490
ZNF780B	0.329	2.290E-03	1.534E-02	375.754
PGPEP1	0.329	1.277E-04	1.411E-03	2643.159
MYO5A	0.329	9.176E-06	1.464E-04	3573.741
FLYWCH1	0.329	3.911E-04	3.603E-03	1682.941
C10orf128	0.329	1.691E-03	1.198E-02	485.872
GLMP	0.329	7.133E-04	5.963E-03	591.486
IL1R1	0.330	3.325E-03	2.068E-02	586.378

TRPV2	0.330	6.299E-05	7.698E-04	4089.960
ZNF211	0.330	8.107E-04	6.618E-03	399.798
IFI44L	0.330	3.059E-05	4.153E-04	10028.209
ICAM3	0.330	1.719E-04	1.813E-03	4351.409
FLJ42627	0.331	3.821E-03	2.296E-02	367.781
DZIP3	0.331	2.241E-03	1.507E-02	585.956
CMTM6	0.332	1.250E-04	1.386E-03	1488.431
LINC00996	0.332	9.426E-03	4.702E-02	706.820
NIPAL3	0.333	1.763E-04	1.851E-03	843.684
UHMK1	0.333	3.435E-04	3.228E-03	2523.851
ITGAV	0.334	3.578E-04	3.331E-03	516.502
ISCU	0.334	2.618E-06	4.873E-05	2771.491
LIMK2	0.335	9.868E-05	1.133E-03	1345.584
LPIN1	0.335	1.537E-04	1.658E-03	2599.049
TMEM127	0.336	7.229E-06	1.182E-04	2455.709
DHRS1	0.337	3.350E-03	2.078E-02	693.850
ARL6IP1	0.337	2.265E-06	4.295E-05	2235.808
TMEM198B	0.337	1.010E-02	4.956E-02	995.148
BMP2K	0.338	3.479E-04	3.264E-03	1120.987
PNOC	0.339	1.989E-03	1.370E-02	523.980
DNAJB5	0.339	1.214E-04	1.352E-03	586.615
LOC100129434	0.339	3.466E-03	2.136E-02	344.129
MTRNR2L2	0.339	1.510E-04	1.633E-03	2799.808
PAK1	0.339	1.946E-06	3.745E-05	3583.691
ACADVL	0.340	1.447E-04	1.570E-03	6280.604
RIPK3	0.340	9.768E-04	7.646E-03	393.001
MAPK8IP3	0.340	3.740E-04	3.468E-03	3689.172
NLRC5	0.340	1.561E-06	3.063E-05	7420.912
ATAT1	0.341	7.792E-03	4.067E-02	300.759
TNFSF10	0.341	4.963E-03	2.820E-02	322.397
ACSF2	0.341	5.420E-03	3.020E-02	479.088
NPNT	0.341	3.500E-03	2.151E-02	522.215
MARCH8	0.341	1.750E-05	2.546E-04	1410.274
BHLHE40	0.342	2.997E-05	4.081E-04	4179.643
PSMA3-AS1	0.342	2.051E-03	1.404E-02	520.006
STAT2	0.343	1.994E-06	3.834E-05	6959.638
TMEM154	0.343	4.437E-03	2.578E-02	398.652
IL4R	0.343	4.570E-04	4.101E-03	2164.014
IFT80	0.343	1.441E-03	1.052E-02	456.885
TNFAIP3	0.344	1.818E-04	1.897E-03	12815.329
KLHL24	0.344	6.425E-03	3.477E-02	290.573
ECHDC2	0.344	2.756E-03	1.786E-02	518.506
ZNF786	0.344	1.900E-03	1.321E-02	323.354
SRC	0.344	6.824E-06	1.123E-04	4480.362
TOM1L2	0.344	8.508E-05	9.971E-04	1313.864
TSPAN14	0.345	8.829E-04	7.071E-03	1247.853
DCAKD	0.345	1.976E-04	2.033E-03	1548.578
LMO4	0.345	1.805E-04	1.884E-03	913.919

AP1S3	0.346	4.041E-03	2.405E-02	498.233
TNFRSF14	0.346	3.600E-03	2.195E-02	3464.540
LDB1	0.346	7.295E-05	8.746E-04	2235.949
PPIP5K1	0.347	6.096E-06	1.015E-04	1526.658
SIPA1L3	0.347	2.177E-04	2.207E-03	4989.684
KCTD21	0.348	5.252E-04	4.611E-03	473.690
PTK2B	0.348	1.747E-05	2.546E-04	14710.552
P2RY8	0.348	2.492E-04	2.467E-03	4980.402
ZNF358	0.348	1.012E-02	4.962E-02	275.196
RABL2B	0.349	1.409E-03	1.035E-02	337.417
FES	0.349	6.995E-03	3.730E-02	342.898
HIP1R	0.349	9.252E-05	1.073E-03	970.024
SLFN5	0.350	2.723E-04	2.672E-03	2387.897
PARP14	0.350	1.880E-07	4.574E-06	9916.146
MAN2B1	0.350	2.157E-05	3.045E-04	6368.088
FAM214A	0.351	8.405E-05	9.880E-04	678.768
BAZ2B	0.351	8.592E-04	6.919E-03	618.069
PLCG1	0.351	4.430E-06	7.668E-05	3325.912
RGL1	0.351	8.334E-05	9.812E-04	623.352
RHOB	0.352	3.862E-03	2.314E-02	337.071
STOM	0.352	8.712E-05	1.017E-03	819.747
ATXN3	0.353	7.789E-04	6.402E-03	378.511
MTRNR2L8	0.353	1.658E-04	1.757E-03	2939.659
DYRK1B	0.354	2.084E-03	1.423E-02	449.709
CTSO	0.354	3.239E-03	2.023E-02	256.599
ITGB1	0.355	1.025E-04	1.168E-03	1118.268
SAMD4A	0.355	5.464E-03	3.038E-02	307.776
ZNF564	0.357	4.360E-03	2.550E-02	198.889
DESI2	0.357	2.760E-04	2.702E-03	595.682
LCP2	0.357	9.825E-04	7.686E-03	378.537
SRA1	0.358	3.030E-06	5.536E-05	1666.457
ZNF254	0.358	7.323E-03	3.866E-02	189.624
MXD1	0.359	6.081E-04	5.219E-03	515.837
LINC01410	0.359	5.444E-03	3.029E-02	280.612
ZNF83	0.360	2.956E-03	1.877E-02	341.412
STX6	0.360	3.732E-07	8.467E-06	1572.504
ZNF737	0.360	2.699E-03	1.755E-02	239.625
TRAF4	0.361	3.592E-05	4.727E-04	5031.200
FBXW7	0.361	7.565E-06	1.232E-04	1586.875
FBF1	0.361	8.538E-04	6.886E-03	504.322
TRIP6	0.361	1.817E-05	2.631E-04	827.194
TSPAN33	0.362	1.798E-06	3.492E-05	5248.319
ZBTB4	0.362	2.822E-05	3.863E-04	2648.859
ERO1B	0.362	1.040E-03	8.079E-03	378.465
APOL6	0.362	1.511E-03	1.093E-02	1331.235
CORO1B	0.363	3.217E-05	4.338E-04	1305.290
DOK4	0.363	9.338E-03	4.671E-02	456.609
CRYBG3	0.363	3.712E-03	2.244E-02	282.064

PCDHGC3	0.364	1.111E-03	8.528E-03	514.143
PXN	0.364	2.204E-03	1.486E-02	1189.066
BCAS1	0.364	2.059E-03	1.409E-02	415.246
KAT2B	0.364	3.998E-05	5.193E-04	628.040
CRTC1	0.367	9.181E-04	7.299E-03	363.097
LINC00674	0.368	7.682E-04	6.341E-03	353.840
SOX9	0.368	8.078E-05	9.564E-04	669.987
PLEKHM1P	0.369	9.977E-04	7.793E-03	449.850
ADAMTS13	0.369	9.664E-03	4.789E-02	232.465
WHAMM	0.370	1.027E-04	1.169E-03	760.792
EVL	0.370	2.188E-05	3.084E-04	2505.771
ATP6AP2	0.371	1.725E-06	3.363E-05	1296.740
ZNF862	0.371	2.180E-04	2.207E-03	875.681
PON2	0.371	6.247E-03	3.394E-02	221.702
POU2F2	0.371	1.884E-04	1.953E-03	6113.828
ZBTB26	0.372	8.983E-03	4.539E-02	164.674
SPATA13	0.372	4.583E-05	5.837E-04	1018.744
VPS37B	0.372	1.641E-05	2.416E-04	1240.936
TBC1D10A	0.373	5.457E-05	6.794E-04	780.087
DDX58	0.373	3.809E-05	4.981E-04	606.247
LMBRD2	0.373	5.170E-03	2.905E-02	222.515
RFX7	0.374	8.325E-07	1.762E-05	1131.456
SPIN3	0.374	1.172E-03	8.915E-03	379.800
DGKA	0.375	3.318E-04	3.146E-03	906.928
FMNL3	0.376	2.587E-05	3.571E-04	7190.665
SAMD9L	0.376	3.697E-07	8.399E-06	3555.663
FSCN1	0.377	1.861E-04	1.935E-03	13574.310
SPI1	0.377	1.636E-03	1.168E-02	795.543
AHR	0.377	2.351E-04	2.353E-03	960.690
CD80	0.377	5.287E-05	6.622E-04	1539.208
PMAIP1	0.378	4.784E-05	6.057E-04	2342.081
ZNF608	0.378	8.239E-04	6.694E-03	694.617
SLC35E3	0.378	6.431E-04	5.460E-03	367.477
GIMAP8	0.378	8.885E-04	7.108E-03	445.387
MAFIP	0.379	7.858E-03	4.093E-02	248.539
SUSD6	0.379	1.554E-07	3.917E-06	3568.043
ITPRIPL2	0.379	8.588E-04	6.919E-03	276.864
FGD2	0.380	2.191E-03	1.481E-02	3140.563
CTSZ	0.380	1.033E-04	1.172E-03	5992.484
CD70	0.380	2.211E-07	5.302E-06	5241.085
ZNF767P	0.380	4.633E-05	5.887E-04	972.938
GAS7	0.381	3.169E-06	5.745E-05	2619.263
LINC00342	0.381	1.515E-03	1.096E-02	581.584
ZFAND4	0.381	9.173E-04	7.299E-03	236.505
SMIM3	0.381	2.711E-04	2.664E-03	465.644
CPNE8	0.381	2.635E-04	2.599E-03	813.520
MARCH2	0.381	1.360E-04	1.494E-03	432.302
PRKXP1	0.382	1.790E-04	1.872E-03	367.787

LOC401320	0.382	1.943E-03	1.346E-02	405.076
SLC45A4	0.382	6.508E-04	5.519E-03	290.345
KMO	0.382	7.795E-03	4.067E-02	145.779
LOC100049716	0.383	7.475E-04	6.194E-03	274.591
GOLIM4	0.383	6.350E-07	1.372E-05	1348.155
PBXIP1	0.383	3.068E-04	2.946E-03	2629.031
SH3BP5-AS1	0.383	2.910E-03	1.857E-02	211.304
REV3L	0.383	7.851E-06	1.273E-04	1355.366
SYNJ2BP	0.383	8.431E-05	9.896E-04	516.055
FCHSD1	0.383	4.930E-04	4.362E-03	1176.961
SETBP1	0.384	3.485E-03	2.145E-02	339.699
ATM	0.384	4.022E-06	7.068E-05	2665.180
PLEKHO2	0.384	6.830E-05	8.247E-04	2908.926
ITIH4	0.384	2.662E-03	1.737E-02	327.936
N4BP2L1	0.385	1.442E-04	1.565E-03	381.716
TTN-AS1	0.385	1.760E-04	1.849E-03	431.671
GALNS	0.385	2.532E-04	2.506E-03	372.332
PARP15	0.386	3.313E-04	3.145E-03	2201.702
CECR1	0.386	1.651E-04	1.752E-03	1198.530
ZNF772	0.386	3.149E-04	3.016E-03	366.468
TGFBR1	0.386	3.888E-04	3.589E-03	397.121
HLA-DPB1	0.386	1.975E-07	4.773E-06	6317.324
PVRL1	0.386	5.501E-03	3.053E-02	1009.543
HLA-DRB6	0.386	7.397E-04	6.142E-03	463.247
C14orf105	0.387	5.625E-03	3.111E-02	312.854
KLHL6	0.388	2.808E-05	3.848E-04	1204.176
HGSNAT	0.388	1.782E-06	3.466E-05	1277.187
CSRNP2	0.388	1.598E-05	2.361E-04	517.337
EPSTI1	0.388	6.207E-06	1.030E-04	1342.075
ZSCAN30	0.388	1.805E-05	2.616E-04	792.044
CSAD	0.388	6.715E-04	5.673E-03	599.722
TBCEL	0.389	5.791E-05	7.164E-04	416.516
FAM13B	0.389	3.180E-06	5.748E-05	1076.144
NEDD9	0.389	1.540E-05	2.293E-04	1852.122
FIG4	0.390	1.822E-05	2.633E-04	680.964
RIMS3	0.391	6.413E-03	3.472E-02	362.394
ZC3H12D	0.392	5.851E-05	7.209E-04	1544.913
PMS2P3	0.392	1.123E-03	8.609E-03	242.060
NFAM1	0.393	4.429E-03	2.578E-02	222.797
SLC5A3	0.393	4.870E-07	1.076E-05	1522.975
ERCC6	0.393	1.379E-04	1.509E-03	451.717
TNFRSF21	0.394	5.705E-04	4.964E-03	378.168
PARVG	0.394	1.886E-05	2.717E-04	1529.606
LHX4-AS1	0.394	1.212E-03	9.168E-03	291.492
BLCAP	0.394	1.229E-07	3.170E-06	1443.349
GCC2	0.394	1.031E-04	1.171E-03	1085.148
P2RX7	0.395	1.517E-03	1.096E-02	242.365
ERI2	0.395	8.114E-04	6.618E-03	255.835

IFIT1	0.396	6.123E-04	5.249E-03	1743.100
PLIN2	0.397	1.655E-07	4.119E-06	1321.624
NDFIP1	0.398	5.028E-04	4.436E-03	263.831
BTBD10	0.398	4.236E-06	7.384E-05	701.191
ST3GAL5	0.398	3.627E-03	2.206E-02	229.465
SORD	0.399	1.026E-04	1.168E-03	1298.172
SAT1	0.399	1.295E-04	1.430E-03	1596.482
LOC155060	0.400	5.332E-03	2.978E-02	259.394
AKT1S1	0.400	5.642E-04	4.918E-03	1755.340
L3HYPDH	0.400	5.613E-04	4.895E-03	248.432
MAP2K6	0.400	5.028E-03	2.845E-02	281.496
LMO2	0.400	1.154E-03	8.793E-03	516.504
ZNF852	0.401	7.454E-03	3.921E-02	126.746
RBL2	0.401	4.035E-08	1.172E-06	1958.753
LYRM1	0.401	2.946E-04	2.843E-03	457.068
SP140L	0.401	2.139E-07	5.145E-06	1151.043
TMEM63B	0.402	1.229E-04	1.366E-03	1033.650
SHISA5	0.403	1.013E-04	1.158E-03	2676.913
SNX13	0.403	5.044E-05	6.365E-04	443.241
CD82	0.403	4.049E-06	7.108E-05	8422.708
KLHL3	0.403	1.437E-04	1.561E-03	715.322
PDE1B	0.404	1.566E-04	1.681E-03	313.022
HCK	0.404	4.450E-04	4.010E-03	1437.124
PLEK	0.404	2.533E-05	3.500E-04	11811.727
ZSWIM8	0.404	8.501E-07	1.794E-05	4433.093
PTP4A1	0.404	1.561E-07	3.923E-06	2045.341
APOL2	0.405	2.118E-05	2.998E-04	1498.843
BBS1	0.405	2.323E-05	3.248E-04	547.988
EFHC1	0.406	3.880E-03	2.321E-02	227.119
DUSP4	0.406	2.226E-05	3.127E-04	5746.499
UGDH-AS1	0.407	5.263E-04	4.619E-03	433.719
ABCB4	0.407	1.626E-03	1.162E-02	208.956
LAPTM5	0.407	1.243E-09	5.204E-08	36771.570
ARID4A	0.407	1.354E-04	1.488E-03	629.205
SLC35F5	0.408	3.941E-04	3.629E-03	307.378
CD53	0.408	1.460E-08	4.729E-07	8169.763
DNASE1	0.410	6.150E-03	3.352E-02	325.001
YPEL5	0.410	3.210E-05	4.332E-04	742.671
MAPK13	0.412	1.884E-03	1.313E-02	291.518
SNX30	0.413	1.353E-03	1.001E-02	407.404
ARMCX2	0.413	5.914E-04	5.107E-03	366.370
TMEM8B	0.413	6.092E-04	5.225E-03	571.992
PANK1	0.415	2.311E-04	2.319E-03	299.982
TCF7	0.415	3.318E-05	4.435E-04	2829.370
ZNF81	0.416	2.378E-05	3.312E-04	400.386
CCDC159	0.416	2.151E-04	2.186E-03	309.817
COL19A1	0.417	1.346E-03	9.969E-03	601.443
SH3BGRL	0.417	3.430E-07	7.840E-06	2027.893

AMPD3	0.417	5.590E-06	9.366E-05	1582.186
MAN2B2	0.417	1.514E-06	2.991E-05	1277.790
ABCA5	0.418	9.614E-04	7.564E-03	570.314
CCDC186	0.418	1.437E-03	1.050E-02	424.598
SGPP1	0.419	8.870E-06	1.420E-04	1094.915
NAPSB	0.419	7.694E-04	6.347E-03	2490.473
CIPC	0.420	2.741E-06	5.067E-05	821.877
DUSP5	0.420	3.688E-08	1.085E-06	3403.827
DMD	0.420	8.035E-07	1.707E-05	1106.458
C3orf62	0.420	1.680E-03	1.192E-02	208.520
TRIM38	0.421	1.892E-07	4.595E-06	2094.383
BTG1	0.421	1.955E-08	6.176E-07	4346.950
ERVK13-1	0.422	8.593E-06	1.379E-04	683.463
PIGZ	0.422	7.121E-03	3.782E-02	148.380
TAB3	0.422	4.286E-07	9.606E-06	795.339
SIDT2	0.422	3.050E-05	4.146E-04	1694.395
ARL6IP5	0.423	7.586E-07	1.619E-05	2660.598
CAPS	0.423	1.044E-03	8.103E-03	440.544
ZNF28	0.424	4.608E-04	4.125E-03	238.965
LAMC1	0.424	4.931E-06	8.430E-05	2318.935
TP53INP2	0.424	2.873E-04	2.781E-03	754.810
VMAC	0.424	3.467E-03	2.136E-02	229.251
GCH1	0.424	1.187E-06	2.404E-05	808.507
RPS6KA1	0.425	5.564E-07	1.217E-05	5014.131
HERC5	0.426	5.106E-06	8.628E-05	1688.944
GPR132	0.427	2.208E-06	4.192E-05	2566.739
GNGT2	0.428	1.527E-05	2.276E-04	584.597
C11orf24	0.428	2.227E-07	5.324E-06	1813.981
RBM41	0.428	2.729E-04	2.675E-03	477.847
ITM2A	0.428	2.328E-05	3.250E-04	442.391
DUSP18	0.428	1.952E-04	2.012E-03	242.626
LTB4R2	0.429	4.448E-03	2.581E-02	135.017
MRC2	0.429	7.727E-03	4.038E-02	252.147
GATSL3	0.430	4.017E-03	2.393E-02	177.489
ZEB2	0.431	8.331E-03	4.292E-02	163.659
TMEM68	0.431	1.375E-04	1.508E-03	313.280
TMEM2	0.432	1.999E-04	2.050E-03	643.168
LOC102724814	0.432	3.491E-04	3.273E-03	423.983
FGD3	0.433	3.029E-04	2.915E-03	1040.279
NFAT5	0.433	5.790E-06	9.658E-05	3215.223
MLF2	0.433	7.987E-09	2.765E-07	4415.372
ZNF585B	0.435	2.243E-04	2.259E-03	246.995
ZBED1	0.435	8.030E-09	2.768E-07	3614.337
KIAA1549L	0.436	9.075E-05	1.055E-03	568.735
CNKSR3	0.436	9.396E-04	7.443E-03	249.173
SLFN12	0.436	2.034E-04	2.081E-03	251.573
BCL2L1	0.437	4.820E-06	8.259E-05	3351.156
GPR15	0.438	1.556E-06	3.060E-05	1564.099

LINC00926	0.438	2.859E-04	2.771E-03	1347.970
LTBP3	0.438	2.690E-03	1.751E-02	198.294
CLIP3	0.438	3.009E-03	1.903E-02	221.330
TCAIM	0.438	6.737E-05	8.148E-04	414.357
WDFY1	0.438	1.868E-07	4.551E-06	1469.977
RAP2B	0.439	2.718E-08	8.226E-07	980.603
DOK3	0.439	2.427E-04	2.415E-03	3014.533
IPCEF1	0.440	1.734E-05	2.535E-04	1927.484
ASCC3	0.441	1.041E-07	2.755E-06	2792.996
C21orf91	0.442	7.683E-05	9.154E-04	524.814
RAPGEFL1	0.443	1.545E-04	1.662E-03	265.966
STAT5A	0.443	1.968E-05	2.817E-04	5739.536
TPP1	0.444	4.933E-09	1.803E-07	3378.748
NLRC3	0.444	1.052E-03	8.159E-03	1276.101
NABP1	0.446	5.800E-05	7.169E-04	743.983
GNA15	0.446	9.154E-06	1.463E-04	839.104
ALPK1	0.446	5.115E-08	1.441E-06	1180.052
REC8	0.447	4.054E-06	7.108E-05	2826.125
CSNK1G1	0.447	1.020E-06	2.115E-05	1685.831
JADE2	0.448	2.904E-08	8.726E-07	4387.564
ABCC5	0.448	1.379E-05	2.091E-04	599.884
SH3BP2	0.449	4.131E-07	9.315E-06	3748.392
ZSCAN18	0.449	3.147E-05	4.251E-04	375.849
ALPK2	0.451	9.065E-04	7.226E-03	272.964
NUB1	0.451	4.552E-08	1.297E-06	1945.864
DOPEY1	0.452	1.294E-07	3.317E-06	816.640
ATG16L2	0.452	1.540E-04	1.659E-03	1598.748
KDM5B	0.452	3.125E-06	5.682E-05	558.672
COL9A2	0.452	5.452E-05	6.794E-04	3212.628
RHOC	0.453	1.074E-07	2.826E-06	3252.817
IFIT2	0.453	3.263E-05	4.383E-04	1621.172
SORL1	0.454	5.583E-08	1.558E-06	15648.702
HLA-DMB	0.455	1.292E-06	2.595E-05	3373.847
SERAC1	0.455	9.646E-03	4.783E-02	84.723
DDIT4	0.456	1.001E-04	1.145E-03	6586.537
YJEFN3	0.456	8.906E-03	4.512E-02	148.622
TNRC6C-AS1	0.456	3.703E-06	6.591E-05	1656.360
GABARAPL1	0.456	4.668E-04	4.170E-03	199.861
ZNF107	0.456	1.880E-05	2.712E-04	970.736
USP35	0.458	4.226E-05	5.442E-04	573.599
CD180	0.458	2.556E-03	1.678E-02	271.654
RGS20	0.459	3.465E-03	2.136E-02	226.567
KIAA1407	0.459	1.351E-03	9.993E-03	147.584
BIRC3	0.460	1.895E-04	1.963E-03	3255.255
CCDC92	0.460	3.593E-05	4.727E-04	520.328
TOB2P1	0.460	6.562E-03	3.543E-02	96.750
DCP1B	0.460	2.706E-06	5.017E-05	461.871
ZNF530	0.462	8.126E-04	6.621E-03	176.898

CPEB4	0.463	1.161E-05	1.798E-04	1285.003
TUBB6	0.464	3.013E-08	8.974E-07	1500.756
DTX1	0.464	7.865E-05	9.340E-04	311.582
ZNF532	0.464	2.133E-03	1.449E-02	150.375
CCDC150	0.465	3.860E-04	3.568E-03	205.013
RAB13	0.466	4.984E-09	1.817E-07	1513.638
LINC01268	0.466	2.271E-03	1.524E-02	172.369
FLVCR2	0.466	7.701E-04	6.348E-03	171.509
SPSB1	0.466	2.337E-03	1.558E-02	168.145
LIPA	0.466	1.045E-08	3.514E-07	1735.304
CCSER2	0.467	3.142E-06	5.708E-05	559.487
ARSD	0.467	6.741E-04	5.679E-03	236.736
PPP1R3B	0.467	9.481E-05	1.097E-03	241.734
RAB38	0.467	3.233E-05	4.351E-04	337.387
AHDC1	0.468	9.285E-07	1.935E-05	1474.474
TCTA	0.469	2.410E-07	5.715E-06	759.195
LPPR2	0.469	5.838E-03	3.211E-02	149.115
EI24	0.471	2.894E-10	1.331E-08	1635.009
HLA-DQA1	0.471	4.339E-05	5.568E-04	4189.832
ACCS	0.471	2.354E-04	2.354E-03	575.987
APAF1	0.472	2.538E-08	7.712E-07	1152.474
ANKRD24	0.472	1.140E-03	8.721E-03	188.493
STARD9	0.472	1.408E-07	3.573E-06	1601.300
PNKD	0.472	3.705E-08	1.088E-06	1796.366
SUSD1	0.473	2.928E-05	4.002E-04	341.674
RALGPS2	0.473	5.573E-04	4.866E-03	200.398
PABPC1L	0.473	6.726E-05	8.141E-04	967.677
RILP	0.474	9.074E-03	4.572E-02	96.244
TNFSF9	0.475	9.386E-07	1.953E-05	2381.473
SERPINB1	0.476	1.194E-05	1.834E-04	503.756
WDR5B	0.476	1.361E-03	1.005E-02	143.547
MXD4	0.476	2.800E-06	5.167E-05	1363.030
GAK	0.477	1.245E-09	5.204E-08	5286.651
SCARF1	0.478	4.386E-03	2.561E-02	173.120
TMEM131	0.478	2.297E-09	8.970E-08	3316.383
CADM1	0.479	4.586E-04	4.113E-03	313.770
GRAMD3	0.479	1.073E-06	2.207E-05	532.300
NINJ1	0.480	2.669E-06	4.960E-05	725.742
IZUMO4	0.480	4.000E-04	3.670E-03	614.238
ZSWIM6	0.481	3.980E-04	3.660E-03	237.487
ZNF654	0.482	1.481E-05	2.214E-04	300.904
ARSA	0.482	3.425E-06	6.133E-05	1492.069
SIAE	0.482	1.618E-05	2.385E-04	361.923
ZIK1	0.482	2.776E-04	2.708E-03	207.054
FLT1	0.482	6.155E-05	7.546E-04	314.781
FLJ32255	0.483	1.383E-05	2.095E-04	324.099
ZFYVE1	0.483	1.073E-08	3.593E-07	1241.516
CCR1	0.483	2.086E-03	1.424E-02	187.621

SERINC5	0.483	7.703E-04	6.348E-03	246.667
GOLGA8B	0.484	6.668E-05	8.097E-04	854.500
PAQR7	0.484	4.901E-04	4.342E-03	186.561
LOC100652768	0.485	3.039E-03	1.920E-02	107.043
PHC1	0.485	1.201E-06	2.430E-05	890.150
RPL23AP7	0.485	6.393E-04	5.434E-03	198.957
CTNNBIP1	0.486	5.110E-06	8.628E-05	428.458
BAX	0.487	1.437E-12	1.011E-10	4281.769
TNFRSF1A	0.488	5.460E-05	6.794E-04	437.661
BASP1	0.488	1.923E-06	3.712E-05	3294.484
ZNF331	0.488	1.709E-05	2.501E-04	449.612
ARL11	0.489	1.389E-04	1.518E-03	224.353
LINC00886	0.489	1.412E-03	1.036E-02	133.741
CST3	0.491	3.634E-08	1.072E-06	1040.643
PPP4R1L	0.492	2.241E-04	2.259E-03	223.257
F11R	0.492	4.773E-10	2.124E-08	2379.072
PLA2G15	0.492	3.741E-07	8.473E-06	610.022
NSF	0.493	3.304E-10	1.506E-08	1571.274
PHLDA1	0.494	1.401E-03	1.030E-02	148.498
ABHD4	0.494	1.106E-06	2.263E-05	1105.978
LATS2	0.495	8.138E-06	1.311E-04	435.013
DSE	0.496	5.694E-08	1.581E-06	1952.864
RABL2A	0.496	9.476E-04	7.494E-03	160.850
PPFIBP1	0.497	1.202E-10	6.083E-09	1287.343
MAL	0.497	7.567E-05	9.030E-04	220.392
CFAP44	0.497	9.307E-05	1.078E-03	220.612
KIF26B	0.498	3.396E-03	2.103E-02	376.861
TLR1	0.498	3.218E-04	3.064E-03	242.434
CIITA	0.498	5.154E-08	1.449E-06	4089.477
ASB16-AS1	0.499	3.359E-06	6.022E-05	548.700
MIB2	0.499	4.389E-06	7.616E-05	1027.477
FSTL3	0.499	4.004E-03	2.387E-02	174.590
CCDC15	0.501	3.310E-03	2.061E-02	109.008
MYH10	0.502	2.171E-10	1.020E-08	2491.382
ZC3H7A	0.502	7.049E-11	3.729E-09	2587.767
PDGFA	0.502	1.096E-06	2.247E-05	413.453
NOTCH2	0.502	1.516E-04	1.638E-03	915.022
GPR137B	0.503	3.746E-09	1.410E-07	860.630
ZBTB46	0.503	1.105E-03	8.488E-03	250.849
HRAS	0.503	1.126E-08	3.743E-07	699.692
WBP1	0.503	7.737E-03	4.042E-02	127.653
ZNFX1	0.504	1.574E-11	9.340E-10	6218.982
SOGA1	0.504	5.064E-05	6.379E-04	1979.841
VNN2	0.505	2.006E-07	4.841E-06	499.726
IKBIP	0.505	3.751E-06	6.661E-05	469.047
ATP7A	0.505	6.075E-08	1.671E-06	885.641
MIR22HG	0.505	2.806E-04	2.732E-03	285.380
YPEL2	0.505	1.753E-03	1.235E-02	151.791

DENND6B	0.506	7.026E-07	1.513E-05	564.628
KIAA1324L	0.507	4.383E-04	3.961E-03	207.484
EEA1	0.507	2.403E-06	4.521E-05	910.564
VIM	0.508	5.730E-10	2.513E-08	18304.656
OTUD1	0.508	3.451E-03	2.128E-02	110.111
RANBP10	0.509	1.281E-09	5.323E-08	1636.545
DTX2P1-UPK3BP1-PMS2P11	0.509	4.563E-03	2.632E-02	105.549
ZC3H12C	0.509	2.933E-07	6.804E-06	1836.942
PTAFR	0.510	2.250E-08	6.935E-07	1802.361
C12orf76	0.511	1.429E-03	1.047E-02	115.195
CALCOCO1	0.512	1.116E-07	2.921E-06	1572.372
RALGDS	0.512	3.794E-06	6.729E-05	2089.204
DTX3L	0.513	1.113E-13	9.906E-12	4051.558
RPS6KA5	0.513	1.568E-03	1.126E-02	110.680
CEP85L	0.513	2.325E-05	3.248E-04	395.013
ADCK3	0.513	1.556E-07	3.917E-06	680.757
PSAP	0.515	4.774E-13	3.704E-11	17950.517
LOC100294145	0.515	3.828E-08	1.118E-06	579.251
TRIM32	0.515	1.689E-09	6.813E-08	749.782
CD83	0.516	4.730E-06	8.115E-05	4387.924
IFI6	0.516	5.938E-07	1.291E-05	2384.659
LYST	0.516	5.817E-07	1.268E-05	752.124
GPC5	0.516	7.580E-04	6.267E-03	151.306
TBC1D9	0.516	1.910E-03	1.327E-02	268.237
COL11A2	0.518	1.756E-03	1.236E-02	121.408
ZNF547	0.518	5.042E-03	2.852E-02	78.660
CES4A	0.520	1.296E-03	9.661E-03	200.756
AHRR	0.520	1.722E-08	5.496E-07	3339.439
GALNT11	0.520	7.162E-06	1.173E-04	343.292
STARD4	0.520	6.324E-09	2.251E-07	1840.763
MICAL2	0.521	2.495E-03	1.646E-02	155.239
RETSAT	0.521	5.148E-10	2.264E-08	1527.753
SMAD5	0.521	7.341E-08	1.990E-06	659.494
SH3TC1	0.521	2.437E-06	4.580E-05	2104.524
SEC14L2	0.522	5.114E-03	2.882E-02	84.291
TAGLN	0.522	1.488E-03	1.081E-02	157.133
INPP4B	0.523	7.203E-04	6.008E-03	162.088
CROT	0.524	8.824E-06	1.414E-04	407.364
MAPRE3	0.524	3.971E-03	2.370E-02	78.742
GPR55	0.526	2.991E-09	1.148E-07	1003.310
GVINP1	0.526	8.844E-07	1.851E-05	933.160
MST1	0.526	3.499E-04	3.276E-03	311.773
SERTAD1	0.527	5.383E-07	1.179E-05	477.000
DZIP1	0.527	6.209E-03	3.378E-02	348.547
S100A11	0.528	2.645E-07	6.195E-06	1268.859
PDCD1LG2	0.528	2.235E-04	2.254E-03	186.663

ZNF425	0.529	2.518E-03	1.659E-02	93.838
MAD1L1	0.531	2.586E-11	1.488E-09	1852.149
OSBPL3	0.531	2.161E-09	8.560E-08	2075.869
GSTM2	0.533	3.846E-05	5.020E-04	308.821
FAM102A	0.533	3.075E-06	5.613E-05	3130.553
LINC01160	0.533	7.160E-03	3.797E-02	142.773
TRPV1	0.533	2.067E-05	2.940E-04	207.890
CLN8	0.534	8.753E-07	1.837E-05	622.653
ABTB2	0.534	1.359E-06	2.716E-05	718.772
ECE1	0.536	8.670E-09	2.954E-07	1921.149
ABCA2	0.537	5.602E-06	9.366E-05	700.981
ABAT	0.538	1.597E-05	2.361E-04	354.682
PLAUR	0.538	3.411E-03	2.110E-02	87.472
TMEM57	0.539	9.322E-12	5.780E-10	920.395
TANK	0.540	4.998E-06	8.516E-05	1575.864
ERV3-1	0.540	3.265E-05	4.383E-04	499.710
ZNF793	0.542	8.506E-03	4.363E-02	78.692
HLA-DOA	0.542	1.519E-11	9.082E-10	3634.863
GPR35	0.542	2.662E-03	1.737E-02	88.077
FRMD8	0.543	1.836E-13	1.570E-11	3476.864
APBB3	0.543	7.737E-06	1.257E-04	650.407
IER3	0.543	8.313E-03	4.286E-02	2131.704
TMEM30A	0.543	1.746E-11	1.028E-09	2608.606
SLC12A4	0.546	1.777E-10	8.616E-09	1334.508
BFSP2	0.547	3.815E-05	4.985E-04	295.719
HLA-DOB	0.549	1.678E-07	4.155E-06	538.418
KIAA0513	0.549	1.365E-04	1.497E-03	568.615
DENND5A	0.549	3.777E-08	1.107E-06	2824.877
PDE4B	0.550	7.806E-08	2.113E-06	393.737
BTG2	0.550	2.107E-10	9.933E-09	11473.493
NIM1K	0.550	4.162E-04	3.793E-03	138.316
LGALS9	0.550	9.503E-08	2.534E-06	6072.959
SYNM	0.551	5.443E-03	3.029E-02	119.709
NEU3	0.551	2.795E-08	8.439E-07	811.942
FAM109A	0.552	7.158E-04	5.977E-03	127.631
KCTD11	0.555	8.707E-08	2.332E-06	489.163
LOC100131564	0.555	1.685E-05	2.471E-04	841.182
TRANK1	0.556	1.437E-05	2.162E-04	2524.421
PVT1	0.556	1.789E-07	4.394E-06	606.629
ZNF846	0.556	1.986E-03	1.368E-02	82.216
ETHE1	0.558	3.448E-12	2.319E-10	1044.287
MCC	0.559	1.562E-04	1.678E-03	278.547
PRR5	0.559	6.636E-05	8.065E-04	244.807
LMO7	0.559	7.253E-07	1.552E-05	1476.149
ZNF506	0.560	4.675E-04	4.170E-03	193.577
STAC3	0.562	5.880E-04	5.083E-03	117.815
KCNC4	0.562	8.148E-04	6.632E-03	163.178
C17orf99	0.563	3.670E-03	2.228E-02	98.199

SLC41A1	0.564	2.023E-06	3.884E-05	508.162
TIE1	0.567	8.996E-03	4.543E-02	83.933
IER5	0.569	5.845E-15	6.408E-13	5374.437
IL12RB2	0.569	1.706E-03	1.206E-02	186.815
ITGA10	0.571	2.109E-03	1.435E-02	102.941
TIMP1	0.571	1.824E-07	4.465E-06	1721.961
NRBP2	0.572	6.870E-05	8.283E-04	257.907
KIR3DX1	0.573	9.417E-03	4.701E-02	67.996
PARP9	0.573	4.914E-16	5.996E-14	3197.610
GBP3	0.574	5.245E-06	8.846E-05	271.998
MYCBPAP	0.574	3.541E-03	2.169E-02	96.597
UPB1	0.574	3.600E-03	2.195E-02	88.779
PTGIR	0.575	7.240E-04	6.029E-03	147.430
DOCK6	0.575	1.916E-07	4.646E-06	548.848
NBPF1	0.576	5.031E-04	4.436E-03	98.049
LDLRAD4	0.576	5.759E-07	1.257E-05	742.382
NRCAM	0.576	2.427E-04	2.415E-03	203.344
ZNF93	0.579	2.137E-04	2.176E-03	170.515
BRMS1L	0.580	3.130E-07	7.208E-06	250.693
SLC35D2	0.581	5.867E-04	5.078E-03	135.832
TMEM217	0.582	4.709E-03	2.698E-02	65.974
SDC4	0.587	1.090E-03	8.410E-03	253.167
JADE3	0.588	6.134E-08	1.684E-06	834.227
PLAC8	0.588	1.289E-08	4.231E-07	1448.265
HHAT	0.588	3.430E-04	3.226E-03	194.426
RSPH3	0.589	1.175E-04	1.312E-03	120.921
IL4I1	0.590	1.654E-07	4.119E-06	3769.404
ITGB8	0.590	5.387E-05	6.734E-04	191.558
MYO5C	0.590	6.668E-03	3.586E-02	63.483
ANXA4	0.592	1.493E-15	1.778E-13	2128.238
ADGRD1	0.593	4.134E-04	3.770E-03	144.304
ENTPD2	0.595	6.734E-06	1.110E-04	1152.178
CTSB	0.596	1.538E-10	7.657E-09	1084.337
CCNG1	0.596	7.008E-16	8.483E-14	2230.117
CD9	0.597	2.189E-05	3.084E-04	289.109
LINC00969	0.599	2.144E-03	1.454E-02	71.502
LOC100128361	0.601	3.506E-03	2.154E-02	61.075
ZNF880	0.603	4.088E-03	2.426E-02	80.489
KAZN	0.603	7.972E-03	4.139E-02	67.690
LMNA	0.603	1.143E-14	1.226E-12	7233.472
HAAO	0.603	3.493E-07	7.972E-06	937.556
TYMSOS	0.604	7.770E-03	4.056E-02	55.171
ZFHX2	0.606	1.422E-05	2.145E-04	318.607
ITPKC	0.607	7.197E-09	2.538E-07	717.845
KRBA2	0.607	1.800E-03	1.264E-02	83.317
BLOC1S2	0.610	9.130E-11	4.731E-09	1097.991
MARVELD1	0.612	2.832E-03	1.820E-02	84.891
TNFRSF9	0.613	1.884E-04	1.953E-03	346.410

LINC01320	0.614	6.070E-03	3.317E-02	113.989
C10orf54	0.614	1.035E-07	2.743E-06	488.301
CCR7	0.615	2.639E-12	1.799E-10	15569.193
SLC6A4	0.616	1.458E-03	1.061E-02	170.895
AGRN	0.617	4.267E-09	1.587E-07	7081.180
MAPK11	0.617	2.937E-07	6.804E-06	616.351
TGM5	0.619	9.789E-03	4.837E-02	59.724
LOC257396	0.620	6.191E-04	5.295E-03	86.565
ZBP1	0.620	1.257E-04	1.392E-03	263.080
ANK1	0.620	1.643E-07	4.102E-06	675.019
SLC35D1	0.621	1.074E-13	9.672E-12	939.398
MIR3916	0.621	9.347E-04	7.408E-03	89.313
FZD6	0.622	8.012E-06	1.294E-04	283.305
UTRN	0.623	8.372E-09	2.872E-07	2438.901
FBXO22	0.623	2.091E-12	1.451E-10	1162.337
LOC729348	0.624	8.878E-04	7.107E-03	144.010
SCD5	0.624	4.845E-03	2.762E-02	69.832
APOBEC3G	0.625	1.811E-17	2.513E-15	3193.287
GDPD5	0.626	6.678E-08	1.824E-06	346.109
GRIK4	0.626	2.857E-03	1.833E-02	72.834
DTX4	0.627	6.212E-10	2.709E-08	4228.818
SLC27A1	0.627	5.302E-05	6.636E-04	196.644
SOCS3	0.629	2.562E-07	6.018E-06	321.773
FOXP1	0.629	4.717E-03	2.700E-02	87.128
TRPV3	0.630	1.690E-07	4.170E-06	476.716
HAPLN3	0.630	8.591E-05	1.005E-03	232.693
NATD1	0.631	6.534E-05	7.972E-04	188.831
NADSYN1	0.632	4.745E-12	3.081E-10	4019.065
CTNND1	0.633	4.815E-03	2.749E-02	70.109
ANKRD20A9P	0.633	2.144E-04	2.181E-03	106.521
CCDC90B	0.633	1.838E-09	7.339E-08	597.277
TMEM168	0.634	1.276E-09	5.319E-08	405.316
AAK1	0.637	4.793E-11	2.647E-09	2112.083
EDARADD	0.637	3.792E-04	3.512E-03	96.913
TIAM1	0.640	1.120E-06	2.285E-05	449.265
RASSF6	0.640	1.613E-03	1.154E-02	143.928
PRKY	0.643	8.008E-03	4.156E-02	155.968
PIK3IP1	0.644	5.332E-06	8.971E-05	252.222
ZBTB20	0.646	1.664E-07	4.135E-06	351.953
LRRK2	0.646	4.284E-08	1.232E-06	980.597
ICAM4	0.646	7.411E-03	3.903E-02	69.340
LINC00893	0.646	9.763E-04	7.645E-03	74.110
PTGER4	0.647	5.744E-04	4.992E-03	178.316
ITGB2-AS1	0.648	3.575E-07	8.146E-06	1179.411
XAF1	0.648	2.907E-08	8.726E-07	2460.740
TNFAIP2	0.648	2.200E-03	1.485E-02	2346.463
SAMD9	0.649	2.226E-09	8.725E-08	1555.791
CCP110	0.650	1.480E-10	7.391E-09	924.384

KIAA1217	0.650	1.452E-06	2.879E-05	1090.272
NCF2	0.651	2.458E-11	1.419E-09	3751.358
IFI27	0.651	1.664E-04	1.762E-03	159.008
TXK	0.652	1.433E-04	1.557E-03	107.927
ADAP2	0.652	1.607E-03	1.151E-02	65.348
FBXO44	0.652	1.052E-06	2.168E-05	596.147
POLH	0.652	1.431E-15	1.719E-13	1794.876
NEAT1	0.653	2.758E-11	1.575E-09	22819.621
DDX60	0.653	4.204E-07	9.437E-06	883.752
GM2A	0.654	7.035E-14	6.490E-12	4772.879
ACTA2	0.654	4.815E-10	2.136E-08	1749.679
KCTD1	0.656	5.082E-10	2.248E-08	400.395
ZNF229	0.656	7.218E-03	3.821E-02	51.364
CARD16	0.657	1.166E-03	8.880E-03	163.814
CORO2A	0.659	1.012E-02	4.962E-02	56.870
CAPN3	0.660	8.075E-07	1.713E-05	230.629
ITPR2	0.661	1.565E-15	1.840E-13	4695.206
C1orf228	0.661	3.171E-03	1.992E-02	79.427
ZFP90	0.661	3.268E-13	2.673E-11	682.114
CYB561A3	0.664	3.796E-18	5.419E-16	2934.804
ITGA1	0.665	2.575E-04	2.543E-03	151.976
ABCA10	0.666	1.899E-03	1.321E-02	58.535
CD44	0.667	1.369E-11	8.285E-10	10074.967
CD68	0.667	3.779E-05	4.955E-04	121.827
IL12RB1	0.669	1.546E-11	9.209E-10	999.554
MYL9	0.670	1.667E-03	1.185E-02	67.309
TLCD1	0.670	3.373E-05	4.500E-04	118.587
FILIP1L	0.671	8.394E-05	9.876E-04	152.334
GBP2	0.672	1.262E-06	2.542E-05	1008.785
FCGBP	0.673	5.487E-05	6.816E-04	145.938
LILRA4	0.673	3.675E-03	2.229E-02	82.310
MOCS1	0.678	2.327E-04	2.330E-03	154.593
PRKAB1	0.679	6.426E-15	6.995E-13	1358.446
IRF1	0.679	6.487E-13	4.957E-11	3021.599
VCAM1	0.680	6.135E-04	5.256E-03	130.096
WDR17	0.681	1.270E-04	1.405E-03	180.704
PLCXD2	0.682	4.506E-04	4.053E-03	70.972
FAS	0.682	4.807E-14	4.575E-12	1967.957
TEX9	0.684	4.464E-06	7.719E-05	225.181
ZNF337	0.685	2.753E-15	3.132E-13	1275.747
CDC42EP4	0.685	1.044E-08	3.514E-07	649.407
C9orf9	0.686	2.822E-03	1.815E-02	54.125
SOCS1	0.687	6.456E-10	2.799E-08	801.671
C12orf77	0.690	9.577E-05	1.105E-03	136.407
NACC2	0.691	4.432E-03	2.578E-02	787.927
SFXN5	0.693	5.459E-08	1.532E-06	461.953
SGK1	0.693	2.466E-05	3.414E-04	141.863
NPL	0.694	9.762E-05	1.123E-03	115.365

CRYM	0.695	2.616E-03	1.713E-02	101.492
PIDD1	0.698	1.852E-10	8.867E-09	1698.198
ZNF79	0.699	8.611E-11	4.508E-09	445.768
CKB	0.701	1.037E-05	1.631E-04	293.546
PLCD3	0.702	3.510E-04	3.284E-03	78.369
INPP1	0.706	8.645E-11	4.510E-09	511.805
ADCY1	0.706	2.296E-07	5.470E-06	219.260
RPS27L	0.706	4.710E-12	3.071E-10	2064.875
DCHS1	0.706	5.321E-04	4.664E-03	83.967
MT2A	0.706	1.266E-13	1.107E-11	579.082
IDUA	0.707	2.072E-05	2.944E-04	289.574
RELL1	0.707	1.407E-03	1.033E-02	82.330
C20orf194	0.707	1.634E-04	1.738E-03	109.114
PCNXL2	0.709	2.300E-09	8.970E-08	469.915
GRN	0.709	7.602E-13	5.722E-11	13573.146
PDK2	0.711	5.787E-06	9.658E-05	137.876
ASB2	0.712	4.721E-04	4.204E-03	88.835
MR1	0.713	1.583E-22	3.991E-20	2243.341
RINL	0.716	2.805E-13	2.319E-11	1579.712
CETP	0.718	6.211E-03	3.378E-02	50.624
CLCF1	0.719	9.272E-06	1.478E-04	225.113
GSN	0.719	1.352E-12	9.651E-11	1003.824
ORAI3	0.721	1.129E-10	5.772E-09	1803.254
CARD6	0.723	1.866E-04	1.939E-03	96.158
AFAP1L2	0.723	1.458E-05	2.182E-04	151.808
RUNX1	0.724	4.668E-09	1.719E-07	439.643
NOL4L	0.726	8.722E-10	3.707E-08	553.025
C12orf79	0.726	1.013E-03	7.895E-03	60.327
CD274	0.726	2.097E-11	1.230E-09	1675.344
RSAD2	0.727	1.173E-07	3.055E-06	277.911
NTPCR	0.729	1.858E-15	2.146E-13	706.084
TOB1	0.730	4.883E-11	2.687E-09	426.330
ANKRA2	0.731	9.438E-10	4.000E-08	463.715
FGR	0.734	5.249E-11	2.867E-09	3199.917
TNFRSF10B	0.734	2.239E-19	3.763E-17	5656.210
TM7SF3	0.734	7.267E-24	2.074E-21	2494.174
PXDC1	0.735	7.123E-04	5.957E-03	65.440
EGR3	0.736	8.683E-07	1.825E-05	722.432
RASSF4	0.736	1.846E-10	8.867E-09	1871.997
ADHFE1	0.737	1.377E-04	1.508E-03	90.515
ZNF154	0.737	1.937E-05	2.777E-04	102.564
XYLT1	0.737	7.030E-07	1.513E-05	432.944
LIPG	0.738	2.389E-03	1.586E-02	62.597
MUC20	0.739	1.694E-03	1.198E-02	54.060
TFPI2	0.741	5.012E-03	2.840E-02	68.578
ZNF528	0.742	1.583E-05	2.346E-04	134.939
ACY3	0.742	9.338E-04	7.405E-03	157.246
DACT3	0.743	2.797E-03	1.807E-02	56.620

LOC153684	0.743	3.521E-05	4.657E-04	106.645
NOP14-AS1	0.746	1.174E-10	5.962E-09	597.156
GPX1	0.747	1.370E-18	2.072E-16	3805.811
DRAM1	0.747	2.289E-13	1.924E-11	1307.380
CHI3L1	0.750	9.413E-03	4.700E-02	50.617
MPEG1	0.751	1.763E-09	7.056E-08	644.907
CLCN4	0.752	2.842E-04	2.760E-03	103.644
SEMA6A	0.753	3.736E-09	1.409E-07	605.211
APOBEC3H	0.755	2.743E-06	5.067E-05	166.774
ZMAT1	0.757	4.325E-04	3.921E-03	69.351
CFAP46	0.759	5.406E-03	3.014E-02	48.712
OXER1	0.760	2.842E-08	8.566E-07	296.176
ZBED2	0.760	2.290E-03	1.534E-02	55.328
SLC7A6	0.764	2.933E-18	4.267E-16	3660.922
GRASP	0.766	9.591E-03	4.770E-02	38.177
PTPRE	0.766	1.009E-10	5.176E-09	528.350
COL27A1	0.767	1.576E-03	1.132E-02	149.036
BAIAP2L1	0.767	9.974E-17	1.268E-14	1197.238
COL4A5	0.767	5.203E-04	4.574E-03	101.260
TRIAP1	0.768	4.622E-13	3.624E-11	588.837
C1orf186	0.769	6.270E-08	1.719E-06	237.982
NTNG1	0.770	2.335E-03	1.558E-02	48.617
CACNA1E	0.770	5.893E-11	3.184E-09	1057.303
PRKCDBP	0.773	1.524E-04	1.644E-03	98.739
HCG22	0.773	1.478E-04	1.601E-03	172.466
ITM2B	0.774	3.007E-13	2.473E-11	785.294
CDHR1	0.775	1.681E-07	4.155E-06	449.634
AEN	0.776	6.242E-27	2.099E-24	3077.146
ZNF117	0.776	5.693E-08	1.581E-06	329.417
SIRPA	0.778	1.119E-06	2.285E-05	470.200
PGAP1	0.779	1.669E-08	5.361E-07	168.979
GGT1	0.783	1.174E-14	1.243E-12	662.433
RAB9B	0.786	7.116E-03	3.781E-02	33.748
DCUN1D3	0.787	1.673E-10	8.258E-09	224.749
GBP1	0.788	1.709E-10	8.339E-09	990.256
TIGAR	0.788	5.257E-14	4.909E-12	886.201
TTYH3	0.789	1.473E-10	7.379E-09	5800.927
ADA	0.789	8.362E-20	1.506E-17	3048.838
PDLIM1	0.790	9.369E-32	4.573E-29	7590.093
IVL	0.792	9.695E-08	2.578E-06	197.950
PARD6G	0.793	1.389E-05	2.101E-04	122.054
PDCL3P4	0.795	2.475E-03	1.636E-02	41.426
SDSL	0.800	4.361E-05	5.586E-04	79.726
TXNIP	0.801	4.367E-20	8.057E-18	1742.528
TRIM3	0.804	7.093E-09	2.507E-07	283.924
ENC1	0.804	1.427E-11	8.565E-10	1723.936
LINC00324	0.806	9.148E-04	7.285E-03	58.578
WFS1	0.806	3.396E-07	7.772E-06	227.693

GATS	0.808	4.021E-13	3.219E-11	433.952
LOC100507577	0.810	2.530E-06	4.738E-05	142.454
IRAK2	0.810	4.587E-11	2.542E-09	304.464
SRGAP3	0.813	1.025E-07	2.722E-06	156.890
KALRN	0.815	7.207E-04	6.008E-03	51.147
STAT4	0.815	7.738E-05	9.211E-04	78.379
CUEDC1	0.816	9.387E-05	1.087E-03	108.051
CCND1	0.817	4.578E-06	7.881E-05	268.094
SBF2	0.817	7.118E-11	3.753E-09	334.654
CSF1	0.821	2.488E-13	2.069E-11	1542.973
SCAMP5	0.821	1.156E-05	1.797E-04	112.114
FAT1	0.822	3.481E-08	1.029E-06	528.868
SYN1	0.822	4.863E-04	4.313E-03	56.429
PPAP2B	0.825	8.048E-06	1.298E-04	121.784
TNFRSF10A	0.825	1.822E-07	4.465E-06	134.203
NDRG1	0.826	7.967E-09	2.765E-07	267.328
RGS9	0.826	1.896E-04	1.963E-03	111.763
LRG1	0.827	2.937E-03	1.871E-02	86.614
CASP1	0.829	1.794E-05	2.604E-04	236.475
TRIM22	0.829	2.385E-25	7.365E-23	9552.476
TCN2	0.830	3.860E-11	2.179E-09	275.007
FCMR	0.832	1.676E-11	9.905E-10	4224.671
PRICKLE2	0.834	1.667E-04	1.763E-03	58.070
TCONS_00029157	0.836	3.750E-04	3.475E-03	69.290
PLEKHF1	0.836	3.972E-14	3.852E-12	515.662
VWA5A	0.837	5.986E-03	3.278E-02	34.963
MMP14	0.839	1.720E-04	1.813E-03	90.818
MYO18B	0.839	7.162E-03	3.797E-02	29.698
CFAP70	0.841	4.070E-05	5.272E-04	64.707
MGC45922	0.842	1.520E-05	2.267E-04	84.168
WFDC2	0.843	3.072E-03	1.936E-02	67.505
TMEM175	0.844	5.976E-13	4.590E-11	1006.748
OSBP2	0.844	2.250E-04	2.264E-03	62.841
CCL22	0.845	3.058E-03	1.930E-02	18952.177
TNFRSF4	0.846	3.121E-05	4.226E-04	79.302
RAB15	0.847	4.012E-05	5.206E-04	121.510
NPDC1	0.847	1.304E-03	9.703E-03	407.501
PHPT1	0.849	2.235E-21	4.831E-19	2017.624
MS4A7	0.852	8.681E-05	1.015E-03	103.250
KLLN	0.854	1.196E-07	3.103E-06	163.507
LOC100507487	0.856	4.682E-03	2.686E-02	40.795
PLEKHB1	0.857	1.386E-03	1.022E-02	44.003
PTPN14	0.857	1.162E-03	8.849E-03	451.189
PERP	0.859	6.608E-05	8.043E-04	68.410
NUDT14	0.861	2.769E-04	2.708E-03	47.623
WDR63	0.862	4.374E-07	9.790E-06	107.229
TSNAXIP1	0.864	4.551E-03	2.626E-02	30.039

SPTBN5	0.864	2.250E-05	3.158E-04	75.090
EEPD1	0.865	1.829E-03	1.282E-02	45.314
APOBEC3C	0.869	5.761E-22	1.282E-19	2885.632
MAP3K12	0.871	1.572E-09	6.377E-08	387.374
KIF5A	0.872	7.328E-03	3.867E-02	39.727
INSL3	0.876	4.262E-03	2.505E-02	30.737
EPAS1	0.876	4.976E-04	4.395E-03	68.433
NLRP7	0.877	6.398E-03	3.465E-02	43.346
METTL7A	0.880	3.378E-14	3.340E-12	310.904
ALOX5	0.880	4.154E-05	5.368E-04	2163.236
PEX11G	0.881	8.376E-03	4.311E-02	24.597
PLEKHG1	0.882	1.891E-10	8.998E-09	485.147
CD109	0.882	1.147E-08	3.806E-07	310.487
LINC00663	0.884	4.381E-06	7.611E-05	74.085
YPEL3	0.884	4.756E-04	4.233E-03	657.346
MIR1204	0.885	8.967E-04	7.159E-03	35.916
SNX22	0.886	7.323E-12	4.617E-10	973.802
CES3	0.888	1.186E-05	1.829E-04	112.841
NOTCH1	0.892	7.962E-17	1.030E-14	6291.174
HCN2	0.893	8.680E-03	4.423E-02	26.604
NSG1	0.893	5.349E-09	1.922E-07	243.194
LPXN	0.895	2.412E-21	5.140E-19	4504.722
DDX60L	0.896	1.375E-07	3.496E-06	312.974
PIGR	0.896	8.125E-13	6.085E-11	332.494
GPM6A	0.896	3.540E-04	3.307E-03	86.197
NEIL1	0.897	1.702E-04	1.797E-03	71.389
FAM49A	0.899	1.718E-03	1.214E-02	39.953
CXCR5	0.901	3.377E-07	7.743E-06	262.843
LRRC56	0.901	3.072E-07	7.095E-06	124.214
CYP1B1	0.904	4.378E-04	3.959E-03	891.934
POU5F1	0.904	2.395E-03	1.589E-02	33.396
PLXNB2	0.905	3.285E-19	5.402E-17	11559.636
FAM83H	0.910	5.806E-10	2.539E-08	335.429
JAG1	0.910	9.774E-21	1.972E-18	1035.512
CDIP1	0.911	2.962E-17	4.074E-15	800.142
RNF19B	0.912	2.109E-30	9.118E-28	1182.246
RRM2B	0.913	1.564E-22	3.991E-20	1284.959
WDR66	0.914	2.541E-03	1.671E-02	27.741
ATP1A3	0.915	4.356E-03	2.548E-02	28.214
GAD1	0.921	7.478E-03	3.929E-02	39.234
ATF3	0.922	7.139E-17	9.393E-15	727.913
MLC1	0.930	1.835E-03	1.285E-02	65.971
SNX29P1	0.930	1.870E-03	1.307E-02	36.271
SNX18	0.930	4.131E-03	2.443E-02	29.397
CNR1	0.933	1.302E-10	6.543E-09	393.824
SNX29P2	0.934	3.784E-03	2.279E-02	27.578
SLC22A1	0.935	8.123E-03	4.199E-02	19.486
LCAT	0.936	2.159E-08	6.711E-07	100.351

CD24	0.937	3.899E-04	3.595E-03	48.961
CEP170B	0.938	5.116E-07	1.122E-05	172.102
KIAA1107	0.942	5.897E-03	3.236E-02	24.513
EMX1	0.942	4.511E-11	2.509E-09	529.364
DEPDC7	0.943	8.652E-04	6.954E-03	33.976
ABLIM1	0.943	8.743E-05	1.019E-03	632.409
CLU	0.945	6.256E-07	1.354E-05	89.812
DLG5	0.945	2.665E-14	2.707E-12	647.159
PLXNB1	0.945	2.733E-05	3.762E-04	992.364
PTRF	0.946	1.635E-06	3.205E-05	123.600
SESN2	0.946	1.581E-04	1.692E-03	3004.202
FOSB	0.948	1.281E-03	9.560E-03	47.147
PPM1D	0.948	3.164E-27	1.088E-24	598.545
ACP5	0.948	3.505E-15	3.928E-13	783.833
TPM2	0.951	6.230E-04	5.319E-03	35.617
RCAN3	0.952	9.866E-03	4.861E-02	25.812
CYP4F35P	0.954	4.649E-05	5.901E-04	90.081
FAM171A2	0.957	3.946E-04	3.632E-03	43.018
LOC284581	0.957	8.107E-03	4.195E-02	36.702
KCNJ2	0.958	4.728E-03	2.705E-02	22.854
ZMAT3	0.960	2.453E-22	5.799E-20	3170.930
PYROXD2	0.961	1.064E-06	2.191E-05	86.158
DDR1	0.964	8.336E-14	7.598E-12	882.335
REEP2	0.964	1.202E-05	1.840E-04	71.356
ZNF540	0.965	6.267E-05	7.672E-04	44.535
LGALS3BP	0.966	3.813E-10	1.727E-08	243.216
GCNT3	0.968	4.252E-05	5.471E-04	62.977
FAM169A	0.971	6.185E-11	3.330E-09	178.516
HS3ST1	0.975	8.589E-03	4.393E-02	23.114
LINC01599	0.975	1.037E-04	1.175E-03	43.498
EGOT	0.981	1.225E-03	9.234E-03	36.793
ARHGEF3	0.984	3.377E-19	5.494E-17	1006.870
GBP5	0.984	1.414E-06	2.815E-05	197.163
IRF6	0.984	9.402E-03	4.696E-02	20.499
CALD1	0.988	3.899E-03	2.331E-02	26.129
COL7A1	0.991	1.173E-06	2.379E-05	216.694
PLA1A	1.005	4.459E-23	1.205E-20	1175.186
FCRL4	1.006	1.174E-05	1.816E-04	143.823
XPC	1.006	2.192E-40	1.746E-37	4873.221
MAST4	1.012	1.200E-05	1.839E-04	75.268
ELF3	1.014	1.465E-08	4.735E-07	194.025
IL2RA	1.016	2.677E-11	1.534E-09	193.241
IL9R	1.018	4.141E-14	3.974E-12	218.334
FAT2	1.020	1.995E-04	2.048E-03	45.530
SIGLEC10	1.024	3.120E-09	1.192E-07	259.636
F5	1.028	4.561E-10	2.035E-08	296.587
BACE1	1.031	8.108E-05	9.592E-04	65.795
IGDCC4	1.031	1.040E-04	1.178E-03	58.912

SESN3	1.034	6.439E-11	3.454E-09	219.326
C1orf115	1.035	5.274E-03	2.951E-02	279.188
LTBR	1.039	6.689E-05	8.107E-04	71.982
LOC100506801	1.047	2.239E-03	1.507E-02	22.815
RAB11FIP4	1.051	5.071E-23	1.346E-20	988.618
TINCR	1.051	1.520E-03	1.097E-02	23.747
LOC100505915	1.052	4.932E-07	1.085E-05	91.688
RARRES3	1.052	6.989E-11	3.710E-09	231.995
P4HA2	1.053	2.694E-15	3.088E-13	195.207
ARHGEF37	1.054	3.216E-03	2.011E-02	27.255
LOC100507053	1.055	4.003E-04	3.671E-03	30.521
TMEM37	1.057	8.936E-04	7.143E-03	29.453
CYP2S1	1.061	7.268E-03	3.841E-02	55.048
CROCCP3	1.064	1.952E-08	6.176E-07	174.117
MAP4K4	1.065	1.900E-13	1.615E-11	886.359
BMP1	1.067	1.021E-04	1.165E-03	40.077
IL1A	1.069	9.202E-03	4.622E-02	47.528
ZNF561-AS1	1.073	1.860E-10	8.879E-09	112.542
IGFBP4	1.074	4.947E-21	1.025E-18	404.447
RAP1GAP2	1.075	1.001E-05	1.585E-04	92.810
COL6A1	1.083	8.538E-05	9.998E-04	43.681
ABCB9	1.090	6.225E-09	2.221E-07	81.874
FUCA1	1.092	3.115E-37	2.049E-34	1403.463
BCL2L14	1.102	8.668E-04	6.961E-03	27.371
ABCA12	1.102	6.747E-19	1.042E-16	523.117
NPR1	1.103	7.272E-05	8.725E-04	38.585
LAMP3	1.108	3.945E-08	1.148E-06	7407.302
BACE2	1.111	1.556E-08	5.019E-07	159.648
UBASH3B	1.115	1.766E-10	8.591E-09	131.077
GAMT	1.117	7.243E-28	2.810E-25	662.762
FAM46A	1.118	1.925E-22	4.623E-20	429.966
DDB2	1.121	9.393E-51	1.093E-47	3728.062
GAS6-AS1	1.130	3.595E-07	8.179E-06	135.229
ANKRD20A5P	1.132	3.794E-08	1.110E-06	157.717
ATP6V1C2	1.137	6.783E-03	3.632E-02	17.457
DNAH6	1.140	5.876E-04	5.083E-03	25.491
PLK3	1.140	1.830E-20	3.462E-18	821.958
E2F7	1.144	9.065E-42	7.620E-39	1694.037
ESPNL	1.148	1.292E-07	3.317E-06	143.339
ACVR1B	1.148	1.194E-05	1.834E-04	77.226
SNX21	1.153	2.398E-07	5.696E-06	63.164
GPR68	1.154	6.039E-07	1.311E-05	54.592
TANC2	1.154	2.713E-05	3.739E-04	37.536
BIRC7	1.155	4.699E-03	2.695E-02	50.803
TP53INP1	1.160	1.424E-28	5.671E-26	1007.845
KCNH3	1.164	8.515E-03	4.364E-02	14.018
C20orf197	1.167	1.142E-03	8.721E-03	23.268
ADCY9	1.174	2.100E-05	2.975E-04	43.257

MAP1LC3A	1.177	5.899E-07	1.284E-05	50.698
DFNB31	1.180	1.421E-05	2.145E-04	44.953
LOC102723824	1.183	9.747E-03	4.821E-02	15.280
LOC100505530	1.188	3.824E-10	1.727E-08	110.651
TLR4	1.190	8.827E-03	4.480E-02	20.612
KLK2	1.191	2.600E-04	2.566E-03	25.515
DOCK4	1.192	2.160E-08	6.711E-07	108.061
LOC101928973	1.193	1.087E-04	1.230E-03	31.037
KCNN3	1.196	2.930E-27	1.031E-24	4141.819
TNFRSF18	1.198	2.205E-07	5.296E-06	110.102
ADGRG1	1.200	2.825E-06	5.199E-05	55.435
MFGE8	1.201	4.946E-05	6.247E-04	280.596
FAM71F2	1.207	7.283E-07	1.556E-05	44.970
MUC19	1.208	1.564E-05	2.320E-04	85.298
SNAI3	1.210	2.533E-08	7.712E-07	74.872
TM4SF19-AS1	1.212	7.037E-03	3.748E-02	13.432
MEGF11	1.213	1.963E-03	1.358E-02	18.267
RAPH1	1.214	5.961E-04	5.142E-03	24.658
ITGAX	1.218	6.039E-04	5.197E-03	774.640
EPHB6	1.218	3.845E-03	2.308E-02	16.703
F2R	1.222	1.516E-17	2.124E-15	217.621
RAB44	1.233	2.379E-03	1.581E-02	16.395
ADAMTS7	1.241	7.752E-10	3.332E-08	216.719
SIGLEC5	1.251	4.825E-04	4.287E-03	62.033
KLHL14	1.252	7.473E-03	3.929E-02	13.482
GBP4	1.253	5.487E-05	6.816E-04	697.531
DPEP2	1.259	4.314E-04	3.917E-03	27.420
PODXL	1.259	1.487E-09	6.083E-08	80.402
LINC01033	1.261	6.569E-03	3.546E-02	14.264
SATB1	1.266	2.872E-04	2.781E-03	29.930
TFEC	1.267	8.245E-03	4.253E-02	29.693
CXCL10	1.268	5.405E-04	4.727E-03	38.394
PSTPIP2	1.269	3.819E-12	2.557E-10	266.408
RGS16	1.275	2.584E-10	1.199E-08	2015.596
APOBR	1.275	2.888E-18	4.243E-16	311.974
C10orf99	1.276	1.858E-07	4.542E-06	133.781
EDA2R	1.285	3.852E-33	2.241E-30	861.493
LPAR6	1.293	1.096E-04	1.238E-03	57.843
TNIP3	1.298	8.451E-10	3.612E-08	140.535
MTURN	1.306	3.195E-15	3.608E-13	162.602
UBD	1.309	3.369E-04	3.180E-03	336.323
DAB2IP	1.318	3.306E-05	4.423E-04	28.445
RIN1	1.319	2.169E-04	2.201E-03	29.976
MAMDC4	1.320	5.714E-21	1.168E-18	507.807
COL5A3	1.321	8.206E-08	2.205E-06	94.329
FADS3	1.326	8.578E-13	6.362E-11	291.428
MFSD4	1.332	2.495E-06	4.683E-05	35.644
PDLIM4	1.340	8.750E-04	7.019E-03	23.180

LAD1	1.358	7.334E-06	1.196E-04	84.124
MDM2	1.359	1.554E-69	5.879E-66	6147.969
FAM212A	1.360	4.398E-10	1.969E-08	61.886
SYTL1	1.360	3.790E-07	8.566E-06	922.051
GADD45A	1.361	6.574E-48	6.217E-45	1008.621
GPR141	1.366	2.360E-03	1.571E-02	15.188
CATIP-AS2	1.367	6.249E-04	5.330E-03	18.433
TNXB	1.369	3.287E-07	7.546E-06	84.760
IL13RA1	1.373	1.139E-10	5.802E-09	101.918
NCS1	1.375	5.729E-04	4.982E-03	22.064
FSD2	1.379	4.536E-06	7.826E-05	31.388
ASTN2	1.380	1.187E-24	3.591E-22	269.276
PTCHD4	1.380	6.893E-03	3.685E-02	11.926
MMP9	1.383	1.092E-05	1.707E-04	30.320
VANGL2	1.386	6.671E-12	4.223E-10	191.867
LINC01588	1.398	4.447E-22	1.004E-19	262.840
PALM	1.400	8.600E-03	4.394E-02	9.103
CD1C	1.402	2.818E-03	1.815E-02	15.375
PLAU	1.407	1.476E-13	1.269E-11	205.769
NPTXR	1.409	4.470E-07	9.961E-06	44.315
ACKR2	1.411	7.332E-03	3.868E-02	10.125
SLC2A12	1.413	1.643E-03	1.172E-02	14.143
TP53I11	1.416	7.238E-04	6.029E-03	22.005
C11orf96	1.436	1.958E-03	1.356E-02	22.896
DNAJC5B	1.436	9.386E-03	4.692E-02	10.211
CEL	1.439	2.467E-10	1.152E-08	58.000
CD7	1.445	9.757E-04	7.645E-03	40.003
CD101	1.447	1.188E-07	3.087E-06	44.441
FZD8	1.456	1.915E-05	2.752E-04	27.496
KCP	1.462	3.497E-14	3.435E-12	157.510
HPX	1.473	7.151E-03	3.794E-02	8.241
TLL2	1.479	1.378E-06	2.751E-05	60.013
ARHGEF10	1.486	3.542E-05	4.680E-04	32.185
FCER1G	1.488	6.053E-04	5.206E-03	19.435
PRF1	1.499	9.887E-11	5.088E-09	109.924
PSD3	1.511	1.785E-04	1.867E-03	19.030
ITGAM	1.519	1.644E-09	6.650E-08	849.489
NLRP3	1.521	4.427E-03	2.578E-02	11.578
BBC3	1.522	9.165E-28	3.382E-25	1025.590
RASGRF1	1.523	1.569E-15	1.840E-13	109.629
IL17RC	1.526	2.828E-04	2.748E-03	16.885
ANKRD65	1.532	2.497E-03	1.646E-02	16.487
MILR1	1.532	9.915E-09	3.356E-07	57.031
LACC1	1.540	4.186E-11	2.346E-09	101.446
PLA2G4E	1.541	3.705E-03	2.241E-02	15.934
RGS12	1.543	1.225E-14	1.287E-12	193.676
ST6GALNAC2	1.546	2.055E-08	6.437E-07	48.633
LOC100129697	1.563	1.963E-03	1.358E-02	13.646

EDN2	1.565	3.142E-05	4.248E-04	22.173
DAGLA	1.569	1.654E-03	1.178E-02	14.295
LILRB3	1.570	3.243E-03	2.025E-02	14.443
CDKN1A	1.572	1.940E-104	2.935E-100	25333.407
CRTAM	1.572	5.038E-06	8.547E-05	40.919
CILP	1.573	1.874E-03	1.309E-02	12.977
VCAN	1.581	2.302E-13	1.924E-11	146.405
TSPAN11	1.582	6.467E-06	1.069E-04	23.337
ST5	1.583	1.308E-12	9.377E-11	65.915
SYTL2	1.586	2.970E-10	1.362E-08	92.262
PPL	1.598	1.130E-05	1.764E-04	20.743
PLEKHG3	1.601	2.188E-09	8.623E-08	58.075
SERPINF1	1.604	3.125E-04	2.996E-03	16.369
MUC22	1.611	6.007E-03	3.288E-02	9.667
MYOF	1.621	3.545E-05	4.680E-04	30.742
GAS6	1.630	5.308E-09	1.912E-07	90.713
LOC101928841	1.640	3.554E-05	4.687E-04	27.199
SERPINA1	1.653	1.290E-09	5.331E-08	53.256
MICALL2	1.658	1.035E-12	7.565E-11	98.141
FOSL1	1.664	1.716E-15	1.997E-13	152.635
RNF144B	1.676	2.032E-03	1.393E-02	12.527
FDXR	1.679	7.193E-69	1.814E-65	3841.658
LOC100130093	1.684	2.135E-06	4.073E-05	28.582
TMEM229B	1.689	2.676E-09	1.033E-07	54.608
EMP1	1.692	5.637E-03	3.116E-02	9.216
CTSW	1.697	2.219E-05	3.120E-04	25.094
SDC1	1.700	3.607E-04	3.354E-03	484.850
TNFRSF10D	1.712	2.859E-31	1.352E-28	218.805
PGF	1.713	6.198E-09	2.217E-07	49.241
BTBD19	1.717	8.601E-14	7.792E-12	106.837
NTN1	1.727	2.042E-16	2.532E-14	113.126
TTLL6	1.727	6.747E-03	3.616E-02	7.347
ACER2	1.735	7.440E-33	4.169E-30	201.443
SH3PXD2B	1.741	1.973E-08	6.218E-07	40.346
DQX1	1.745	2.474E-07	5.839E-06	29.077
FAXDC2	1.754	2.829E-03	1.819E-02	10.047
RGAG4	1.755	1.252E-14	1.307E-12	74.800
CMBL	1.758	3.264E-32	1.703E-29	387.864
FHL2	1.767	1.155E-19	2.055E-17	137.953
CABYR	1.771	1.281E-08	4.214E-07	41.082
ADRB2	1.773	1.387E-09	5.704E-08	42.547
HSPG2	1.774	1.775E-22	4.402E-20	173.031
CAMP	1.776	1.713E-09	6.876E-08	49.631
MYH14	1.778	4.311E-03	2.525E-02	8.850
MUC4	1.778	6.308E-04	5.371E-03	17.190
TNFSF4	1.783	2.247E-11	1.308E-09	649.982
FAM212B	1.790	5.608E-58	8.485E-55	1314.782
SESN1	1.790	4.756E-50	5.139E-47	331.489

HCAR2	1.791	1.212E-03	9.166E-03	20.889
ITIH5	1.795	9.608E-03	4.775E-02	9.543
APLP1	1.796	3.459E-17	4.715E-15	94.642
LOC101927412	1.799	9.005E-03	4.546E-02	7.342
IL6	1.802	9.113E-03	4.588E-02	6.998
PHLDA3	1.808	2.684E-69	8.121E-66	2038.593
CASKIN1	1.819	3.087E-06	5.623E-05	20.305
FEZ1	1.825	1.576E-12	1.104E-10	821.411
ETV7	1.834	2.265E-13	1.915E-11	138.372
GAL3ST4	1.839	2.163E-18	3.241E-16	126.328
ESPN	1.852	3.910E-10	1.761E-08	59.975
GLS2	1.853	2.348E-23	6.459E-21	148.671
GNAI1	1.859	3.001E-03	1.900E-02	13.140
PROCR	1.862	2.193E-06	4.168E-05	22.075
LINC01573	1.865	1.154E-14	1.229E-12	84.448
FAM212B-AS1	1.867	1.326E-07	3.388E-06	26.021
MYO1A	1.872	4.591E-06	7.893E-05	18.813
CYP4F3	1.877	8.447E-10	3.612E-08	49.853
DLGAP2	1.878	7.569E-03	3.967E-02	7.419
TNC	1.888	4.468E-07	9.961E-06	41.638
PTPRF	1.893	6.573E-17	8.724E-15	73.492
IL10RB-AS1	1.904	1.408E-04	1.534E-03	13.003
PAPLN	1.925	5.198E-19	8.107E-17	1291.457
FRMPD2	1.925	1.501E-22	3.916E-20	139.333
COL23A1	1.925	3.849E-03	2.309E-02	8.632
CASP14	1.939	2.281E-03	1.531E-02	10.268
LPIN3	1.956	1.073E-07	2.826E-06	26.841
LOC100996351	1.957	5.706E-03	3.146E-02	6.122
LRRC66	1.966	3.648E-03	2.218E-02	6.775
RHCE	1.972	4.569E-03	2.634E-02	6.827
LINC01021	1.982	8.752E-47	7.790E-44	312.103
MYO7A	1.989	2.784E-03	1.801E-02	8.375
RGAG1	2.006	7.805E-09	2.721E-07	32.626
PLEKHA6	2.006	1.170E-16	1.475E-14	89.560
PLEKHG6	2.012	8.411E-28	3.181E-25	173.399
TPTE2P1	2.023	6.969E-03	3.718E-02	7.294
CXCL8	2.033	2.289E-03	1.534E-02	11.441
OTP	2.035	4.277E-17	5.777E-15	102.827
OLFM2	2.046	6.923E-03	3.699E-02	5.354
HCAR3	2.047	3.164E-14	3.150E-12	96.580
CHST6	2.059	1.090E-13	9.758E-12	63.951
COL3A1	2.060	3.674E-04	3.413E-03	9.349
C10orf10	2.068	2.874E-06	5.277E-05	484.910
SERPINE1	2.069	5.048E-08	1.425E-06	23.347
TNFSF15	2.071	3.316E-04	3.145E-03	15.552
TFCP2L1	2.075	1.049E-08	3.521E-07	37.062
WBSCR27	2.080	2.183E-08	6.762E-07	32.390
BDNF	2.081	6.629E-03	3.569E-02	7.781

LRP1	2.082	1.609E-20	3.081E-18	168.162
LRRC25	2.084	3.052E-12	2.071E-10	53.304
CALHM1	2.089	1.320E-04	1.456E-03	12.478
TP53I3	2.094	1.032E-55	1.420E-52	1481.213
CALML6	2.122	1.693E-08	5.428E-07	26.559
ITIH3	2.123	4.386E-04	3.962E-03	9.957
KLB	2.138	1.018E-02	4.982E-02	4.646
PYHIN1	2.138	1.593E-10	7.903E-09	49.870
GPSM1	2.150	1.192E-05	1.834E-04	13.297
CYP4F2	2.151	6.294E-05	7.698E-04	12.087
CCL1	2.160	2.422E-04	2.412E-03	20.825
CD6	2.239	1.766E-19	3.002E-17	90.694
SIGLEC14	2.262	2.041E-08	6.407E-07	135.621
RRAD	2.263	3.796E-37	2.393E-34	180.759
ASPA	2.265	7.092E-07	1.524E-05	20.686
FAM198B	2.284	2.095E-04	2.140E-03	9.465
SPATA18	2.297	1.857E-60	3.122E-57	357.673
MAMLD1	2.314	1.016E-02	4.976E-02	3.988
DRAXIN	2.320	9.290E-03	4.656E-02	4.749
PLK2	2.320	1.272E-24	3.773E-22	76.299
ITK	2.328	3.057E-10	1.398E-08	30.045
ZFR2	2.330	6.779E-05	8.193E-04	10.287
TAC3	2.333	3.879E-03	2.321E-02	4.836
SEC16B	2.344	2.717E-04	2.668E-03	8.565
KIAA1671	2.374	6.207E-48	6.217E-45	350.852
ARVCF	2.384	3.920E-13	3.172E-11	30.699
LOC253573	2.416	8.863E-03	4.494E-02	3.914
TNFRSF10C	2.436	2.996E-22	6.974E-20	76.131
HLF	2.450	5.390E-05	6.734E-04	10.441
PLIN4	2.469	5.886E-08	1.631E-06	17.833
EPS8L2	2.493	8.220E-09	2.827E-07	424.070
VWCE	2.496	1.822E-70	9.188E-67	508.688
IFNG	2.512	2.338E-11	1.355E-09	37.809
DNAH3	2.516	4.197E-03	2.474E-02	5.092
ZNF385A	2.528	1.385E-13	1.198E-11	577.993
ISLR2	2.538	7.450E-05	8.903E-04	10.417
SORCS2	2.539	1.825E-06	3.541E-05	24.891
LINC01107	2.563	3.504E-05	4.638E-04	11.324
NYNRIN	2.571	5.748E-05	7.117E-04	9.839
GRIN2C	2.575	8.023E-09	2.768E-07	21.604
GABBR2	2.584	5.354E-04	4.685E-03	7.626
PRODH	2.597	3.240E-18	4.669E-16	69.932
EPHA2	2.602	3.235E-20	6.042E-18	89.748
COL2A1	2.610	4.972E-03	2.821E-02	4.521
SMPD3	2.617	1.690E-38	1.162E-35	151.287
SEZ6	2.620	3.573E-03	2.183E-02	5.560
RUNDC3A	2.628	3.536E-03	2.168E-02	4.295
FBN2	2.631	6.910E-26	2.273E-23	113.495

CSTA	2.641	7.172E-03	3.801E-02	3.554
NEURL1B	2.643	1.563E-19	2.718E-17	76.321
LY6K	2.653	5.341E-04	4.679E-03	8.455
FLJ16779	2.656	3.339E-03	2.073E-02	5.624
PDE4C	2.672	3.032E-32	1.638E-29	182.037
KANK3	2.710	5.414E-12	3.471E-10	27.318
PHYHIP	2.719	8.301E-08	2.227E-06	16.313
SULF2	2.747	1.738E-16	2.173E-14	540.851
NOS1	2.748	1.647E-05	2.422E-04	10.450
TCEA3	2.760	7.759E-04	6.387E-03	5.466
STRA6	2.761	5.186E-14	4.904E-12	28.922
AATBC	2.766	7.451E-09	2.610E-07	23.378
GRM4	2.790	5.507E-03	3.055E-02	3.455
TGFBI	2.792	8.506E-06	1.367E-04	14.245
PRSS12	2.793	7.910E-05	9.386E-04	7.700
ATP4B	2.817	1.915E-03	1.329E-02	4.580
DMBT1	2.821	3.577E-03	2.183E-02	5.360
PXT1	2.821	5.123E-12	3.313E-10	21.079
ADGRA2	2.838	1.499E-04	1.624E-03	6.280
P2RY2	2.840	1.299E-03	9.673E-03	4.913
AXL	2.858	1.653E-08	5.320E-07	30.312
LGALS9C	2.872	4.170E-31	1.856E-28	146.930
DPYSL4	2.877	1.274E-07	3.279E-06	96.355
ANKRD20A12P	2.920	1.076E-06	2.208E-05	13.134
CSF2	2.971	3.897E-04	3.595E-03	5.792
LINC00887	2.977	3.891E-03	2.327E-02	3.384
LOC102723769	3.007	1.071E-05	1.675E-04	9.408
C17orf82	3.030	3.417E-04	3.217E-03	5.732
LIF	3.034	5.592E-51	7.051E-48	171.110
PADI4	3.059	1.547E-25	4.982E-23	83.803
CEACAM1	3.073	1.681E-10	8.258E-09	84.346
KRT17	3.113	3.986E-31	1.827E-28	91.227
GDF15	3.125	6.971E-18	9.857E-16	365.532
EPPK1	3.134	4.107E-06	7.184E-05	10.556
EFCAB10	3.135	1.546E-03	1.113E-02	3.953
RIMS4	3.142	3.523E-03	2.161E-02	3.950
GRHL3	3.175	3.534E-29	1.485E-26	55.602
PVRL4	3.179	1.167E-20	2.264E-18	51.774
CXCL9	3.191	9.718E-04	7.626E-03	37.858
ZSCAN4	3.235	8.671E-07	1.825E-05	10.237
FAM135B	3.258	1.425E-06	2.832E-05	12.317
UNC5B	3.290	6.951E-04	5.842E-03	4.168
C12orf54	3.304	7.289E-04	6.063E-03	4.014
FAM83H-AS1	3.328	2.295E-04	2.304E-03	4.813
TRIM55	3.358	1.824E-08	5.811E-07	21.831
LCE1D	3.427	3.455E-05	4.586E-04	9.142
KLK4	3.490	7.453E-04	6.179E-03	3.589
SLC5A5	3.494	5.340E-11	2.906E-09	16.313

ATP8B4	3.530	4.350E-08	1.247E-06	13.135
SLC52A1	3.535	1.849E-10	8.867E-09	17.727
LGALS9B	3.536	7.077E-13	5.381E-11	30.007
IP6K3	3.547	1.266E-03	9.484E-03	3.902
MGC50722	3.574	1.922E-22	4.623E-20	52.104
LOC102723373	3.588	1.512E-04	1.634E-03	5.704
AREG	3.625	1.354E-05	2.055E-04	7.842
FLJ36000	3.635	3.184E-04	3.040E-03	5.033
TEX37	3.638	1.409E-04	1.534E-03	6.049
HES2	3.673	1.564E-61	3.381E-58	140.831
ALDH1A3	3.700	9.973E-05	1.143E-03	4.947
FAM13C	3.850	1.048E-03	8.131E-03	3.542
LCE1F	3.900	2.108E-11	1.232E-09	68.769
CXCR2	3.957	4.215E-12	2.785E-10	18.157
PLXNB3	4.011	5.020E-39	3.617E-36	69.970
CSPG4	4.066	2.070E-25	6.526E-23	370.955
LCE1C	4.107	1.899E-61	3.591E-58	194.983
SMIM10L2A	4.173	7.137E-07	1.532E-05	8.221
LCE1B	4.206	1.623E-19	2.790E-17	150.008
UNC13A	4.330	2.270E-24	6.605E-22	202.782
LCE1E	4.346	1.235E-80	9.346E-77	194.380
RGMA	4.511	3.984E-06	7.018E-05	5.769
LOC101927501	4.580	3.745E-11	2.122E-09	15.875
UNC5B-AS1	4.748	4.971E-06	8.488E-05	5.020
USH1G	4.778	1.399E-11	8.431E-10	15.369
GDNF	5.335	2.675E-06	4.967E-05	4.078
ZBTB7C	5.542	6.990E-08	1.906E-06	6.457
KITLG	5.643	5.940E-08	1.643E-06	6.127
