# Supplementary material for: A novel integrative multi-omics approach to unravel the genetic determinants of rare diseases with application in sinusoidal obstruction syndrome
Source: PLoS One. 2023 Apr 5;18(4):e0281892. doi: 10.1371/journal.pone.0281892 (PMC10075428; doi:10.1371/journal.pone.0281892)
Supplement: S3 Table — (PDF) [file pone.0281892.s003.pdf]

Supplementary Table S02. Over-representation analysis of differentially expressed genes in lymphoblastoid cell lines, sorted by strength of association.

	Enriched pathway-based sets	Main Catergory	Category	Genes	GeneRatio	Bg-Ratio	p-value	p-adjusted	q-value
**UP-regulated**									
**KEGG ID**									
hsa04115	***p53 signaling pathway***	Cellular Processes	Cell growth and death	APAF1/ATM/BAX/BBC3/BCL2/BCL2L1/CCND1/CCNG1/CCNG2/CD82/CDKN1A/DDB2/EI24/FAS/GADD45A/MDM2/PERP/PIDD1/PMAIP1/PPM1D/PTEN/RRM2B/SERPINE1/SESN1/SESN2/SESN3/SHISA5/TNFRSF10A/TNFRSF10B/TP53I3/ZMAT3	31/745	72/6791	2.67E-12	8.22E-10	6.01E-10
hsa04210	***Apoptosis***	Cellular Processes	Cell growth and death	APAF1/ATM/BAX/BBC3/BCL2/BCL2L1/BIRC3/CFLAR/CTSB/CTSD/CTSH/CTSO/CTSS/CTSW/CTSZ/DAB2IP/FAS/GADD45A/HRAS/ITPR1/ITPR2/LMNA/MAP2K2/PIDD1/PIK3R3/PMAIP1/PRF1/TNFRSF10A/TNFRSF10B/TNFRSF1A/TNFSF10/TRAF1	32/745	134/6791	1.38E-05	1.57E-04	1.15E-04
hsa04217	***Necroptosis***	Cellular Processes	Cell growth and death	BAX/BCL2/BIRC3/CASP1/CFLAR/CYLD/FAS/IFNG/IL1A/JAK1/JAK3/NLRP3/PLA2G4E/RIPK3/SQSTM1/STAT1/STAT2/STAT3/STAT4/STAT5A/TLR4/TNFAIP3/TNFRSF10A/TNFRSF10B/TNFRSF1A/TNFSF10/TYK2/ZBP1	28/745	121/6791	8.59E-05	7.78E-04	5.69E-04
hsa04215	***Apoptosis - multiple species***	Cellular Processes	Cell growth and death	APAF1/BAX/BBC3/BCL2/BCL2L1/BIRC3/BIRC7/PMAIP1/TNFRSF1A	9/745	30/6791	3.73E-03	2.17E-02	1.59E-02

hsa04142	***Lysosome***	Cellular Processes	Transport and catabolism	ABCA2/ABCB9/ACP5/AP1G2/AP1S3/ARSA/ATP6AP1/CD68/CTSA/CTSB/CTSD/CTSH/CTSO/CTSS/CTSW/CTSZ/FUCA1/GALNS/GM2A/GNPTAB/GNS/HEXA/HGSNAT/IDS/IDUA/LAMP3/LAPTM5/LIPA/MAN2B1/MANBA/NEU1/PLA2G15/PSAP/TPP1	34/745	124/6791	2.44E-07	6.26E-06	4.58E-06
hsa04145	***Phagosome***	Cellular Processes	Transport and catabolism	ATP6AP1/ATP6V1A/ATP6V1C2/CTSS/DYNC1H1/DYNC1LI2/EEA1/HLA-DMA/HLA-DMB/HLA-DOA/HLA-DOB/HLA-DPA1/HLA-DPB1/HLA-DQA1/HLA-DQA2/HLA-DRA/HLA-DRB1/HLA-DRB5/ITGAM/ITGAV/ITGB1/MRC2/NCF1/NCF2/NOS1/RAB5B/RILP/STX12/TAP1/TLR4/TLR6/TUBB6	32/745	146/6791	8.45E-05	7.78E-04	5.69E-04
hsa04668	***TNF signaling pathway***	Environmental Information Processing	Signal transduction	BIRC3/CCL5/CFLAR/CSF1/CSF2/CXCL10/DAB2IP/FAS/ICAM1/IL6/IRF1/JAG1/LIF/MAP2K6/MAPK11/MAPK13/MMP14/MMP9/PIK3R3/RIPK3/RPS6KA5/SOCS3/TAB3/TNFAIP3/TNFRSF1A/TNFRSF1B/TRAF1/VCAM1	28/745	108/6791	9.18E-06	1.09E-04	7.95E-05
hsa04064	***NF-kappa B signaling pathway***	Environmental Information Processing	Signal transduction	ATM/BCL10/BCL2/BCL2L1/BIRC3/CD40/CFLAR/CXCL8/CYLD/DDX58/EDA2R/EDARADD/GADD45A/ICAM1/IL1R1/LTBR/PIDD1/PLAU/PLCG1/PLCG2/TAB3/TLR4/TNFAIP3/TNFRSF1A/TRAF1/VCAM1	26/745	99/6791	1.49E-05	1.64E-04	1.20E-04

hsa04070	***Phosphatidylinositol signaling system***	Environmental Information Processing	Signal transduction	CALML6/CDS2/DGKA/DGKZ/INPP1/INPP4A/INPP4B/INPP5D/IP6K2/IP6K3/ITPKC/ITPR1/ITPR2/PIK3R3/PIP4K2C/PLCD3/PLCG1/PLCG2/PPIP5K1/PTEN	20/745	91/6791	1.69E-03	1.15E-02	8.40E-03
hsa04068	***FoxO signaling pathway***	Environmental Information Processing	Signal transduction	ATM/CCND1/CCNG2/CDKN1A/GABARAP/GABARAPL1/GABARAPL2/GADD45A/HRAS/IL6/MAP2K2/MAPK11/MAPK13/MDM2/PIK3R3/PLK2/PLK3/PRKAB1/PRKAB2/PTEN/RBL2/SGK1/STAT3/TGFBR1/TNFSF10	25/745	129/6791	3.10E-03	1.91E-02	1.40E-02
hsa04630	***JAK-STAT signaling pathway***	Environmental Information Processing	Signal transduction	BCL2/BCL2L1/CCND1/CDKN1A/CSF2/HRAS/IFNG/IL12RB1/IL12RB2/IL13RA1/IL2RA/IL4R/IL6/IL9R/JAK1/JAK3/LIF/PDGFA/PIK3R3/SOCS1/SOCS3/STAT1/STAT2/STAT3/STAT4/STAT5A/TYK2	27/745	145/6791	3.88E-03	2.21E-02	1.62E-02
hsa04514	***Cell adhesion molecules***	Environmental Information Processing	Signaling molecules and interaction	CADM1/CD226/CD274/CD40/CD6/CD80/CD86/CD99/F11R/HLA-DMA/HLA-DMB/HLA-DOA/HLA-DOB/HLA-DPA1/HLA-DPB1/HLA-DQA1/HLA-DQA2/HLA-DRA/HLA-DRB1/HLA-DRB5/ICAM1/ICAM3/ITGAM/ITGAV/ITGB1/ITGB8/NRCAM/NTNG1/PDCD1LG2/PTPRF/SDC1/SDC4/SPN/VCAM1/VCAN	35/745	132/6791	3.95E-07	8.69E-06	6.36E-06

hsa04060	***Cytokine-cytokine receptor interaction***	Environmental Information Processing	Signaling molecules and interaction	ACVR1B/CCL1/CCL22/CCL5/CCR1/CCR7/CD40/CD70/CLCF1/CSF1/CSF2/CXCL10/CXCL8/CXCL9/CXCR2/CXCR5/EBI3/EDA2R/FAS/GDF15/IFNG/IL12RB1/IL12RB2/IL13RA1/IL16/IL17RC/IL1A/IL1R1/IL2RA/IL4R/IL6/IL9R/LIF/LTBR/RELT/TGFBR1/TNFRSF10A/TNFRSF10B/TNFRSF10C/TNFRSF10D/TNFRSF14/TNFRSF18/TNFRSF1A/TNFRSF1B/TNFRSF21/TNFRSF4/TNFRSF9/TNFSF10/TNFSF15/TNFSF4/TNFSF9	51/745	246/6791	3.98E-06	5.84E-05	4.27E-05
hsa04061	***Viral protein interaction with cytokine and cytokine receptor***	Environmental Information Processing	Signaling molecules and interaction	CCL1/CCL22/CCL5/CCR1/CCR7/CSF1/CXCL10/CXCL8/CXCL9/CXCR2/CXCR5/IL2RA/IL6/LTBR/TNFRSF10A/TNFRSF10B/TNFRSF10C/TNFRSF10D/TNFRSF14/TNFRSF1A/TNFRSF1B/TNFSF10	22/745	83/6791	5.75E-05	5.64E-04	4.12E-04
hsa04512	***ECM-receptor interaction***	Environmental Information Processing	Signaling molecules and interaction	AGRN/CD44/COL2A1/COL4A5/COL6A1/COL9A2/HSPG2/ITGA1/ITGA10/ITGAV/ITGB1/ITGB8/LAMC1/NPNT/SDC1/SDC4/TNC/TNXB	18/745	83/6791	3.29E-03	1.99E-02	1.45E-02

hsa05200	***Pathways in cancer***	Human Diseases	Cancer: overview	ADCY1/ADCY9/APAF1/ARHGEF12/BAX/BBC3/BCL2/BCL2L1/BIRC3/BIRC7/CALML6/CCND1/CDKN1A/COL4A5/CXCL8/DDB2/EPAS1/F2R/FAS/FZD6/FZD8/GADD45A/GNAI1/GNG2/GNGT2/GSTM2/HDAC1/HIF1A/HRAS/IFNG/IL12RB1/IL12RB2/IL13RA1/IL2RA/IL4R/IL6/ITGAV/ITGB1/JAG1/JAK1/JAK3/KITLG/LAMC1/LPAR6/MAP2K2/MDM2/MMP9/NCOA3/NCOA4/NOTCH1/NOTCH2/NQO1/PDGFA/PGF/PIK3R3/PLCG1/PLCG2/PMAIP1/PML/PTEN/PTGER4/RALB/RALGDS/RB1/RPS6KA5/RUNX1/SPI1/STAT1/STAT2/STAT3/STAT4/STAT5A/TCF7/TGFBR1/TRAF1/TRAF4	76/745	496/6791	1.26E-03	9.28E-03	6.79E-03
hsa05202	***Transcriptional misregulation in cancer***	Human Diseases	Cancer: overview	ATM/BAX/BCL2L1/BIRC3/BMP2K/CD40/CD86/CDKN1A/CSF2/CXCL8/DDB2/ETV5/ETV6/ETV7/FLT1/GADD45A/HDAC1/IL6/ITGAM/LDB1/LMO2/MDM2/MMP9/PBX3/PDGFA/PLAU/PML/RUNX1/SPI1/TRAF1	30/745	156/6791	1.43E-03	9.99E-03	7.31E-03

hsa05205	***Proteoglycans in cancer***	Human Diseases	Cancer: overview	ANK1/ARHGEF12/CCND1/CD44/CDKN1A/FAS/FZD6/FZD8/HIF1A/HRAS/HSPG2/ITGAV/ITGB1/ITPR1/ITPR2/MAP2K2/MAPK11/MAPK13/MDM2/MMP9/PAK1/PIK3R3/PLAU/PLAUR/PLCG1/PLCG2/PXN/SDC1/SDC4/SRC/STAT3/TIAM1/TLR4/VAV2	34/745	197/6791	4.64E-03	2.46E-02	1.80E-02
hsa05222	***Small cell lung cancer***	Human Diseases	Cancer: specific types	APAF1/BAX/BCL2/BCL2L1/BIRC3/BIRC7/CCND1/CDKN1A/COL4A5/DDB2/GADD45A/ITGAV/ITGB1/LAMC1/PIK3R3/PTEN/RB1/TRAF1/TRAF4	19/745	92/6791	4.56E-03	2.46E-02	1.80E-02
hsa05223	***Non-small cell lung cancer***	Human Diseases	Cancer: specific types	BAX/CCND1/CDKN1A/DDB2/GADD45A/HRAS/JAK3/KIF5A/MAP2K2/PIK3R3/PLCG1/PLCG2/RB1/STAT3/STAT5A	15/745	72/6791	1.02E-02	4.83E-02	3.54E-02
hsa05416	***Viral myocarditis***	Human Diseases	Cardiovascular disease	CCND1/CD40/CD80/CD86/DMD/FYN/HLA-DMA/HLA-DMB/HLA-DOA/HLA-DOB/HLA-DPA1/HLA-DPB1/HLA-DQA1/HLA-DQA2/HLA-DRA/HLA-DRB1/HLA-DRB5/ICAM1/PRF1	19/745	57/6791	5.16E-06	6.62E-05	4.84E-05
hsa05417	***Lipid and atherosclerosis***	Human Diseases	Cardiovascular disease	APAF1/BAX/BCL2/BCL2L1/CALML6/CASP1/CCL5/CD40/CXCL8/FAS/HRAS/ICAM1/IL6/ITPR1/MAP2K6/MAPK11/MAPK13/MIB2/MMP9/NCF1/NCF2/NLRP3/PIK3R3/PLCG1/POU2F2/SRC/STAT3/TANK/TLR4/TLR6/TNFRSF10A/TNFRSF10B/TNFRSF1A/TNFSF10/VAV2/VCAM1	36/745	197/6791	1.32E-03	9.45E-03	6.91E-03

hsa01524	***Platinum drug resistance***	Human Diseases	Drug resistance: antineoplastic	APAF1/ATM/ATP7A/BAX/BBC3/BCL2/BCL2L1/BIRC3/CDKN1A/FAS/GSTM2/MDM2/PIK3R3/PMAIP1/POLH/REV3L	16/745	67/6791	1.94E-03	1.27E-02	9.30E-03
hsa01522	***Endocrine resistance***	Human Diseases	Drug resistance: antineoplastic	ADCY1/ADCY9/BAX/BCL2/CCND1/CDKN1A/HRAS/JAG1/MAP2K2/MAPK11/MAPK13/MDM2/MMP9/NCOA3/NOTCH1/NOTCH2/PIK3R3/RB1/SRC	19/745	92/6791	4.56E-03	2.46E-02	1.80E-02
hsa01521	***EGFR tyrosine kinase inhibitor resistance***	Human Diseases	Drug resistance: antineoplastic	AXL/BAX/BCL2/BCL2L1/GAS6/HRAS/IL6/JAK1/MAP2K2/PDGFA/PIK3R3/PLCG1/PLCG2/PTEN/SRC/STAT3	16/745	78/6791	9.45E-03	4.62E-02	3.38E-02
hsa04940	***Type I diabetes mellitus***	Human Diseases	Endocrine and metabolic disease	CD80/CD86/FAS/GAD1/HLA-DMA/HLA-DMB/HLA-DOA/HLA-DOB/HLA-DPA1/HLA-DPB1/HLA-DQA1/HLA-DQA2/HLA-DRA/HLA-DRB1/HLA-DRB5/IFNG/IL1A/PRF1	18/745	39/6791	2.90E-08	1.49E-06	1.09E-06
hsa04933	***AGE-RAGE signaling pathway in diabetic complications***	Human Diseases	Endocrine and metabolic disease	BAX/BCL2/CCND1/COL3A1/COL4A5/CXCL8/HRAS/ICAM1/IL1A/IL6/MAPK11/MAPK13/PIK3R3/PLCD3/PLCG1/PLCG2/PRKCD/SERPINE1/STAT1/STAT3/STAT5A/TGFBR1/VCAM1	23/745	97/6791	2.47E-04	2.17E-03	1.59E-03
hsa05332	***Graft-versus-host disease***	Human Diseases	Immune disease	CD80/CD86/FAS/HLA-DMA/HLA-DMB/HLA-DOA/HLA-DOB/HLA-DPA1/HLA-DPB1/HLA-DQA1/HLA-DQA2/HLA-DRA/HLA-DRB1/HLA-DRB5/IFNG/IL1A/IL6/PRF1	18/745	34/6791	1.75E-09	2.70E-07	1.97E-07

hsa05330	***Allograft rejection***	Human Diseases	Immune disease	CD40/CD80/CD86/FAS/HLA-DMA/HLA-DMB/HLA-DOA/HLA-DOB/HLA-DPA1/HLA-DPB1/HLA-DQA1/HLA-DQA2/HLA-DRA/HLA-DRB1/HLA-DRB5/IFNG/PRF1	17/745	35/6791	2.72E-08	1.49E-06	1.09E-06
hsa05323	***Rheumatoid arthritis***	Human Diseases	Immune disease	ACP5/ATP6AP1/ATP6V1A/ATP6V1C2/CCL5/CD80/CD86/CSF1/CSF2/CXCL8/FLT1/HLA-DMA/HLA-DMB/HLA-DOA/HLA-DOB/HLA-DPA1/HLA-DPB1/HLA-DQA1/HLA-DQA2/HLA-DRA/HLA-DRB1/HLA-DRB5/ICAM1/IFNG/IL1A/IL6/TLR4	27/745	84/6791	1.28E-07	3.93E-06	2.88E-06
hsa05310	***Asthma***	Human Diseases	Immune disease	CD40/FCER1G/HLA-DMA/HLA-DMB/HLA-DOA/HLA-DOB/HLA-DPA1/HLA-DPB1/HLA-DQA1/HLA-DQA2/HLA-DRA/HLA-DRB1/HLA-DRB5	13/745	25/6791	4.46E-07	9.15E-06	6.69E-06
hsa05321	***Inflammatory bowel disease***	Human Diseases	Immune disease	HLA-DMA/HLA-DMB/HLA-DOA/HLA-DOB/HLA-DPA1/HLA-DPB1/HLA-DQA1/HLA-DQA2/HLA-DRA/HLA-DRB1/HLA-DRB5/IFNG/IL12RB1/IL12RB2/IL1A/IL4R/IL6/STAT1/STAT3/STAT4/TLR4	21/745	62/6791	1.22E-06	2.24E-05	1.64E-05
hsa05320	***Autoimmune thyroid disease***	Human Diseases	Immune disease	CD40/CD80/CD86/FAS/HLA-DMA/HLA-DMB/HLA-DOA/HLA-DOB/HLA-DPA1/HLA-DPB1/HLA-DQA1/HLA-DQA2/HLA-DRA/HLA-DRB1/HLA-DRB5/PRF1	16/745	40/6791	1.86E-06	3.02E-05	2.21E-05

hsa05322	***Systemic lupus erythematosus***	Human Diseases	Immune disease	CD40/CD80/CD86/HLA-DMA/HLA-DMB/HLA-DOA/HLA-DOB/HLA-DPA1/HLA-DPB1/HLA-DQA1/HLA-DQA2/HLA-DRA/HLA-DRB1/HLA-DRB5/IFNG	15/745	38/6791	4.77E-06	6.39E-05	4.67E-05
hsa05152	***Tuberculosis***	Human Diseases	Infectious disease: bacterial	APAF1/ARHGEF12/ATP6AP1/BAX/BCL10/BCL2/CALML6/CAMP/CIITA/CTSD/CTSS/EEA1/FCER1G/HLA-DMA/HLA-DMB/HLA-DOA/HLA-DOB/HLA-DPA1/HLA-DPB1/HLA-DQA1/HLA-DQA2/HLA-DRA/HLA-DRB1/HLA-DRB5/IFNG/IL1A/IL6/IRAK2/ITGAM/ITGAX/JAK1/KSR1/LSP1/MAPK11/MAPK13/MRC2/PLK3/RAB5B/SRC/STAT1/TLR1/TLR4/TLR6/TNFRSF1A	44/745	163/6791	6.87E-09	7.05E-07	5.16E-07
hsa05150	***Staphylococcus aureus infection***	Human Diseases	Infectious disease: bacterial	CAMP/HLA-DMA/HLA-DMB/HLA-DOA/HLA-DOB/HLA-DPA1/HLA-DPB1/HLA-DQA1/HLA-DQA2/HLA-DRA/HLA-DRB1/HLA-DRB5/ICAM1/ITGAM/KRT17/PTAFR	16/745	57/6791	2.85E-04	2.44E-03	1.78E-03

hsa05132	***Salmonella infection***	Human Diseases	Infectious disease: bacterial	BAX/BCL2/BIRC3/CASP1/CASP4/CXCL8/DCTN5/DYNC1H1/DYNC1LI2/FBXO22/GCC2/HRAS/IL6/KIF5A/MAP2K2/MAP2K6/MAPK11/MAPK13/MYL9/MYO6/NCKAP1L/NLRP3/PAK1/PIK3CG/PODXL/RAB5B/RAB9B/RHOB/RILP/RIPK3/SNX18/TAB3/TCF7/TLR4/TLR6/TNFRSF10A/TNFRSF10B/TNFRSF1A/TNFSF10/TUBB6/VPS39/VPS41	42/745	245/6791	2.00E-03	1.29E-02	9.41E-03
hsa05130	***Pathogenic Escherichia coli infection***	Human Diseases	Infectious disease: bacterial	ARHGEF12/BAIAP2L1/BAX/CASP1/CASP4/CXCL8/F2R/FAS/FYN/IL1R1/IL6/ITGB1/MAPK11/MAPK13/MYH10/MYH14/MYO1A/MYO1G/MYO5A/MYO5C/MYO6/NCKAP1L/NLRP3/PAK1/SRC/TAB3/TLR4/TNFRSF10A/TNFRSF10B/TNFRSF1A/TNFSF10/TUBB6/WIPF2	33/745	189/6791	4.36E-03	2.44E-02	1.79E-02
hsa05131	***Shigellosis***	Human Diseases	Infectious disease: bacterial	AKT1S1/ATM/BAX/BCL10/BCL2/BCL2L1/CASP1/CASP4/CCL5/CD44/CSF2/CXCL8/GABARAP/GABARAPL1/GABARAPL2/IL1R1/ITGB1/ITPR1/ITPR2/MAPK11/MAPK13/MDM2/MYL9/NLRP3/PIK3R3/PLCD3/PLCG1/PLCG2/PRKCD/PXN/RPS6KA5/SQSTM1/SRC/TAB3/TLR4/TNFRSF1A	36/745	213/6791	5.17E-03	2.70E-02	1.97E-02

hsa05145	***Toxoplasmosis***	Human Diseases	Infectious disease: parasitic	ALOX5/BCL2/BCL2L1/BIRC3/BIRC7/CD40/CIITA/GNAI1/HLA-DMA/HLA-DMB/HLA-DOA/HLA-DOB/HLA-DPA1/HLA-DPB1/HLA-DQA1/HLA-DQA2/HLA-DRA/HLA-DRB1/HLA-DRB5/IFNG/ITGB1/JAK1/LAMC1/MAP2K6/MAPK11/MAPK13/PIK3CG/SOCS1/STAT1/STAT3/TLR4/TNFRSF1A/TYK2	33/745	110/6791	3.45E-08	1.52E-06	1.11E-06
hsa05140	***Leishmaniasis***	Human Diseases	Infectious disease: parasitic	HLA-DMA/HLA-DMB/HLA-DOA/HLA-DOB/HLA-DPA1/HLA-DPB1/HLA-DQA1/HLA-DQA2/HLA-DRA/HLA-DRB1/HLA-DRB5/IFNG/IL1A/ITGAM/ITGB1/JAK1/MAPK11/MAPK13/MARCKSL1/NCF1/NCF2/STAT1/TLR4	23/745	72/6791	1.24E-06	2.24E-05	1.64E-05
hsa05164	***Influenza A***	Human Diseases	Infectious disease: viral	APAF1/BAX/CASP1/CCL5/CIITA/CXCL10/CXCL8/DDX58/FAS/HLA-DMA/HLA-DMB/HLA-DOA/HLA-DOB/HLA-DPA1/HLA-DPB1/HLA-DQA1/HLA-DQA2/HLA-DRA/HLA-DRB1/HLA-DRB5/ICAM1/IFIH1/IFNG/IL1A/IL6/JAK1/MAP2K2/NLRP3/PIK3R3/PML/RSAD2/SOCS3/STAT1/STAT2/TLR4/TNFRSF10A/TNFRSF10B/TNFRSF1A/TNFSF10/TYK2	40/745	153/6791	8.84E-08	3.40E-06	2.49E-06

hsa05169	***Epstein-Barr virus infection***	Human Diseases	Infectious disease: viral	APAF1/BAX/BCL2/CCND1/CD40/CD44/CDKN1A/CXCL10/DDB2/DDX58/FAS/GADD45A/HDAC1/HLA-DMA/HLA-DMB/HLA-DOA/HLA-DOB/HLA-DPA1/HLA-DPB1/HLA-DQA1/HLA-DQA2/HLA-DRA/HLA-DRB1/HLA-DRB5/ICAM1/IL6/JAK1/JAK3/MAP2K6/MAPK11/MAPK13/MDM2/PIK3R3/PLCG2/RB1/STAT1/STAT2/STAT3/TAP1/TNFAIP3/TYK2/VIM	42/745	189/6791	4.71E-06	6.39E-05	4.67E-05
hsa05161	***Hepatitis B***	Human Diseases	Infectious disease: viral	APAF1/ATP6AP1/BAX/BCL2/CDKN1A/CXCL8/DDB2/DDX58/EGR3/FAS/HRAS/HSPG2/IFIH1/IL6/JAK1/JAK3/MAP2K2/MAP2K6/MAPK11/MAPK13/MMP9/PCNA/PIK3R3/PTK2B/RB1/SRC/STAT1/STAT2/STAT3/STAT4/STAT5A/TGFBR1/TLR4/TYK2	34/745	152/6791	3.28E-05	3.49E-04	2.55E-04
hsa05167	***Kaposi sarcoma-associated herpesvirus infection***	Human Diseases	Infectious disease: viral	BAX/CALML6/CCND1/CCR1/CD86/CDKN1A/CSF2/CXCL8/FAS/GABARAP/GABARAPL1/GABARAPL2/GNG2/GNGT2/HCK/HIF1A/HRAS/ICAM1/IL6/ITPR1/ITPR2/JAK1/MAP2K2/MAP2K6/MAPK11/MAPK13/PIK3CG/PIK3R3/PLCG1/PLCG2/RB1/SRC/STAT1/STAT2/STAT3/TCF7/TNFRSF1A/TYK2	38/745	180/6791	4.52E-05	4.64E-04	3.39E-04

hsa05166	***Human T-cell leukemia virus 1 infection***	Human Diseases	Infectious disease: viral	ADCY1/ADCY9/ATM/BAX/BCL2L1/CCND1/CD40/CDKN1A/CRTC1/CSF2/FOSL1/HLA-DMA/HLA-DMB/HLA-DOA/HLA-DOB/HLA-DPA1/HLA-DPB1/HLA-DQA1/HLA-DQA2/HLA-DRA/HLA-DRB1/HLA-DRB5/HRAS/ICAM1/IL1R1/IL2RA/IL6/JAK1/JAK3/KAT2B/LTBR/MAD1L1/MAP2K2/PIK3R3/PTEN/RB1/SPI1/STAT5A/TGFBR1/TNFRSF1A	40/745	212/6791	3.73E-04	3.10E-03	2.27E-03
hsa05163	***Human cytomegalovirus infection***	Human Diseases	Infectious disease: viral	ADCY1/ADCY9/ARHGEF12/BAX/CALML6/CCL5/CCND1/CCR1/CDKN1A/CXCL8/CXCR2/FAS/GNAI1/GNG2/GNGT2/HRAS/IL1R1/IL6/ITGAV/ITPR1/ITPR2/JAK1/MAP2K2/MAP2K6/MAPK11/MAPK13/MDM2/PIK3R3/PTGER4/PTK2B/PXN/RB1/SRC/STAT3/TAP1/TNFRSF1A	36/745	206/6791	2.94E-03	1.85E-02	1.35E-02

hsa05168	***Herpes simplex virus 1 infection***	Human Diseases	Infectious disease: viral	APAF1/BAX/BCL2/BCL2L1/BIRC3/CCL5/DDX58/FAS/HLA-DMA/HLA-DMB/HLA-DOA/HLA-DOB/HLA-DPA1/HLA-DPB1/HLA-DQA1/HLA-DQA2/HLA-DRA/HLA-DRB1/HLA-DRB5/IFIH1/IFNG/IL6/JAK1/PIK3R3/PILRA/PML/POU2F2/SOCS3/SRC/STAT1/STAT2/TAP1/TNFRSF14/TNFRSF1A/TYK2/ZFP90/ZIK1/ZNF107/ZNF154/ZNF189/ZNF211/ZNF229/ZNF251/ZNF254/ZNF331/ZNF337/ZNF37A/ZNF425/ZNF490/ZNF506/ZNF528/ZNF530/ZNF540/ZNF547/ZNF550/ZNF561/ZNF564/ZNF585B/ZNF623/ZNF737/ZNF772/ZNF780B/ZNF786/ZNF79/ZNF793/ZNF81/ZNF83/ZNF84/ZNF846/ZNF93	70/745	467/6791	3.45E-03	2.04E-02	1.49E-02
hsa05170	***Human immunodeficiency virus 1 infection***	Human Diseases	Infectious disease: viral	AP1G2/AP1S3/APOBEC3C/APOBEC3F/APOBEC3G/APOBEC3H/ATM/BAX/BCL2/BCL2L1/CALML6/FAS/GNAI1/GNG2/GNGT2/HRAS/ITPR1/ITPR2/LIMK2/MAP2K2/MAP2K6/MAPK11/MAPK13/PAK1/PIK3R3/PLCG1/PLCG2/PTK2B/PXN/TAP1/TLR4/TNFRSF1A/TNFRSF1B	33/745	192/6791	5.59E-03	2.87E-02	2.10E-02

hsa00511	***Other glycan degradation***	Metabolism	Glycan biosynthesis and metabolism	FUCA1/HEXA/MAN2B1/MAN2B2/MANBA/NEU1/NEU3	7/745	16/6791	8.72E-04	7.07E-03	5.17E-03
hsa00531	***Glycosaminoglycan degradation***	Metabolism	Glycan biosynthesis and metabolism	GALNS/GNS/HEXA/HGSNAT/IDS/IDUA	6/745	18/6791	9.95E-03	4.79E-02	3.50E-02
hsa04380	***Osteoclast differentiation***	Organismal Systems	Development and regeneration	ACP5/CSF1/CYLD/FHL2/FOSB/FOSL1/FYN/IFNG/IL1A/IL1R1/JAK1/LCP2/LILRA4/LILRB1/LILRB3/MAP2K6/MAPK11/MAPK13/NCF1/NCF2/PIK3R3/PLCG2/SIRPA/SOCS1/SOCS3/SPI1/SQSTM1/STAT1/STAT2/TGFBR1/TNFRSF1A/TYK2	32/745	121/6791	1.33E-06	2.27E-05	1.66E-05
hsa04621	***NOD-like receptor signaling pathway***	Organismal Systems	Immune system	BCL2/BCL2L1/BIRC3/CAMP/CARD16/CARD6/CASP1/CASP4/CCL5/CTSB/CXCL8/GABARAP/GABARAPL1/GABARAPL2/GBP1/GBP2/GBP3/GBP4/GBP5/IL6/ITPR1/ITPR2/JAK1/MAPK11/MAPK13/NLRP3/NLRP7/P2RX7/PKN2/PRKCD/RIPK3/STAT1/STAT2/TAB3/TANK/TLR4/TNFAIP3/TP53BP1/TRIP6/TRPV2/TXNIP/TYK2	42/745	156/6791	1.66E-08	1.28E-06	9.34E-07

hsa04658	***Th1 and Th2 cell differentiation***	Organismal Systems	Immune system	HLA-DMA/HLA-DMB/HLA-DOA/HLA-DOB/HLA-DPA1/HLA-DPB1/HLA-DQA1/HLA-DQA2/HLA-DRA/HLA-DRB1/HLA-DRB5/IFNG/IL12RB1/IL12RB2/IL2RA/IL4R/JAG1/JAK1/JAK3/MAPK11/MAPK13/NOTCH1/NOTCH2/PLCG1/STAT1/STAT4/STAT5A/TYK2	28/745	89/6791	1.25E-07	3.93E-06	2.88E-06
hsa04640	***Hematopoietic cell lineage***	Organismal Systems	Immune system	CD1C/CD24/CD44/CD7/CD9/CSF1/CSF2/HLA-DMA/HLA-DMB/HLA-DOA/HLA-DOB/HLA-DPA1/HLA-DPB1/HLA-DQA1/HLA-DQA2/HLA-DRA/HLA-DRB1/HLA-DRB5/IL1A/IL1R1/IL2RA/IL4R/IL6/IL9R/ITGA1/ITGAM/KITLG/MS4A1	28/745	90/6791	1.63E-07	4.55E-06	3.33E-06
hsa04659	***Th17 cell differentiation***	Organismal Systems	Immune system	AHR/HIF1A/HLA-DMA/HLA-DMB/HLA-DOA/HLA-DOB/HLA-DPA1/HLA-DPB1/HLA-DQA1/HLA-DQA2/HLA-DRA/HLA-DRB1/HLA-DRB5/IFNG/IL12RB1/IL1R1/IL2RA/IL4R/IL6/JAK1/JAK3/MAPK11/MAPK13/PLCG1/RUNX1/STAT1/STAT3/STAT5A/TGFBR1/TYK2	30/745	103/6791	3.00E-07	7.10E-06	5.19E-06

hsa04672	***Intestinal immune network for IgA production***	Organismal Systems	Immune system	CD40/CD80/CD86/HLA-DMA/HLA-DMB/HLA-DOA/HLA-DOB/HLA-DPA1/HLA-DPB1/HLA-DQA1/HLA-DQA2/HLA-DRA/HLA-DRB1/HLA-DRB5/IL6/LTBR/PIGR	17/745	45/6791	2.28E-06	3.51E-05	2.57E-05
hsa04625	***C-type lectin receptor signaling pathway***	Organismal Systems	Immune system	ARHGEF12/BCL10/CALML6/CASP1/CCL22/CYLD/EGR3/FCER1G/HRAS/IL6/IRF1/ITPR1/ITPR2/KSR1/LSP1/MAPK11/MAPK13/MDM2/NLRP3/PAK1/PIK3R3/PLCG2/PLK3/PRKCD/SRC/STAT1/STAT2	27/745	101/6791	7.14E-06	8.80E-05	6.44E-05
hsa04062	***Chemokine signaling pathway***	Organismal Systems	Immune system	ADCY1/ADCY9/ARRB1/CCL1/CCL22/CCL5/CCR1/CCR7/CXCL10/CXCL8/CXCL9/CXCR2/CXCR5/FGR/GNAI1/GNG2/GNGT2/HCK/HRAS/ITK/JAK3/NCF1/PAK1/PIK3CG/PIK3R3/PLCG1/PLCG2/PRKCD/PTK2B/PXN/SRC/STAT1/STAT2/STAT3/TIAM1/VAV2	36/745	169/6791	5.86E-05	5.64E-04	4.12E-04
hsa04612	***Antigen processing and presentation***	Organismal Systems	Immune system	CIITA/CTSB/CTSS/HLA-DMA/HLA-DMB/HLA-DOA/HLA-DOB/HLA-DPA1/HLA-DPB1/HLA-DQA1/HLA-DQA2/HLA-DRA/HLA-DRB1/HLA-DRB5/IFNG/TAP1	16/745	63/6791	9.62E-04	7.40E-03	5.42E-03
hsa04664	***Fc epsilon RI signaling pathway***	Organismal Systems	Immune system	ALOX5/CSF2/FCER1G/FYN/HRAS/INPP5D/LCP2/MAP2K2/MAP2K6/MAPK11/MAPK13/PIK3R3/PLA2G4E/PLCG1/PLCG2/VAV2	16/745	63/6791	9.62E-04	7.40E-03	5.42E-03

hsa04670	***Leukocyte transendothelial migration***	Organismal Systems	Immune system	CD99/CTNND1/F11R/GNAI1/ICAM1/ITGAM/ITGB1/ITK/MAPK11/MAPK13/MMP9/MYL9/NCF1/NCF2/PIK3R3/PLCG1/PLCG2/PTK2B/PXN/TXK/VAV2/VCAM1	22/745	104/6791	1.72E-03	1.15E-02	8.40E-03
hsa04650	***Natural killer cell mediated cytotoxicity***	Organismal Systems	Immune system	CSF2/FAS/FCER1G/FYN/HRAS/ICAM1/IFNG/LCP2/MAP2K2/PAK1/PIK3R3/PLCG1/PLCG2/PRF1/PTK2B/SH3BP2/TNFRSF10A/TNFRSF10B/TNFSF10/VAV2	20/745	103/6791	7.53E-03	3.80E-02	2.78E-02
hsa04620	***Toll-like receptor signaling pathway***	Organismal Systems	Immune system	CCL5/CD40/CD80/CD86/CXCL10/CXCL8/CXCL9/IL6/IRF5/MAP2K2/MAP2K6/MAPK11/MAPK13/PIK3R3/STAT1/TLR1/TLR4/TLR6	18/745	91/6791	9.01E-03	4.48E-02	3.27E-02
hsa04750	***Inflammatory mediator regulation of TRP channels***	Organismal Systems	Sensory system	ADCY1/ADCY9/CALML6/IL1R1/ITPR1/ITPR2/MAP2K6/MAPK11/MAPK13/P2RY2/PIK3R3/PLA2G4E/PLCG1/PLCG2/PRKCD/PTGER4/SRC/TRPV1/TRPV2/TRPV3	20/745	89/6791	1.27E-03	9.28E-03	6.79E-03
**Reactome**									
R-HSA-373755	***Semaphorin interactions***	Developmental Biology	Nervous system development	ARHGEF12/DPYSL4/FES/FYN/ITGA1/ITGB1/LIMK2/MYH10/MYH14/MYL9/PAK1/PLXNB1/PLXNB3/RHOB/RHOC/SEMA6A/SEMA7A	17/939	64/10821	2.13E-05	1.57E-03	1.40E-03
R-HSA-416572	***Sema4D induced cell migration and growth-cone collapse***	Developmental Biology	Nervous system development	ARHGEF12/LIMK2/MYH10/MYH14/MYL9/PLXNB1/RHOB/RHOC	8/939	20/10821	1.52E-04	6.41E-03	5.68E-03

R-HSA-400685	***Sema4D in semaphorin signaling***	Developmental Biology	Nervous system development	ARHGEF12/LIMK2/MYH10/MYH14/MYL9/PLXNB1/RHOB/RHOC	8/939	24/10821	6.50E-04	1.67E-02	1.48E-02
R-HSA-375165	***NCAM signaling for neurite out-growth***	Developmental Biology	Nervous system development	AGRN/COL2A1/COL3A1/COL4A5/COL5A3/COL6A1/COL9A2/FYN/GDNF/HRAS/RPS6KA5/SPTBN5/SRC	13/939	63/10821	2.55E-03	4.18E-02	3.71E-02
R-HSA-3560783	***Defective B4GALT7 causes EDS, progeroid type***	Disease	Diseases of metabolism	AGRN/CSPG4/GPC5/HSPG2/SDC1/SDC4/VCAN	7/939	20/10821	1.02E-03	2.12E-02	1.88E-02
R-HSA-2206281	***Mucopolysaccharidoses***	Disease	Diseases of metabolism	GALNS/GNS/HGSNAT/IDS/IDUA	5/939	11/10821	1.44E-03	2.69E-02	2.39E-02
R-HSA-9669938	***Signaling by KIT in disease***	Disease	Diseases of signal transduction by growth factor receptors and second messengers	FYN/HRAS/PIK3R3/SRC/STAT1/STAT3/STAT5A	7/939	20/10821	1.02E-03	2.12E-02	1.88E-02
R-HSA-2022090	***Assembly of collagen fibrils and other multimeric structures***	Extracellular matrix organization	Collagen formation	BMP1/COL11A2/COL27A1/COL2A1/COL3A1/COL4A5/COL5A3/COL6A1/COL7A1/COL9A2/CTSB/CTSS/MMP9/TLL2	14/939	61/10821	5.85E-04	1.64E-02	1.45E-02
R-HSA-8948216	***Collagen chain trimerization***	Extracellular matrix organization	Collagen formation	COL11A2/COL19A1/COL23A1/COL27A1/COL2A1/COL3A1/COL4A5/COL5A3/COL6A1/COL7A1/COL9A2	11/939	44/10821	1.05E-03	2.12E-02	1.88E-02

R-HSA-1650814	***Collagen biosynthesis and modifying enzymes***	Extracellular matrix organization	Collagen formation	BMP1/COL11A2/COL19A1/COL23A1/COL27A1/COL2A1/COL3A1/COL4A5/COL5A3/COL6A1/COL7A1/COL9A2/P4HA2/TLL2	14/939	67/10821	1.56E-03	2.83E-02	2.51E-02
R-HSA-1442490	***Collagen degradation***	Extracellular matrix organization	Degradation of the extracellular matrix	COL11A2/COL19A1/COL23A1/COL2A1/COL3A1/COL4A5/COL5A3/COL6A1/COL7A1/COL9A2/CTSB/CTSD/FURIN/MMP14/MMP9	15/939	64/10821	2.95E-04	1.06E-02	9.43E-03
R-HSA-216083	***Integrin cell surface interactions***	Extracellular matrix organization		AGRN/BSG/CD44/COL23A1/COL2A1/COL3A1/COL4A5/COL5A3/COL6A1/COL7A1/COL9A2/F11R/HSPG2/ICAM1/ICAM3/ICAM4/ITGA1/ITGA10/ITGAM/ITGAV/ITGAX/ITGB1/ITGB8/TNC/VCAM1	25/939	85/10821	2.90E-08	4.18E-06	3.71E-06
R-HSA-1474244	***Extracellular matrix organization***	Extracellular matrix organization		AGRN/BMP1/BSG/CAPN3/CAPN7/CD44/CEACAM1/COL11A2/COL19A1/COL23A1/COL27A1/COL2A1/COL3A1/COL4A5/COL5A3/COL6A1/COL7A1/COL9A2/CTSB/CTSD/CTSS/DDR1/DMD/F11R/FBN2/FURIN/HSPG2/ICAM1/ICAM3/ICAM4/ITGA1/ITGA10/ITGAM/ITGAV/ITGAX/ITGB1/ITGB8/KLK2/LAMC1/LTBP3/MMP14/MMP9/NCSTN/P4HA2/PDGFA/SDC1/SDC4/SERPINE1/TIMP1/TLL2/TNC/TNXB/VCAM1/VCAN	54/939	301/10821	1.78E-07	2.25E-05	1.99E-05

R-HSA-1474228	***Degradation of the extracellular matrix***	Extracellular matrix organization		BMP1/BSG/CAPN3/CAPN7/CD44/COL11A2/COL19A1/COL23A1/COL2A1/COL3A1/COL4A5/COL5A3/COL6A1/COL7A1/COL9A2/CTSB/CTSD/CTSS/FBN2/FURIN/HSPG2/KLK2/LAMC1/MMP14/MMP9/NCSTN/TIMP1/TLL2	28/939	140/10821	2.18E-05	1.57E-03	1.40E-03
R-HSA-3000171	***Non-integrin membrane-ECM interactions***	Extracellular matrix organization		AGRN/COL11A2/COL2A1/COL3A1/COL4A5/COL5A3/DDR1/DMD/HSPG2/ITGAV/ITGB1/LAMC1/PDGFA/SDC1/SDC4/TNC	16/939	59/10821	2.80E-05	1.77E-03	1.57E-03
R-HSA-3000178	***ECM proteoglycans***	Extracellular matrix organization		AGRN/COL2A1/COL3A1/COL4A5/COL5A3/COL6A1/COL9A2/HSPG2/ITGAV/ITGAX/ITGB1/LAMC1/SERPINE1/TNC/TNXB/VCAN	16/939	76/10821	6.80E-04	1.67E-02	1.48E-02
R-HSA-1474290	***Collagen formation***	Extracellular matrix organization		BMP1/COL11A2/COL19A1/COL23A1/COL27A1/COL2A1/COL3A1/COL4A5/COL5A3/COL6A1/COL7A1/COL9A2/CTSB/CTSS/MMP9/P4HA2/TLL2	17/939	90/10821	1.69E-03	2.98E-02	2.65E-02
R-HSA-5633008	***TP53 Regulates Transcription of Cell Death Genes***	Gene expression (Transcription)	RNA Polymerase II Transcription	APAF1/ATM/BAX/BBC3/BCL2L14/CASP1/FAS/NDRG1/PERP/PIDD1/PMAIP1/RABGGTA/TNFRSF10A/TNFRSF10B/TNFRSF10C/TNFRSF10D/TP53I3/TP53INP1/TRIAP1	19/939	44/10821	9.80E-10	2.47E-07	2.19E-07

R-HSA-3700989	***Transcriptional Regulation by TP53***	Gene expression (Transcription)	RNA Polymerase II Transcription	APAF1/ATM/BAX/BBC3/BCL2L14/BTG2/CASP1/CCNG1/CDKN1A/DDB2/DDIT4/E2F7/FAS/GADD45A/GLS2/HDAC1/MAP2K6/MAPK11/MDM2/MTA2/NDRG1/PCNA/PERP/PIDD1/PIP4K2C/PLK2/PLK3/PMAIP1/PML/PRKAB1/PRKAB2/PRR5/PTEN/RABGGTA/RBL2/RICTOR/RRM2B/SESN1/SESN2/SESN3/SGK1/TFDP2/TIGAR/TNFRSF10A/TNFRSF10B/TNFRSF10C/TNFRSF10D/TP53I3/TP53INP1/TRIAP1/ZNF385A	51/939	362/10821	3.50E-04	1.14E-02	1.01E-02
R-HSA-6803211	***TP53 Regulates Transcription of Death Receptors and Ligands***	Gene expression (Transcription)	RNA Polymerase II Transcription	FAS/TNFRSF10A/TNFRSF10B/TNFRSF10C/TNFRSF10D	5/939	12/10821	2.30E-03	3.86E-02	3.42E-02
R-HSA-6791312	***TP53 Regulates Transcription of Cell Cycle Genes***	Gene expression (Transcription)	RNA Polymerase II Transcription	BAX/BTG2/CDKN1A/E2F7/GADD45A/PCNA/PLK2/PLK3/RBL2/TFDP2/ZNF385A	11/939	49/10821	2.67E-03	4.28E-02	3.79E-02
R-HSA-6804114	***TP53 Regulates Transcription of Genes Involved in G2 Cell Cycle Arrest***	Gene expression (Transcription)	RNA Polymerase II Transcription	BAX/GADD45A/PCNA/RBL2/TFDP2/ZNF385A	6/939	18/10821	3.13E-03	4.94E-02	4.38E-02
R-HSA-210990	***PECAM1 interactions***	Hemostasis	Cell surface interactions at the vascular wall	FYN/INPP5D/ITGAV/PLCG1/SRC	5/939	12/10821	2.30E-03	3.86E-02	3.42E-02

R-HSA-76002	***Platelet activation, signaling and aggregation***	Hemostasis		ARRB1/CD109/CD9/CLU/CTSW/DAGLA/DGKA/DGKZ/F2R/F5/FCER1G/FYN/GAS6/GNA15/GNAI1/GNG2/GNGT2/ITIH3/ITIH4/ITPR1/ITPR2/LCP2/LGALS3BP/PDGFA/PIK3CG/PIK3R3/PLCG2/PLEK/PRKCD/PSAP/QSOX1/RHOB/SERPINA1/SERPINE1/SRC/SRGN/TIMP1/VAV2	38/939	261/10821	1.04E-03	2.12E-02	1.88E-02
R-HSA-202433	***Generation of second messenger molecules***	Immune System	Adaptive Immune System	CD101/EVL/HLA-DPA1/HLA-DPB1/HLA-DQA1/HLA-DQA2/HLA-DRA/HLA-DRB1/HLA-DRB5/ITK/LCP2/PAK1/PLCG1/PLCG2	14/939	39/10821	2.38E-06	2.19E-04	1.94E-04
R-HSA-388841	***Costimulation by the CD28 family***	Immune System	Adaptive Immune System	CD274/CD80/CD86/FYN/HLA-DPA1/HLA-DPB1/HLA-DQA1/HLA-DQA2/HLA-DRA/HLA-DRB1/HLA-DRB5/PAK1/PDCD1LG2/PIK3R3/PRR5/RICTOR/SRC/TNFRSF14	18/939	74/10821	4.48E-05	2.45E-03	2.17E-03
R-HSA-2132295	***MHC class II antigen presentation***	Immune System	Adaptive Immune System	AP1S3/CTSA/CTSB/CTSD/CTSH/CTSO/CTSS/DCTN5/DYNC1H1/DYNC1LI2/HLA-DMA/HLA-DMB/HLA-DOA/HLA-DOB/HLA-DPA1/HLA-DPB1/HLA-DQA1/HLA-DQA2/HLA-DRA/HLA-DRB1/HLA-DRB5/KIF5A/RILP/TUBB6	24/939	126/10821	1.83E-04	7.40E-03	6.56E-03
R-HSA-389948	***PD-1 signaling***	Immune System	Adaptive Immune System	CD274/HLA-DPA1/HLA-DPB1/HLA-DQA1/HLA-DQA2/HLA-DRA/HLA-DRB1/HLA-DRB5/PDCD1LG2	9/939	28/10821	4.07E-04	1.25E-02	1.10E-02

R-HSA-877300	***Interferon gamma signaling***	Immune System	Cytokine Signaling in Immune system	CD44/CIITA/GBP1/GBP2/GBP3/GBP4/GBP5/HLA-DPA1/HLA-DPB1/HLA-DQA1/HLA-DQA2/HLA-DRA/HLA-DRB1/HLA-DRB5/ICAM1/IFNG/IRF1/IRF5/IRF6/JAK1/MT2A/PML/PRKCD/PTAFR/SOCS1/SOCS3/STAT1/TRIM22/TRIM3/TRIM35/TRIM38/VCAM1	32/939	93/10821	3.17E-12	3.20E-09	2.84E-09
R-HSA-6783783	***Interleukin-10 signaling***	Immune System	Cytokine Signaling in Immune system	CCL22/CCL5/CCR1/CD80/CD86/CSF1/CSF2/CXCL10/CXCL8/ICAM1/IL1A/IL1R1/IL6/JAK1/LIF/PTAFR/STAT3/TIMP1/TNFRSF1A/TNFRSF1B/TYK2	21/939	46/10821	3.48E-11	1.75E-08	1.55E-08
R-HSA-913531	***Interferon Signaling***	Immune System	Cytokine Signaling in Immune system	CD44/CIITA/DDX58/GBP1/GBP2/GBP3/GBP4/GBP5/HERC5/HLA-DPA1/HLA-DPB1/HLA-DQA1/HLA-DQA2/HLA-DRA/HLA-DRB1/HLA-DRB5/ICAM1/IFI27/IFI6/IFIT1/IFIT2/IFIT3/IFNG/IP6K2/IRF1/IRF5/IRF6/JAK1/MT2A/PLCG1/PML/PRKCD/PTAFR/RSAD2/SOCS1/SOCS3/STAT1/STAT2/TRIM22/TRIM3/TRIM35/TRIM38/TYK2/UBA7/VCAM1/XAF1	46/939	203/10821	8.65E-10	2.47E-07	2.19E-07

R-HSA-6785807	***Interleukin-4 and Interleukin-13 signaling***	Immune System	Cytokine Signaling in Immune system	ALOX5/BCL2/BCL2L1/CCL22/CCND1/CDKN1A/CXCL8/FSCN1/HIF1A/ICAM1/IL13RA1/IL1A/IL4R/IL6/ITGAM/ITGAX/ITGB1/JAK1/JAK3/LIF/MMP9/SOCS1/SOCS3/STAT1/STAT3/TIMP1/TNFRSF1B/TYK2/VCAM1/VIM	30/939	111/10821	1.09E-08	2.19E-06	1.95E-06
R-HSA-449147	***Signaling by Interleukins***	Immune System	Cytokine Signaling in Immune system	ALOX5/BCL2/BCL2L1/BRWD1/CASP1/CCL22/CCL5/CCND1/CCR1/CD80/CD86/CDKN1A/CLCF1/CSF1/CSF2/CXCL10/CXCL8/DUSP4/EBI3/FSCN1/FYN/HCK/HIF1A/ICAM1/IFNG/IL12RB1/IL12RB2/IL13RA1/IL16/IL17RC/IL1A/IL1R1/IL2RA/IL4R/IL6/IL9R/INPP5D/IRAK2/IRAK3/ITGAM/ITGAX/ITGB1/JAK1/JAK3/LGALS9/LIF/MAP2K6/MAPK11/MMP9/PIK3R3/PTAFR/PTK2B/PTPN14/RPS6KA1/RPS6KA5/SDC1/SOCS1/SOCS3/SQSTM1/STAT1/STAT2/STAT3/STAT4/STAT5A/TAB3/TIMP1/TNFRSF1A/TNFRSF1B/TYK2/VAMP7/VCAM1/VIM	72/939	462/10821	5.61E-07	6.28E-05	5.57E-05
R-HSA-5669034	***TNFs bind their physiological receptors***	Immune System	Cytokine Signaling in Immune system	CD70/EDA2R/EDARADD/TNFRSF14/TNFRSF18/TNFRSF1A/TNFRSF1B/TNFRSF4/TNFRSF9/TNFSF15/TNFSF4/TNFSF9	12/939	29/10821	2.19E-06	2.19E-04	1.94E-04
R-HSA-8984722	***Interleukin-35 Signalling***	Immune System	Cytokine Signaling in Immune system	EBI3/IL12RB2/JAK1/STAT1/STAT3/STAT4/TYK2	7/939	12/10821	1.94E-05	1.57E-03	1.40E-03

R-HSA-909733	***Interferon alpha/beta signaling***	Immune System	Cytokine Signaling in Immune system	GBP2/IFI27/IFI6/IFIT1/IFIT2/IFIT3/IP6K2/IRF1/IRF5/IRF6/JAK1/RSAD2/SOCS1/SOCS3/STAT1/STAT2/TYK2/XAF1	18/939	71/10821	2.46E-05	1.65E-03	1.47E-03
R-HSA-451927	***Interleukin-2 family signaling***	Immune System	Cytokine Signaling in Immune system	CSF2/IL2RA/IL9R/INPP5D/JAK1/JAK3/LGALS9/PIK3R3/PTK2B/STAT1/STAT3/STAT4/STAT5A	13/939	44/10821	5.92E-05	2.99E-03	2.65E-03
R-HSA-9020958	***Interleukin-21 signaling***	Immune System	Cytokine Signaling in Immune system	JAK1/JAK3/STAT1/STAT3/STAT4/STAT5A	6/939	10/10821	6.50E-05	3.10E-03	2.75E-03
R-HSA-1059683	***Interleukin-6 signaling***	Immune System	Cytokine Signaling in Immune system	IL6/JAK1/SOCS3/STAT1/STAT3/TYK2	6/939	11/10821	1.33E-04	5.82E-03	5.16E-03
R-HSA-8854691	***Interleukin-20 family signaling***	Immune System	Cytokine Signaling in Immune system	JAK1/JAK3/SOCS3/STAT1/STAT2/STAT3/STAT4/STAT5A/TYK2	9/939	26/10821	2.16E-04	8.38E-03	7.43E-03
R-HSA-6783589	***Interleukin-6 family signaling***	Immune System	Cytokine Signaling in Immune system	CLCF1/IL6/JAK1/LIF/SOCS3/STAT1/STAT3/TYK2	8/939	24/10821	6.50E-04	1.67E-02	1.48E-02
R-HSA-9020956	***Interleukin-27 signaling***	Immune System	Cytokine Signaling in Immune system	EBI3/JAK1/STAT1/STAT3/TYK2	5/939	11/10821	1.44E-03	2.69E-02	2.39E-02
R-HSA-9020558	***Interleukin-2 signaling***	Immune System	Cytokine Signaling in Immune system	IL2RA/JAK1/JAK3/PTK2B/STAT5A	5/939	12/10821	2.30E-03	3.86E-02	3.42E-02

R-HSA-6798695	***Neutrophil degranulation***	Immune System	Innate Immune System	ALOX5/AMPD3/APAF1/ARSA/ATP6AP2/ATP8B4/CAMP/CD44/CD53/CD68/CEACAM1/CEP290/CHI3L1/CMTM6/CPNE3/CST3/CTSA/CTSB/CTSD/CTSH/CTSS/CTSZ/CXCR2/DOK3/DYNC1H1/FCER1G/FGR/FUCA1/GALNS/GM2A/GNS/GRN/GSN/HGSNAT/ITGAM/ITGAV/ITGAX/LGALS3/LILRB3/LRG1/MAN2B1/MANBA/METTL7A/MMP9/MVP/NBEAL2/NCKAP1L/NCSTN/NEU1/NFAM1/NHLRC3/PIGR/PLAC8/PLAU/PLAUR/PLEKHO2/PRKCD/PSAP/PTAFR/QSOX1/RAB44/RAB5B/RAB9B/RAP2B/S100A11/SERPINA1/SERPINB1/SIGLEC14/SIGLEC5/SIRPA/SNAP23/STK10/STOM/TMBIM1/TMEM30A/TNFRSF1B/TSPAN14/YPEL5	78/939	479/10821	2.76E-08	4.18E-06	3.71E-06
R-HSA-168643	***Nucleotide-binding domain, leucine rich repeat containing receptor (NLR) signaling pathways***	Immune System	Innate Immune System	BCL2/BCL2L1/BIRC3/CASP1/CASP4/CYLD/IRAK2/MAP2K6/MAPK11/MAPK13/NLRP3/P2RX7/TAB3/TNFAIP3/TXNIP	15/939	55/10821	4.62E-05	2.45E-03	2.17E-03
R-HSA-168638	***NOD1/2 Signaling Pathway***	Immune System	Innate Immune System	BIRC3/CASP1/CASP4/CYLD/IRAK2/MAP2K6/MAPK11/MAPK13/TAB3/TNFAIP3	10/939	36/10821	7.24E-04	1.70E-02	1.51E-02

R-HSA-1793185	***Chondroitin sulfate/dermatan sulfate metabolism***	Metabolism	Metabolism of carbohydrates	AGRN/CHPF2/CSPG4/DSE/GPC5/HEXA/HSPG2/IDS/IDUA/SDC1/SDC4/VCAN/XYLT1	13/939	50/10821	2.48E-04	9.27E-03	8.22E-03
R-HSA-2024096	***HS-GAG degradation***	Metabolism	Metabolism of carbohydrates	AGRN/GPC5/HGSNAT/HSPG2/IDS/IDUA/SDC1/SDC4	8/939	22/10821	3.31E-04	1.13E-02	1.00E-02
R-HSA-1630316	***Glycosaminoglycan metabolism***	Metabolism	Metabolism of carbohydrates	ABCC5/AGRN/B4GAT1/CD44/CHPF2/CHST2/CHST6/CSPG4/DSE/GALNS/GNS/GPC5/HEXA/HGSNAT/HS3ST1/HSPG2/IDS/IDUA/SDC1/SDC4/SLC35D2/VCAN/XYLT1	23/939	124/10821	3.70E-04	1.17E-02	1.04E-02
R-HSA-1638091	***Heparan sulfate/heparin (HS-GAG) metabolism***	Metabolism	Metabolism of carbohydrates	AGRN/CSPG4/GPC5/HGSNAT/HS3ST1/HSPG2/IDS/IDUA/SDC1/SDC4/SLC35D2/VCAN/XYLT1	13/939	55/10821	6.75E-04	1.67E-02	1.48E-02
R-HSA-1971475	***A tetrasaccharide linker sequence is required for GAG synthesis***	Metabolism	Metabolism of carbohydrates	AGRN/CSPG4/GPC5/HSPG2/SDC1/SDC4/VCAN/XYLT1	8/939	26/10821	1.18E-03	2.34E-02	2.07E-02
R-HSA-1483249	***Inositol phosphate metabolism***	Metabolism		INPP1/INPP4A/INPP4B/INPP5D/IP6K2/IP6K3/ITPKC/PLCD3/PLCG1/PLCG2/PPIP5K1/PTEN	12/939	48/10821	6.28E-04	1.67E-02	1.48E-02
R-HSA-3371378	***Regulation by c-FLIP***	Programmed Cell Death	Apoptosis	CFLAR/FAS/TNFRSF10A/TNFRSF10B/TNFSF10	5/939	11/10821	1.44E-03	2.69E-02	2.39E-02
R-HSA-140534	***Caspase activation via Death Receptors in the presence of ligand***	Programmed Cell Death	Apoptosis	CFLAR/FAS/TLR4/TNFRSF10A/TNFRSF10B/TNFSF10	6/939	16/10821	1.57E-03	2.83E-02	2.51E-02

R-HSA-5213460	***RIPK1-mediated regulated necrosis***	Programmed Cell Death	Regulated Necrosis	BIRC3/CFLAR/FAS/RIPK3/TNFRSF10A/TNFRSF10B/TNFSF10	7/939	20/10821	1.02E-03	2.12E-02	1.88E-02
R-HSA-112409	***RAF-independent MAPK1/3 activation***	Signal Transduction	MAPK family signaling cascades	DUSP4/DUSP5/IL6/JAK1/MAP2K2/PEA15/TYK2	7/939	23/10821	2.57E-03	4.18E-02	3.71E-02
R-HSA-2122948	***Activated NOTCH1 Transmits Signal to the Nucleus***	Signal Transduction	Signaling by NOTCH	ARRB1/DTX1/DTX2/DTX4/JAG1/MIB2/NCSTN/NEURL1B/NOTCH1	9/939	31/10821	9.38E-04	2.10E-02	1.86E-02
R-HSA-194138	***Signaling by VEGF***	Signal Transduction	Signaling by Receptor Tyrosine Kinases	AXL/CTNND1/FLT1/FYN/HRAS/ITGAV/ITPR1/ITPR2/MAPK11/MAPK13/NCF1/NCF2/NCKAP1L/PAK1/PGF/PLCG1/PRKCD/PRR5/PTK2B/PXN/RICTOR/SRC/VAV2	23/939	106/10821	3.04E-05	1.80E-03	1.60E-03
R-HSA-186797	***Signaling by PDGF***	Signal Transduction	Signaling by Receptor Tyrosine Kinases	COL2A1/COL3A1/COL4A5/COL5A3/COL6A1/COL9A2/FURIN/HRAS/PDGFA/PLCG1/SRC/STAT1/STAT3/STAT5A	14/939	58/10821	3.37E-04	1.13E-02	1.00E-02
R-HSA-171007	***p38MAPK events***	Signal Transduction	Signaling by Receptor Tyrosine Kinases	HRAS/MAPK11/MAPK13/RALB/RALGDS/SRC	6/939	13/10821	4.23E-04	1.25E-02	1.11E-02
R-HSA-166520	***Signaling by NTRKs***	Signal Transduction	Signaling by Receptor Tyrosine Kinases	BAX/BDNF/DUSP4/EGR3/FOSB/FOSL1/FURIN/FYN/HRAS/ID3/MAP2K2/MAPK11/MAPK13/PLCG1/RALB/RALGDS/REST/RPS6KA1/RPS6KA5/RRAD/SGK1/SRC/STAT3/TIAM1	24/939	134/10821	4.77E-04	1.38E-02	1.22E-02
R-HSA-1433557	***Signaling by SCF-KIT***	Signal Transduction	Signaling by Receptor Tyrosine Kinases	FES/FYN/HRAS/KITLG/MMP9/PIK3R3/SOCS1/SRC/STAT1/STAT3/STAT5A	11/939	43/10821	8.52E-04	1.95E-02	1.73E-02

R-HSA-5627117	***RHO GTPases Activate ROCKs***	Signal Transduction	Signaling by Rho GTPases, Miro GTPases and RHOBTB3	LIMK2/MYH10/MYH14/MYL9/PAK1/RHOB/RHOC	7/939	19/10821	7.19E-04	1.70E-02	1.51E-02
R-HSA-73887	***Death Receptor Signalling***	Signal Transduction		ARHGEF10/ARHGEF12/ARHGEF3/ARHGEF37/BIRC3/CFLAR/CLIP3/CYLD/FAS/FGD2/FGD3/HDAC1/KALRN/NCSTN/SMPD3/SQSTM1/TAB3/TIAM1/TNFAIP3/TNFRSF10A/TNFRSF10B/TNFRSF10D/TNFRSF1A/TNFSF10/TRAF1/USP4/VAV2	27/939	141/10821	6.75E-05	3.10E-03	2.75E-03
**DOWN-regulated**									
**KEGG ID**									
hsa04110	***Cell cycle***	Cellular Processes	Cell growth and death	ANAPC11/BUB1/BUB1B/BUB3/CCNA2/CCNB1/CCNB2/CCNE1/CCNE2/CDC20/CDC25A/CDC45/CDC6/CDC7/CDK1/CDK4/CDKN2C/CDKN2D/CHEK1/DBF4/E2F1/E2F2/ESPL1/GADD45B/MAD2L1/MCM2/MCM3/MCM4/MCM5/MCM6/MCM7/MYC/ORC1/PKMYT1/PLK1/PRKDC/RAD21/RBX1/SKP2/SMC1A/SMC3/STAG1/TFDP1/YWHAE/YWHAH	45/654	124/6791	4.16E-16	1.32E-13	1.18E-13

hsa04114	***Oocyte meiosis***	Cellular Processes	Cell growth and death	ADCY3/ANAPC11/AURKA/BUB1/CALM1/CCNB1/CCNB2/CCNE1/CCNE2/CDC20/CDK1/ESPL1/FBXO43/FBXO5/ITPR3/MAD2L1/MAPK12/PKMYT1/PLK1/PPP1CC/PPP3CB/RBX1/SMC1A/SMC3/STAG3/YWHAE/YWHAH	27/654	112/6791	5.00E-06	1.59E-04	1.41E-04
hsa03060	***Protein export***	Genetic Information Processing	Folding, sorting and degradation	HSPA5/IMMP2L/SEC62/SEC63/SPCS2/SRP9/SRPRB	7/654	22/6791	3.49E-03	3.58E-02	3.19E-02
hsa03030	***DNA replication***	Genetic Information Processing	Replication and repair	DNA2/FEN1/MCM2/MCM3/MCM4/MCM5/MCM6/MCM7/POLA1/POLD1/POLD2/POLD3/POLE/POLE2/RFC2/RFC4/RFC5/RNASEH1/RNASEH2A/RNASEH2C/RPA3/SSBP1	22/654	36/6791	3.24E-14	3.43E-12	3.06E-12
hsa03410	***Base excision repair***	Genetic Information Processing	Replication and repair	APEX1/FEN1/HMGB1/LIG3/NEIL3/NTHL1/PARP1/PARP2/POLD1/POLD2/POLD3/POLE/POLE2/UNG	14/654	33/6791	7.42E-07	3.37E-05	3.00E-05
hsa03430	***Mismatch repair***	Genetic Information Processing	Replication and repair	EXO1/MSH2/MSH6/POLD1/POLD2/POLD3/RFC2/RFC4/RFC5/RPA3/SSBP1	11/654	23/6791	2.79E-06	9.85E-05	8.77E-05
hsa03420	***Nucleotide excision repair***	Genetic Information Processing	Replication and repair	POLD1/POLD2/POLD3/POLE/POLE2/RAD23A/RAD23B/RBX1/RFC2/RFC4/RFC5/RPA3	12/654	44/6791	6.72E-04	1.02E-02	9.06E-03
hsa03450	***Non-homologous end-joining***	Genetic Information Processing	Replication and repair	FEN1/NHEJ1/PRKDC/XRCC5/XRCC6	5/654	Dec.91	3.63E-03	3.61E-02	3.22E-02

hsa03040	***Spliceosome***	Genetic Information Processing	Transcription	ALYREF/CCDC12/CHERP/DHX15/EIF4A3/FUS/HNRNPA3/HNRNPC/HNRNPM/HNRNPU/LSM4/LSM7/MAGOHB/PCBP1/PHF5A/PPIH/PRPF19/PRPF40B/PUF60/RBMX/SF3A2/SF3B2/SF3B5/SNRNP40/SNRNP70/SNRPA/SNRPA1/SNRPB/SNRPC/SNRPD1/SNRPD2/SNRPE/SNRPF/SNRPG/SNU13/SRSF1/SRSF2/SRSF3/SRSF4/SRSF7/SRSF9/TCERG1/TRA2B/TXNL4A/U2AF2	45/654	136/6791	2.18E-14	3.43E-12	3.06E-12
hsa03013	***RNA transport***	Genetic Information Processing	Translation	AAAS/ALYREF/EIF1AX/EIF2S2/EIF4A3/EIF4E/EIF4EBP1/EIF4G1/ELAC2/FUS/GEMIN2/GEMIN4/KPNB1/MAGOHB/NDC1/NUP107/NUP153/NUP155/NUP35/NUP50/NUP85/NUP88/NXT1/PABPC4/POP7/PRMT5/RAN/RANGAP1/RNPS1/RPP25/SEC13/SEH1L/SNUPN/SRRM1/TACC3/THOC6/UBE2I/XPOT	38/654	157/6791	5.28E-08	3.36E-06	2.99E-06
hsa03008	***Ribosome biogenesis in eukaryotes***	Genetic Information Processing	Translation	DKC1/EMG1/FBL/GAR1/GNL3/IMP4/NHP2/NOP56/NOP58/NXT1/POP7/RAN/REXO1/RPP25/RRP7A/SNU13/TCOF1/UTP18	18/654	76/6791	2.38E-04	4.74E-03	4.22E-03
hsa00970	***Aminoacyl-tRNA biosynthesis***	Genetic Information Processing	Translation	DARS2/FARS2/FARSA/FARSB/MARS2/NARS2/SARS2/YARS2	8/654	25/6791	1.74E-03	2.12E-02	1.89E-02

hsa03010	***Ribosome***	Genetic Information Processing	Translation	MRPL11/MRPL12/MRPL14/MRPL16/MRPL17/MRPL2/MRPL20/MRPL22/MRPL23/MRPL24/MRPL3/MRPL34/MRPL36/MRPL4/MRPL9/MRPS12/MRPS15/MRPS17/MRPS2/RPL17/RPL35/RPS12/RPS26	23/654	130/6791	2.84E-03	3.11E-02	2.77E-02
hsa05415	***Diabetic cardiomyopathy***	Human Diseases	Cardiovascular disease	AKT1/COX5A/COX6A1/COX7B/CYC1/GAPDH/IRS1/MAPK12/MAPK9/NDUFA7/NDUFAB1/NDUFB10/NDUFB6/NDUFS6/NDUFS7/NDUFS8/NDUFV2/PARP1/PDHA1/PDHB/PPIF/PPP1CC/RAC1/SLC25A5/UQCRC1/UQCRQ/VDAC1	27/654	167/6791	4.78E-03	4.61E-02	4.10E-02
hsa04932	***Non-alcoholic fatty liver disease***	Human Diseases	Endocrine and metabolic disease	AKT1/ATF4/COX5A/COX6A1/COX7B/CYC1/CYCS/IL6R/IRS1/LEPR/MAPK12/MAPK9/NDUFA7/NDUFAB1/NDUFB10/NDUFB6/NDUFS6/NDUFS7/NDUFS8/NDUFV2/RAC1/SREBF1/UQCRC1/UQCRQ/XBP1	25/654	145/6791	2.74E-03	3.11E-02	2.77E-02

hsa05014	***Amyotrophic lateral sclerosis***	Human Diseases	Neurodegenerative disease	ACTB/ACTG1/ALYREF/ATF4/ATXN2L/CHCHD10/COX5A/COX6A1/COX7B/CYC1/CYCS/FUS/HNRNPA2B1/HNRNPA3/HSPA5/ITPR3/MAPK12/NDC1/NDUFA7/NDUFAB1/NDUFB10/NDUFB6/NDUFS6/NDUFS7/NDUFS8/NDUFV2/NUP107/NUP153/NUP155/NUP35/NUP50/NUP85/NUP88/NXT1/PFN1/PPP3CB/PSMA3/PSMA5/PSMA7/PSMB2/PSMB6/PSMC3/PSMD3/RAC1/SEC13/SEH1L/SIGMAR1/SRSF3/SRSF7/TOMM40/TUBA1B/TUBA1C/TUBB/TUBB4B/UQCRC1/UQCRQ/VDAC1/XBP1	58/654	323/6791	1.54E-06	6.11E-05	5.44E-05
hsa05012	***Parkinson disease***	Human Diseases	Neurodegenerative disease	ATF4/CALM1/COX5A/COX6A1/COX7B/CYC1/CYCS/HSPA5/ITPR3/MAPK9/NDUFA7/NDUFAB1/NDUFB10/NDUFB6/NDUFS6/NDUFS7/NDUFS8/NDUFV2/PPIF/PSMA3/PSMA5/PSMA7/PSMB2/PSMB6/PSMC3/PSMD3/SLC25A5/TRAP1/TUBA1B/TUBA1C/TUBB/TUBB4B/UBE2J1/UCHL1/UQCRC1/UQCRQ/VDAC1/XBP1	38/654	212/6791	1.07E-04	2.83E-03	2.52E-03

hsa05020	***Prion disease***	Human Diseases	Neurodegenerative disease	ATF4/COX5A/COX6A1/COX7B/CYC1/CYCS/HSPA5/ITPR3/MAPK12/MAPK9/NDUFA7/NDUFAB1/NDUFB10/NDUFB6/NDUFS6/NDUFS7/NDUFS8/NDUFV2/PPIF/PPP3CB/PSMA3/PSMA5/PSMA7/PSMB2/PSMB6/PSMC3/PSMD3/RAC1/SLC25A5/STIP1/TUBA1B/TUBA1C/TUBB/TUBB4B/UQCRC1/UQCRQ/VDAC1	37/654	233/6791	1.46E-03	1.86E-02	1.66E-02
hsa00270	***Cysteine and methionine metabolism***	Metabolism	Amino acid metabolism	AMD1/CTH/DNMT1/GOT2/LDHA/LDHB/MDH2/MTAP/PHGDH/PSAT1/SRM/TST	12/654	45/6791	8.37E-04	1.21E-02	1.08E-02
hsa00010	***Glycolysis / Gluconeogenesis***	Metabolism	Carbohydrate metabolism	ALDH1B1/ALDOA/ALDOC/DLAT/ENO1/GAPDH/GPI/HK2/LDHA/LDHB/PCK2/PDHA1/PDHB/PGAM1/PGK1/PGM1/TPI1	17/654	61/6791	3.99E-05	1.15E-03	1.03E-03
hsa00020	***Citrate cycle (TCA cycle)***	Metabolism	Carbohydrate metabolism	ACLY/CS/DLAT/FH/IDH1/MDH2/PCK2/PDHA1/PDHB/SUCLG2	10/654	29/6791	2.35E-04	4.74E-03	4.22E-03
hsa00030	***Pentose phosphate pathway***	Metabolism	Carbohydrate metabolism	ALDOA/ALDOC/GPI/PGD/PGM1/PRPS1/PRPS2/RPIA/TALDO1/TKT	10/654	29/6791	2.35E-04	4.74E-03	4.22E-03
hsa00051	***Fructose and mannose metabolism***	Metabolism	Carbohydrate metabolism	AKR1B1/ALDOA/ALDOC/ENOSF1/GMDS/GMPPB/HK2/KHK/PFKFB4/TPI1	10/654	30/6791	3.22E-04	6.03E-03	5.37E-03
hsa00620	***Pyruvate metabolism***	Metabolism	Carbohydrate metabolism	ACACA/ACAT2/ALDH1B1/DLAT/FH/LDHA/LDHB/MDH2/ME2/PCK2/PDHA1/PDHB	12/654	42/6791	4.22E-04	7.24E-03	6.45E-03

hsa01200	***Carbon metabolism***	Metabolism	Global and overview maps	ACAT2/ALDOA/ALDOC/CS/DLAT/ENO1/FH/GAPDH/GCSH/GLUD1/GOT2/GPI/GPT2/HK2/IDH1/MDH2/ME2/PCCA/PDHA1/PDHB/PGAM1/PGD/PGK1/PGP/PHGDH/PRPS1/PRPS2/PSAT1/RPIA/SHMT2/SUCLG2/TALDO1/TKT/TPI1	34/654	111/6791	3.86E-10	3.07E-08	2.73E-08
hsa01230	***Biosynthesis of amino acids***	Metabolism	Global and overview maps	ALDOA/ALDOC/ASNS/CS/CTH/ENO1/GAPDH/GOT2/GPT2/IDH1/PGAM1/PGK1/PHGDH/PRPS1/PRPS2/PSAT1/PYCR1/RPIA/SHMT2/TALDO1/TKT/TPI1	22/654	70/6791	3.07E-07	1.63E-05	1.45E-05
hsa00100	***Steroid biosynthesis***	Metabolism	Lipid metabolism	CYP51A1/DHCR7/EBP/FDFT1/LBR/LSS/MSMO1/SQLE	8/654	19/6791	2.04E-04	4.74E-03	4.22E-03
hsa00670	***One carbon pool by folate***	Metabolism	Metabolism of cofactors and vitamins	ALDH1L2/ATIC/DHFR/GART/MTHFD1/MTHFD1L/SHMT2	7/654	19/6791	1.33E-03	1.77E-02	1.57E-02
hsa00900	***Terpenoid backbone biosynthesis***	Metabolism	Metabolism of terpenoids and polyketides	ACAT2/FDPS/HMGCR/HMGCS1/ICMT/MVD/MVK	7/654	21/6791	2.59E-03	3.05E-02	2.72E-02
hsa00240	***Pyrimidine metabolism***	Metabolism	Nucleotide metabolism	CAD/CTPS1/DCTPP1/DHODH/DTYMK/DUT/NME4/NT5C/NT5C1A/NT5M/RRM1/RRM2/TK1/UCK2	14/654	54/6791	4.33E-04	7.24E-03	6.45E-03
hsa00230	***Purine metabolism***	Metabolism	Nucleotide metabolism	ADCY3/ADK/AK2/APRT/ATIC/FHIT/GART/GMPS/HPRT1/IMPDH1/NME4/NT5C/NT5C1A/NT5M/PAICS/PDE4A/PFAS/PGM1/PPAT/PRPS1/PRPS2/RRM1/RRM2	23/654	117/6791	6.52E-04	1.02E-02	9.06E-03

hsa04914	***Progesterone-mediated oocyte maturation***	Organismal Systems	Endocrine system	ADCY3/AKT1/ANAPC11/AURKA/BUB1/CCNA2/CCNB1/CCNB2/CDC25A/CDK1/HSP90AB1/KIF22/MAD2L1/MAPK12/MAPK9/PKMYT1/PLK1	17/654	87/6791	3.40E-03	3.58E-02	3.19E-02
hsa04714	***Thermogenesis***	Organismal Systems	Environmental adaptation	ACSL3/ACTB/ACTG1/ACTL6A/ADCY3/BMP8B/COA7/COX10/COX17/COX18/COX5A/COX6A1/COX7B/CYC1/KDM1A/LIPE/MAPK12/MLST8/NDUFA7/NDUFAB1/NDUFAF2/NDUFAF3/NDUFAF4/NDUFB10/NDUFB6/NDUFS6/NDUFS7/NDUFS8/NDUFV2/SMARCA4/UQCRC1/UQCRQ	32/654	191/6791	1.23E-03	1.70E-02	1.51E-02
**REACTOME**									
R-HSA-69618	***Mitotic Spindle Checkpoint***	Cell Cycle	Cell Cycle Checkpoints	ANAPC11/ANAPC15/AURKB/BIRC5/BUB1/BUB1B/BUB3/CDC20/CDCA8/CENPA/CENPE/CENPF/CENPM/ERCC6L/INCENP/KNTC1/MAD2L1/NDC80/NUDC/NUF2/NUP107/NUP85/PLK1/PPP1CC/RANGAP1/SEC13/SEH1L/SKA1/SPC25/SPDL1/UBE2C/UBE2S/ZW10/ZWINT	34/908	111/10821	1.08E-11	6.21E-10	4.81E-10
R-HSA-176187	***Activation of ATR in response to replication stress***	Cell Cycle	Cell Cycle Checkpoints	CDC25A/CDC45/CDC6/CDC7/CHEK1/CLSPN/DBF4/MCM10/MCM2/MCM3/MCM4/MCM5/MCM6/MCM7/ORC1/RFC2/RFC4/RFC5/RPA3	19/908	37/10821	1.23E-11	6.65E-10	5.16E-10

R-HSA-69481	***G2/M Checkpoints***	Cell Cycle	Cell Cycle Checkpoints	BARD1/CCNB1/CCNB2/CDC25A/CDC45/CDC6/CDC7/CDK1/CHEK1/CLSPN/DBF4/DNA2/EXO1/GTSE1/KAT5/MCM10/MCM2/MCM3/MCM4/MCM5/MCM6/MCM7/ORC1/PKMYT1/PSMA3/PSMA5/PSMA7/PSMB2/PSMB6/PSMC3/PSMD3/RBBP8/RFC2/RFC4/RFC5/RPA3/YWHAE/YWHAH	38/908	168/10821	1.09E-08	3.46E-07	2.68E-07
R-HSA-141405	***Inhibition of the proteolytic activity of APC/C required for the onset of anaphase by mitotic spindle checkpoint components***	Cell Cycle	Cell Cycle Checkpoints	ANAPC11/ANAPC15/BUB1B/BUB3/CDC20/MAD2L1/UBE2C/UBE2S	8/908	21/10821	1.80E-04	1.46E-03	1.13E-03
R-HSA-75035	***Chk1/Chk2(Cds1) mediated inactivation of Cyclin B:Cdk1 complex***	Cell Cycle	Cell Cycle Checkpoints	CCNB1/CDK1/CHEK1/YWHAE/YWHAH	5/908	13/10821	2.99E-03	1.64E-02	1.27E-02
R-HSA-69615	***G1/S DNA Damage Checkpoints***	Cell Cycle	Cell Cycle Checkpoints	CCNA2/CCNE1/CCNE2/CDC25A/CHEK1/PSMA3/PSMA5/PSMA7/PSMB2/PSMB6/PSMC3/PSMD3	12/908	68/10821	1.03E-02	4.91E-02	3.81E-02

R-HSA-68886	***M Phase***	Cell Cycle	Cell Cycle, Mitotic	AAAS/ALMS1/ANAPC11/ANAPC15/AURKB/BANF1/BIRC5/BUB1/BUB1B/BUB3/CCNB1/CCNB2/CDC20/CDCA5/CDCA8/CDK1/CENPA/CENPE/CENPF/CENPM/CEP41/CTDNEP1/ERCC6L/ESPL1/FBXO5/HAUS7/INCENP/KIF20A/KIF23/KNTC1/KPNB1/LBR/LMNB1/MAD2L1/MASTL/MZT2B/NCAPD2/NCAPD3/NCAPG/NCAPG2/NCAPH/NDC1/NDC80/NUDC/NUF2/NUP107/NUP153/NUP155/NUP35/NUP50/NUP85/NUP88/PLK1/PLK4/PPP1CC/PSMA3/PSMA5/PSMA7/PSMB2/PSMB6/PSMC3/PSMD3/RAB1B/RAD21/RAN/RANGAP1/RCC1/SEC13/SEH1L/SET/SKA1/SMC1A/SMC2/SMC3/SMC4/SPC25/SPDL1/SSNA1/STAG1/TMPO/TUBA1B/TUBA1C/TUBB/TUBB4B/TUBG1/TUBGCP3/TUBGCP4/UBE2C/UBE2I/UBE2S/YWHAE/ZW10/ZWINT	93/908	417/10821	4.02E-19	9.26E-17	7.18E-17

R-HSA-69242	***S Phase***	Cell Cycle	Cell Cycle, Mitotic	AKT1/ANAPC11/ANAPC15/CABLES1/CCNA2/CCNE1/CCNE2/CDC25A/CDC45/CDC6/CDCA5/CDK4/CKS1B/DNA2/E2F1/ESCO2/FEN1/GINS2/GINS3/MCM2/MCM3/MCM4/MCM5/MCM6/MCM7/MYC/ORC1/POLA1/POLD1/POLD2/POLD3/POLE/POLE2/PSMA3/PSMA5/PSMA7/PSMB2/PSMB6/PSMC3/PSMD3/RAD21/RBX1/RFC2/RFC4/RFC5/RPA3/SKP2/SMC1A/SMC3/STAG1/TFDP1/UBE2C/UBE2S	53/908	162/10821	6.19E-19	1.14E-16	8.84E-17
R-HSA-2555396	***Mitotic Metaphase and Anaphase***	Cell Cycle	Cell Cycle, Mitotic	ANAPC11/ANAPC15/AURKB/BANF1/BIRC5/BUB1/BUB1B/BUB3/CCNB1/CCNB2/CDC20/CDCA5/CDCA8/CDK1/CENPA/CENPE/CENPF/CENPM/ERCC6L/ESPL1/FBXO5/INCENP/KNTC1/KPNB1/LBR/LMNB1/MAD2L1/NDC1/NDC80/NUDC/NUF2/NUP107/NUP155/NUP35/NUP85/PLK1/PPP1CC/PSMA3/PSMA5/PSMA7/PSMB2/PSMB6/PSMC3/PSMD3/RAD21/RAN/RANGAP1/RCC1/SEC13/SEH1L/SKA1/SMC1A/SMC3/SPC25/SPDL1/STAG1/TMPO/TUBA1B/TUBA1C/TUBB4B/UBE2C/UBE2I/UBE2S/ZW10/ZWINT	65/908	236/10821	1.58E-18	2.43E-16	1.89E-16

R-HSA-68877	***Mitotic Prometaphase***	Cell Cycle	Cell Cycle, Mitotic	ALMS1/AURKB/BIRC5/BUB1/BUB1B/BUB3/CCNB1/CCNB2/CDC20/CDCA5/CDCA8/CDK1/CENPA/CENPE/CENPF/CENPM/CEP41/ERCC6L/HAUS7/INCENP/KNTC1/MAD2L1/MZT2B/NCAPD2/NCAPG/NCAPH/NDC80/NUDC/NUF2/NUP107/NUP85/PLK1/PLK4/PPP1CC/RAD21/RANGAP1/SEC13/SEH1L/SKA1/SMC1A/SMC2/SMC3/SMC4/SPC25/SPDL1/SSNA1/STAG1/TUBA1B/TUBA1C/TUBB/TUBB4B/TUBG1/TUBGCP3/TUBGCP4/YWHAE/ZW10/ZWINT	57/908	203/10821	8.41E-17	9.69E-15	7.51E-15
R-HSA-453279	***Mitotic G1 phase and G1/S transition***	Cell Cycle	Cell Cycle, Mitotic	AKT1/CABLES1/CCNA2/CCNB1/CCNE1/CCNE2/CDC25A/CDC45/CDC6/CDC7/CDK1/CDK4/CDKN2C/CDKN2D/CKS1B/DBF4/DHFR/E2F1/E2F2/FBXO5/GMNN/MCM10/MCM2/MCM3/MCM4/MCM5/MCM6/MCM7/MYBL2/MYC/ORC1/POLA1/POLE/POLE2/PSMA3/PSMA5/PSMA7/PSMB2/PSMB6/PSMC3/PSMD3/RPA3/RRM2/SKP2/TFDP1/TK1	46/908	149/10821	1.67E-15	1.71E-13	1.32E-13

R-HSA-2500257	***Resolution of Sister Chromatid Cohesion***	Cell Cycle	Cell Cycle, Mitotic	AURKB/BIRC5/BUB1/BUB1B/BUB3/CCNB1/CCNB2/CDC20/CDCA5/CDCA8/CDK1/CENPA/CENPE/CENPF/CENPM/ERCC6L/INCENP/KNTC1/MAD2L1/NDC80/NUDC/NUF2/NUP107/NUP85/PLK1/PPP1CC/RAD21/RANGAP1/SEC13/SEH1L/SKA1/SMC1A/SMC3/SPC25/SPDL1/STAG1/TUBA1B/TUBA1C/TUBB4B/ZW10/ZWINT	41/908	126/10821	7.75E-15	7.14E-13	5.54E-13
R-HSA-2467813	***Separation of Sister Chromatids***	Cell Cycle	Cell Cycle, Mitotic	ANAPC11/ANAPC15/AURKB/BIRC5/BUB1/BUB1B/BUB3/CDC20/CDCA5/CDCA8/CENPA/CENPE/CENPF/CENPM/ERCC6L/ESPL1/INCENP/KNTC1/MAD2L1/NDC80/NUDC/NUF2/NUP107/NUP85/PLK1/PPP1CC/PSMA3/PSMA5/PSMA7/PSMB2/PSMB6/PSMC3/PSMD3/RAD21/RANGAP1/SEC13/SEH1L/SKA1/SMC1A/SMC3/SPC25/SPDL1/STAG1/TUBA1B/TUBA1C/TUBB4B/UBE2C/UBE2S/ZW10/ZWINT	50/908	191/10821	1.41E-13	1.19E-11	9.19E-12
R-HSA-2980766	***Nuclear Envelope Breakdown***	Cell Cycle	Cell Cycle, Mitotic	AAAS/BANF1/CCNB1/CCNB2/CDK1/CTDNEP1/LMNB1/NDC1/NUP107/NUP153/NUP155/NUP35/NUP50/NUP85/NUP88/PLK1/SEC13/SEH1L/TMPO	19/908	53/10821	2.26E-08	6.14E-07	4.76E-07

R-HSA-2995410	***Nuclear Envelope (NE) Reassembly***	Cell Cycle	Cell Cycle, Mitotic	BANF1/CCNB1/CCNB2/CDK1/KPNB1/LBR/LMNB1/NDC1/NUP107/NUP155/NUP35/NUP85/RAN/RANGAP1/RCC1/SEC13/SEH1L/TMPO/TUBA1B/TUBA1C/TUBB4B/UBE2I	22/908	76/10821	1.48E-07	3.04E-06	2.36E-06
R-HSA-69205	***G1/S-Specific Transcription***	Cell Cycle	Cell Cycle, Mitotic	CCNE1/CDC25A/CDC45/CDC6/CDK1/DHFR/E2F1/FBXO5/ORC1/POLA1/RRM2/TFDP1/TK1	13/908	29/10821	1.79E-07	3.51E-06	2.72E-06
R-HSA-2514853	***Condensation of Prometaphase Chromosomes***	Cell Cycle	Cell Cycle, Mitotic	CCNB1/CCNB2/CDK1/NCAPD2/NCAPG/NCAPH/SMC2/SMC4	8/908	11/10821	3.13E-07	5.66E-06	4.39E-06
R-HSA-3301854	***Nuclear Pore Complex (NPC) Disassembly***	Cell Cycle	Cell Cycle, Mitotic	AAAS/CCNB1/CCNB2/CDK1/NDC1/NUP107/NUP153/NUP155/NUP35/NUP50/NUP85/NUP88/SEC13/SEH1L	14/908	36/10821	5.10E-07	8.71E-06	6.75E-06
R-HSA-174143	***APC/C-mediated degradation of cell cycle proteins***	Cell Cycle	Cell Cycle, Mitotic	ANAPC11/ANAPC15/AURKA/AURKB/BUB1B/BUB3/CCNA2/CCNB1/CDC20/CDK1/FBXO5/MAD2L1/PLK1/PSMA3/PSMA5/PSMA7/PSMB2/PSMB6/PSMC3/PSMD3/SKP2/UBE2C/UBE2S	23/908	88/10821	5.93E-07	9.75E-06	7.56E-06
R-HSA-9615933	***Postmitotic nuclear pore complex (NPC) reformation***	Cell Cycle	Cell Cycle, Mitotic	KPNB1/NDC1/NUP107/NUP155/NUP35/NUP85/RAN/RANGAP1/RCC1/SEC13/SEH1L/UBE2I	12/908	27/10821	6.03E-07	9.75E-06	7.56E-06

R-HSA-453274	***Mitotic G2-G2/M phases***	Cell Cycle	Cell Cycle, Mitotic	ALMS1/AURKA/CCNA2/CCNB1/CCNB2/CDC25A/CDK1/CENPF/CEP41/E2F1/FOXM1/GTSE1/HAUS7/HMMR/HSP90AB1/MYBL2/MZT2B/PKMYT1/PLK1/PLK4/PSMA3/PSMA5/PSMA7/PSMB2/PSMB6/PSMC3/PSMD3/RBX1/SSNA1/TPX2/TUBA1B/TUBA1C/TUBB/TUBB4B/TUBG1/TUBGCP3/TUBGCP4/YWHAE	38/908	200/10821	1.30E-06	1.99E-05	1.54E-05
R-HSA-156711	***Polo-like kinase mediated events***	Cell Cycle	Cell Cycle, Mitotic	CCNB1/CCNB2/CDC25A/CENPF/FOXM1/MYBL2/PKMYT1/PLK1	8/908	16/10821	1.67E-05	1.86E-04	1.44E-04
R-HSA-68884	***Mitotic Telophase/Cytokinesis***	Cell Cycle	Cell Cycle, Mitotic	KIF20A/KIF23/PLK1/RAD21/SMC1A/SMC3/STAG1	7/908	13/10821	3.13E-05	3.28E-04	2.54E-04
R-HSA-1538133	***G0 and Early G1***	Cell Cycle	Cell Cycle, Mitotic	CCNA2/CCNE1/CCNE2/CDC25A/CDC6/CDK1/E2F1/MYBL2/MYC/TFDP1	10/908	27/10821	3.70E-05	3.71E-04	2.87E-04
R-HSA-69656	***Cyclin A:Cdk2-associated events at S phase entry***	Cell Cycle	Cell Cycle, Mitotic	AKT1/CABLES1/CCNA2/CCNE1/CCNE2/CDC25A/CDK4/CKS1B/E2F1/MYC/PSMA3/PSMA5/PSMA7/PSMB2/PSMB6/PSMC3/PSMD3/SKP2/TFDP1	19/908	85/10821	6.10E-05	5.74E-04	4.45E-04
R-HSA-2995383	***Initiation of Nuclear Envelope (NE) Reformation***	Cell Cycle	Cell Cycle, Mitotic	BANF1/CCNB1/CCNB2/CDK1/KPNB1/LBR/LMNB1/TMPO	8/908	19/10821	7.79E-05	7.18E-04	5.56E-04
R-HSA-2468052	***Establishment of Sister Chromatid Cohesion***	Cell Cycle	Cell Cycle, Mitotic	CDCA5/ESCO2/RAD21/SMC1A/SMC3/STAG1	6/908	11/10821	1.10E-04	9.46E-04	7.33E-04

R-HSA-68875	***Mitotic Prophase***	Cell Cycle	Cell Cycle, Mitotic	AAAS/BANF1/CCNB1/CCNB2/CDK1/CTDNEP1/LMNB1/MASTL/NCAPD3/NCAPG2/NDC1/NUP107/NUP153/NUP155/NUP35/NUP50/NUP85/NUP88/PLK1/RAB1B/SEC13/SEH1L/SET/SMC2/SMC4/TMPO	26/908	143/10821	1.29E-04	1.08E-03	8.36E-04
R-HSA-174178	***APC/C:Cdh1 mediated degradation of Cdc20 and other APC/C:Cdh1 targeted proteins in late mitosis/early G1***	Cell Cycle	Cell Cycle, Mitotic	ANAPC11/ANAPC15/AURKA/AURKB/CDC20/PLK1/PSMA3/PSMA5/PSMA7/PSMB2/PSMB6/PSMC3/PSMD3/SKP2/UBE2C/UBE2S	16/908	74/10821	3.42E-04	2.65E-03	2.06E-03
R-HSA-69273	***Cyclin A/B1/B2 associated events during G2/M transition***	Cell Cycle	Cell Cycle, Mitotic	CCNA2/CCNB1/CCNB2/CDC25A/CDK1/FOXM1/PKMYT1/PLK1	8/908	25/10821	7.07E-04	4.98E-03	3.86E-03
R-HSA-8852276	***The role of GTSE1 in G2/M progression after G2 checkpoint***	Cell Cycle	Cell Cycle, Mitotic	CCNB1/CCNB2/CDK1/GTSE1/HSP90AB1/PLK1/PSMA3/PSMA5/PSMA7/PSMB2/PSMB6/PSMC3/PSMD3/TUBA1B/TUBA1C/TUBB4B	16/908	79/10821	7.38E-04	5.12E-03	3.97E-03
R-HSA-176412	***Phosphorylation of the APC/C***	Cell Cycle	Cell Cycle, Mitotic	ANAPC11/ANAPC15/CCNB1/CDK1/PLK1/UBE2C/UBE2S	7/908	20/10821	8.37E-04	5.76E-03	4.47E-03
R-HSA-179409	***APC-Cdc20 mediated degradation of Nek2A***	Cell Cycle	Cell Cycle, Mitotic	ANAPC11/ANAPC15/BUB1B/BUB3/CDC20/MAD2L1/UBE2C/UBE2S	8/908	26/10821	9.47E-04	6.29E-03	4.87E-03

R-HSA-187577	***SCF(Skp2)-mediated degradation of p27/p21***	Cell Cycle	Cell Cycle, Mitotic	CCNA2/CCNE1/CCNE2/CDK4/CKS1B/PSMA3/PSMA5/PSMA7/PSMB2/PSMB6/PSMC3/PSMD3/SKP2	13/908	60/10821	1.18E-03	7.60E-03	5.89E-03
R-HSA-8854518	***AURKA Activation by TPX2***	Cell Cycle	Cell Cycle, Mitotic	ALMS1/AURKA/CDK1/CEP41/HAUS7/HMMR/PLK1/PLK4/SSNA1/TPX2/TUBB/TUBB4B/TUBG1/YWHAE	14/908	72/10821	2.33E-03	1.33E-02	1.03E-02
R-HSA-174048	***APC/C:Cdc20 mediated degradation of Cyclin B***	Cell Cycle	Cell Cycle, Mitotic	ANAPC11/ANAPC15/CCNB1/CDC20/CDK1/UBE2C/UBE2S	7/908	24/10821	2.78E-03	1.54E-02	1.20E-02
R-HSA-162658	***Golgi Cisternae Pericentriolar Stack Reorganization***	Cell Cycle	Cell Cycle, Mitotic	CCNB1/CCNB2/CDK1/PLK1/RAB1B	5/908	14/10821	4.33E-03	2.28E-02	1.77E-02
R-HSA-69231	***Cyclin D associated events in G1***	Cell Cycle	Cell Cycle, Mitotic	CCNE1/CCNE2/CDK4/CDKN2C/CDKN2D/CKS1B/E2F1/E2F2/SKP2/TFDP1	10/908	47/10821	4.87E-03	2.54E-02	1.97E-02
R-HSA-380320	***Recruitment of NuMA to mitotic centrosomes***	Cell Cycle	Cell Cycle, Mitotic	ALMS1/CDK1/CEP41/HAUS7/MZT2B/PLK1/PLK4/SSNA1/TUBA1B/TUBA1C/TUBB/TUBB4B/TUBG1/TUBGCP3/TUBGCP4/YWHAE	16/908	95/10821	5.34E-03	2.77E-02	2.14E-02
R-HSA-4419969	***Depolymerisation of the Nuclear Lamina***	Cell Cycle	Cell Cycle, Mitotic	CCNB1/CDK1/CTDNEP1/LMNB1/TMPO	5/908	15/10821	6.06E-03	3.12E-02	2.42E-02
R-HSA-2470946	***Cohesin Loading onto Chromatin***	Cell Cycle	Cell Cycle, Mitotic	RAD21/SMC1A/SMC3/STAG1	4/908	10/10821	6.85E-03	3.47E-02	2.69E-02
R-HSA-380270	***Recruitment of mitotic centrosome proteins and complexes***	Cell Cycle	Cell Cycle, Mitotic	ALMS1/CDK1/CEP41/HAUS7/MZT2B/PLK1/PLK4/SSNA1/TUBB/TUBB4B/TUBG1/TUBGCP3/TUBGCP4/YWHAE	14/908	81/10821	7.03E-03	3.53E-02	2.73E-02

R-HSA-113510	***E2F mediated regulation of DNA replication***	Cell Cycle	Cell Cycle, Mitotic	CCNB1/CDK1/E2F1/ORC1/POLA1/TFDP1	6/908	22/10821	7.95E-03	3.92E-02	3.04E-02
R-HSA-1362300	***Transcription of E2F targets under negative control by p107 (RBL1) and p130 (RBL2) in complex with HDAC1***	Cell Cycle	Cell Cycle, Mitotic	CCNA2/CDK1/E2F1/MYBL2/TFDP1	5/908	16/10821	8.21E-03	4.03E-02	3.12E-02
R-HSA-180786	***Extension of Telomeres***	Cell Cycle	Chromosome Maintenance	CCNA2/DKC1/DNA2/FEN1/GAR1/NHP2/PIF1/POLA1/POLD1/POLD2/POLD3/RFC2/RFC4/RFC5/RPA3/RUVBL1/RUVBL2/WRAP53	18/908	51/10821	7.01E-08	1.58E-06	1.22E-06
R-HSA-174437	***Removal of the Flap Intermediate from the C-strand***	Cell Cycle	Chromosome Maintenance	DNA2/FEN1/POLD1/POLD2/POLD3/RPA3	6/908	11/10821	1.10E-04	9.46E-04	7.33E-04
R-HSA-157579	***Telomere Maintenance***	Cell Cycle	Chromosome Maintenance	CCNA2/DKC1/DNA2/FEN1/GAR1/NHP2/PIF1/POLA1/POLD1/POLD2/POLD3/POLR2E/POLR2F/POLR2H/POLR2I/RFC2/RFC4/RFC5/RPA3/RUVBL1/RUVBL2/WRAP53	22/908	113/10821	1.50E-04	1.23E-03	9.57E-04
R-HSA-174417	***Telomere C-strand (Lagging Strand) Synthesis***	Cell Cycle	Chromosome Maintenance	DNA2/FEN1/POLA1/POLD1/POLD2/POLD3/RFC2/RFC4/RFC5/RPA3	10/908	34/10821	3.34E-04	2.61E-03	2.03E-03
R-HSA-171319	***Telomere Extension By Telomerase***	Cell Cycle	Chromosome Maintenance	CCNA2/DKC1/GAR1/NHP2/PIF1/RUVBL1/RUVBL2/WRAP53	8/908	23/10821	3.73E-04	2.82E-03	2.18E-03

R-HSA-174414	***Processive synthesis on the C-strand of the telomere***	Cell Cycle	Chromosome Maintenance	DNA2/FEN1/POLD1/POLD2/POLD3/RPA3	6/908	19/10821	3.59E-03	1.94E-02	1.50E-02
R-HSA-174411	***Polymerase switching on the C-strand of the telomere***	Cell Cycle	Chromosome Maintenance	POLA1/POLD1/POLD2/POLD3/RFC2/RFC4/RFC5	7/908	26/10821	4.55E-03	2.39E-02	1.85E-02
R-HSA-69620	***Cell Cycle Checkpoints***	Cell Cycle		ANAPC11/ANAPC15/AURKB/BARD1/BIRC5/BUB1/BUB1B/BUB3/CCNA2/CCNB1/CCNB2/CCNE1/CCNE2/CDC20/CDC25A/CDC45/CDC6/CDC7/CDCA8/CDK1/CENPA/CENPE/CENPF/CENPM/CHEK1/CLSPN/DBF4/DNA2/ERCC6L/EXO1/GTSE1/INCENP/KAT5/KNTC1/MAD2L1/MCM10/MCM2/MCM3/MCM4/MCM5/MCM6/MCM7/NDC80/NUDC/NUF2/NUP107/NUP85/ORC1/PKMYT1/PLK1/PPP1CC/PSMA3/PSMA5/PSMA7/PSMB2/PSMB6/PSMC3/PSMD3/RANGAP1/RBBP8/RFC2/RFC4/RFC5/RPA3/SEC13/SEH1L/SKA1/SPC25/SPDL1/UBE2C/UBE2S/YWHAE/YWHAH/ZW10/ZWINT	75/908	292/10821	2.80E-19	8.61E-17	6.67E-17

R-HSA-73886	***Chromosome Maintenance***	Cell Cycle		CCNA2/CENPA/CENPM/DKC1/DNA2/FEN1/GAR1/HJURP/NHP2/NPM1/PIF1/POLA1/POLD1/POLD2/POLD3/POLR2E/POLR2F/POLR2H/POLR2I/RBBP7/RFC2/RFC4/RFC5/RPA3/RUVBL1/RUVBL2/WRAP53	27/908	140/10821	3.31E-05	3.43E-04	2.66E-04
R-HSA-9648895	***Response of EIF2AK1 (HRI) to heme deficiency***	Cellular responses to external stimuli	Cellular responses to stress	ASNS/ATF4/CEBPG/CHAC1/EIF2S2/TRIB3	6/908	15/10821	8.87E-04	5.97E-03	4.63E-03
R-HSA-3371556	***Cellular response to heat stress***	Cellular responses to external stimuli	Cellular responses to stress	AAAS/FKBP4/HSP90AB1/HSPA5/MLST8/NDC1/NUP107/NUP153/NUP155/NUP35/NUP50/NUP85/NUP88/PTGES3/RPA3/SEC13/SEH1L/YWHAE	18/908	101/10821	1.70E-03	1.02E-02	7.94E-03
R-HSA-3371453	***Regulation of HSF1-mediated heat shock response***	Cellular responses to external stimuli	Cellular responses to stress	AAAS/FKBP4/HSPA5/NDC1/NUP107/NUP153/NUP155/NUP35/NUP50/NUP85/NUP88/RPA3/SEC13/SEH1L/YWHAE	15/908	82/10821	3.08E-03	1.68E-02	1.30E-02
R-HSA-2559585	***Oncogene Induced Senescence***	Cellular responses to external stimuli	Cellular responses to stress	CDK4/CDKN2C/CDKN2D/E2F1/E2F2/ETS1/ETS2/TFDP1	8/908	35/10821	7.27E-03	3.60E-02	2.79E-02
R-HSA-9659787	***Aberrant regulation of mitotic G1/S transition in cancer due to RB1 defects***	Disease	Diseases of mitotic cell cycle	CCNE1/CCNE2/CDK4/E2F1/E2F2/TFDP1	6/908	17/10821	1.89E-03	1.12E-02	8.68E-03

R-HSA-177243	***Interactions of Rev with host cellular proteins***	Disease	Infectious disease	AAAS/KPNB1/NDC1/NPM1/NUP107/NUP153/NUP155/NUP35/NUP50/NUP85/NUP88/RAN/RANBP1/RANGAP1/RCC1/SEC13/SEH1L	17/908	37/10821	1.39E-09	6.05E-08	4.69E-08
R-HSA-162909	***Host Interactions of HIV factors***	Disease	Infectious disease	AAAS/ARF1/BANF1/HMGA1/KPNB1/NDC1/NPM1/NUP107/NUP153/NUP155/NUP35/NUP50/NUP85/NUP88/PPIA/PSIP1/PSMA3/PSMA5/PSMA7/PSMB2/PSMB6/PSMC3/PSMD3/RAC1/RAN/RANBP1/RANGAP1/RBX1/RCC1/SEC13/SEH1L/SLC25A5	32/908	131/10821	2.26E-08	6.14E-07	4.76E-07
R-HSA-180746	***Nuclear import of Rev protein***	Disease	Infectious disease	AAAS/KPNB1/NDC1/NPM1/NUP107/NUP153/NUP155/NUP35/NUP50/NUP85/NUP88/RAN/RCC1/SEC13/SEH1L	15/908	34/10821	2.60E-08	6.86E-07	5.32E-07
R-HSA-162906	***HIV Infection***	Disease	Infectious disease	AAAS/ARF1/BANF1/FEN1/GTF2E2/HMGA1/KPNB1/NDC1/NELFB/NPM1/NUP107/NUP153/NUP155/NUP35/NUP50/NUP85/NUP88/POLR2E/POLR2F/POLR2H/POLR2I/PPIA/PSIP1/PSMA3/PSMA5/PSMA7/PSMB2/PSMB6/PSMC3/PSMD3/RAC1/RAN/RANBP1/RANGAP1/RBX1/RCC1/SEC13/SEH1L/SLC25A5/SSRP1/SUPT16H/TAF15/TAF5/XRCC5/XRCC6	45/908	231/10821	6.40E-08	1.51E-06	1.17E-06

R-HSA-176033	***Interactions of Vpr with host cellular proteins***	Disease	Infectious disease	AAAS/BANF1/HMGA1/NDC1/NUP107/NUP153/NUP155/NUP35/NUP50/NUP85/NUP88/PSIP1/SEC13/SEH1L/SLC25A5	15/908	37/10821	1.03E-07	2.22E-06	1.72E-06
R-HSA-162587	***HIV Life Cycle***	Disease	Infectious disease	AAAS/BANF1/FEN1/GTF2E2/HMGA1/NDC1/NELFB/NUP107/NUP153/NUP155/NUP35/NUP50/NUP85/NUP88/POLR2E/POLR2F/POLR2H/POLR2I/PPIA/PSIP1/RAN/RANBP1/RANGAP1/RCC1/SEC13/SEH1L/SSRP1/SUPT16H/TAF15/TAF5/XRCC5/XRCC6	32/908	149/10821	5.48E-07	9.19E-06	7.13E-06
R-HSA-168325	***Viral Messenger RNA Synthesis***	Disease	Infectious disease	AAAS/NDC1/NUP107/NUP153/NUP155/NUP35/NUP50/NUP85/NUP88/POLR2E/POLR2F/POLR2H/POLR2I/SEC13/SEH1L	15/908	44/10821	1.45E-06	2.15E-05	1.67E-05
R-HSA-168276	***NS1 Mediated Effects on Host Pathways***	Disease	Infectious disease	AAAS/KPNA2/KPNA3/KPNB1/NDC1/NUP107/NUP153/NUP155/NUP35/NUP50/NUP85/NUP88/SEC13/SEH1L	14/908	41/10821	3.18E-06	4.44E-05	3.44E-05
R-HSA-168274	***Export of Viral Ribonucleoproteins from Nucleus***	Disease	Infectious disease	AAAS/NDC1/NUP107/NUP153/NUP155/NUP35/NUP50/NUP85/NUP88/RAN/SEC13/SEH1L	12/908	33/10821	7.66E-06	9.54E-05	7.40E-05
R-HSA-162594	***Early Phase of HIV Life Cycle***	Disease	Infectious disease	BANF1/FEN1/HMGA1/PPIA/PSIP1/XRCC5/XRCC6	7/908	14/10821	5.80E-05	5.57E-04	4.32E-04
R-HSA-168255	***Influenza Infection***	Disease	Infectious disease	AAAS/IPO5/KPNA2/KPNA3/KPNB1/NDC1/NUP107/NUP153/NUP155/NUP35/NUP50/NUP85/NUP88/PARP1/POLR2E/POLR2F/POLR2H/POLR2I/RAN/RPL17/RPL35/RPS12/RPS26/SEC13/SEH1L	25/908	157/10821	1.33E-03	8.29E-03	6.43E-03

R-HSA-9675126	***Diseases of mitotic cell cycle***	Disease		ANAPC11/ANAPC15/CCNE1/CCNE2/CDK4/E2F1/E2F2/SKP2/TFDP1/UBE2C/UBE2S	11/908	36/10821	1.16E-04	9.88E-04	7.66E-04
R-HSA-9645723	***Diseases of programmed cell death***	Disease		C1QBP/CDC25A/CDK5R1/LMNB1/PRDX2/SOD2/YWHAE	7/908	24/10821	2.78E-03	1.54E-02	1.20E-02
R-HSA-9675135	***Diseases of DNA repair***	Disease		MSH2/MSH6/NEIL3/NTHL1	4/908	10/10821	6.85E-03	3.47E-02	2.69E-02
R-HSA-110373	***Resolution of AP sites via the multiple-nucleotide patch replacement pathway***	DNA Repair	Base Excision Repair	APEX1/FEN1/PARP1/PARP2/POLD1/POLD2/POLD3/POLE/POLE2/RFC2/RFC4/RFC5/RPA3	13/908	25/10821	1.88E-08	5.60E-07	4.34E-07
R-HSA-73933	***Resolution of Abasic Sites (AP sites)***	DNA Repair	Base Excision Repair	APEX1/FEN1/LIG3/NTHL1/PARP1/PARP2/POLD1/POLD2/POLD3/POLE/POLE2/RFC2/RFC4/RFC5/RPA3/UNG	16/908	38/10821	2.02E-08	5.81E-07	4.50E-07
R-HSA-5651801	***PCNA-Dependent Long Patch Base Excision Repair***	DNA Repair	Base Excision Repair	APEX1/FEN1/POLD1/POLD2/POLD3/POLE/POLE2/RFC2/RFC4/RFC5/RPA3	11/908	21/10821	2.20E-07	4.14E-06	3.21E-06
R-HSA-110314	***Recognition of DNA damage by PCNA-containing replication complex***	DNA Repair	DNA Damage Bypass	DTL/POLD1/POLD2/POLD3/POLE/POLE2/RBX1/RFC2/RFC4/RFC5/RPA3/USP1	12/908	30/10821	2.37E-06	3.46E-05	2.68E-05
R-HSA-5656169	***Termination of translesion DNA synthesis***	DNA Repair	DNA Damage Bypass	POLD1/POLD2/POLD3/POLE/POLE2/RFC2/RFC4/RFC5/RPA3	9/908	32/10821	9.48E-04	6.29E-03	4.87E-03

R-HSA-110313	***Translesion synthesis by Y family DNA polymerases bypasses lesions on DNA template***	DNA Repair	DNA Damage Bypass	POLD1/POLD2/POLD3/POLE/POLE2/RFC2/RFC4/RFC5/RPA3	9/908	39/10821	4.21E-03	2.24E-02	1.74E-02
R-HSA-5685942	***HDR through Homologous Recombination (HRR)R_***	DNA Repair	DNA Double-Strand Break Repair	BARD1/CHEK1/DNA2/EXO1/KAT5/POLD1/POLD2/POLD3/POLE/POLE2/RBBP8/RFC2/RFC4/RFC5/RPA3	15/908	67/10821	3.49E-04	2.68E-03	2.08E-03
R-HSA-5685939	***HDR through MMEJ (alt-NHEJ)***	DNA Repair	DNA Double-Strand Break Repair	FEN1/LIG3/PARP1/PARP2/RBBP8	5/908	10/10821	7.25E-04	5.06E-03	3.92E-03
R-HSA-5693538	***Homology Directed Repair***	DNA Repair	DNA Double-Strand Break Repair	BARD1/CCNA2/CHEK1/CLSPN/DNA2/EXO1/FEN1/KAT5/LIG3/PARP1/PARP2/POLD1/POLD2/POLD3/POLE/POLE2/RBBP8/RFC2/RFC4/RFC5/RPA3/TIPIN/UBE2I	23/908	138/10821	1.09E-03	7.19E-03	5.58E-03
R-HSA-5693579	***Homologous DNA Pairing and Strand Exchange***	DNA Repair	DNA Double-Strand Break Repair	BARD1/CHEK1/DNA2/EXO1/KAT5/RBBP8/RFC2/RFC4/RFC5/RPA3	10/908	42/10821	2.03E-03	1.18E-02	9.17E-03
R-HSA-5685938	***HDR through Single Strand Annealing (SSA)***	DNA Repair	DNA Double-Strand Break Repair	BARD1/DNA2/EXO1/KAT5/RBBP8/RFC2/RFC4/RFC5/RPA3	9/908	37/10821	2.87E-03	1.59E-02	1.23E-02
R-HSA-5696399	***Global Genome Nucleotide Excision Repair (GG-NER)***	DNA Repair	Nucleotide Excision Repair	ACTB/ACTL6A/GPS1/LIG3/MCRS1/PARP1/PARP2/POLD1/POLD2/POLD3/POLE/POLE2/RAD23A/RAD23B/RBX1/RFC2/RFC4/RFC5/RPA3/RUVBL1/UBE2I	21/908	84/10821	3.95E-06	5.28E-05	4.10E-05

R-HSA-5696397	***Gap-filling DNA repair synthesis and ligation in GG-NER***	DNA Repair	Nucleotide Excision Repair	LIG3/POLD1/POLD2/POLD3/POLE/POLE2/RFC2/RFC4/RFC5/RPA3	10/908	25/10821	1.67E-05	1.86E-04	1.44E-04
R-HSA-6781827	***Transcription-Coupled Nucleotide Excision Repair (TC-NER)***	DNA Repair	Nucleotide Excision Repair	GPS1/HMGN1/LIG3/POLD1/POLD2/POLD3/POLE/POLE2/POLR2E/POLR2F/POLR2H/POLR2I/PRPF19/RBX1/RFC2/RFC4/RFC5/RPA3/USP7	19/908	78/10821	1.69E-05	1.86E-04	1.44E-04
R-HSA-6782135	***Dual incision in TC-NER***	DNA Repair	Nucleotide Excision Repair	HMGN1/POLD1/POLD2/POLD3/POLE/POLE2/POLR2E/POLR2F/POLR2H/POLR2I/PRPF19/RBX1/RFC2/RFC4/RFC5/RPA3/USP7	17/908	65/10821	1.72E-05	1.86E-04	1.44E-04
R-HSA-5696400	***Dual Incision in GG-NER***	DNA Repair	Nucleotide Excision Repair	PARP1/PARP2/POLD1/POLD2/POLD3/POLE/POLE2/RBX1/RFC2/RFC4/RFC5/RPA3	12/908	41/10821	9.09E-05	8.21E-04	6.37E-04
R-HSA-5696394	***DNA Damage Recognition in GG-NER***	DNA Repair	Nucleotide Excision Repair	ACTB/ACTL6A/GPS1/MCRS1/PARP1/PARP2/RAD23A/RAD23B/RBX1/RUVBL1	10/908	38/10821	8.86E-04	5.97E-03	4.63E-03

R-HSA-73894	***DNA Repair***	DNA Repair	ACTB/ACTL6A/ALKBH2/ALKBH5/APEX1/BARD1/CCNA2/CHEK1/CLSPN/DNA2/DTL/EXO1/FANCA/FANCF/FEN1/GPS1/HMGN1/KAT5/KPNA2/LIG3/MCRS1/MSH2/MSH6/NEIL3/NHEJ1/NTHL1/PARP1/PARP2/PAXIP1/POLD1/POLD2/POLD3/POLE/POLE2/POLR2E/POLR2F/POLR2H/POLR2I/PPP5C/PRKDC/PRPF19/RAD23A/RAD23B/RBBP8/RBX1/RFC2/RFC4/RFC5/RPA3/RUVBL1/TDP1/TIPIN/UBE2I/UBE2T/UNG/USP1/USP7/XRCC5/XRCC6	59/908	331/10821	1.80E-08	5.54E-07	4.29E-07
R-HSA-5696398	***Nucleotide Excision Repair***	DNA Repair	ACTB/ACTL6A/GPS1/HMGN1/LIG3/MCRS1/PARP1/PARP2/POLD1/POLD2/POLD3/POLE/POLE2/POLR2E/POLR2F/POLR2H/POLR2I/PRPF19/RAD23A/RAD23B/RBX1/RFC2/RFC4/RFC5/RPA3/RUVBL1/UBE2I/USP7	28/908	110/10821	6.65E-08	1.53E-06	1.19E-06
R-HSA-5693532	***DNA Double-Strand Break Repair***	DNA Repair	BARD1/CCNA2/CHEK1/CLSPN/DNA2/EXO1/FEN1/KAT5/KPNA2/LIG3/NHEJ1/PARP1/PARP2/PAXIP1/POLD1/POLD2/POLD3/POLE/POLE2/PPP5C/PRKDC/RBBP8/RFC2/RFC4/RFC5/RPA3/TDP1/TIPIN/UBE2I/XRCC5/XRCC6	31/908	166/10821	1.74E-05	1.86E-04	1.44E-04
R-HSA-5358508	***Mismatch Repair***	DNA Repair	EXO1/MSH2/MSH6/POLD1/POLD2/POLD3/RPA3	7/908	15/10821	1.01E-04	8.87E-04	6.87E-04

R-HSA-73893	***DNA Damage Bypass***	DNA Repair		DTL/POLD1/POLD2/POLD3/POLE/POLE2/RBX1/RFC2/RFC4/RFC5/RPA3/USP1	12/908	48/10821	4.63E-04	3.42E-03	2.65E-03
R-HSA-73884	***Base Excision Repair***	DNA Repair		APEX1/FEN1/LIG3/NEIL3/NTHL1/PARP1/PARP2/POLD1/POLD2/POLD3/POLE/POLE2/RFC2/RFC4/RFC5/RPA3/UNG	17/908	91/10821	1.33E-03	8.29E-03	6.43E-03
R-HSA-69002	***Activation of the pre-replicative complex***	DNA Replication	DNA Replication Pre-Initiation	CDC45/CDC6/CDC7/DBF4/GMNN/MCM10/MCM2/MCM3/MCM4/MCM5/MCM6/MCM7/ORC1/POLA1/POLE/POLE2/RPA3	17/908	33/10821	1.41E-10	7.22E-09	5.60E-09
R-HSA-68867	***Assembly of the pre-replicative complex***	DNA Replication	DNA Replication Pre-Initiation	CDC6/E2F1/E2F2/GMNN/MCM2/MCM3/MCM4/MCM5/MCM6/MCM7/ORC1/PSMA3/PSMA5/PSMA7/PSMB2/PSMB6/PSMC3/PSMD3	18/908	68/10821	8.13E-06	9.99E-05	7.74E-05
R-HSA-68689	***CDC6 association with the ORC:origin complex***	DNA Replication	DNA Replication Pre-Initiation	CDC6/E2F1/E2F2/ORC1	4/908	11/10821	1.01E-02	4.86E-02	3.77E-02
R-HSA-69190	***DNA strand elongation***	DNA Replication	Synthesis of DNA	CDC45/DNA2/FEN1/GINS2/GINS3/MCM2/MCM3/MCM4/MCM5/MCM6/MCM7/POLA1/POLD1/POLD2/POLD3/RFC2/RFC4/RFC5/RPA3	19/908	32/10821	3.62E-13	2.57E-11	1.99E-11
R-HSA-176974	***Unwinding of DNA***	DNA Replication	Synthesis of DNA	CDC45/GINS2/GINS3/MCM2/MCM3/MCM4/MCM5/MCM6/MCM7	9/908	12/10821	3.46E-08	8.63E-07	6.69E-07

R-HSA-69052	***Switching of origins to a post-replicative state***	DNA Replication	Synthesis of DNA	ANAPC11/ANAPC15/CCNA2/CCNE1/CCNE2/CDC6/MCM2/MCM3/MCM4/MCM5/MCM6/MCM7/ORC1/PSMA3/PSMA5/PSMA7/PSMB2/PSMB6/PSMC3/PSMD3/RBX1/SKP2/UBE2C/UBE2S	24/908	91/10821	2.82E-07	5.19E-06	4.03E-06
R-HSA-69186	***Lagging Strand Synthesis***	DNA Replication	Synthesis of DNA	DNA2/FEN1/POLA1/POLD1/POLD2/POLD3/RFC2/RFC4/RFC5/RPA3	10/908	20/10821	1.40E-06	2.11E-05	1.64E-05
R-HSA-68949	***Orc1 removal from chromatin***	DNA Replication	Synthesis of DNA	CCNA2/CDC6/MCM2/MCM3/MCM4/MCM5/MCM6/MCM7/ORC1/PSMA3/PSMA5/PSMA7/PSMB2/PSMB6/PSMC3/PSMD3/RBX1/SKP2	18/908	71/10821	1.56E-05	1.77E-04	1.37E-04
R-HSA-69091	***Polymerase switching***	DNA Replication	Synthesis of DNA	POLA1/POLD1/POLD2/POLD3/RFC2/RFC4/RFC5	7/908	14/10821	5.80E-05	5.57E-04	4.32E-04
R-HSA-69183	***Processive synthesis on the lagging strand***	DNA Replication	Synthesis of DNA	DNA2/FEN1/POLA1/POLD1/POLD2/POLD3/RPA3	7/908	15/10821	1.01E-04	8.87E-04	6.87E-04
R-HSA-69306	***DNA Replication***	DNA Replication		ANAPC11/ANAPC15/CCNA2/CCNE1/CCNE2/CDC45/CDC6/CDC7/DBF4/DNA2/E2F1/E2F2/FEN1/GINS2/GINS3/GMNN/MCM10/MCM2/MCM3/MCM4/MCM5/MCM6/MCM7/ORC1/POLA1/POLD1/POLD2/POLD3/POLE/POLE2/PSMA3/PSMA5/PSMA7/PSMB2/PSMB6/PSMC3/PSMD3/RBX1/RFC2/RFC4/RFC5/RPA3/SKP2/UBE2C/UBE2S	45/908	128/10821	1.25E-17	1.64E-15	1.27E-15

R-HSA-69002	***DNA Replication Pre-Initiation***	DNA Replication		CDC45/CDC6/CDC7/DBF4/E2F1/E2F2/GMNN/MCM10/MCM2/MCM3/MCM4/MCM5/MCM6/MCM7/ORC1/POLA1/POLE/POLE2/PSMA3/PSMA5/PSMA7/PSMB2/PSMB6/PSMC3/PSMD3/RPA3	26/908	85/10821	3.02E-09	1.16E-07	9.01E-08
R-HSA-203927	***MicroRNA (miRNA) biogenesis***	Gene expression (Transcription)	Gene Silencing by RNA	DGCR8/POLR2E/POLR2F/POLR2H/POLR2I/PRKRA/RAN	7/908	25/10821	3.58E-03	1.94E-02	1.50E-02
R-HSA-73856	***RNA Polymerase II Transcription Termination***	Gene expression (Transcription)	RNA Polymerase II Transcription	ALYREF/CHTOP/DDX39A/EIF4A3/MAGOHB/RNPS1/SARNP/SLBP/SNRPB/SNRPE/SNRPF/SNRPG/SRRM1/SRSF1/SRSF2/SRSF3/SRSF4/SRSF7/SRSF9/SYMPK/THOC6/U2AF2	22/908	66/10821	8.39E-09	2.87E-07	2.22E-07
R-HSA-3700989	***Transcriptional Regulation by TP53***	Gene expression (Transcription)	RNA Polymerase II Transcription	AKT1/AURKA/AURKB/BARD1/BIRC5/CARM1/CCNA2/CCNB1/CCNE1/CCNE2/CDK1/CDK5R1/CHD3/CHEK1/COX18/COX5A/COX6A1/COX7B/CYCS/DNA2/E2F1/E2F8/EXO1/GPI/KAT5/MBD3/MLST8/MSH2/NELFB/NOC2L/NPM1/POLR2E/POLR2F/POLR2H/POLR2I/PRDX2/PRELID1/PRMT1/PRMT5/RABGGTB/RBBP7/RBBP8/RFC2/RFC4/RFC5/RPA3/SSRP1/SUPT16H/TAF15/TAF5/TFDP1/TPX2/USP7/YWHAE/YWHAH	55/908	362/10821	1.00E-05	1.22E-04	9.44E-05

R-HSA-6804756	***Regulation of TP53 Activity through Phosphorylation***	Gene expression (Transcription)	RNA Polymerase II Transcription	AURKA/AURKB/BARD1/CCNA2/CDK5R1/CHEK1/DNA2/EXO1/KAT5/NOC2L/RBBP8/RFC2/RFC4/RFC5/RPA3/SSRP1/SUPT16H/TAF15/TAF5/TPX2	20/908	92/10821	6.00E-05	5.70E-04	4.42E-04
R-HSA-5633007	***Regulation of TP53 Activity***	Gene expression (Transcription)	RNA Polymerase II Transcription	AKT1/AURKA/AURKB/BARD1/CCNA2/CDK1/CDK5R1/CHD3/CHEK1/DNA2/EXO1/KAT5/MBD3/MLST8/NOC2L/PRMT5/RBBP7/RBBP8/RFC2/RFC4/RFC5/RPA3/SSRP1/SUPT16H/TAF15/TAF5/TPX2/USP7	28/908	160/10821	1.43E-04	1.19E-03	9.19E-04
R-HSA-6791312	***TP53 Regulates Transcription of Cell Cycle Genes***	Gene expression (Transcription)	RNA Polymerase II Transcription	AURKA/CARM1/CCNA2/CCNB1/CCNE1/CCNE2/CDK1/E2F1/E2F8/NPM1/PRMT1/TFDP1	12/908	49/10821	5.68E-04	4.08E-03	3.16E-03
R-HSA-8953750	***Transcriptional Regulation by E2F6***	Gene expression (Transcription)	RNA Polymerase II Transcription	CBX3/CDC7/CHEK1/E2F1/EZH2/RBBP7/RBBP8/RRM2/TFDP1	9/908	34/10821	1.52E-03	9.35E-03	7.25E-03
R-HSA-6804114	***TP53 Regulates Transcription of Genes Involved in G2 Cell Cycle Arrest***	Gene expression (Transcription)	RNA Polymerase II Transcription	AURKA/CARM1/CCNB1/CDK1/PRMT1/TFDP1	6/908	18/10821	2.64E-03	1.49E-02	1.15E-02
R-HSA-6804116	***TP53 Regulates Transcription of Genes Involved in G1 Cell Cycle Arrest***	Gene expression (Transcription)	RNA Polymerase II Transcription	CCNA2/CCNE1/CCNE2/E2F1/E2F8	5/908	14/10821	4.33E-03	2.28E-02	1.77E-02

R-HSA-73980	***RNA Polymerase III Transcription Termination***	Gene expression (Transcription)	RNA Polymerase III Transcription	NFIX/POLR2E/POLR2F/POLR2H/POLR3E/POLR3H/POLR3K/SSB	8/908	23/10821	3.73E-04	2.82E-03	2.18E-03
R-HSA-76071	***RNA Polymerase III Transcription Initiation From Type 3 Promoter***	Gene expression (Transcription)	RNA Polymerase III Transcription	POLR2E/POLR2F/POLR2H/POLR3E/POLR3H/POLR3K/POU2F1/SNAPC2	8/908	28/10821	1.62E-03	9.90E-03	7.67E-03
R-HSA-73780	***RNA Polymerase III Chain Elongation***	Gene expression (Transcription)	RNA Polymerase III Transcription	POLR2E/POLR2F/POLR2H/POLR3E/POLR3H/POLR3K	6/908	18/10821	2.64E-03	1.49E-02	1.15E-02
R-HSA-76061	***RNA Polymerase III Transcription Initiation From Type 1 Promoter***	Gene expression (Transcription)	RNA Polymerase III Transcription	GTF3A/POLR2E/POLR2F/POLR2H/POLR3E/POLR3H/POLR3K	7/908	28/10821	7.07E-03	3.53E-02	2.73E-02
R-HSA-74158	***RNA Polymerase III Transcription***	Gene expression (Transcription)		GTF3A/NFIX/POLR2E/POLR2F/POLR2H/POLR3E/POLR3H/POLR3K/POU2F1/SNAPC2/SSB	11/908	41/10821	4.12E-04	3.07E-03	2.38E-03
R-HSA-983189	***Kinesins***	Hemostasis	Factors involved in megakaryocyte development and platelet production	CENPE/KIF11/KIF15/KIF18B/KIF20A/KIF22/KIF23/KIF4A/KIFC1/RACGAP1/TUBA1B/TUBA1C/TUBB4B	13/908	61/10821	1.39E-03	8.58E-03	6.65E-03
R-HSA-8950505	***Gene and protein expression by JAK-STAT signaling after Interleukin-12 stimulation***	Immune System	Cytokine Signaling in Immune system	ANXA2/ARF1/CFL1/HNRNPA2B1/HNRNPDL/HNRNPF/IL10/LMNB1/MIF/MTAP/PPIA/SNRPA1/SOD2/TALDO1/TCP1	15/908	38/10821	1.58E-07	3.16E-06	2.45E-06

R-HSA-9020591	***Interleukin-12 signaling***	Immune System	Cytokine Signaling in Immune system	ANXA2/ARF1/CFL1/HNRNPA2B1/HNRNPDL/HNRNPF/IL10/LMNB1/MIF/MTAP/PPIA/SNRPA1/SOD2/TALDO1/TCP1	15/908	47/10821	3.73E-06	5.05E-05	3.92E-05
R-HSA-447115	***Interleukin-12 family signaling***	Immune System	Cytokine Signaling in Immune system	ANXA2/ARF1/CFL1/HNRNPA2B1/HNRNPDL/HNRNPF/IL10/LMNB1/MIF/MTAP/PPIA/SNRPA1/SOD2/TALDO1/TCP1	15/908	57/10821	4.93E-05	4.89E-04	3.79E-04
R-HSA-1169410	***Antiviral mechanism by IFN-stimulated genes***	Immune System	Cytokine Signaling in Immune system	AAAS/EIF4A3/EIF4E/EIF4G1/KPNA2/KPNA3/KPNB1/NDC1/NUP107/NUP153/NUP155/NUP35/NUP50/NUP85/NUP88/SEC13/SEH1L	17/908	82/10821	3.83E-04	2.87E-03	2.22E-03
R-HSA-351202	***Metabolism of polyamines***	Metabolism	Metabolism of amino acids and derivatives	AMD1/OAZ1/ODC1/PSMA3/PSMA5/PSMA7/PSMB2/PSMB6/PSMC3/PSMD3/SRM	11/908	59/10821	9.12E-03	4.45E-02	3.45E-02
R-HSA-70326	***Glucose metabolism***	Metabolism	Metabolism of carbohydrates	AAAS/ALDOA/ALDOC/ENO1/GAPDH/GOT2/GPI/HK2/MDH2/NDC1/NUP107/NUP153/NUP155/NUP35/NUP50/NUP85/NUP88/PCK2/PFKFB4/PGAM1/PGK1/PGP/SEC13/SEH1L/SLC25A1/SLC25A10/SLC25A11/TPI1	28/908	92/10821	8.50E-10	3.92E-08	3.04E-08
R-HSA-70171	***Glycolysis***	Metabolism	Metabolism of carbohydrates	AAAS/ALDOA/ALDOC/ENO1/GAPDH/GPI/HK2/NDC1/NUP107/NUP153/NUP155/NUP35/NUP50/NUP85/NUP88/PFKFB4/PGAM1/PGK1/PGP/SEC13/SEH1L/TPI1	22/908	72/10821	5.06E-08	1.23E-06	9.53E-07

R-HSA-70263	***Gluconeogenesis***	Metabolism	Metabolism of carbohydrates	ALDOA/ALDOC/ENO1/GAPDH/GOT2/GPI/MDH2/PCK2/PGAM1/PGK1/SLC25A1/SLC25A10/SLC25A11/TPI1	14/908	34/10821	2.19E-07	4.14E-06	3.21E-06
R-HSA-170822	***Regulation of Glucokinase by Glucokinase Regulatory Protein***	Metabolism	Metabolism of carbohydrates	AAAS/NDC1/NUP107/NUP153/NUP155/NUP35/NUP50/NUP85/NUP88/SEC13/SEH1L	11/908	32/10821	3.40E-05	3.44E-04	2.67E-04
R-HSA-71336	***Pentose phosphate pathway***	Metabolism	Metabolism of carbohydrates	PGD/PRPS1/PRPS2/RPIA/TALDO1/TKT	6/908	15/10821	8.87E-04	5.97E-03	4.63E-03
R-HSA-191273	***Cholesterol biosynthesis***	Metabolism	Metabolism of lipids	ACAT2/CYP51A1/DHCR7/EBP/FDFT1/FDPS/HMGCR/HMGCS1/LBR/LSS/MSMO1/MVD/MVK/SQLE	14/908	25/10821	1.44E-09	6.05E-08	4.69E-08
R-HSA-1655829	***Regulation of cholesterol biosynthesis by SREBP (SREBF)***	Metabolism	Metabolism of lipids	ACACA/CARM1/CYP51A1/DHCR7/ELOVL6/FASN/FDFT1/FDPS/HMGCR/HMGCS1/INSIG1/KPNB1/LSS/MVD/MVK/RAN/SCD/SEC23A/SQLE/SREBF1	20/908	55/10821	7.23E-09	2.56E-07	1.99E-07
R-HSA-2426168	***Activation of gene expression by SREBF (SREBP)***	Metabolism	Metabolism of lipids	ACACA/CARM1/CYP51A1/DHCR7/ELOVL6/FASN/FDFT1/FDPS/HMGCR/HMGCS1/LSS/MVD/MVK/SCD/SQLE/SREBF1	16/908	42/10821	1.09E-07	2.29E-06	1.78E-06

R-HSA-8957322	***Metabolism of steroids***	Metabolism	Metabolism of lipids	ACACA/ACAT2/AKR1B1/CARM1/CYP51A1/DHCR7/EBP/ELOVL6/FASN/FDFT1/FDPS/HMGCR/HMGCS1/INSIG1/KPNB1/LBR/LSS/MSMO1/MVD/MVK/RAN/SCD/SEC23A/SLC27A5/SQLE/SREBF1/UBE2I	27/908	151/10821	1.29E-04	1.08E-03	8.36E-04
R-HSA-75105	***Fatty acyl-CoA biosynthesis***	Metabolism	Metabolism of lipids	ACACA/ACLY/ACSL3/ELOVL6/FASN/SCD/SLC25A1/TECR	8/908	37/10821	1.03E-02	4.91E-02	3.81E-02
R-HSA-8956320	***Nucleobase biosynthesis***	Metabolism	Metabolism of nucleotides	ATIC/CAD/DHODH/GART/GMPS/IMPDH1/PAICS/PFAS/PPAT	9/908	15/10821	6.25E-07	9.93E-06	7.70E-06
R-HSA-73817	***Purine ribonucleoside monophosphate biosynthesis***	Metabolism	Metabolism of nucleotides	ATIC/GART/GMPS/IMPDH1/PAICS/PFAS/PPAT	7/908	12/10821	1.56E-05	1.77E-04	1.37E-04
R-HSA-499943	***Interconversion of nucleotide di- and triphosphates***	Metabolism	Metabolism of nucleotides	AK2/CTPS1/DCTPP1/DTYMK/DUT/NME4/RRM1/RRM2	8/908	29/10821	2.08E-03	1.20E-02	9.33E-03
R-HSA-196757	***Metabolism of folate and pterines***	Metabolism	Metabolism of vitamins and cofactors	ALDH1L2/DHFR/MTHFD1/MTHFD1L/SHMT2/SLC19A1	6/908	17/10821	1.89E-03	1.12E-02	8.68E-03
R-HSA-611105	***Respiratory electron transport***	Metabolism	The citric acid (TCA) cycle and respiratory electron transport	ACAD9/COX18/COX5A/COX6A1/COX7B/CYC1/CYCS/ECSIT/NDUFA7/NDUFAB1/NDUFAF2/NDUFAF3/NDUFAF4/NDUFB10/NDUFB6/NDUFS6/NDUFS7/NDUFS8/NDUFV2/TRAP1/UQCRC1/UQCRQ	22/908	100/10821	2.14E-05	2.27E-04	1.76E-04

R-HSA-6799198	***Complex I biogenesis***	Metabolism	The citric acid (TCA) cycle and respiratory electron transport	ACAD9/ECSIT/NDUFA7/NDUFAB1/NDUFAF2/NDUFAF3/NDUFAF4/NDUFB10/NDUFB6/NDUFS6/NDUFS7/NDUFS8/NDUFV2	13/908	55/10821	4.90E-04	3.59E-03	2.78E-03
R-HSA-163200	***Respiratory electron transport, ATP synthesis by chemiosmotic coupling, and heat production by uncoupling proteins***	Metabolism	The citric acid (TCA) cycle and respiratory electron transport	ACAD9/COX18/COX5A/COX6A1/COX7B/CYC1/CYCS/ECSIT/NDUFA7/NDUFAB1/NDUFAF2/NDUFAF3/NDUFAF4/NDUFB10/NDUFB6/NDUFS6/NDUFS7/NDUFS8/NDUFV2/TRAP1/UQCRC1/UQCRQ	22/908	124/10821	5.87E-04	4.16E-03	3.23E-03
R-HSA-71406	***Pyruvate metabolism and Citric Acid (TCA) cycle***	Metabolism	The citric acid (TCA) cycle and respiratory electron transport	CS/DLAT/FH/LDHA/LDHB/MDH2/ME2/PDHA1/PDHB/SLC16A1/SUCLG2/VDAC1	12/908	55/10821	1.69E-03	1.02E-02	7.94E-03
R-HSA-15869	***Metabolism of nucleotides***	Metabolism		ADK/AK2/APRT/ATIC/CAD/CTPS1/DCTPP1/DHODH/DNPH1/DTYMK/DUT/GART/GMPS/HPRT1/IMPDH1/NME4/NT5C/NT5C1A/NT5M/PAICS/PFAS/PPAT/RRM1/RRM2/TK1/UCK2	26/908	99/10821	9.91E-08	2.18E-06	1.69E-06

R-HSA-1428517	***The citric acid (TCA) cycle and respiratory electron transport***	Metabolism		ACAD9/COX18/COX5A/COX6A1/COX7B/CS/CYC1/CYCS/DLAT/ECSIT/FH/LDHA/LDHB/MDH2/ME2/NDUFA7/NDUFAB1/NDUFAF2/NDUFAF3/NDUFAF4/NDUFB10/NDUFB6/NDUFS6/NDUFS7/NDUFS8/NDUFV2/PDHA1/PDHB/SLC16A1/SUCLG2/TRAP1/UQCRC1/UQCRQ/VDAC1	34/908	175/10821	2.77E-06	3.94E-05	3.05E-05
R-HSA-71387	***Metabolism of carbohydrates***	Metabolism		AAAS/AKR1B1/ALDOA/ALDOC/B3GNT2/B4GALT2/CALM1/DCXR/ENO1/EPM2A/FUT11/GALK1/GAPDH/GOT2/GPI/HK2/HMMR/HS6ST1/KHK/MDH2/NAGLU/NDC1/NUP107/NUP153/NUP155/NUP35/NUP50/NUP85/NUP88/PCK2/PFKFB4/PGAM1/PGD/PGK1/PGM1/PGP/PRPS1/PRPS2/PYGL/RPIA/SEC13/SEH1L/SLC25A1/SLC25A10/SLC25A11/TALDO1/TKT/TPI1	48/908	293/10821	4.74E-06	6.15E-05	4.77E-05
R-HSA-4615885	***SUMOylation of DNA replication proteins***	Metabolism of proteins	Post-translational protein modification	AAAS/AURKA/AURKB/BIRC5/CDCA8/INCENP/NDC1/NUP107/NUP153/NUP155/NUP35/NUP50/NUP85/NUP88/RANGAP1/SEC13/SEH1L/UBE2I	18/908	46/10821	1.06E-08	3.46E-07	2.68E-07
R-HSA-4570464	***SUMOylation of RNA binding proteins***	Metabolism of proteins	Post-translational protein modification	AAAS/HNRNPC/NDC1/NOP58/NUP107/NUP153/NUP155/NUP35/NUP50/NUP85/NUP88/SCMH1/SEC13/SEH1L/UBE2I	15/908	47/10821	3.73E-06	5.05E-05	3.92E-05

R-HSA-2990846	***SUMOylation***	Metabolism of proteins	Post-translational protein modification	AAAS/AURKA/AURKB/BIRC5/CDCA8/CHD3/CTBP1/DNMT1/HDAC7/HNRNPC/INCENP/NDC1/NOP58/NPM1/NUP107/NUP153/NUP155/NUP35/NUP50/NUP85/NUP88/PARP1/RAD21/RANGAP1/SAE1/SAFB/SCMH1/SEC13/SEH1L/SMC1A/SMC3/STAG1/TRIM28/UBA2/UBE2I	35/908	187/10821	4.81E-06	6.16E-05	4.77E-05
R-HSA-3108214	***SUMOylation of DNA damage response and repair proteins***	Metabolism of proteins	Post-translational protein modification	AAAS/HDAC7/NDC1/NUP107/NUP153/NUP155/NUP35/NUP50/NUP85/NUP88/PARP1/RAD21/SCMH1/SEC13/SEH1L/SMC1A/SMC3/STAG1/UBE2I	19/908	77/10821	1.39E-05	1.64E-04	1.27E-04
R-HSA-4085377	***SUMOylation of SUMOylation proteins***	Metabolism of proteins	Post-translational protein modification	AAAS/NDC1/NUP107/NUP153/NUP155/NUP35/NUP50/NUP85/NUP88/SEC13/SEH1L/UBE2I	12/908	35/10821	1.54E-05	1.77E-04	1.37E-04
R-HSA-3232142	***SUMOylation of ubiquitinylation proteins***	Metabolism of proteins	Post-translational protein modification	AAAS/NDC1/NUP107/NUP153/NUP155/NUP35/NUP50/NUP85/NUP88/SEC13/SEH1L/UBE2I	12/908	39/10821	5.26E-05	5.16E-04	4.00E-04
R-HSA-4551638	***SUMOylation of chromatin organization proteins***	Metabolism of proteins	Post-translational protein modification	AAAS/CHD3/NDC1/NUP107/NUP153/NUP155/NUP35/NUP50/NUP85/NUP88/SCMH1/SEC13/SEH1L/UBE2I	14/908	71/10821	2.03E-03	1.18E-02	9.17E-03
R-HSA-8866652	***Synthesis of active ubiquitin: roles of E1 and E2 enzymes***	Metabolism of proteins	Post-translational protein modification	UBE2C/UBE2D2/UBE2E3/UBE2S/UBE2T/USP5/USP7	7/908	30/10821	1.05E-02	4.99E-02	3.87E-02
R-HSA-390450	***Folding of actin by CCT/TriC***	Metabolism of proteins	Protein folding	ACTB/CCT2/CCT4/CCT5/CCT7/CCT8/TCP1	7/908	10/10821	2.74E-06	3.94E-05	3.05E-05

R-HSA-389958	***Cooperation of Prefoldin and TriC/CCT in actin and tubulin folding***	Metabolism of proteins	Protein folding	ACTB/CCT2/CCT4/CCT5/CCT7/CCT8/PFDN2/PFDN6/TCP1/TUBA1B/TUBA1C/TUBB4B	12/908	33/10821	7.66E-06	9.54E-05	7.40E-05
R-HSA-389960	***Formation of tubulin folding intermediates by CCT/TriC***	Metabolism of proteins	Protein folding	CCT2/CCT4/CCT5/CCT7/CCT8/TCP1/TUBA1B/TUBA1C/TUBB4B	9/908	26/10821	1.67E-04	1.36E-03	1.06E-03
R-HSA-391251	***Protein folding***	Metabolism of proteins	Protein folding	ACTB/ARL2/CCNE1/CCNE2/CCT2/CCT4/CCT5/CCT7/CCT8/GNAZ/NOP56/PFDN2/PFDN6/TBCB/TBCD/TCP1/TUBA1B/TUBA1C/TUBB4B/WRAP53	20/908	102/10821	2.67E-04	2.11E-03	1.63E-03
R-HSA-390471	***Association of TriC/CCT with target proteins during biosynthesis***	Metabolism of proteins	Protein folding	CCNE1/CCNE2/CCT2/CCT4/CCT5/CCT7/CCT8/NOP56/TCP1/WRAP53	10/908	39/10821	1.10E-03	7.21E-03	5.59E-03
R-HSA-389977	***Post-chaperonin tubulin folding pathway***	Metabolism of proteins	Protein folding	ARL2/TBCB/TBCD/TUBA1B/TUBA1C/TUBB4B	6/908	23/10821	1.00E-02	4.86E-02	3.76E-02
R-HSA-5368287	***Mitochondrial translation***	Metabolism of proteins	Translation	AURKAIP1/CHCHD1/GADD45GIP1/MRPL11/MRPL12/MRPL14/MRPL16/MRPL17/MRPL2/MRPL20/MRPL22/MRPL23/MRPL24/MRPL3/MRPL34/MRPL36/MRPL37/MRPL38/MRPL4/MRPL48/MRPL54/MRPL55/MRPL9/MRPS12/MRPS15/MRPS17/MRPS2/MRPS26/MRPS28/MRPS34/TSFM/TUFM	32/908	96/10821	3.43E-12	2.26E-10	1.75E-10

R-HSA-379726	***Mitochondrial tRNA aminoacylation***	Metabolism of proteins	Translation	DARS2/FARS2/MARS2/NARS2/PPA2/SARS2/YARS2	7/908	21/10821	1.17E-03	7.57E-03	5.87E-03
R-HSA-379724	***tRNA Aminoacylation***	Metabolism of proteins	Translation	DARS2/FARS2/FARSA/FARSB/MARS2/NARS2/PPA2/SARS2/YARS2	9/908	42/10821	7.06E-03	3.53E-02	2.73E-02
R-HSA-381042	***PERK regulates gene expression***	Metabolism of proteins	Unfolded Protein Response (UPR)	ASNS/ATF4/CEBPG/EIF2S2/EXOSC2/EXOSC4/EXOSC5/EXOSC8/EXOSC9/HSPA5/KHSRP	11/908	32/10821	3.40E-05	3.44E-04	2.67E-04
R-HSA-380994	***ATF4 activates genes in response to endoplasmic reticulum stress***	Metabolism of proteins	Unfolded Protein Response (UPR)	ASNS/ATF4/CEBPG/EXOSC2/EXOSC4/EXOSC5/EXOSC8/EXOSC9/KHSRP	9/908	27/10821	2.32E-04	1.86E-03	1.44E-03
R-HSA-72766	***Translation***	Metabolism of proteins		APEH/AURKAIP1/CHCHD1/DARS2/EIF1AX/EIF2S2/EIF4E/EIF4EBP1/EIF4G1/FARS2/FARSA/FARSB/GADD45GIP1/GSPT1/MARS2/MRPL11/MRPL12/MRPL14/MRPL16/MRPL17/MRPL2/MRPL20/MRPL22/MRPL23/MRPL24/MRPL3/MRPL34/MRPL36/MRPL37/MRPL38/MRPL4/MRPL48/MRPL54/MRPL55/MRPL9/MRPS12/MRPS15/MRPS17/MRPS2/MRPS26/MRPS28/MRPS34/NARS2/PPA2/RPL17/RPL35/RPN1/RPS12/RPS26/SARS2/SPCS2/SRP9/SRPRB/TSFM/TUFM/YARS2	56/908	295/10821	4.13E-09	1.52E-07	1.18E-07

R-HSA-381119	***Unfolded Protein Response (UPR)***	Metabolism of proteins		ASNS/ATF4/CEBPG/CXXC1/DDX11/EIF2S2/EXOSC2/EXOSC4/EXOSC5/EXOSC8/EXOSC9/HDGF/HSPA5/KHSRP/PDIA6/SRPRB/XBP1/YIF1A	18/908	92/10821	5.48E-04	3.98E-03	3.08E-03
R-HSA-429958	***mRNA decay by 3' to 5' exoribonuclease***	Metabolism of RNA	Deadenylation-dependent mRNA decay	DCPS/EXOSC2/EXOSC4/EXOSC5/EXOSC8/EXOSC9	6/908	16/10821	1.32E-03	8.29E-03	6.43E-03
R-HSA-191859	***snRNP Assembly***	Metabolism of RNA	Metabolism of non-coding RNA	AAAS/GEMIN2/GEMIN4/NDC1/NUP107/NUP153/NUP155/NUP35/NUP50/NUP85/NUP88/PRMT5/SEC13/SEH1L/SNRPB/SNRPD1/SNRPD2/SNRPE/SNRPF/SNRPG/SNUPN	21/908	54/10821	7.17E-10	3.48E-08	2.70E-08
R-HSA-72172	***mRNA Splicing***	Metabolism of RNA	Processing of Capped Intron-Containing Pre-mRNA	ALYREF/CHERP/DHX15/DHX9/EIF4A3/ELAVL1/FUS/HNRNPA0/HNRNPA2B1/HNRNPA3/HNRNPC/HNRNPD/HNRNPF/HNRNPL/HNRNPM/HNRNPR/HNRNPU/HNRNPUL1/LSM4/LSM7/MAGOHB/PCBP1/PCBP2/PHF5A/POLR2E/POLR2F/POLR2H/POLR2I/PPIH/PRPF19/PTBP1/PUF60/RBMX/RNPS1/SF1/SF3A2/SF3B2/SF3B5/SNRNP35/SNRNP40/SNRNP70/SNRPA/SNRPA1/SNRPB/SNRPC/SNRPD1/SNRPD2/SNRPE/SNRPF/SNRPG/SNU13/SRRM1/SRRT/SRSF1/SRSF2/SRSF3/SRSF4/SRSF7/SRSF9/SYMPK/TRA2B/TXNL4A/U2AF2/YBX1	64/908	188/10821	1.04E-23	4.81E-21	3.73E-21

R-HSA-72202	***Transport of Mature Transcript to Cytoplasm***	Metabolism of RNA	Processing of Capped Intron-Containing Pre-mRNA	AAAS/ALYREF/CHTOP/DDX39A/EIF4A3/EIF4E/MAGOHB/NDC1/NUP107/NUP153/NUP155/NUP35/NUP50/NUP85/NUP88/NXT1/RNPS1/SARNP/SEC13/SEH1L/SLBP/SRRM1/SRSF1/SRSF2/SRSF3/SRSF4/SRSF7/SRSF9/SYMPK/THOC6/U2AF2	31/908	84/10821	3.30E-13	2.54E-11	1.97E-11
R-HSA-72165	***mRNA Splicing - Minor Pathway***	Metabolism of RNA	Processing of Capped Intron-Containing Pre-mRNA	POLR2E/POLR2F/POLR2H/POLR2I/SF3B2/SF3B5/SNRNP35/SNRNP40/SNRPB/SNRPD1/SNRPD2/SNRPE/SNRPF/SNRPG/SNU13/SRSF1/SRSF2/SRSF7/TXNL4A/YBX1	20/908	52/10821	2.30E-09	9.22E-08	7.15E-08
R-HSA-159230	***Transport of the SLBP Dependant Mature mRNA***	Metabolism of RNA	Processing of Capped Intron-Containing Pre-mRNA	AAAS/ALYREF/EIF4E/NDC1/NUP107/NUP153/NUP155/NUP35/NUP50/NUP85/NUP88/SEC13/SEH1L/SLBP	14/908	36/10821	5.10E-07	8.71E-06	6.75E-06
R-HSA-159234	***Transport of Mature mRNAs Derived from Intronless Transcripts***	Metabolism of RNA	Processing of Capped Intron-Containing Pre-mRNA	AAAS/ALYREF/EIF4E/NDC1/NUP107/NUP153/NUP155/NUP35/NUP50/NUP85/NUP88/SEC13/SEH1L/SLBP/SYMPK	15/908	43/10821	1.03E-06	1.62E-05	1.25E-05
R-HSA-77588	***SLBP Dependent Processing of Replication-Dependent Histone Pre-mRNAs***	Metabolism of RNA	Processing of Capped Intronless Pre-mRNA	SLBP/SNRPB/SNRPE/SNRPF/SNRPG	5/908	11/10821	1.24E-03	7.92E-03	6.14E-03

R-HSA-450604	***KSRP (KHSRP) binds and destabilizes mRNA***	Metabolism of RNA	Regulation of mRNA stability by proteins that bind AU-rich elements	AKT1/EXOSC2/EXOSC4/EXOSC5/EXOSC8/EXOSC9/KHSRP	7/908	17/10821	2.63E-04	2.09E-03	1.62E-03
R-HSA-450385	***Butyrate Response Factor 1 (BRF1) binds and destabilizes mRNA***	Metabolism of RNA	Regulation of mRNA stability by proteins that bind AU-rich elements	AKT1/EXOSC2/EXOSC4/EXOSC5/EXOSC8/EXOSC9	6/908	17/10821	1.89E-03	1.12E-02	8.68E-03
R-HSA-6790901	***rRNA modification in the nucleus and cytosol***	Metabolism of RNA	rRNA processing	DKC1/EMG1/FBL/GAR1/IMP4/NHP2/NOC4L/NOP56/NOP58/RRP7A/RRP9/SNU13/TSR3/UTP18/UTP20	15/908	60/10821	9.36E-05	8.38E-04	6.50E-04
R-HSA-6784531	***tRNA processing in the nucleus***	Metabolism of RNA	tRNA processing	AAAS/ELAC2/NDC1/NUP107/NUP153/NUP155/NUP35/NUP50/NUP85/NUP88/POP7/RAN/RPP25/SEC13/SEH1L/TSEN15/XPOT	17/908	59/10821	4.13E-06	5.44E-05	4.21E-05

R-HSA-72203	***Processing of Capped Intron-Containing Pre-mRNA***	Metabolism of RNA	AAAS/ALYREF/CHERP/CHTOP/DDX39A/DHX15/DHX9/EIF4A3/EIF4E/ELAVL1/FUS/HNRNPA0/HNRNPA2B1/HNRNPA3/HNRNPC/HNRNPD/HNRNPF/HNRNPL/HNRNPM/HNRNPR/HNRNPU/HNRNPUL1/LSM4/LSM7/MAGOHB/NDC1/NUP107/NUP153/NUP155/NUP35/NUP50/NUP85/NUP88/NXT1/PCBP1/PCBP2/PHF5A/POLR2E/POLR2F/POLR2H/POLR2I/PPIH/PRPF19/PTBP1/PUF60/RBMX/RNPS1/SARNP/SEC13/SEH1L/SF1/SF3A2/SF3B2/SF3B5/SLBP/SNRNP35/SNRNP40/SNRNP70/SNRPA/SNRPA1/SNRPB/SNRPC/SNRPD1/SNRPD2/SNRPE/SNRPF/SNRPG/SNU13/SRRM1/SRRT/SRSF1/SRSF2/SRSF3/SRSF4/SRSF7/SRSF9/SYMPK/THOC6/TRA2B/TXNL4A/U2AF2/YBX1	82/908	242/10821	5.57E-30	5.13E-27	3.98E-27
R-HSA-72312	***rRNA processing***	Metabolism of RNA	BOP1/BYSL/DDX21/DKC1/EBNA1BP2/ELAC2/EMG1/EXOSC2/EXOSC4/EXOSC5/EXOSC8/EXOSC9/FBL/GAR1/GNL3/IMP4/ISG20L2/LAS1L/MRM1/NCL/NHP2/NOC4L/NOL12/NOP56/NOP58/PELP1/PES1/RPL17/RPL35/RPP25/RPS12/RPS26/RRP7A/RRP9/SNU13/TSR3/UTP18/UTP20/WDR12/WDR18	40/908	205/10821	3.31E-07	5.86E-06	4.55E-06

R-HSA-72306	***tRNA processing***	Metabolism of RNA		AAAS/CTU2/ELAC2/FTSJ1/GTPBP3/NDC1/NUP107/NUP153/NUP155/NUP35/NUP50/NUP85/NUP88/POP7/PUS1/PUS7/RAN/RPP25/SEC13/SEH1L/TRMT61A/TSEN15/WDR4/XPOT	24/908	111/10821	1.25E-05	1.50E-04	1.16E-04
R-HSA-450531	***Regulation of mRNA stability by proteins that bind AU-rich elements***	Metabolism of RNA		AKT1/ANP32A/EIF4G1/ELAVL1/EXOSC2/EXOSC4/EXOSC5/EXOSC8/EXOSC9/HNRNPD/KHSRP/PSMA3/PSMA5/PSMA7/PSMB2/PSMB6/PSMC3/PSMD3/SET	19/908	87/10821	8.54E-05	7.79E-04	6.04E-04
R-HSA-429914	***Deadenylation-dependent mRNA decay***	Metabolism of RNA		DCPS/EIF4A3/EIF4E/EIF4G1/EXOSC2/EXOSC4/EXOSC5/EXOSC8/EXOSC9/LSM4/LSM7	11/908	56/10821	6.11E-03	3.13E-02	2.42E-02
R-HSA-1268020	***Mitochondrial protein import***	Protein localization		BCS1L/CHCHD10/CHCHD2/CHCHD4/CMC2/COQ2/COX17/CS/CYC1/FXN/GRPEL1/GRPEL2/HSPD1/TIMM13/TIMM17B/TIMM44/TIMM50/TOMM40/TOMM5/TOMM6/VDAC1	21/908	65/10821	3.42E-08	8.63E-07	6.69E-07
R-HSA-9609507	***Protein localization***	Protein localization		BCS1L/CHCHD10/CHCHD2/CHCHD4/CMC2/COQ2/COX17/CS/CYC1/FXN/GRPEL1/GRPEL2/HSPD1/IDH1/TIMM13/TIMM17B/TIMM44/TIMM50/TOMM40/TOMM5/TOMM6/TYSND1/UBE2D2/UBL4A/VDAC1	25/908	164/10821	2.47E-03	1.41E-02	1.09E-02
R-HSA-6803529	***FGFR2 alternative splicing***	Signal Transduction	Signaling by Receptor Tyrosine Kinases	HNRNPF/HNRNPM/POLR2E/POLR2F/POLR2H/POLR2I/PTBP1/TIAL1	8/908	27/10821	1.25E-03	7.94E-03	6.15E-03

R-HSA-5663220	***RHO GTPases Activate Formins***	Signal Transduction	Signaling by Rho GTPases, Miro GTPases and RHOBTB3	ACTB/ACTG1/AURKB/BIRC5/BUB1/BUB1B/BUB3/CDC20/CDCA8/CENPA/CENPE/CENPF/CENPM/DIAPH3/DVL1/ERCC6L/INCENP/KNTC1/MAD2L1/NDC80/NUDC/NUF2/NUP107/NUP85/PFN1/PLK1/PPP1CC/RAC1/RANGAP1/SEC13/SEH1L/SKA1/SPC25/SPDL1/TUBA1B/TUBA1C/TUBB4B/ZW10/ZWINT	39/908	140/10821	8.90E-12	5.47E-10	4.24E-10
R-HSA-195258	***RHO GTPase Effectors***	Signal Transduction	Signaling by Rho GTPases, Miro GTPases and RHOBTB3	ACTB/ACTG1/AURKB/BIRC5/BUB1/BUB1B/BUB3/CALM1/CDC20/CDCA8/CENPA/CENPE/CENPF/CENPM/CFL1/DIAPH3/DVL1/ERCC6L/INCENP/KDM1A/KIF14/KNTC1/MAD2L1/NCKIPSD/NDC80/NUDC/NUF2/NUP107/NUP85/PFN1/PKN3/PLK1/PPP1CC/PRC1/RAC1/RANGAP1/SEC13/SEH1L/SKA1/SPC25/SPDL1/TUBA1B/TUBA1C/TUBB4B/YWHAE/YWHAH/ZW10/ZWINT	48/908	324/10821	7.02E-05	6.53E-04	5.06E-04

R-HSA-194315	***Signaling by Rho GTPases***	Signal Transduction	Signaling by Rho GTPases, Miro GTPases and RHOBTB3	ACTB/ACTG1/ARHGAP11A/ARHGAP17/ARHGAP19/ARHGDIA/ARHGEF39/AURKB/BIRC5/BUB1/BUB1B/BUB3/CALM1/CDC20/CDCA8/CENPA/CENPE/CENPF/CENPM/CFL1/DEPDC1B/DIAPH3/DVL1/ECT2/ERCC6L/INCENP/KDM1A/KIF14/KNTC1/MAD2L1/NCKIPSD/NDC80/NUDC/NUF2/NUP107/NUP85/PFN1/PKN3/PLK1/PPP1CC/PRC1/RAC1/RACGAP1/RANGAP1/SEC13/SEH1L/SKA1/SPC25/SPDL1/TUBA1B/TUBA1C/TUBB4B/YWHAE/YWHAH/ZW10/ZWINT	56/908	454/10821	2.10E-03	1.21E-02	9.38E-03
R-HSA-6811434	***COPI-dependent Golgi-to-ER retrograde traffic***	Vesicle-mediated transport	Membrane Trafficking	ARF1/CENPE/KDELR1/KDELR2/KIF11/KIF15/KIF18B/KIF20A/KIF22/KIF23/KIF4A/KIFC1/RAB1B/RACGAP1/SURF4/TUBA1B/TUBA1C/TUBB4B/ZW10	19/908	100/10821	5.70E-04	4.08E-03	3.16E-03
R-HSA-8856688	***Golgi-to-ER retrograde transport***	Vesicle-mediated transport	Membrane Trafficking	ARF1/CENPE/KDELR1/KDELR2/KIF11/KIF15/KIF18B/KIF20A/KIF22/KIF23/KIF4A/KIFC1/PAFAH1B3/PLA2G4A/RAB1B/RACGAP1/SURF4/TUBA1B/TUBA1C/TUBB4B/ZW10	21/908	134/10821	3.79E-03	2.03E-02	1.58E-02
