# Supplementary material for: A novel integrative multi-omics approach to unravel the genetic determinants of rare diseases with application in sinusoidal obstruction syndrome
Source: PLoS One. 2023 Apr 5;18(4):e0281892. doi: 10.1371/journal.pone.0281892 (PMC10075428; doi:10.1371/journal.pone.0281892)
Supplement: S4 Table — The SNPs are sorted by chromosome and position. Functional annotations of the variants were retrieved from ENSEMBL (Genome assembly: GRCh37.p13). The r2 column shows the r2 measure of linkage disequilibrium between each SNP and the following SNP in the table, in the CEU population from the 1,000 Genomes phase 3 data, as reported in ENSEMBL. (PDF) [file pone.0281892.s004.pdf]

Supplementary Table S03. List of SNPs that are found to be in significant association with VOD, after correction for multiple testing. The SNPs are sorted by chromosome and position. Functional annotations of the variants were retrieved from ENSEMBL (Genome assembly: GRCh37.p13). The r2 column shows the r2 measure of linkage disequilibrium between each SNP and the following SNP in the table, in the CEU population from the 1,000 Genomes phase 3 data, as reported in ENSEMBL.

Chromosome	Position	Reference	Alternative	Ancestral	r2	*Gene name*	SNP	SNP location	CADD	unadjusted p-value	adjusted p-value (Bonferroni)	adjusted p-value (FDR_BH)
1	1639073	A	T		<0.05	***CDK11A***	rs376287692	intronic	5.566	8.16E-07	2.04E-01	**1.70E-02**
1	38155699	T	G	T	0.890	***C1orf109***	rs4653319	UTR5	6.734	7.58E-06	1.00E+00	**3.88E-02**
1	38158211	C	T	C	1.000	***CDCA8***	rs2306625	UTR5	7.554	7.58E-06	1.00E+00	**3.88E-02**
1	38174366	G	T	G	1.000	***CDCA8***	rs7526362	UTR3	3.178	7.58E-06	1.00E+00	**3.88E-02**
1	38182135	C	T	C	<0.05	***EPHA10***	rs900676	UTR3	6.142	2.33E-06	5.83E-01	**2.40E-02**
1	47602879	C	A	A	<0.05	***CYP4A22***	rs11581909	intronic	0.745	1.13E-05	1.00E+00	**4.79E-02**
1	49711281	C	T	C	<0.05	***AGBL4***	rs7528510	intronic	1.219	1.01E-05	1.00E+00	**4.38E-02**
1	52373863	G	A	G	1.000	***RAB3B***	rs1045633	UTR3	3.490	1.26E-06	3.15E-01	**1.75E-02**
1	52373894	A	C	A	0.865	***RAB3B***	rs12730	UTR3	9.962	1.26E-06	3.15E-01	**1.75E-02**
1	52374413	A	T	T	0.865	***RAB3B***	rs34922023	UTR3	2.542	1.26E-06	3.15E-01	**1.75E-02**
1	52374576	T	A	T	1.000	***RAB3B***	rs6701110	UTR3	8.930	1.26E-06	3.15E-01	**1.75E-02**
1	52376045	G	A	G	<0.05	***RAB3B***	rs34173577	UTR3	3.805	1.26E-06	3.15E-01	**1.75E-02**
1	67702141	C	T	C	<0.05	***IL23R***	rs11465803	intronic	6.770	3.71E-06	9.29E-01	**2.73E-02**
1	196748676	A	G		1.000	***CFHR3***	rs620015	intronic	6.047	7.49E-09	**1.88E-03**	**1.88E-03**
1	196763126	G	C		<0.05	***CFHR3***	rs390837	UTR3	0.285	1.15E-05	1.00E+00	**4.79E-02**
1	203017087	C	T	C		***PPFIA4***	rs754894	intronic	2.446	9.60E-06	1.00E+00	**4.25E-02**
2	107418036	T	C	T	0.980	***RGPD3,ST6GAL2***	rs1345198	intergenic	2.047	8.28E-06	1.00E+00	**3.88E-02**
2	107420143	C	T	T	1.000	***ST6GAL2***	rs10496407	UTR3	0.767	8.28E-06	1.00E+00	**3.88E-02**
2	107421166	G	A	G	0.980	***ST6GAL2***	rs6543444	UTR3	0.201	2.97E-06	7.45E-01	**2.40E-02**
2	107423188	G	A	G	<0.05	***ST6GAL2***	rs1470591	exonic (synonymous)	0.002	8.28E-06	1.00E+00	**3.88E-02**
2	127657770	G	T	G	<0.05	***AC114783.1***	rs13401458	intronic	4.913	9.66E-06	1.00E+00	**4.25E-02**
2	189918337	T	C	C		***COL5A2***	rs6750027	intronic	0.193	4.31E-06	1.00E+00	**2.84E-02**

3	168867725	G	C	C		***MECOM***	rs1975981	intronic	1.425	2.65E-06	6.64E-01	**2.40E-02**
4	74702119	G	T	G	1.000	***CXCL6***	rs79143912	upstream	5.293	4.31E-06	1.00E+00	**2.84E-02**
4	74702625	G	C	G	<0.05	***CXCL6***	rs41401748	intronic	7.945	4.31E-06	1.00E+00	**2.84E-02**
4	74719681	T	A	T		***PF4V1***	rs2233653	intronic	0.085	5.26E-06	1.00E+00	**3.13E-02**
4	74719729	G	A	A		***PF4V1***	rs2233654	intronic	0.355	5.26E-06	1.00E+00	**3.13E-02**
4	108925994	A	G	A	1.000	***HADH***	rs17510991	exonic (non-synonymous)	1.325	3.62E-06	9.07E-01	**2.73E-02**
4	108944732	G	A	G	0.884	***HADH***	rs17511214	intronic	9.906	3.62E-06	9.07E-01	**2.73E-02**
4	108956331	A	G	A		***HADH***	rs17511319	UTR3	0.242	4.00E-07	1.00E-01	**1.25E-02**
5	34845473	C	A	A		***TTC23L***	rs336486	intronic	0.430	6.33E-06	1.00E+00	**3.48E-02**
6	32632528	G	T	G		***HLA-DQB1***	rs28746793	intronic	10.020	1.37E-06	3.42E-01	**1.80E-02**
6	41196890	T	C	T		***TREML4***	rs2009934	intronic	4.301	7.96E-06	1.00E+00	**3.88E-02**
6	137144027	T	C	T	0.650	***PEX7***	rs13220063	intronic	9.145	6.53E-07	1.64E-01	**1.49E-02**
6	137166650	A	G	A	1.000	***PEX7***	rs2295592	intronic	3.389	2.31E-06	5.80E-01	**2.40E-02**
6	137191970	G	A	G	0.091	***PEX7***	rs34118640	UTR3	1.034	9.78E-08	**2.45E-02**	**3.50E-03**
6	151743948	A	G	A		***RMND1***	rs9478203	intronic	2.271	6.39E-06	1.00E+00	**3.48E-02**
7	38393857	C	T	C		***TRGV4***	rs2392548	UTR5	1.553	5.96E-07	1.49E-01	**1.49E-02**
7	38393858	C	T	C	0.087	***TRGV4***	rs2392549	UTR5	5.679	5.96E-07	1.49E-01	**1.49E-02**
7	73513106	G	A	G	o	***LIMK1***	rs58406437	intronic	8.131	5.98E-06	1.00E+00	**3.41E-02**
8	65537168	T	C	T	<0.05	***CYP7B1***	rs73237769	intronic	4.155	1.26E-06	3.15E-01	**1.75E-02**
8	126034052	C	T	C	1.000	***SQLE***	rs2293985	exonic (synonymous)	8.569	2.92E-06	7.32E-01	**2.40E-02**
8	126067985	A	T	T	1.000	***KIAA0196***	rs2303529	intronic	3.885	2.92E-06	7.32E-01	**2.40E-02**
8	126069142	T	C	T	1.000	***KIAA0196***	rs2303521	intronic	6.503	2.92E-06	7.32E-01	**2.40E-02**
8	126071380	A	G	A	1.000	***KIAA0196***	rs3765214	intronic	4.113	2.92E-06	7.32E-01	**2.40E-02**
8	126071452	T	C	T		***KIAA0196***	rs3765213	intronic	1.664	2.92E-06	7.32E-01	**2.40E-02**
9	114412261	G	A	G		***DNAJC25,DNAJC25-GNG10***	rs10980991	intronic	3.208	6.90E-08	**1.73E-02**	**2.88E-03**
10	61115482	G	A	G		***FAM13C***	rs12358821	intronic	0.321	4.70E-06	1.00E+00	**3.02E-02**
11	92625944	G	T	G		***FAT3***	rs11823754	UTR3	1.283	3.24E-08	**8.13E-03**	**2.03E-03**
11	111384426	T	G	T		***MIR34B,MIR34C***	rs28690953	intergenic	1.207	5.18E-08	**1.30E-02**	**2.59E-03**
14	69329029	T	C	C	0.478	***ZFP36L1,ACTN1***	rs441983	intergenic	2.770	9.37E-06	1.00E+00	**4.25E-02**

14	69329149	C	T	T		***ZFP36L1,ACTN1***	rs1742501	intergenic	13.010	5.26E-06	1.00E+00	**3.13E-02**
15	81480893	T	C	T	1.000	***IL16***	rs74639442	intronic	0.178	3.03E-08	**7.58E-03**	**2.03E-03**
15	81480896	G	A	G		***IL16***	rs78380152	intronic	4.295	2.06E-08	**5.15E-03**	**2.03E-03**
16	85145802	G	A	G		***FAM92B***	rs74031949	intronic	4.679	2.92E-06	7.32E-01	**2.40E-02**
16	88802494	G	A	G		***PIEZO1***	rs2879906	intronic	0.458	8.37E-06	1.00E+00	**3.88E-02**
18	54606452	C	T	T	1.000	***WDR7***	rs28595099	intronic	1.292	2.92E-06	7.32E-01	**2.40E-02**
18	54606734	T	C	T		***WDR7***	rs8093847	intronic	4.459	2.92E-06	7.32E-01	**2.40E-02**
20	23805953	C	T	G		***CST2***	rs45539436	exonic (non-synonymous)	12.200	4.31E-06	1.00E+00	**2.84E-02**
22	45182076	G	A	G		***ARHGAP8,PRR5-ARHGAP8***	rs731548	intronic	0.359	5.38E-06	1.00E+00	**3.13E-02**
