# Supplementary material for: A novel integrative multi-omics approach to unravel the genetic determinants of rare diseases with application in sinusoidal obstruction syndrome
Source: PLoS One. 2023 Apr 5;18(4):e0281892. doi: 10.1371/journal.pone.0281892 (PMC10075428; doi:10.1371/journal.pone.0281892)
Supplement: S5 Table — (PDF) [file pone.0281892.s005.pdf]

Supplementary Table S04. Whole-exome sequencing association analysis of 57 individuals (11 cases with sinusoidal obstruction syndrome and 46 controls), genes with the strongest association, sorted by adjusted association metric.

Chr	Gene	Full name	nSNPs	Start	Stop	P-value adj.bonf	P-value adj.BH
**8**	***NACAP1***	*NACA family member 4, pseudogene*	99	1993157	2093380	**2.382E-02**	**7.942E-03**
**16**	***E2F4***	*E2F transcription factor 4*	11	80574853	80584541	**2.382E-02**	**7.942E-03**
**16**	***SHCBP1***	*SHC binding and spindle associated 1*	16	70557690	70611571	**2.382E-02**	**7.942E-03**
**11**	***MIR34C***	*microRNA 34c*	2	111384163	111384240	**4.765E-02**	**1.191E-02**
**4**	***PF4V1***	*platelet factor 4 variant 1*	3	74719012	74720198	7.147E-02	**1.191E-02**
**8**	***CYP7B1***	*cytochrome P450 family 7 subfamily B member 1*	4	65508528	65711348	7.147E-02	**1.191E-02**
**16**	***TMEM208***	*transmembrane protein 208*	7	8889036	8891505	1.429E-01	**2.042E-02**
**4**	***CXCL6***	*C-X-C motif chemokine ligand 6*	2	74702272	74704477	3.097E-01	**3.872E-02**
**1**	***RAB3B***	*Ras-Related Protein Rab-3B*	24	52373627	52456436	5.003E-01	**4.964E-02**
**7**	***LOC101928744***	-	2	41004276	41019537	5.241E-01	**4.964E-02**
**7**	***LOC101928773***	-	2	41141201	41173099	5.718E-01	**4.964E-02**
**1**	***CFHR3***	*Complement Factor H Related 3*	16	196743929	196763203	5.956E-01	**4.964E-02**
