# Supplementary material for: A novel integrative multi-omics approach to unravel the genetic determinants of rare diseases with application in sinusoidal obstruction syndrome
Source: PLoS One. 2023 Apr 5;18(4):e0281892. doi: 10.1371/journal.pone.0281892 (PMC10075428; doi:10.1371/journal.pone.0281892)
Supplement: S6 Table — (PDF) [file pone.0281892.s006.pdf]

Supplementary Table S05. Combined association analysis of differential gene expression in lymphoblastoid cell lines and whole-exome sequencing in 57 individuals; genes sorted by adjusted association metric.

Gene	WES analysis gene p-value	WES analysis z-score	DEG LCL analysis log2 fold change	DEG LCL analysis p-value unadjusted	DEG LCL analysis p-value rescaled	DEG LCL analysis z-score	Combined statistic average z-score	Combined statistic average z-score p-value	Combined statistic average z-score p-value adjusted
DDR1	1.400E-04	3.633E+00	9.637E-01	8.336E-14	3.000E-06	4.671E+00	5.872E+00	2.156E-09	3.911E-05
HCAR3	1.600E-03	2.948E+00	2.047E+00	3.164E-14	3.000E-06	4.671E+00	5.387E+00	3.578E-08	6.492E-04
ITGAM	2.400E-03	2.820E+00	1.519E+00	1.644E-09	3.002E-06	4.671E+00	5.297E+00	5.891E-08	1.069E-03
GPR137B	4.760E-03	2.593E+00	5.027E-01	3.746E-09	3.004E-06	4.671E+00	5.136E+00	1.403E-07	2.547E-03
PTP4A1	5.380E-03	2.550E+00	4.040E-01	1.561E-07	3.156E-06	4.660E+00	5.099E+00	1.709E-07	3.101E-03
PPL	2.380E-03	2.823E+00	1.598E+00	1.130E-05	1.431E-05	4.339E+00	5.064E+00	2.050E-07	3.720E-03
AIM1	2.170E-04	3.518E+00	2.573E-01	2.754E-04	2.787E-04	3.634E+00	5.058E+00	2.120E-07	3.847E-03
GBP5	5.640E-03	2.534E+00	9.836E-01	1.414E-06	4.415E-06	4.591E+00	5.038E+00	2.353E-07	4.269E-03
ITGB8	1.041E-03	3.078E+00	5.901E-01	5.387E-05	5.693E-05	4.025E+00	5.023E+00	2.545E-07	4.618E-03
PHYHIP	7.400E-03	2.437E+00	2.719E+00	8.301E-08	3.083E-06	4.665E+00	5.022E+00	2.554E-07	4.635E-03
TNFRSF10B	7.780E-03	2.419E+00	7.341E-01	2.239E-19	3.000E-06	4.671E+00	5.013E+00	2.675E-07	4.854E-03
LACC1	8.270E-03	2.397E+00	1.540E+00	4.186E-11	3.000E-06	4.671E+00	4.998E+00	2.903E-07	5.268E-03
UTRN	1.201E-02	2.257E+00	6.229E-01	8.372E-09	3.008E-06	4.670E+00	4.898E+00	4.837E-07	8.776E-03
TRIM55	1.327E-02	2.218E+00	3.358E+00	1.824E-08	3.018E-06	4.670E+00	4.870E+00	5.568E-07	1.010E-02
CLCF1	7.070E-03	2.454E+00	7.189E-01	9.272E-06	1.228E-05	4.373E+00	4.827E+00	6.935E-07	1.258E-02
ANKRA2	1.670E-02	2.127E+00	7.310E-01	9.438E-10	3.001E-06	4.671E+00	4.807E+00	7.664E-07	1.391E-02
HSPG2	1.805E-02	2.096E+00	1.774E+00	1.775E-22	3.000E-06	4.671E+00	4.785E+00	8.561E-07	1.553E-02
POLH	1.851E-02	2.086E+00	6.523E-01	1.431E-15	3.000E-06	4.671E+00	4.777E+00	8.876E-07	1.610E-02
DDX60	1.832E-02	2.090E+00	6.526E-01	4.204E-07	3.421E-06	4.644E+00	4.761E+00	9.616E-07	1.745E-02
AFAP1L2	9.250E-03	2.355E+00	7.235E-01	1.458E-05	1.759E-05	4.293E+00	4.701E+00	1.291E-06	2.343E-02
PGAP1	2.454E-02	1.968E+00	7.789E-01	1.669E-08	3.017E-06	4.670E+00	4.693E+00	1.343E-06	2.437E-02
CALML6	2.540E-02	1.953E+00	2.122E+00	1.693E-08	3.017E-06	4.670E+00	4.683E+00	1.413E-06	2.564E-02
ZBTB7C	2.586E-02	1.945E+00	5.542E+00	6.990E-08	3.070E-06	4.666E+00	4.675E+00	1.469E-06	2.666E-02
GNA15	1.439E-02	2.186E+00	4.459E-01	9.154E-06	1.216E-05	4.375E+00	4.639E+00	1.747E-06	3.170E-02
MAP4K4	3.055E-02	1.873E+00	1.065E+00	1.900E-13	3.000E-06	4.671E+00	4.627E+00	1.855E-06	3.366E-02
LINC01021	3.170E-02	1.856E+00	1.982E+00	8.752E-47	3.000E-06	4.671E+00	4.615E+00	1.961E-06	3.559E-02
IL17RC	1.900E-03	2.894E+00	1.526E+00	2.828E-04	2.861E-04	3.628E+00	4.612E+00	1.997E-06	3.624E-02

MDM2	3.258E-02	1.844E+00	1.359E+00	1.554E-69	3.000E-06	4.671E+00	4.607E+00	2.045E-06	3.710E-02
CCND1	2.205E-02	2.013E+00	8.169E-01	4.578E-06	7.583E-06	4.477E+00	4.589E+00	2.227E-06	4.041E-02
TMEM168	3.554E-02	1.805E+00	6.338E-01	1.276E-09	3.001E-06	4.671E+00	4.579E+00	2.336E-06	4.238E-02
GAS7	2.594E-02	1.944E+00	3.808E-01	3.169E-06	6.173E-06	4.520E+00	4.571E+00	2.426E-06	4.402E-02
TRPV3	3.591E-02	1.800E+00	6.300E-01	1.690E-07	3.169E-06	4.660E+00	4.568E+00	2.465E-06	4.472E-02
FAS	3.757E-02	1.780E+00	6.822E-01	4.807E-14	3.000E-06	4.671E+00	4.561E+00	2.544E-06	4.616E-02
SEMA6A	3.824E-02	1.771E+00	7.528E-01	3.736E-09	3.004E-06	4.671E+00	4.555E+00	2.617E-06	4.748E-02
PADI4	3.848E-02	1.769E+00	3.059E+00	1.547E-25	3.000E-06	4.671E+00	4.553E+00	2.640E-06	4.790E-02
