# Supplementary material for: A novel integrative multi-omics approach to unravel the genetic determinants of rare diseases with application in sinusoidal obstruction syndrome
Source: PLoS One. 2023 Apr 5;18(4):e0281892. doi: 10.1371/journal.pone.0281892 (PMC10075428; doi:10.1371/journal.pone.0281892)
Supplement: S7 Table — (PDF) [file pone.0281892.s007.pdf]

Supplementary Table S06. Over-representation analysis of differentially expressed genes in a combined test statistic integrating differential expression data of lymphoblastoid cell lines after exposure to busulfan and a whole-exome association analysis.

KEGG ID	Description	Category	geneID	size	p-value	q-value
hsa04115	p53 signaling pathway	Cell growth and death	CCND1/FAS/MDM2/TNFRSF10B	72	1.00E-05	8.74E-04
hsa04218	Cellular senescence	Cell growth and death	CCND1/MDM2/CALML6	160	3.45E-03	1.37E-02
hsa04060	Cytokine-cytokine receptor interaction	Signaling molecules and interaction	FAS/CLCF1/IL17RC/TNFRSF10B	294	2.23E-03	1.14E-02
hsa05205	Proteoglycans in cancer	Cancer: overview	CCND1/FAS/MDM2/HSPG2	201	5.45E-04	3.95E-03
hsa05214	Glioma	Cancer: specific types	CCND1/MDM2/CALML6	71	3.30E-04	3.32E-03
hsa05219	Bladder cancer	Cancer: specific types	CCND1/MDM2	41	2.78E-03	1.27E-02
hsa05221	Acute myeloid leukemia	Cancer: specific types	CCND1/ITGAM	66	7.07E-03	2.28E-02
hsa05218	Melanoma	Cancer: specific types	CCND1/MDM2	72	8.37E-03	2.60E-02
hsa05220	Chronic myeloid leukemia	Cancer: specific types	CCND1/MDM2	76	9.29E-03	2.69E-02
hsa05133	Pertussis	Infectious disease: bacterial	ITGAM/CALML6	76	9.29E-03	2.69E-02
hsa05163	Human cytomegalovirus infection	Infectious disease: viral	CCND1/FAS/MDM2/CALML6	225	8.32E-04	5.57E-03
hsa05162	Measles	Infectious disease: viral	CCND1/TNFRSF10B/FAS	132	1.96E-03	1.14E-02
hsa05161	Hepatitis B	Infectious disease: viral	CCND1/FAS/HSPG2	144	2.56E-03	1.24E-02
hsa05165	Human papillomavirus infection	Infectious disease: viral	CCND1/ITGB8/MDM2/FAS	339	3.73E-03	1.41E-02
hsa05167	Kaposi sarcoma-associated herpesvirus infection	Infectious disease: viral	CCND1/FAS/CALML6	186	5.27E-03	1.83E-02
hsa05169	Epstein-Barr virus infection	Infectious disease: viral	CCND1/FAS/MDM2	201	6.44E-03	2.15E-02

Reactome	Description	Category	geneID	size	pvalue	qvalue
R-HSA-216083	Integrin cell surface interactions	Integrin cell surface interactions	ITGB8/HSPG2/ITGAM	67	2.78E-04	3.32E-03
R-HSA-3000171	Non-integrin membrane-ECM interactions	Non-integrin membrane-ECM interactions	DDR1/HSPG2	42	2.92E-03	1.27E-02
R-HSA-1474244	Extracellular matrix organization		ITGB8/DDR1/HSPG2/ITGAM	294	2.23E-03	1.14E-02
R-HSA-6803211	TP53 Regulates Transcription of Death Receptors and Ligands	RNA Polymerase II Transcription	FAS/TNFRSF10B	12	2.32E-04	3.32E-03
R-HSA-5633008	TP53 Regulates Transcription of Cell Death Genes	RNA Polymerase II Transcription	FAS/TNFRSF10B	44	3.20E-03	1.33E-02
R-HSA-8878159	Transcriptional regulation by RUNX3	RNA Polymerase II Transcription	CCND1/MDM2	52	4.28E-03	1.55E-02
R-HSA-69416	Dimerization of procaspase-8	Apoptosis	FAS/TNFRSF10B	11	1.93E-04	3.32E-03
R-HSA-3371378	Regulation by c-FLIP	Apoptosis	FAS/TNFRSF10B	11	1.93E-04	3.32E-03
R-HSA-140534	Ligand-dependent caspase activation	Apoptosis	FAS/TNFRSF10B	16	4.20E-04	3.32E-03
R-HSA-5357769	Caspase activation via extrinsic apoptotic signalling pathway	Apoptosis	FAS/TNFRSF10B	26	1.12E-03	6.98E-03
R-HSA-5218900	CASP8 activity is inhibited	Regulated Necrosis	FAS/TNFRSF10B	11	1.93E-04	3.32E-03
R-HSA-5675482	Regulation of necroptotic cell death	Regulated Necrosis	FAS/TNFRSF10B	11	1.93E-04	3.32E-03
R-HSA-5213460	RIPK1-mediated regulated necrosis	Regulated Necrosis	FAS/TNFRSF10B	16	4.20E-04	3.32E-03
R-HSA-5218859	Regulated Necrosis	Regulated Necrosis	FAS/TNFRSF10B	16	4.20E-04	3.32E-03
