# Supplementary material for: A novel integrative multi-omics approach to unravel the genetic determinants of rare diseases with application in sinusoidal obstruction syndrome
Source: PLoS One. 2023 Apr 5;18(4):e0281892. doi: 10.1371/journal.pone.0281892 (PMC10075428; doi:10.1371/journal.pone.0281892)
Supplement: S1 File — • Text S01. Description of lymphoblastic cell models, RNA sequencing, and post-processing. • Text S02. Description of data filtering steps used in the whole-exome sequencing dataset. • Figure S01. Venn diagram showing the number of genes identified in (a) the whole-exome sequencing analysis using the VEGAS2 tool (VEGAS2), (b) the differential gene expression (DGE) from the in vitro analysis before and after treatment of lymphoblastoid cell lines with busulfan, and (c) the combined test aggregating data from both previously mentioned tests into one test statistic. • Figure S02. Polygenic risk score including 209 SNPs as output of the data analysis pipeline combining LCL expression and WES clinical association data (MAF > 0.05, p < 0.05), stratified by presence (cases) or absence (controls) of sinusoidal obstruction syndrome. (DOCX) [file pone.0281892.s008.DOCX]

**A novel integrative multi-omics approach to unravel the genetic determinants of rare diseases with application in sinusoidal obstruction syndrome**

Nicolas Waespe, MD,*^1,2,3^ Simona Jurkovic Mlakar, PhD,*^2^ Isabelle Dupanloup, PhD,^4^ Mohamed Aziz Rezgui,^5^ Henrique Bittencourt, MD, PhD,^5,6,7^ Maja Krajinovic, MD, PhD,^5,6,7^ Claudia E. Kuehni, MD, MSc, ^1,8^ Tiago Nava, MD, PhD,^2,9^ Marc Ansari, MD.^2,9^

**Supplementary material**

**Supplementary Text S01.** Description of lymphoblastic cell models, RNA sequencing, and post-processing.

***Sample preparation:***

A set of six immortalized non-malignant lymphoblastoid cell lines (LCLs; GM7056, GM12239, GM12762, GM12057, GM12489, and GM12546), acquired in 2012 from the International HapMap Consortium’s CEPH Families Reference Panel 142011/147712 (Coriell Cell Repository, Camden, NJ, USA), were used for assessing the baseline and busulfan-induced gene expression profiles. Upon arrival, the cells were immediately stored at -196 °C and were not manipulated prior to the start of experiments. At the start of the experiment, the cell lines were thawed and cultured in Roswell Park Memorial Institute (RPMI) 1640 medium (Gibco, Carlsbad, CA, USA), supplemented with 10% fetal bovine serum (HyClone, South Logan, UT, USA) and 1% Penicillin-Streptomycin (Gibco) and incubated at 37°C, 5% CO2-humidified atmosphere according to manufacturer’s recommendations.

***RNA extraction:***

Messenger RNA (mRNA) samples were extracted from LCLs after a 48h long incubation with and without 100 µM busulfan (Sigma-Aldrich, Germany) and 1% dimethylsulfoxide (DMSO; Sigma-Aldrich, Germany) using PureLink RNA Mini kit (Invitrogen, Thermo Scientific, Wilmington, DE, USA).

***RNA-seq data pre-processing step:***

Library preparation and mRNA sequencing were carried out at the iGE3 Genomics Platform – CMU, Geneva, Switzerland. Sequencing libraries were prepared from total RNA using the stranded Illumina TruSeq RNA-seq protocol with polyA enrichment. Sequencing libraries were prepared from 0.5 or 1 μg total RNA using the TruSeq stranded mRNA reagents (Illumina HiSeq 2000 library preparation kit with millions of reads [100PE]). The sequence quality was assessed with FastQC v.0.11.5 (RRID: SCR_014583) [1]. The reads were mapped to the UCSC hg19 human reference genome with the TopHat 1049v.2.0.11 software (RRID: SCR_013035) [2]. The alignments were sorted and indexed using SAM tools (RRID: SCR_005514) [3] and saved in BAM format. The table of gene counts, i.e. the number of reads mapping to each gene feature, was generated with HTSeq v0.6p1 1052 (htseq-count, RRID: SCR_005514).

***RNA-seq data post-processing step:***

The differential gene expression (DEG) analysis was performed with the statistical analysis R/Bioconductor package DESeq2 package (RRID:SCR_015687) [4]. Briefly, the counts were normalized according to the library size. The genes without any expression evidence in all samples were discarded from the analysis. The test of differential expression was performed using the general linear model (GLM) implemented in DESeq2. The genes with a false discovery rate below 0.05 were identified as being differentially expressed.

**Supplementary Text S02.** Description of data filtering steps used.

We used the following criteria to reduce genetic heterogeneity in the whole-exome sequencing (WES) dataset:

(1) individual and SNP missingness: we excluded SNPs with > 20% missing genotypes and individuals for whom the genotypes were missing for > 20% SNPs;

(2) inconsistencies in assigned and genetic sex of subjects: we checked for discrepancies between the sex of the individuals recorded in the dataset and their sex based on X chromosome homozygosity rates: we considered discrepancies as a surrogate for low-quality genotype data and removed males with X chromosome homozygosity estimate > 0.8 and females with X chromosome homozygosity estimate < 0.2;

(3) minor allele frequency (MAF): we excluded SNPs with MAF < 0.05;

(4) deviations from Hardy–Weinberg equilibrium (HWE): we excluded markers which deviated from HWE (p-value <1e−10 in cases and <1e−6 in controls);

(5) heterozygosity rate: we excluded individuals with high or low heterozygosity rates, i.e. who deviated ±3 SD from the samples' heterozygosity rate means;

(6) relatedness: we excluded all individuals with an estimated inbreeding coefficient > 0.2;

(7) ethnic outliers: we used the multidimensional scaling approach implemented in PLINK. Briefly, this method allows estimating the genome‐wide average proportion of alleles shared between any pair of individuals within the sample to generate quantitative indices of the genetic variation for each individual. The individual component scores are then plotted in 2-dimensional space to explore whether groups of individuals are genetically more similar to each other than expected. We used the data of the 1000 Genomes project (<http://www.1000genomes.org/>) as representing a population of known ethnic structures for the anchoring step. The individuals who appeared as outliers based on the multidimensional scaling analysis were removed from further analyses.

**References**

1. Wingett SW, Andrews S. FastQ Screen: A tool for multi-genome mapping and quality control. F1000Res. 2018;7: 1338. doi:10.12688/f1000research.15931.2

2. Trapnell C, Pachter L, Salzberg SL. TopHat: discovering splice junctions with RNA-Seq. Bioinformatics. 2009;25: 1105–1111. doi:10.1093/bioinformatics/btp120

3. Li H, Handsaker B, Wysoker A, Fennell T, Ruan J, Homer N, et al. The Sequence Alignment/Map format and SAMtools. Bioinformatics. 2009;25: 2078–2079. doi:10.1093/bioinformatics/btp352

4. Love MI, Huber W, Anders S. Moderated estimation of fold change and dispersion for RNA-seq data with DESeq2. Genome Biology. 2014;15: 550. doi:10.1186/s13059-014-0550-8

**Supplementary Figure S01.** Venn diagram showing the number of genes identified in (a) the whole-exome sequencing analysis using the VEGAS2 tool (VEGAS2), (b) the differential gene expression (DGE) from the in vitro analysis before and after treatment of lymphoblastoid cell lines with busulfan, and (c) the combined test aggregating data from both previously mentioned tests into one test statistic.

**Supplementary Figure S02.** Polygenic risk score including 209 SNPs as output of the data analysis pipeline combining LCL expression and WES clinical association data (MAF > 0.05, p < 0.05), stratified by presence (cases) or absence (controls) of sinusoidal obstruction syndrome.

**Legend:** LCL, lymphoblastoid cell line; MAF, minor allele frequency; SNP, single nucleotide polymorphism; WES, whole exome sequencing.
